# Long-read genome sequencing reveals complex variability in lentiviral provirus insertions in clonal CD19 CAR-T VCN reference cell lines

**DOI:** 10.1016/j.omta.2026.201873

**Published:** 2026-10-21

**Authors:** Zhiyong He, Jennifer H. McDaniel, Linhua Tian, Mahir Mohiuddin, Ningchun Xu, Lili Wang, Sheng Lin-Gibson, Justin M. Zook, Hua-Jun He

**Affiliations:** 1 Materials Measurement Laboratory, National Institute of Standards and Technology, 100 Bureau Dr., Gaithersburg, MD 20899, USA

**Keywords:** chimeric antigen receptor, CAR-T cell, lentiviral vector, vector copy number, integration site, reference material, long-read genome sequencing

## Abstract

Characterizing proviral insertions in chimeric antigen receptor (CAR)-T cells is critical for cell therapy product safety and efficacy. We generated clonal CD19 CAR-T cell lines designated VCN1–VCN5 based on RRE copies per cell measured by droplet digital PCR (ddPCR). Some clones also contained additional partial vector-derived integrations not reflected in this designation. Short- and long-read whole-genome sequencing (WGS) were used for characterizing the insertions. Long reads proved essential, fully resolving each insertion and revealing that only 3 of 10 insertions matched the expected sequence. The remainder exhibited structural variants and/or abnormalities. One particularly important structural abnormality resolved only by long reads is a 724-bp deletion of the EF-1α promoter disrupting expression of the CD19 CAR. Standard short-read and ddPCR approaches miss this deletion due to this promoter being in the unengineered human genome. These results demonstrate that these cell lines are suitable VCN candidate reference standards for 1–5 copies of full proviral DNA insertions (VCN4 and VCN5 harboring an extra *EGFP* copy) and highlight the utility of long-read sequencing in characterizing both quantity and quality of insertions in lentiviral-engineered cells.

## Graphical abstract

**Figure:**
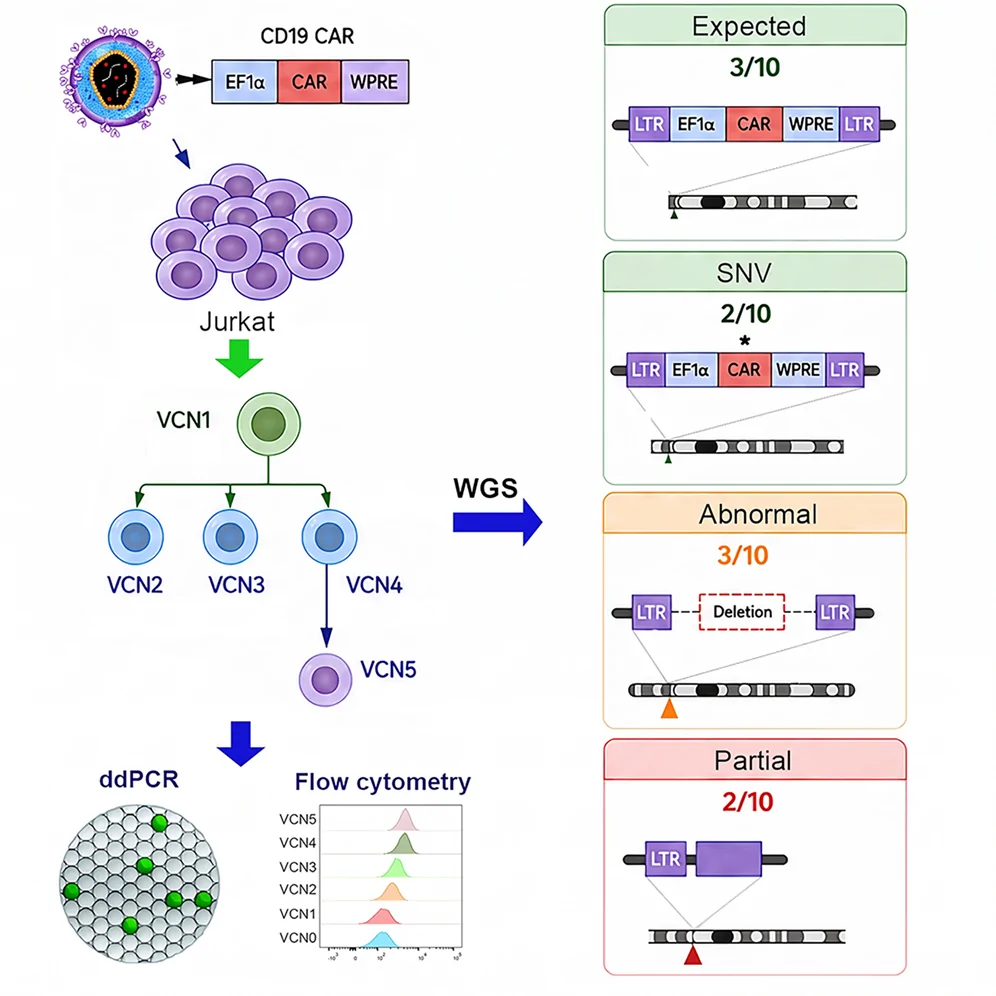

## Teaser

By establishing CD19 CAR-T reference cell lines, this study proves that quantitative vector copy number assays miss structural defects. Long-read sequencing revealed that only 3 of 10 insertions were intact, uncovering promoter deletions invisible to short-read NGS and ddPCR. Thus, long-read WGS is essential for evaluating cell therapy safety and vector integrity.

## Introduction

Chimeric antigen receptor (CAR)-T cell therapy is a revolutionary novel cancer therapy and has shown promise in leukemias, with emerging but more limited evidence in solid tumors.^1^^,^^2^^,^^3^^,^^4^^,^^5^ A significant milestone for CAR-T cell therapy was US Food and Drug Administration (FDA) approval of tisagenlecleucel and axicabtagene ciloleucel, two CD19-specific CAR-T cell products.^4^^,^^5^ Currently, there are 7 CAR-T cell therapy products approved by the FDA for treating blood cancers such as acute lymphoblastic leukemia (ALL), large B cell lymphoma, and multiple myeloma (https://www.fda.gov/vaccines-blood-biologics/cellular-gene-therapy-products/approved-cellular-and-gene-therapy-products). Many other cancer-related cell surface markers were targeted by CAR-T cell therapy in preclinical and clinical stages, such as CD22,^6^ HER2,^7^ and EGFR,^8^ inspired by the success of these CAR-T cell therapies. A CAR molecule typically contains four functional domains: an extracellular antigen-binding domain, a spacer, a transmembrane domain, and an intracellular activation domain.^9^ The T cells for therapy (either autologous or allogeneic) are genetically engineered to express the CAR protein.

Currently, all FDA-approved CAR-T cells are primarily engineered using viral transduction, such as lentiviral and gammaretroviral vectors.^5^^,^^10^ More pre-clinical research reported using CRISPR-Cas9 to engineer the T cells at specific sites, but none are yet FDA approved.^11^^,^^12^^,^^13^^,^^14^ The commonly used method for CAR-T production of therapies approved by the FDA is the lentivirus vector (LVV) transduction. LVV is a retrovirus vector derived from HIV and is able to insert the target gene into the cell genome.^15^ Thus far, 141 CAR-T and 6 CAR-NK cell therapies utilize lentivirus as the gene delivery method (www.clinicaltrials.gov as of May 2026). Advantages of the LVV method include relatively reliable and efficient gene transfer. However, LVV insertion into the target cell genome is uncontrolled, which could lead to aberrant RNA splicing.^16^ High copies of LVV integration may also cause insertional mutagenesis and/or oncogenicity.^17^^,^^18^ Multiple insertions in the same chromosome close to each other may cause the fragment between the insertions to be inverted or deleted through homologous recombination. Furthermore, the biological process of lentiviral integration (including reverse transcription of LV RNA genome and double-stranded proviral DNA insertion) is inherently error prone.^15^^,^^19^^,^^20^ Therefore, to reduce safety concerns associated with high vector copy number (VCN), it is carefully controlled during product development. Further studies of a gene therapy for hematologic cancer identified insertions within oncogenes in patients with subsequent cancer,^21^ which suggested that the identification of LVV insertion sites is important for safety. Several case reports prompted an FDA investigation and longitudinal study, which did not find evidence directly linking CAR-T cells to these cancers, though follow-up data remain limited.^22^^,^^23^^,^^24^^,^^25^

Common measurement methods for integrated LVV copy number include qPCR, droplet digital PCR (ddPCR), and next-generation sequencing (NGS), each with its own limitations. For example, an earlier report showed that the integrated VCN was often underestimated by the qPCR method.^26^ In addition, the assays developed based on each method vary from laboratory to laboratory. Targeted NGS of integration sites has been used to measure integration with short reads^27^ and long reads,^28^ but these targeted methods did not measure the full insertion sequences. Long reads have detected large structural variants at CRISPR-Cas9-engineered sites,^29^ leading the FDA to mention the use of long-reads or alternative methods for large variants (https://www.fda.gov/media/191966/download). Although CRISPR-Cas9 results in different genomic changes than LVVs, long reads also can resolve LVV insertion events difficult to see with short reads. For example, targeted long reads identified HIV-associated internal proviral deletions, and a modified approach recently found promoter deletions in LVVs as well by sequencing full integration sequences.^20^^,^^30^ For these reasons, the community has called for reference samples for assay validation, optimization, and benchmarking.^31^^,^^32^

We previously developed NIST RGTM 10268 using an LVV containing only a cassette for GFP expression.^33^ The clonal cell lines contained 0–4 copies of proviral DNA per cell, and all insertion sites were identified.^33^ Here, we developed an additional set of VCN candidate reference material for the following reasons. First, the actual lentiviruses used in the clinical setting such as cell and gene therapy usually have longer RNA genomes. Second, since higher VCN samples make it more difficult to reach consensus measurement results,^34^^,^^35^ higher VCN clonal cell lines need to be included in the candidate reference materials for benchmarking the measurements. In a World Health Organization (WHO) interlaboratory study, measurements of cells with an estimated VCN of 10.67 varied widely from 6.15 to 13.6, and few insertion sites were identified.^35^ This broad range highlighted the challenges of measuring VCN for higher copies of high copy number integrated vectors. Moreover, the FDA recommends maintaining a VCN of fewer than 5 copies per cell. Third, there is no CAR expression cassette in the previous candidate reference materials for potential functional CAR cytotoxicity assays. Finally, the proviral DNA sequence of previous candidate reference materials could not be disclosed due to intellectual property restrictions.

To address this unmet need, we developed a set of clonal cell lines with 1–5 copies of integrated CD19 CAR LVV and deeply characterized the integration sites using orthogonal methods. The precise sequences of each LVV insertion in at least one clone were measured with long-read whole-genome sequencing, revealing functionally important structural anomalies in the LVV insertion sequences in the host genome relative to the expected provirus DNA sequence. These clonal cell lines could serve as reference materials for CAR-T cell VCN measurements, insertion site identification, and identification of potential random insertions.

## Results

### Stable clonal cell lines with 1–5 copies of integrated CD19 CAR

CD19 CAR lentivirus-transduced Jurkat cells were selected with 10 μg/mL of bleomycin for the first 3 passages and then with 5 μg/mL of bleomycin. The design of CD19 CAR lentivirus is illustrated in Figure 1A. The bleomycin-resistant cells were subjected to single-cell clone printing and screening. The single-cell clones were screened by ddPCR using RRE assay and S2 as single-copy control gene. Initial transduction using MOI = 1 and 5 resulted in mostly VCN1 clonal cell lines (detected copy number of RRE/detected copy number of S2 × 2 = 1). We only obtained two VCN2 (detected copy number of RRE/detected copy number of S2 × 2 = 2) clonal cell lines with no GFP expression. We chose one of the GFP-positive VCN1 clonal cell lines for lentiviral transduction to generate higher VCN clonal cell lines (as shown in Figure 1B). Finally, we used VCN4 clonal cell line to generate VCN5 clonal cell line (Figure 1B). We analyzed the cells by flow cytometry to detect the GFP and CD19 CAR expression level. The first-round screening resulted in many clones that either lost GFP expression after a few passages or did not show correlation of CD19 CAR expression with the integrated VCN (data not shown). Thus, we did a second lentivirus transduction on the selected VCN1 clone based on GFP expression. With the same screening process, we obtained VCN2, VCN3, and VCN4 clones that had GFP and CD19 CAR expression correlated with the integrated VCN. We further transduced VCN4 with the same LVV to obtain VCN5 with one additional integration. The GFP expression levels were similar between all the cell lines (Figure 2A); however, the CD19 CAR expression levels were correlated with the integrated VCN (Figures 2B and 2C) except VCN1 cells, which exhibited GFP-positive but no detectable CD19 CAR expression.

**Figure 1:**
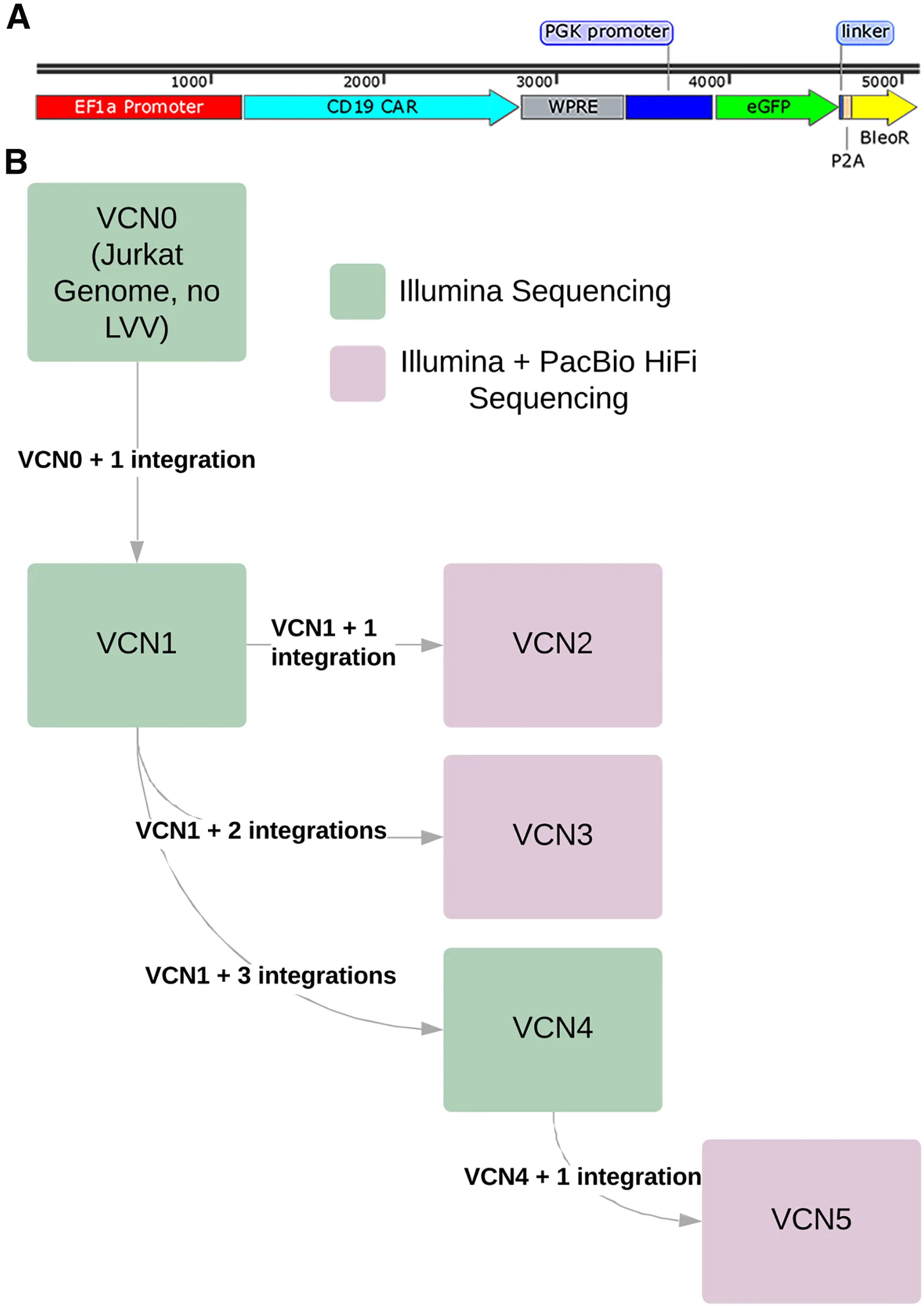
CD19 CAR vector design and schematic view of VCN clonal cell line generation strategy (A) Schematic view of CD19 CAR transfer vector design. (B) Schematic view of VCN clonal cell line generation strategy. VCN was designated by the detected copies of RRE per cell. Short-read sequencing (Illumina) identified integration loci across all clones, whereas long-read sequencing (PacBio) captured the integration loci while also resolving the complete integrated sequence and its integrity. Direct long-read sequencing was performed selectively on VCN2, VCN3, and VCN5. The full integration structures for the intermediate clones, VCN1 and VCN4, were inferred from the shared long-read sequencing data of their respective descendant clones, because no differences were seen in the short-read data for these integration sequences.

**Figure 2:**
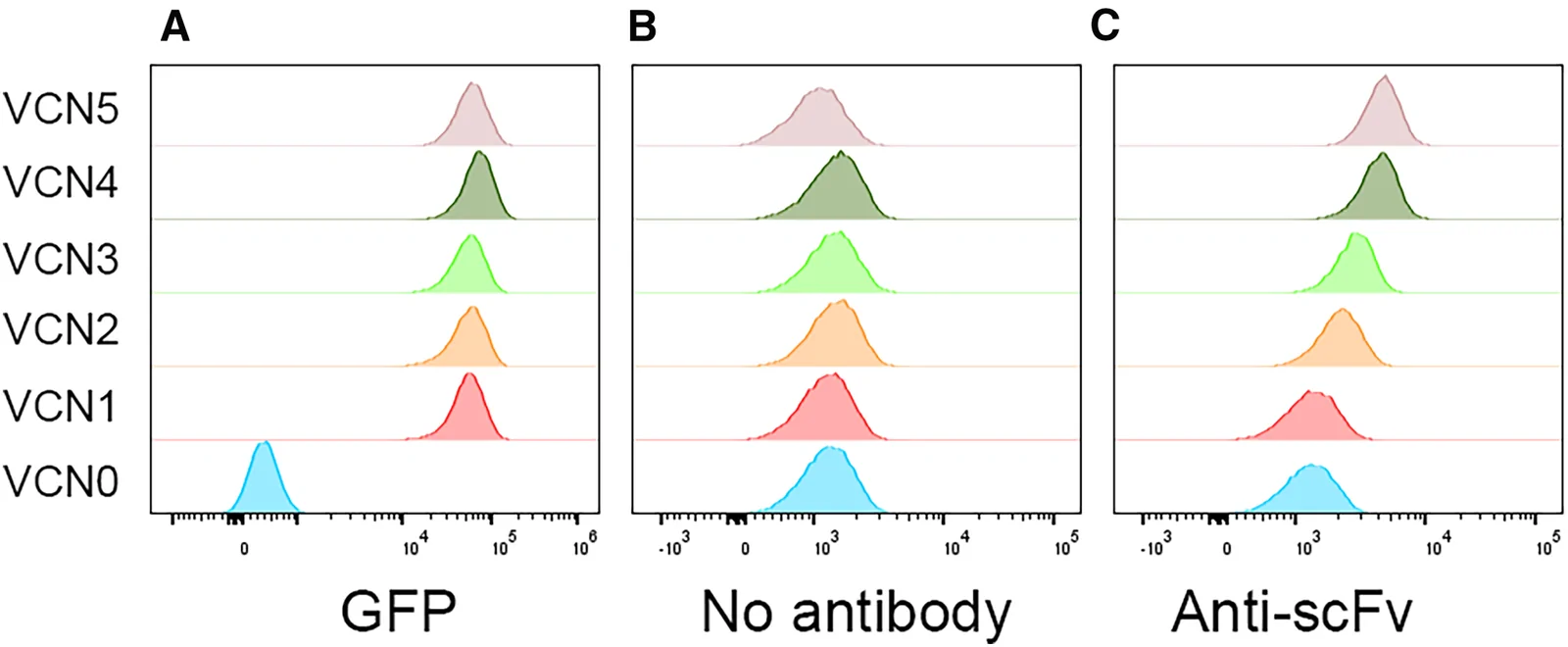
GFP and CD19 CAR expression in VCN clonal cell lines (A) GFP expression of each clonal cell line analyzed by flow cytometry. (B) No primary antibody controls for analyzing CD19 CAR expression by flow cytometry. (C) CD19 CAR surface expression on each clonal cell line. Data are representative of 3 independent experiments.

### Measurement of VCN by different PCR targets

We utilized the assays developed based on the lentivirus elements (woodchuck hepatitis virus posttranscriptional regulatory element [WPRE], RRE, Psi, and long terminal repeat [LTR] assays were published previously)^36^ and the CD19 CAR. Single-copy reference locus S2 and *RPL32* (ribosomal protein L32)^37^ were used as single-copy reference genes. Most of the assays resulted in the expected copy number for each VCN cell line (Figure 3). However, VCN3 appeared to have an additional copy for WPRE, and both VCN4 and VCN5 appeared to have an extra copy of *EGFP*, results corroborated below by long-read NGS.

**Figure 3:**
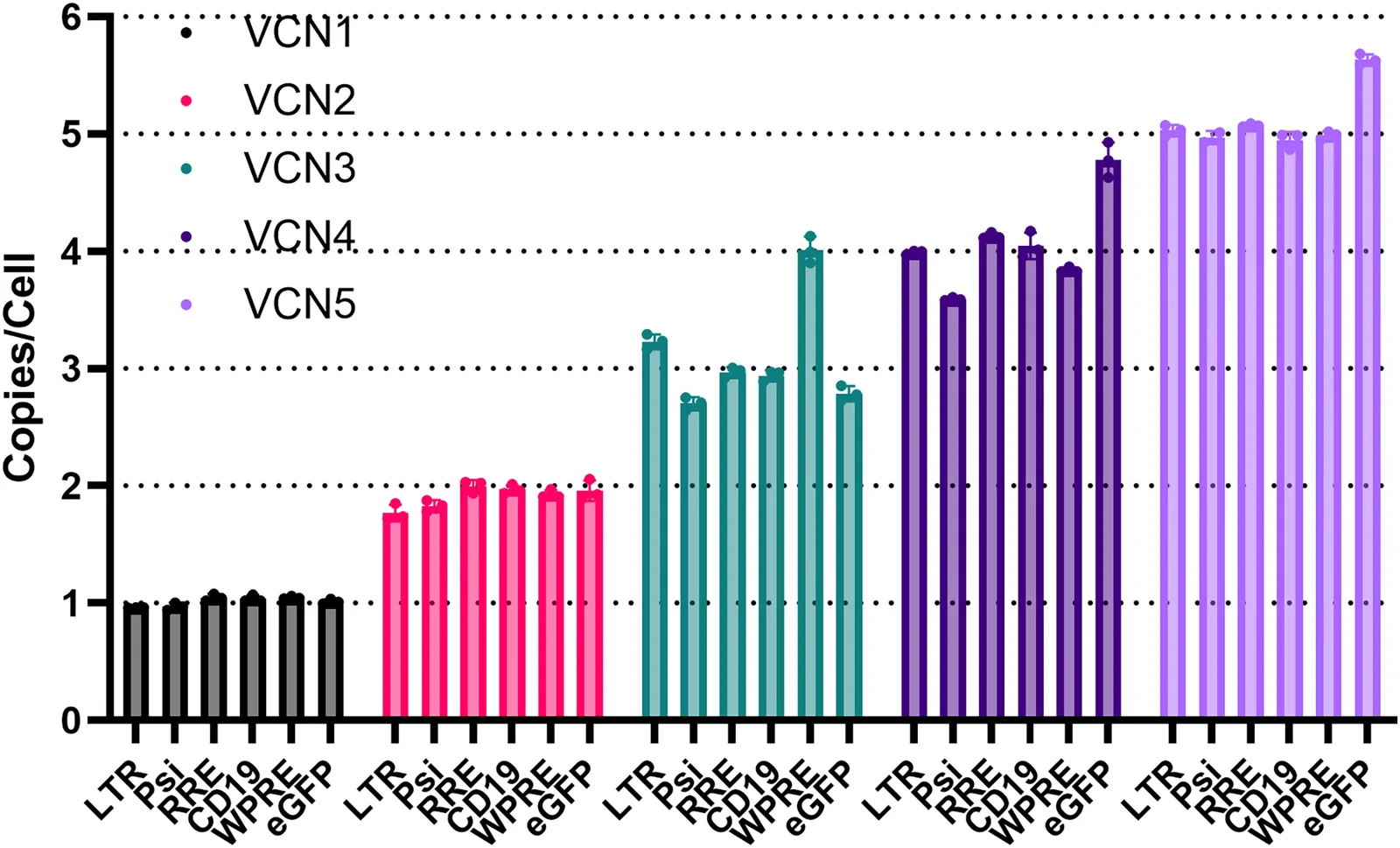
VCN detected by 6 different assays in each clonal cell line Representative of 3 experiments. Error bars represent standard deviation (*n* = 3). *y* axis: vector copy number (copies/cell).

### Stability of the clonal cell lines

The stability of the cell lines was assayed by passaging the cells up to passage 42. Genomic DNA was extracted from the cells every week (3 passages per week) for 8 weeks and was analyzed by ddPCR to quantify the VCNs based on RRE and CD19 CAR. As shown in Figure 4, the VCN cell lines exhibited minimal variations for atleast 8 weeks, which indicated that the cell lines remained stable for the VCN measurements.

**Figure 4:**
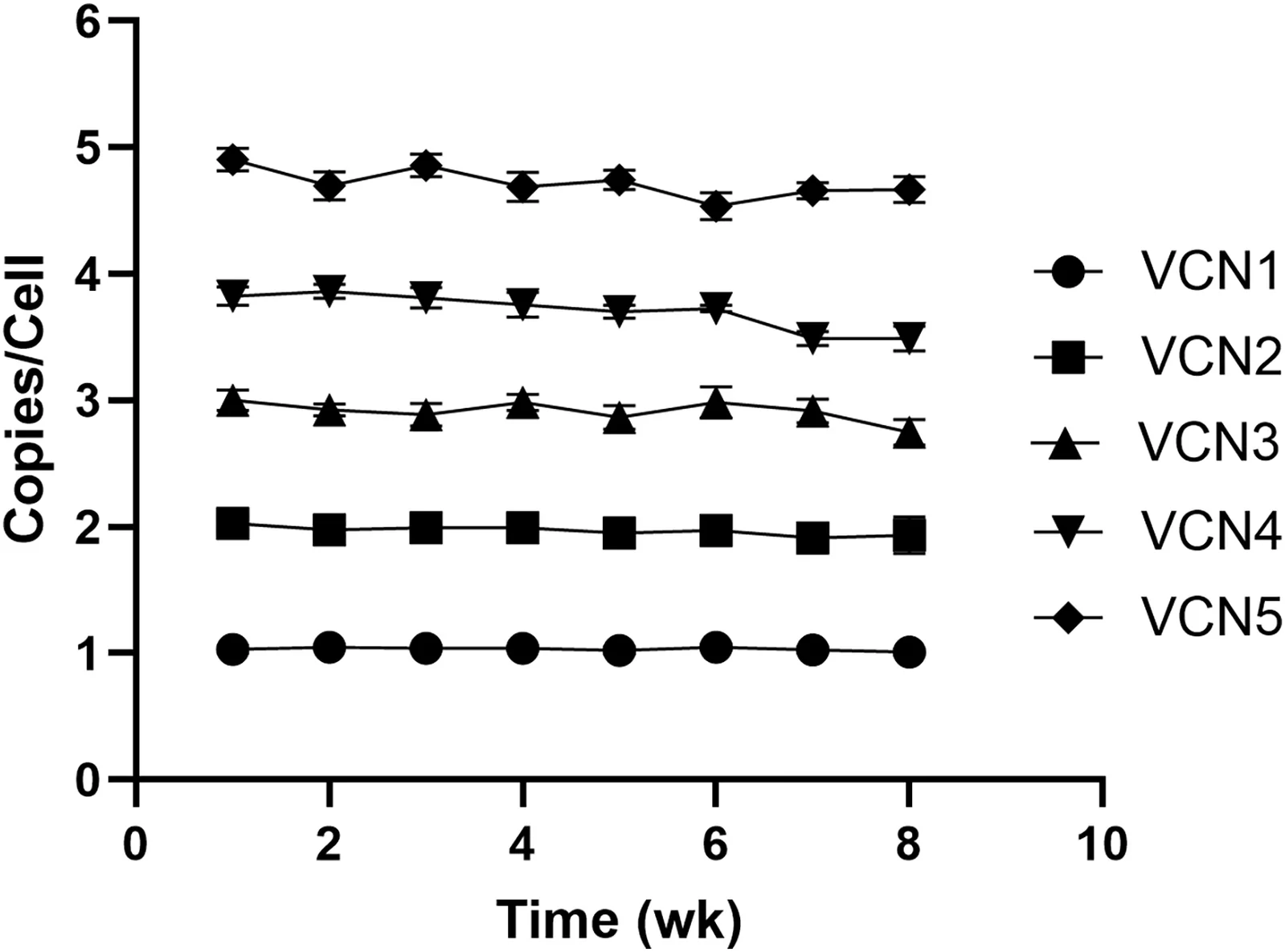
Stability of clonal cell lines VCN of each cell line was determined by ddPCR using RRE and CD19 CAR target assays. Data are representative of three independent experiments. Plotted values represent the mean ± SD of technical triplicates for both assays within a single experiment (*n* = 6 total technical replicates per timepoint). *x* axis: time (weeks).

### Long-read sequencing identifies structural and small variants in provirus integrations

A combination of long-read and short-read whole-genome sequencing identified and confirmed precise integration sites, target site duplicated sequences, and variation within each inserted sequence in the Jurkat cell genome (Figure 5B). All insertions occurred in introns except for one in the 3′ UTR of *HNRNPR*. Interestingly, of the 10 unique integration events, only 3 contained the expected sequence, with others containing substitutions, deletions, and/or duplications relative to the expected sequence. VCN1 clonal cell line showed one copy of proviral DNA inserted in the minus strand of chromosome 17 at position 78,730,345. Long-read sequencing revealed a 724-bp deletion in the EF-1α promoter region (Figure 5B), which explained why VCN1 cells did not express CD19 CAR (Figure 2C). This deletion is not visible by short-read sequencing due to the low mapping quality caused by high identity with the EF-1α promoter in the reference. Since VCN2–5 clonal cell lines were all derived from VCN1, all the cell lines had a copy of the 724-bp-deletion EF-1α promoter proviral DNA on chromosome 17 (Figure 5B). As expected, VCN2 cells contained an additional insertion. However, it harbored four unexpected substitutions. Although both short reads and long reads detected these substitutions, only long-read data provided sufficient context to confirm that all four were located within the second insertion (Figure 5B, yellow lines). In VCN3 cells, one of the new proviral insertions had the precise expected sequence. However, the other proviral insertion had a mostly expected sequence except for one single-nucleotide variant (SNV), and an additional 804-bp sequence on the 3′ end of the proviral insertion. This additional sequence contains a 778-bp duplication of a portion of the WPRE sequence, followed by 2 bp (GT) and 24 bp of inverted 3′ LTR sequence. This 778-bp duplication accounts for the additional WPRE copy detected by the WPRE ddPCR assay in the VCN3 cell line (Figure 3). Interestingly, both short- and long-read whole-genome sequencing data revealed an additional non-functional 263-bp insertion of predominantly LTR sequence that was not detected by ddPCR assay. Analysis of VCN4 revealed one new proviral insertion exhibiting the expected sequence, a second containing a single-nucleotide substitution, and a third harboring both a substitution and an additional 41-bp sequence downstream of the 3′ LTR. This additional 41-bp sequence comprises 15 bp of unknown origin and 26 bp derived from the 3′ LTR (Figure 5B). Furthermore, short- and long-read sequencing data identified an additional 2,070-bp integration corresponding to the 3′ region of the proviral DNA. This fragment contained a 1-bp deletion and encompassed the *EGFP* gene, thereby accounting for the extra copy of *EGFP* detected by ddPCR assay in the VCN4 cells (Figure 3). The VCN5 cell line contained all the insertions previously identified in VCN4, alongside one additional insertion exhibiting the precise expected sequence (a representative visualization utilized for manual curation is provided in Figure S1). Furthermore, the allele frequencies of SNVs and insertions/deletions (indels) within the LVV sequences, as determined by both short- and long-read sequencing, corresponded with the expected fraction of variant-containing copies (Table S1). All reported integrated sequences derive from the HiFi pbmm2-pbsv calls, starting with the 4–5 bp of duplicated integration site sequence, and are available in supplemental information. In summary, whole-genome sequencing revealed alterations within multiple inserted sequences, thereby elucidating the unexpected PCR results.

**Figure 5:**
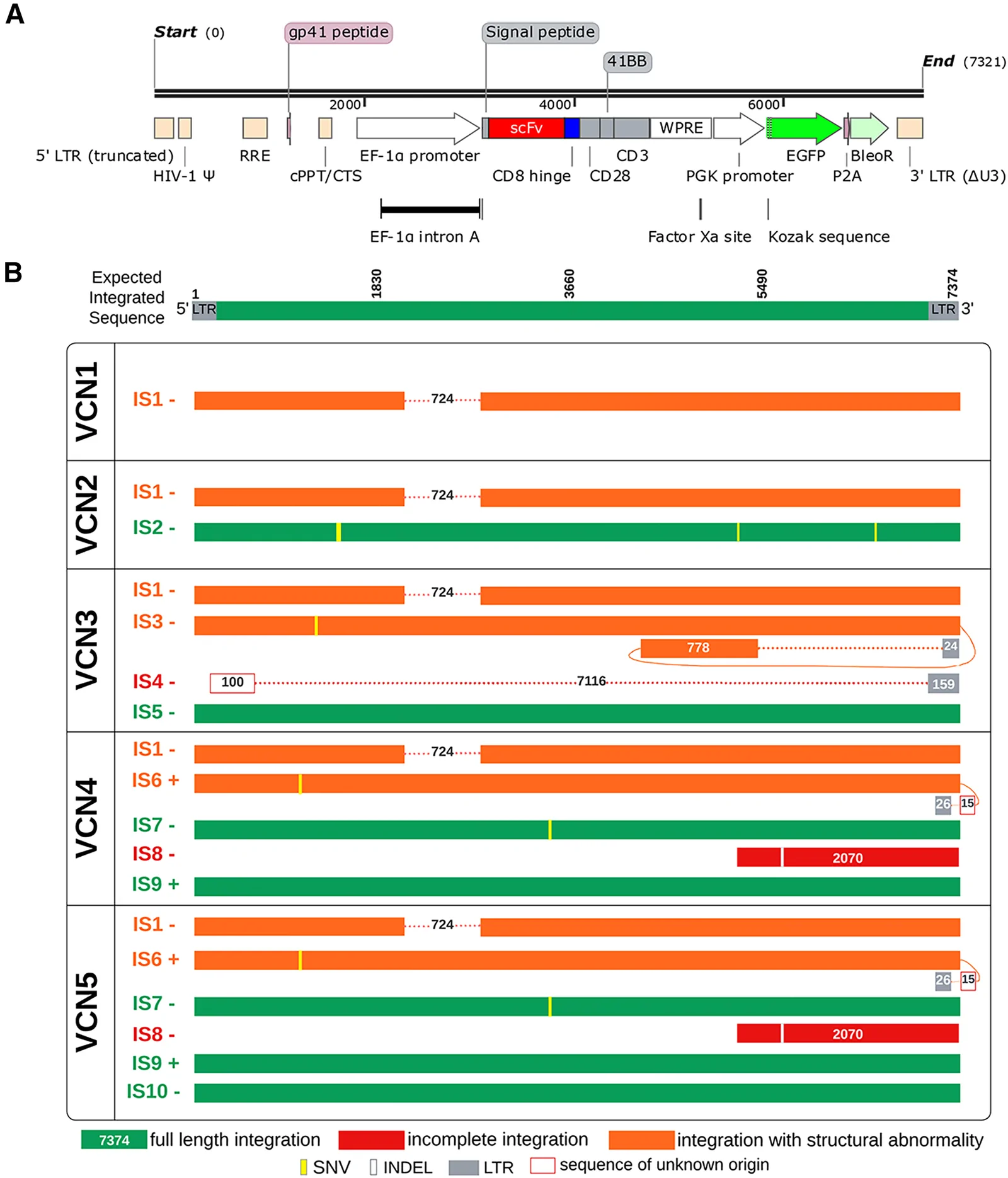
Schematic diagram of lentiviral DNA insertions and variants inside the insertions in each clonal cell line (A) Schematic diagram of each element of the lentiviral DNA insertion. The truncated 5′ LTR is replaced by the 3′ LTR during reverse transcription. (B) Schematic diagram of proviral DNA integration sites across VCN1–VCN5 characterized using short- and long-read sequencing. Integrations are categorized as full length (green), structurally abnormal (orange), or incomplete (red), with genomic orientation indicated by +/− symbols. Horizontal dotted lines signify missing regions, while vertical yellow and white lines denote SNVs and INDELs, respectively. Gray boxes represent LTR sequence. Curved orange lines connect unexpected sequence post expected sequence. More information about each integration is in Table 1 and Tables S2 and S3.

**Table 1:** Summary of all integration sites and integration observations

Integration identifier (IS_ID)	VCN	Chromosome	Start	Integration site sequence	Integrated sequence integrity	Integrated sequence length
IS1	VCN1, VCN2, VCN3, VCN4, VCN5	chr17	78,730,345	GAAAC	large 724-bp deletion in the middle of the integrated sequence (bases 1,917–2,640 of the expected sequence) in the EF-1α promoter region.	6,655
IS2	VCN2	chr19	21,610,872	CCCCC	four SNVs identified in integrated sequence at expected sequence positions 1,692:A>T, 1,693: C>T, 4,878: G>C, and 6,601: G>A. Other than SNVs, no other abnormalities.	7,379
IS3	VCN3	chr1	23,307,863	ACAGG	structural abnormality at the 3′ end of a full-length integration. The abnormality contains additional sequence composed of a large duplication, corresponding to expected integration sequence positions 4,416–5,193 (778 bp), followed by 2 bp (GT), and then 24 bp of the 3' LTR sequence (AGTCAGTGTGGAAAATCTCTAGCA). Within the full-length portion of this integration, there is an SNV T>A at position 1,450.	8,183
IS4	VCN3	chr8	99,830,407	AAAC	a short 263-bp integrated sequence primarily composed of LTR sequence was identified. The sequence begins with a 4-bp duplication of the chr8 integration site. Following this duplication, 159 bp correspond to LTR positions 7,216–7,374 of the expected integration. The remaining 100 bp are less clearly defined but appear to include a mixture of unknown and LTR-derived sequences (33 bp unknown, 21 bp possible LTR, and 46 bp unknown using BLAST). BLAST of the 79-bp “unknown” sequence was performed, and no significant similarities were found.	263
IS5	VCN3	chrX	103,930,456	AATAC	expected integration sequence	7,379
IS6	VCN4, VCN5	chr1	243,273,502	ACCCC	SNV G>A at position 1,066 in full integrated sequence. End of full integrated sequence has an additional 41 bp. This sequence appears to be 15 bases of unknown origin followed by the first 26 bp of the LTR sequence in reverse orientation.	7,420
IS7	VCN4, VCN5	chr3	18,115,277	TACTC	SNV G>A at full integrated sequence position 3,649. Other than SNV, no other abnormalities.	7,379
IS8	VCN4, VCN5	chr6	15,601,040	unclear, possibly AG	integrated sequence is incomplete (2,070 bp) and only contains bases corresponding to positions 5,306–7,374 of the expected integration sequence. It appears that 22 bases of chr6 were deleted due to the insertion (chr6:15,601,040–15,601,062). 22 bases out of 24 bases, “CGAAGACGCGGAAGAGGCCGCA,” align to the expected integration sequence, but the final two bases, “AG,” do not. These two bases are part of chr6 sequence adjacent to the deleted portion of chr6, so they may have been duplicated upon integration. There is also deletion of a “C” at position 5,579 in a C homopolymer.	2,070
IS9	VCN4, VCN5	chr6	24,825,065	GTACT	expected integration sequence	7,379
IS10	VCN5	chr5	39,044,696	TTCAC	expected integration sequence	7,379

The IS_ID corresponds to chromosome integration site; IS_IDs can be shared across VCN. Integration positions on the chromosome (start) are the first base of the integration site; they are not left aligned. Positions of abnormalities are relative to the expected proviral sequence, not the position within the sequence reported in the fasta format. A full summary of all integrations and visualization of the integration sites are listed in Tables S2 and S3 and Figure S3, respectively.

Analysis of both short- and long-read whole-genome sequencing data also revealed the absence of chromosome Y in VCN1–VCN5 cell lines. A previous report^38^ and our G-band karyotype analysis (Figure S2A) indicated that a subpopulation of Jurkat cells lacks chromosome Y (7 of 20 metaphase spreads). In this work, sequencing and G-band karyotyping confirmed this absence of chromosome Y in the VCN1 cell line (Figure S2B). Because all the other VCN cell lines were derived directly from VCN1, this shared lineage accounts for the absence of chromosome Y across all the VCN1–VCN5 clonal cell lines.

## Discussion

In this study, we generated deeply characterized clonal cell lines as reference samples for LVV integration site, quality, and copy number. With short- and long-read whole-genome sequencing, we identified all LVV integration sites and sequences, including some small segments of the LVV in the cell lines (Figure 5).^39^ Long-read WGS (whole-genome sequencing) uncovered the structural complexity of LVV integrations that were invisible to standard short-read methods. While short-read sequencing is effective for identifying general integration sites, it often fails to resolve full insertion sequences or detect large internal rearrangements due to low mapping quality in repetitive or high-identity regions and an inability to resolve full insertions. A primary example in this study is the characterization of all the integration sites present in the clonal cell lines, where long-read sequencing revealed a 724-bp deletion in the EF-1α promoter region. This specific deletion explained why the VCN1 cells failed to express the CD19 CAR protein despite the presence of the proviral DNA. This was invisible to short-read sequencing because the deletion occurred in a region with high identity to the native EF-1α promoter in the human reference genome. Beyond simple deletions, long-read data identified complex variants across the clones, revealing that only 3 of 10 unique integration events actually contained the precise expected sequence. The remaining integrations featured unexpected substitutions, deletions, and duplications, such as the 778-bp duplication found in a VCN3 insertion and a 2,070-bp partial insertion in VCN4 that included an extra copy of the *EGFP* gene. These findings highlight that LVV integration is not always a clean, full-length process and that long-read sequencing is essential for the high-resolution characterization required to validate reference materials and may help ensure the safety of CAR-T cell therapies. One of the possible mechanisms of the deletion of 724 bp in the EF-1α promoter is that the host-recognized cryptic splicing during viral RNA transcription in packaging cells generates truncated genomes that are subsequently packaged and integrated. If such truncated species constitute a certain subpopulation in the given LVV preparation batch, similar deletion patterns would likely recur in independent transductions using the same LVV preparation. It is unclear whether the cryptic splicing is specific to promoter sequence since Kaiser et al. revealed deletions in both the human ubiquitin C promoter and the EF-1α promoter.^30^ Whether vector optimization can eliminate the cryptic splicing motifs in the LVV warrants further investigation. Ultimately, these findings further support developing a standardized reference LVV backbone along with genomic reference cell lines, which could enable the field to systematically decouple vector-intrinsic sequence instability from host cell- or assay-driven variations.

To echo the findings in these CAR VCN materials, we also found SNVs in the proviral DNA regions in VCN4, VCN2, and VCN1 samples of NIST Research Grade Test Material (RGTM) 10268^33^ and a 648-kb genomic deletion near the integration site Chr14: 22 564 447 in the VCN3 sample (unpublished data from the RGTM 10268).

There are some limitations of this study. First, we generated our clonal VCN cell lines using Jurkat cells, a leukemia cell line known for inherent genomic instability, including frequent mutations, chromosome rearrangements, and single-nucleotide polymorphisms.^40^ Because of this instability, it is possible that the variant lentiviral DNA insertions we observed were a by-product of the host cells’ tendency to rearrange or truncate DNA during or after integration. However, we did not observe partial lentiviral DNA insertions in our previous study,^33^ which indicated that the likelihood of Jurkat cell genome causing these specific partial insertions is low. In addition, a new targeted long-read approach identified similar promoter deletions in a variety of non-clonal CAR-T products.^30^ In the future, since we established a novel method for immortalizing human primary T cells,^41^ we plan to collect explicitly consented patient primary cells to establish immortalized T cell lines, which would minimize the possibility of rearrangements or mutations in the cell line. Second, this set of materials has yet to be validated by multiple laboratories to prove its utility and commutability.

### Conclusion

We developed a set of VCN clonal cell lines as candidate reference samples, aimed to assure confidence in measurements of one to five LVV copies, as well as integration sites and sequences to evaluate insertion quality.

## Materials and methods

### Establishment of CD19 CAR-T VCN standard cell lines

Anti-CD19 CAR-T lentivirus transfer vector was designed at NIST based on the third generation of lentivirus packaging system.^1^ CD19 CAR vector design is illustrated in Figure 1A. Briefly, the *CD19* CAR coding sequence was driven by an EF-1α promoter. A WPRE sequence was placed downstream of the *CD19* CAR coding sequence. The *EGFP* coding sequence and bleomycin-resistant sequence were separated by a P2A sequence under the same PGK promoter. The transfer vector sequence was validated by Sanger sequencing. The lentivirus particles were packaged by VectorBuilder (Malvern, USA). Jurkat cell line (E6-1), purchased from ATCC (Cat# TIB-152, Manassas, VA), was transduced at different multiplicity of infection (MOI = 1, 5, and 10) to generate the VCN cell lines. The transduced cells were then selected with a bleomycin mass concentration of 10 μg/mL in RPMI-1640 growth medium supplemented with 10 % fetal bovine serum (FBS, Thermo Fisher, Cat# A5670402) for selecting the positive integration for the first 3 passages and then with 5 μg/mL of bleomycin. The selected positive single cells were deposited into each well of 96-well plates by using Cytena Single-Cell Printer (Cytena) to generate the clonal cell lines. The clonal cell lines were screened by ddPCR using RRE ddPCR assay^36^ and S2 as single-copy reference locus or *RPL32* as the reference gene. The VCN cell lines were then further expanded and characterized.

The clonal CD19 CAR vector copy-number candidate reference cell lines were generated from ATCC TIB-152 (Jurkat E6-1) and are therefore subject to applicable ATCC material-transfer restrictions. A request to deposit the VCN1–VCN5 clones with ATCC has been submitted and is currently under review, with the goal of making these reference cell lines broadly available through ATCC.

### ddPCR

Integrated VCN was measured by ddPCR using the common third-generation lentiviral vector elements, LTR, Psi, RRE, and WPRE (target elements). The primer and probe sequences are listed in our previous publication.^36^ Additional target genes *EGFP* and *CD19* CAR were also used to measure the VCNs. Bio-Rad ddPCR Supermix for probes (no dUTP) was purchased from Bio-Rad (Cat# 186–3024, Hercules, CA). Genomic DNA (gDNA) was extracted from each cell line using Quick-DNA Miniprep Kit (Zymo Research, Cat # D3024). 5–20 ng of gDNA was used per ddPCR reaction; the same amount of DNA template was used for the target genes and reference genes (S2 or *RPL32*) in each reaction. VCN was calculated as the ratio of the lentiviral vector elements to the reference gene determined with the Bio-Rad QX200 ddPCR system following manufacturer’s instructions.^33^ VCNs were calculated by the following formula:

$$\text{VCN}=\frac{Copies\;of\;target\;gene}{Copies\;of\;reference\;gene}\times 2$$

VCN results at the screening stage using RRE and S2 assays were assigned to the clonal cell lines. For example, if a clonal cell line had copies of RRE/copies of S2 × 2 = 3, this clonal cell line was designated as VCN3.

### Flow cytometry analysis

The expression of EGFP and CD19 CAR in the clonal VCN cell lines was analyzed by flow cytometry. The cells (5 × 10^5^ cells per sample) were washed with PBS containing 10 % FBS (fluorescence-activated cell sorting [FACS] buffer) once and resuspended in 100 μL of FACS buffer. The cells were then incubated with 2 ng of antibody, biotinylated anti-FMC63 scFV antibody (ACROBiosystems, Cat# FM3-BY45), on ice for 30 min. After 30 min, the cells were washed with 1 mL of FACS buffer once and resuspended in 100 μL of FACS buffer and 100 ng of APC-conjugated streptavidin was added to each sample and further incubated on ice for 30 min. The cells were washed once with FACS buffer, resuspended in 300 μL of FACS buffer, and then subjected to flow cytometry on a CytoFLEX LX Flow Cytometer. Cells incubated exclusively with streptavidin-APC in the absence of primary antibody incubation served as no-antibody controls. The experiment was repeated three times to confirm the repeatability of the results.

### NGS

The lentiviral vector integration sites in parental Jurkat cell line genome were determined by whole-genome sequencing analysis. Genomic DNA extracted from each cell line was sent to Novogene for short-read Illumina library preparation and whole-genome sequencing. Whole-genome long-read PacBio sequencing of VCN2, VCN3, and VCN5 was performed by Maryland Genomics. The raw sequencing data were analyzed at NIST to identify copy numbers of the integrated provirus sequence and their precise integration sites. Whole-genome sequencing data were deposited at the NIH/NCBI/SRA BioProject: PRJNA1282768 (Biosamples: SAMN49572894, VCN0; SAMN49572895, VCN1; SAMN49572896, VCN2; SAMN49572897, VCN3; SAMN49572898, VCN4; and SAMN49572899, VCN5.)

### NGS read alignment to reference genome

We used short- and long-read whole-genome sequencing (Illumina 2 × 150 bp and PacBio HiFi, N50 range of 18,751–22,133 bp) to identify LVV integration sites (ISs) in the Jurkat cell genome. Reads were mapped to a modified reference genome (GRCh38-GIABv3_plusInsert-with-3primeLTR_flanks.fasta). Specifically, the expected 7,374-bp LVV sequence was added as an additional chromosome to the GIABv3 (https://ftp-trace.ncbi.nlm.nih.gov/ReferenceSamples/giab/release/references/GRCh38/) GRCh38 human reference genome, which masks false duplications and adds decoy sequences from CHM13.^42^ Unlike the original LVV RNA sequence (7,321 bases), the LVV sequence in our reference has identical 5′ and 3′ LTRs because during reverse transcription, the U3 region of the viral genome is copied to the 5′ LTR (7,374 bp).^43^ Both long and short reads were mapped to the modified reference using BWA-MEM (v.0.7.17, https://arxiv.org/abs/1303.3997) with default parameters. The resulting alignment files (.sam) were then converted to .bam files, sorted, and indexed with Samtools (v.1.9)^44^ view, sort, and index, respectively. Duplicates were then marked with Picard (v.2.18.29) MarkDuplicates (https://broadinstitute.github.io/picard/) for short reads. Alignment post processing steps used tool default parameters. Additionally, long reads were mapped to the modified reference using pbmm2 (v.1.13.1, https://github.com/PacificBiosciences/pbmm2), a minimap2^45^ wrapper for PacBio reads, using default parameters. BWA alignments of Illumina and PacBio reads resulted in approximately 27× coverage. Pbmm2 alignments of PacBio reads resulted in approximately 38× coverage.

### Variant calling

Variant calling was performed to confirm the expected number of integration sites (ISs) in the Jurkat cell genome, identify the integration locations in GRCh38, and compare the actual inserted sequence to the expected LVV sequence. Insertions were identified using two distinct structural variant (SV) callers: Delly (v.1.2.6)^46^ for short reads data and PacBio pbsv (v.2.9.0, https://github.com/PacificBiosciences/pbsv) for long read sequencing data. Variant calling was performed with the BWA short-read alignments and pbmm2 long-read alignments against the modified reference.

### Identification and confirmation of putative integration sites using NGS

To identify putative integration sites, Delly was used to identify translocations and breakpoints when variant calling with BWA-aligned short reads resulted in passing SVTYPE=BND (breakend) on the LVV chromosome with ALT sequence identified on a different chromosome. Putative integration sites were then confirmed by manually curating sites using all alignments and variant calls with the Integrative Genome Viewer (IGV v.2.16.2). For a given VCN, the VCN alignments, variant calls, and modified reference genome were loaded into an IGV session. VCN0 alignments were also loaded in to serve as a negative control, as no integrations are expected in this sample. All alignments to the LVV chromosome were first examined to identify reads that were partially or fully aligned to the expected LVV sequence. BWA short-read alignments were first “grouped by chromosome” to quickly visualize where short-read paired mates map to other chromosomes. It was expected that this grouping would align with ALT chromosomes identified *as BND calls with* Delly. Each integration site had an increase in short-read coverage for the 4–5 bp that are duplicated around the LVV inserted sequence during repair of the staggered cuts. While paired-end short-read alignments were useful for identifying integration sites, they *cannot generally confirm the* expected full integration of the LVV sequence. To confirm full integration, PacBio long-read alignments and variant calls were used. To cover all possible integrations, VCN2, VCN3, and VCN5 were sequenced with PacBio. Karyotyping demonstrated that this Jurkat clone was near diploid in most cells except for deletions of chromosome Y and the p arm of chromosome 2; so all reads from one haplotype will support the integration at a given site. For PacBio, BWA alignments generally have split reads at integration sites, where part of the read maps to the GRCh38 reference chromosome and the remainder to the LVV chromosome as a supplementary alignment. In contrast*,* pbmm2 has more reads aligning across the integration site with an insertion that corresponds to the LVV inserted sequence, with no supplementary alignments to the LVV chromosome if the read contains the insertion. Pbsv insertion variant calls at the pbmm2 PacBio alignment integration sites were used to confirm complete insertion of the LVV sequence. To confirm integration of the LVV sequence and that no partial integrations were missed, all pbsv identified insertions greater than 50 bp were aligned back to the modified reference to determine if they aligned to the LVV sequence. These alignments were visualized in IGV. All previously identified integrations were confirmed, and no additional integrations were present.

SNVs and a 1-bp deletion were identified within the integrated sequences when pbsv insertion calls aligned to the LVV chromosome in the modified reference. We evaluated support for the putative variants in PacBio and Illumina reads. Generally, if a variant was present, the fraction of reads supporting the variant should be consistent with the fraction of insertions within the genome containing the variant.

## Data and code availability

Whole-genome sequencing data are available on public databases (NCBI Bioproject accession: PRJNA1282768). The National Institute of Standards and Technology Research Protections Office reviewed the protocol (MML-2024-0377) for this project and determined it is “not human subjects research” as defined in 15CFR27, the Common Rule for the Protection of Human Subjects. The Jurkat cell line was derived from tissues from a 14-year-old with acute T cell leukemia in the 1970s. The cell line was established prior to the current research use consent process. In 2013, The Cancer Genome Atlas (TCGA) steering committee administered by NCI and NHGRI at the NIH decided that the benefits outweighed the risks for making genomic data fully public without controlled access for Jurkat and other widely used, commercially available cell lines in the Cancer Cell Line Encyclopedia (https://www.cancer.gov/ccg/research/structural-genomics/tcga/history/policies/ccle-open-release-justification.pdf). For the Jurkat cell line, short-read whole-genome sequencing data were previously published (https://bmcgenomics.biomedcentral.com/articles/10.1186/s12864-018-4718-6) without controlled access in public databases, so there is minimal additional risk of reidentification for publishing the long-read whole-genome sequencing data in this manuscript. To make progress toward similar cell lines with explicit consent for public genome data sharing and cell line development, NIST is currently working toward establishing T cell lines and other types of cell lines from individuals who have provided consent under the Personal Genome Project (https://www.pnas.org/doi/abs/10.1073/pnas.1201904109). This consent includes permission to share genomic data and cell lines publicly for open research and commercial use, understanding the risks associated with this. For these reasons, the NIST Research Protections Office determined that the whole-genome sequencing data generated in this work could be made publicly available.

## Acknowledgments

This work is solely funded by 10.13039/100000161NIST intramural fund. We thank Dr. Jerilyn R. Izac and Dr. Simona Patange for their critical reading, insightful comments, and valuable suggestions.

## Author contributions

Conceptualization, Z.H., J.H.M., L.W., S.L.-G., J.M.Z., and H.-J.H.; methodology, Z.H., J.H.M.., L.T., M.M., N.X., S.L.-G., J.M.Z., and H.-J.H.; investigation, Z.H., J.H.M., L.T., M.M., and N.X.; data curation, Z.H., J.H.M., L.T., and J.M.Z.; resources, Z.H., L.W., S.L.-G., J.M.Z., and H.-J.H.; writing – original draft preparation, Z.H., J.H.M., and J.M.Z.; writing – review and editing, Z.H., J.H.M., L.T., M.M., N.X., L.W., S.L.-G., J.M.Z., and H.-J.H.; visualization, Z.H., J.H.M., L.T., and N.X.

## Declaration of interests

Certain commercial equipment, instruments, and materials are identified to specify the experimental procedure. In no case does such identification imply recommendation or endorsement by the National Institute of Standards and Technology, nor does it imply that the materials or equipment are necessarily the best available for the purpose.

## Supplemental information

Document S1. Figures S1 and S2 and Tables S1–S3

Document S2. Article plus supplemental information
