# Supplementary material for: Long-read genome sequencing reveals complex variability in lentiviral provirus insertions in clonal CD19 CAR-T VCN reference cell lines
Source: Mol Ther Adv. 2026 Oct 21;34(4):201873. doi: 10.1016/j.omta.2026.201873 (PMC13769313; doi:10.1016/j.omta.2026.201873)
Supplement: Supplementary file 1 [file mmc1.pdf]

OMTA, Volume *34*

Supplemental information

Long-read genome sequencing reveals complex variability in lentiviral provirus insertions in clonal CD19 CAR-T VCN reference cell lines

Zhiyong He, Jennifer H. McDaniel, Linhua Tian, Mahir Mohiuddin, Ningchun Xu, Lili Wang, Sheng Lin-Gibson, Justin M. Zook, and Hua-Jun He

Supplemental Text

Observed integrated sequence for all integrations sites (IS) and expected proviral sequence.

```
>IS1_VCN1-VCN2-VCN3-VCN4-VCN5_chr17:78730345
GAAACTGCTAGAGATTTTCCACACTGACTAAAAGGGTCTGAGGGATCTCTAGTTACCAGAGTCACACAACAGACGGGCA
CACACTACTTGAAGCACTCAAGGCAAGCTTTATTGAGGCTTAAGCAGTGGGTTCCCTAGTTAGCCAGAGAGCTCCCAGG
CTCAGATCTGGTCTAACCAGAGAGACCCAGTACAAGCAAAAAGCAGATCTTATCTTCGTTGGGAGTGAATTAGCCCTTC
CAGTCCCCCCTTTTCTTTTAAAAAGTGGCTAAGATCTACAGCTGCCTTGTAAGTCATTGGTCTTAAATTATCAGTCCTG
CTCCTCTGCCACAAAGTGCACGCAGTTGCCGGCCGGGTCGCGCAGGGCGAACTCCCGCCCCCACGGCTGCTCGCCGATC
TCGGTCATGGCCGGCCCGGAGGCGTCCCGGAAGTTCGTGGACACGACCTCCGACCACTCGGCGTACAGCTCGTCCAGGC
CGCGCACCCACACCCAGGCCAGGGTGTTGTCCGGCACCACCTGGTCCTGGACCGCGCTGATGAACAGGGTCACGTCGTC
CCGGACCACACCGGCGAAGTCGTCCTCCACGAAGTCCCGGGAGAACCCGAGCCGGTCGGTCCAGAACTCGACCGCTCCG
GCGACGTCGCGCGCGGTGAGCACCGGAACGGCACTGGTCAACTTGGCCATAGGACCGGGGTTTTCTTCCACGTCTCCTG
CTTGCTTTAACAGAGAGAAGTTCGTGGCTCCAGAACCCTTGTACAGCTCGTCCATGCCGAGAGTGATCCCGGCGGCGGT
CACGAACTCCAGCAGGACCATGTGATCGCGCTTCTCGTTGGGGTCTTTGCTCAGGGCGGACTGGGTGCTCAGGTAGTGG
TTGTCGGGCAGCAGCACGGGGCCGTCGCCGATGGGGGTGTTCTGCTGGTAGTGGTCGGCGAGCTGCACGCTGCCGTCCT
CGATGTTGTGGCGGATCTTGAAGTTCACCTTGATGCCGTTCTTCTGCTTGTCGGCCATGATATAGACGTTGTGGCTGTT
GTAGTTGTACTCCAGCTTGTGCCCCAGGATGTTGCCGTCCTCCTTGAAGTCGATGCCCTTCAGCTCGATGCGGTTCACC
AGGGTGTCGCCCTCGAACTTCACCTCGGCGCGGGTCTTGTAGTTGCCGTCGTCCTTGAAGAAGATGGTGCGCTCCTGGA
CGTAGCCTTCGGGCATGGCGGACTTGAAGAAGTCGTGCTGCTTCATGTGGTCGGGGTAGCGGCTGAAGCACTGCACGCC
GTAGGTCAGGGTGGTCACGAGGGTGGGCCAGGGCACGGGCAGCTTGCCGGTGGTGCAGATGAACTTCAGGGTCAGCTTG
CCGTAGGTGGCATCGCCCTCGCCCTCGCCGGACACGCTGAACTTGTGGCCGTTTACGTCGCCGTCCAGCTCGACCAGGA
TGGGCACCACCCCGGTGAACAGCTCCTCGCCCTTGCTCACCATGGTGGCAAGCTTGGGCTGCAGGTCGAAAGGCCCGGA
GATGAGGAAGAGGAGAACAGCGCGGCAGACGTGCGCTTTTGAAGCGTGCAGAATGCCGGGCCTCCGGAGGACCTTCGGG
CGCCCGCCCCGCCCCTGAGCCCGCCCCTGAGCCCGCCCCCGGACCCACCCCTTCCCAGCCTCTGAGCCCAGAAAGCGAA
GGAGCAAAGCTGCTATTGGCCGCTGCCCCAAAGGCCTACCCGCTTCCATTGCTCAGCGGTGCTGTCCATCTGCACGAGA
CTAGTGAGACGTGCTACTTCCATTTGTCACGTCCTGCACGACGCGAGCTGCGGGGCGGGGGGGAACTTCCTGACTAGGG
GAGGAGTAGAAGGTGGCGCGAAGGGGCCACCAAAGAACGGAGCCGGTTGGCGCCTACCGGTGGATGTGGAATGTGTGCG
AGGCCAGAGGCCACTTGTGTAGCGCCAAGTGCCCAGCGGGGCTGCTAAAGCGCATGCTCCAGACTGCCTTGGGAAAAGC
GCCTCCCCTACCCGGTAGAATTGGTACCAGGCGGGGAGGCGGCCCAAAGGGAGATCCGACTCGTCTGAGGGCGAAGGCG
AAGACGCGGAAGAGGCCGCAGAGCCGGCAGCAGGCCGCGGGAAGGAAGGTCCGCTGGATTGAGGGCCGAAGGGACGTAG
CAGAAGGACGTCCCGCGCAGAATCCAGGTGGCAACACAGGCGAGCAGCCAAGGAAAGGACGATGATTTCCCCGACAACA
CCACGGAATTGTCAGTGCCCAACAGCCGAGCCCCTGTCCAGCAGCGGGCAAGGCAGGCGGCGATGAGTTCCGCCGTGGC
AATAGGGAGGGGGAAAGCGAAAGTCCCGGAAAGGAGCTGACAGGTGGTGGCAATGCCCCAACCAGTGGGGGTTGCGTCA
GCAAACACAGTGCACACCACGCCACGTTGCCTGACAACGGGCCACAACTCCTCATAAAGAGACAGCAACCAGGATTTAT
ACAAGGAGGAGAAAATGAAAGCCATACGGGAAGCAATAGCATGATACAAAGGCATTAAAGCAGCGTATCCACATAGCGT
AAAAGGAGCAACATAGTTAAGAATACCAGTCAATCTTTCACAAATTTTGTAATCCAGAGGTTGATTTCTAGATTATTAG
CGAGGGGGCAGGGCCTGCATGTGAAGGGCGTCGTAGGTGTCCTTGGTGGCTGTACTGAGACCCTGGTAAAGGCCATCGT
GCCCCTTGCCCCTCCGGCGCTCGCCTTTCATCCCAATCTCACTGTAGGCCTCCGCCATCTTATCTTTCTGCAGTTCATT
GTACAGGCCTTCCTGAGGGTTCTTCCTTCTCGGCTTTCCCCCATCTCAGGGTCCCGGCCACGTCTCTTGTCCAAAACA
TCGTACTCCTCTCTTCGTCCTAGATTGAGCTCGTTATAGAGCTGGTTCTGGCCCTGCTTGTACGCGGGGGCGTCTGCGC
TCCTGCTGAACTTCACTCTCAGTTCACATCCTCCTTCTTCTTCTTCTGGAAATCGGCAGCTACAGCCATCTTCCTCTTG
AGTAGTTTGTACTGGTCTCATAAATGGTTGTTTGAATATATACAGGAGTTTCTTTCTGCCCCGTTTGCTCCGGTAGGCG
GCGAAATCCCTGGGAGGGGCGTAGGGCTGGTAGTGCTTCCGGGTGGGGCCAGGCCTCCGGGGGGTCATGTTCATGTAGT
CGCTGTGCAGCAGTCTGCTCCGCTTGCTCCTCACCCAAAAGATGATGAAGGCCACTGTCACCAGCAGGCTGTAGCAGGC
CAGCACGCCGCCCACAACGACCAGCACCCAGAAATCACAGGCGAAGTCCAGCCCCCTCGTGTGCACTGCGCCCCCCGCC
GCTGGCCGGCACGCTTCTGGGCGCAGGGACAGGGGCTGCGACGCGATGGTGGGCGCCGGTGTTGGTGGTCGCGGCGCTG
GCGTCGTGGTAGAAGAAACGGTCACGCTGGTGCCCTGGCCCCAATAATCCATGGCGTAGCTGCCGCCGTAGTAGTAGTG
CTTGGCGCAGTAGTAGATGGCGGTGTCGTCGGTCTGCAGGCTGTTCATCTTCAGGAACACCTGGCTCTTGCTGTTGTCC
TTGATGATGGTCAGCCGGGACTTCAGGGCGCTGTTGTAGTAGGTTGTCTCGCTGCCCCAGATCACTCCCAGCCATTCCA
```

```
GGCCTTTCCGAGGAGGCTGTCTGATCCAGGACACGCCGTAATCAGGCAGGGACACTCCGCTGACTGTACAGGTCACGCT
CAGAGACTGAGATGGGGCCACCAGTCCAGGGCCAGACTCTTGCAGCTTCACTTCAGAACCGCCTCCGCCACTTCCTCCG
CCACCAGATCCTCCGCCTCCGGTGATTTCCAGCTTGGTGCCTCCGCCAAAGGTGTAAGGCAGGGTGTTGCCTTGCTGGC
AGAAGTAGGTAGCGATATCCTCTTGTTCCAGGTTGCTGATTGTCAGGCTGTAGTCGGTGCCAGAGCCGCTGCCAGAAAA
TCTGCTTGGCACGCCGCTGTGCAGTCTGCTGGTGTGGTAGATCAGCAGCTTCACGGTGCCGTCGGGTTTCTGCTGATAC
CAGTTCAGGTACTTGCTGATGTCCTGGCTGGCTCTACAGCTGATGGTCACTCTATCGCCCAGGCTGGCAGACAGGCTGC
TTGTGGTCTGGGTCATCTGGATGTCGGGCCGAGCGGCGTGGAGCAGAAGAGCCAGCGGAAGCAGCAGGGCGGTGACAGG
GAGGGCCATGGTGGCGGCTTCGAATCACGACACCTGAAATGGAAGAAAAAAACTTTGAACCACTGTCTGAGGCTTGAGA
ATGAACCAAGATCCAAACTCAAAAAGGGCAAATTCCAAGGAGAATTACATCAAGTGCCAAGCTGGCCTAACTTCAGTCT
CCACCCACTCAGTGTGGGGAAACTCCATCGCATAAAACCCCTCCCCCCAACCTAAAGACGACGTACTCCAAAAGCTCGA
GAACTAATCGAGGTGCCTGGACGGCGCCCGGTACTCCGTGGAGTCACATGAAGCGACGGCTGAGGACGGAAAGGCCCTT
TTCCTTTGTGTGGGTGACTCACCCGCCCGCTCTCCCGAGCGCCGCGTCCTCCATTTTGAGCTCCCTGCAGCAGGGCCGG
GAAGCGGCCATCTTTCCGCTCACGCAACTGGTGCCGACCGGGCCAGCCTTGCCGCCCAGGGCGGGGCGATACACGGCGG
CGCGAGGCCAGGCACCAGAGCAGGCCGGCCAGCTTGAGACTACCCCCGTCCGATTCTCGGTGGCCGCGCTCGCAGGCCT
CTGGGTTCTACGTTAGTGGGAGTTTAACGACGGTCCCTGGGATTCCCCAAGGCAGGGGCGAGTCCTTTTGTATGAATTA
CTCCATGGTAATAGCGATGACTAATACGTAGATGTACTGCCAAGTAGGAAAGTCCCATAAGGTCATGTACTGGGCATAA
TACTAGTATCGATAAAATTTTGAATTTTGTAATTTGTTTTTGTAATTCTTTAGTTTGTATGTCTGTTGCTATTATGTCT
ACTATTCTTTCCCCTGCACTGTACCCCCCAATCCCCCCTTTTCTTTTAAAAGTTAACCGATACCGTCGAGATCCGTTCA
CTAATCGAATGGATCTGTCTCTGTCTCTCTCTCCACCTTCTTCTTCTATTCCTTCGGGCCTGTCGGGTCCCCTCGGGGT
TGGGAGGTGGGTCTGAAACGATAATGGTGAATATCCCTGCCTAACTCTATTCACTATAGAAAGTACAGCAAAAACTATT
CTTAAACCTACCAAGCCTCCTACTATCATTATGAATAATTTTATATACCACAGCCAATTTGTTATGTTAAACCAATTCC
ACAAACTTGCCCATTTATCTAATTCCAATAATTCTTGTTCATTCTTTTCTTGCTGGTTTTGCGATTCTTCAATTAAGGA
GTGTATTAAGCTTGTGTAATTGTTAATTTCTCTGTCCCACTCCATCCAGGTCGTGTGATTCCAAATCTGTTCCAGAGAT
TTATTACTCCAACTAGCATTCCAAGGCACAGCAGTGGTGCAAATGAGTTTTCCAGAGCAACCCCAAATCCCCAGGAGCT
GTTGATCCTTTAGGTATCTTTCCACAGCCAGGATTCTTGCCTGGAGCTGCTTGATGCCCCAGACTGTGAGTTGCAACAG
ATGCTGTTGCGCCTCAATAGCCCTCAGCAAATTGTTCTGCTGCTGCACTATACCAGACAATAATTGTCTGGCCTGTACC
GTCAGCGTCATTGACGCTGCGCCCATAGTGCTTCCTGCTGCTCCCAAGAACCCAAGGAACAAAGCTCCTATTCCCACTG
CTCTTTTTTCTCTCTGCACCACTCTTCTCTTTGCCTTGGTGGGTGCTACTCCTAATGGTTCAATTTTTACTACTTTATA
TTTATATAATTCACTTCTCCAATTGTCCCTCATATCTCCTCCTCCAGGTCTGAAGATCAGTGGCCGCTTGCTGTGCGGT
GGTCTTACTTTTGTTTTGCTCTTCCTCTATCTTGTCTAAAGCTTCCTTGGTGTCTTTTATCTCTATCCTTTGATGCACA
CAATAGAGGGTTGCTACTGTATTATATAATGATCTAAGTTCTTCTGATCCTGTCTGAAGGGATGGTTGTAGCTGTCCCA
GTATTTGTCTACAGCCTTCTGATGTTTCTAACAGGCCAGGATTAACTGCGAATCGTTCTAGCTCCCTGCTTGCCCATAC
TATATGTTTTAATTTATATTTTTTCTTTCCCCCTGGCCTTAACCGAATTTTTTCCCATCGCGATCTAATTCTCCCCCGC
TTAATACTGACGCTCTCGCACCCATCTCTCTCCTTCTAGCCTCCGCTAGTCAAAATTTTTGGCGTACTCACCAGTCGCC
GCCCCTCGCCTCTTGCCGTGCGCGCTTCAGCAAGCCGAGTCCTGCGTCGAGAGAGCTCCTCTGGTTTCCCTTTCGCTTT
CAAGTCCCTGTTCGGGCGCCACTGCTAGAGATTTTCCACACTGACTAAAAGGGTCTGAGGGATCTCTAGTTACCAGAGT
CACACAACAGACGGGCACACACTACTTGAAGCACTCAAGGCAAGCTTTATTGAGGCTTAAGCAGTGGGTTCCCTAGTTA
GCCAGAGAGCTCCCAGGCTCAGATCTGGTCTAACCAGAGAGACCCAGTACAAGCAAAAAGCAGATCTTATCTTCGTTGG
GAGTGAATTAGCCCTTCCA
>IS2_VCN2_chr19:21610872
CCCCCTGCTAGAGATTTTCCACACTGACTAAAAGGGTCTGAGGGATCTCTAGTTACCAGAGTCACACAACAGACGGGCA
CACACTACTTGAAGCACTCAAGGCAAGCTTTATTGAGGCTTAAGCAGTGGGTTCCCTAGTTAGCCAGAGAGCTCCCAGG
CTCAGATCTGGTCTAACCAGAGAGACCCAGTACAAGCAAAAAGCAGATCTTATCTTCGTTGGGAGTGAATTAGCCCTTC
CAGTCCCCCCTTTTCTTTTAAAAAGTGGCTAAGATCTACAGCTGCCTTGTAAGTCATTGGTCTTAAATTATCAGTCCTG
CTCCTCTGCCACAAAGTGCACGCAGTTGCCGGCCGGGTCGCGCAGGGCGAACTCCCGCCCCCACGGCTGCTCGCCGATC
TCGGTCATGGCCGGCCCGGAGGCGTCCCGGAAGTTCGTGGACACGACCTCCGACCACTCGGCGTACAGCTCGTCCAGGC
CGCGCACCCACACCCAGGCCAGGGTGTTGTCCGGCACCACCTGGTCCTGGACCGCGCTGATGAACAGGGTCACGTCGTC
CCGGACCACACCGGCGAAGTCGTCCTCCACGAAGTCCCGGGAGAACCCGAGCCGGTCGGTCCAGAACTCGACCGCTCCG
GCGACGTCGCGCGCGGTGAGCACCGGAACGGCACTGGTCAACTTGGCCATAGGACCGGGGTTTTCTTCCACGTCTCCTG
CTTGCTTTAACAGAGAGAAGTTCGTGGCTCCAGAACCCTTGTACAGCTCGTCCATGCCGAGAGTGATTCCGGCGGCGGT
CACGAACTCCAGCAGGACCATGTGATCGCGCTTCTCGTTGGGGTCTTTGCTCAGGGCGGACTGGGTGCTCAGGTAGTGG
```

TTGTCGGGCAGCAGCACGGGGCCGTCGCCGATGGGGGTGTTCTGCTGGTAGTGGTCGGCGAGCTGCACGCTGCCGTCCT
CGATGTTGTGGCGGATCTTGAAGTTCACCTTGATGCCGTTCTTCTGCTTGTCGGCCATGATATAGACGTTGTGGCTGTT
GTAGTTGTACTCCAGCTTGTGCCCCAGGATGTTGCCGTCCTCCTTGAAGTCGATGCCCTTCAGCTCGATGCGGTTCACC
AGGGTGTCGCCCTCGAACTTCACCTCGGCGCGGGTCTTGTAGTTGCCGTCGTCCTTGAAGAAGATGGTGCGCTCCTGGA
CGTAGCCTTCGGGCATGGCGGACTTGAAGAAGTCGTGCTGCTTCATGTGGTCGGGGTAGCGGCTGAAGCACTGCACGCC
GTAGGTCAGGGTGGTCACGAGGGTGGGCCAGGGCACGGGCAGCTTGCCGGTGGTGCAGATGAACTTCAGGGTCAGCTTG
CCGTAGGTGGCATCGCCCTCGCCCTCGCCGGACACGCTGAACTTGTGGCCGTTTACGTCGCCGTCCAGCTCGACCAGGA
TGGGCACCACCCCGGTGAACAGCTCCTCGCCCTTGCTCACCATGGTGGCAAGCTTGGGCTGCAGGTCGAAAGGCCCGGA
GATGAGGAAGAGGAGAACAGCGCGGCAGACGTGCGCTTTTGAAGCGTGCAGAATGCCGGGCCTCCGGAGGACCTTCGGG
CGCCCGCCCCGCCCCTGAGCCCGCCCCTGAGCCCGCCCCCGGACCCACCCCTTCCCAGCCTCTGAGCCCAGAAAGCGAA
GGAGCAAAGCTGCTATTGGCCGCTGCCCCAAAGGCCTACCCGCTTCCATTGCTCAGCGGTGCTGTCCATCTGCACGAGA
CTAGTGAGACGTGCTACTTCCATTTGTCACGTCCTGCACGACGCGAGCTGCGGGGCGGGGGGGAACTTCCTGACTAGGG
GAGGAGTAGAAGGTGGCGCGAAGGGGCCACCAAAGAACGGAGCCGGTTGGCGCCTACCGGTGGATGTGGAATGTGTGCG
AGGCCAGAGGCCACTTGTGTAGCGCCAAGTGCCCAGCGGGGCTGCTAAAGCGCATGCTCCAGACTGCCTTGGGAAAAGC
GCCTCCCCTACCCGGTAGAATTGGTACCAGGCGGGGAGGCGGCCCAAAGGGAGATCCGACTCGTCTGAGGGCGAAGGCG
AAGACGCGGAAGAGGCCGCAGAGCCGGCAGCAGGCCGCGGGAAGGAAGGTCCGCTGGATTGAGGGCCGAAGGGACGTAG
CAGAAGGACGTCCCGCGCAGAATCCAGGTGGCAACACAGGCGAGCAGCCAAGGAAAGGACGATGATTTCCCCGACAACA
CCACGGAATTGTCAGTGCCCAACAGCCGAGCCCCTGTCCAGCAGCGGGCAAGGCAGGCGGCGATGAGTTCCGCCGTGGC
AATAGGGAGGGGGAAAGCGAAAGTCCCGGAAAGGAGCTGACAGGTGGTGGCAATGCCCCAACCAGTGGGGGTTGCGTCA
GCAAACACAGTGCACACCACGCCACGTTGCCTGACAACGGGCCACAACTCCTCATAAAGAGACAGCAACCAGGATTTAT
ACAAGGAGGAGAAAATGAAAGCCATACGGGAAGCAATAGCATGATACAAAGGGATTAAAGCAGCGTATCCACATAGCGT
AAAAGGAGCAACATAGTTAAGAATACCAGTCAATCTTTCACAAATTTTGTAATCCAGAGGTTGATTTCTAGATTATTAG
CGAGGGGGCAGGGCCTGCATGTGAAGGGCGTCGTAGGTGTCCTTGGTGGCTGTACTGAGACCCTGGTAAAGGCCATCGT
GCCCCTTGCCCCTCCGGCGCTCGCCTTTCATCCCAATCTCACTGTAGGCCTCCGCCATCTTATCTTTCTGCAGTTCATT
GTACAGGCCTTCCTGAGGGTTCTTCCTTCTCGGCTTTCCCCCCATCTCAGGGTCCCGGCCACGTCTCTTGTCCAAAACA
TCGTACTCCTCTCTTCGTCCTAGATTGAGCTCGTTATAGAGCTGGTTCTGGCCCTGCTTGTACGCGGGGGCGTCTGCGC
TCCTGCTGAACTTCACTCTCAGTTCACATCCTCCTTCTTCTTCTTCTGGAAATCGGCAGCTACAGCCATCTTCCTCTTG
AGTAGTTTGTACTGGTCTCATAAATGGTTGTTTGAATATATACAGGAGTTTCTTTCTGCCCCGTTTGCTCCGGTAGGCG
GCGAAATCCCTGGGAGGGGCGTAGGGCTGGTAGTGCTTCCGGGTGGGGCCAGGCCTCCGGGGGGTCATGTTCATGTAGT
CGCTGTGCAGCAGTCTGCTCCGCTTGCTCCTCACCCAAAAGATGATGAAGGCCACTGTCACCAGCAGGCTGTAGCAGGC
CAGCACGCCGCCCACAACGACCAGCACCCAGAAATCACAGGCGAAGTCCAGCCCCCTCGTGTGCACTGCGCCCCCCGCC
GCTGGCCGGCACGCTTCTGGGCGCAGGGACAGGGGCTGCGACGCGATGGTGGGCGCCGGTGTTGGTGGTCGCGGCGCTG
GCGTCGTGGTAGAAGAAACGGTCACGCTGGTGCCCTGGCCCCAATAATCCATGGCGTAGCTGCCGCCGTAGTAGTAGTG
CTTGGCGCAGTAGTAGATGGCGGTGTCGTCGGTCTGCAGGCTGTTCATCTTCAGGAACACCTGGCTCTTGCTGTTGTCC
TTGATGATGGTCAGCCGGGACTTCAGGGCGCTGTTGTAGTAGGTTGTCTCGCTGCCCCAGATCACTCCCAGCCATTCCA
GGCCTTTCCGAGGAGGCTGTCTGATCCAGGACACGCCGTAATCAGGCAGGGACACTCCGCTGACTGTACAGGTCACGCT
CAGAGACTGAGATGGGGCCACCAGTCCAGGGCCAGACTCTTGCAGCTTCACTTCAGAACCGCCTCCGCCACTTCCTCCG
CCACCAGATCCTCCGCCTCCGGTGATTTCCAGCTTGGTGCCTCCGCCAAAGGTGTAAGGCAGGGTGTTGCCTTGCTGGC
AGAAGTAGGTAGCGATATCCTCTTGTTCCAGGTTGCTGATTGTCAGGCTGTAGTCGGTGCCAGAGCCGCTGCCAGAAAA
TCTGCTTGGCACGCCGCTGTGCAGTCTGCTGGTGTGGTAGATCAGCAGCTTCACGGTGCCGTCGGGTTTCTGCTGATAC
CAGTTCAGGTACTTGCTGATGTCCTGGCTGGCTCTACAGCTGATGGTCACTCTATCGCCCAGGCTGGCAGACAGGCTGC
TTGTGGTCTGGGTCATCTGGATGTCGGGCCGAGCGGCGTGGAGCAGAAGAGCCAGCGGAAGCAGCAGGGCGGTGACAGG
GAGGGCCATGGTGGCGGCTTCGAATCACGACACCTGAAATGGAAGAAAAAAACTTTGAACCACTGTCTGAGGCTTGAGA
ATGAACCAAGATCCAAACTCAAAAAGGGCAAATTCCAAGGAGAATTACATCAAGTGCCAAGCTGGCCTAACTTCAGTCT
CCACCCACTCAGTGTGGGGAAACTCCATCGCATAAAACCCCTCCCCCCAACCTAAAGACGACGTACTCCAAAAGCTCGA
GAACTAATCGAGGTGCCTGGACGGCGCCCGGTACTCCGTGGAGTCACATGAAGCGACGGCTGAGGACGGAAAGGCCCTT
TTCCTTTGTGTGGGTGACTCACCCGCCCGCTCTCCCGAGCGCCGCGTCCTCCATTTTGAGCTCCCTGCAGCAGGGCCGG
GAAGCGGCCATCTTTCCGCTCACGCAACTGGTGCCGACCGGGCCAGCCTTGCCGCCCAGGGCGGGGCGATACACGGCGG
CGCGAGGCCAGGCACCAGAGCAGGCCGGCCAGCTTGAGACTACCCCCGTCCGATTCTCGGTGGCCGCGCTCGCAGGCCC
CGCCTCGCCGAACATGTGCGCTGGGACGCACGGGCCCCGTCGCCGCCCGCGGCCCCAAAAACCGAAATACCAGTGTGCA
GATCTTGGCCCGCATTTACAAGACTATCTTGCCAGAAAAAAAGCGTCGCAGCAGGTCATCAAAAATTTTAAATGGCTAG

AGACTTATCGAAAGCAGCGAGACAGGCGCGAAGGTGCCACCAGATTCGCACGCGGCGGCCCCAGCGCCCAAGCCAGGCC
TCAACTCAAGCACGAGGCGAAGGGGCTCCTTAAGCGCAAGGCCTCGAACTCTCCCACCCACTTCCAACCCGAAGCTCGG
GATCAAGAATCACGTACTGCAGCCAGGGGCGTGGAAGTAATTCAAGGCACGCAAGGGCCATAACCCGTAAAGAGGCCAG
GCCCGCGGGAACCACACACGGCACTTACCTGTGTTCTGGCGGCAAACCCGTTGCGAAAAAGAACGTTCACGGCGACTAC
TGCACTTATATACGGTTCTCCCCCACCCTCGGGAAAAAGGCGGAGCCAGTACACGACATCACTTTCCCAGTTTACCCCG
CGCCACCTTCTCTAGGCACCGGTTCAATTGCCGACCCCTCCCCCCAACTTCTCGGGGACTGTGGGCGATGTGCGCTCTG
CCCACTGACGGGCACCGGAGCCTCACGCATGCTCTTCTCCACCTCAGTGATGACGAGAGCGGGCGGGTGAGGGGGCGGG
AACGCAGCGATCTCTGGGTTCTACGTTAGTGGGAGTTTAACGACGGTCCCTGGGATTCCCCAAGGCAGGGGCGAGTCCT
TTTGTATGAATTACTCCATGGTAATAGCGATGACTAATACGTAGATGTACTGCCAAGTAGGAAAGTCCCATAAGGTCAT
GTACTGGGCATAATACTAGTATCGATAAAATTTTGAATTTTGTAATTTGTTTTTGTAATTCTTTAGTTTGTATGTCTAA
TGCTATTATGTCTACTATTCTTTCCCCTGCACTGTACCCCCCAATCCCCCCTTTTCTTTTAAAAGTTAACCGATACCGT
CGAGATCCGTTCACTAATCGAATGGATCTGTCTCTGTCTCTCTCTCCACCTTCTTCTTCTATTCCTTCGGGCCTGTCGG
GTCCCCTCGGGGTTGGGAGGTGGGTCTGAAACGATAATGGTGAATATCCCTGCCTAACTCTATTCACTATAGAAAGTAC
AGCAAAAACTATTCTTAAACCTACCAAGCCTCCTACTATCATTATGAATAATTTTATATACCACAGCCAATTTGTTATG
TTAAACCAATTCCACAAACTTGCCCATTTATCTAATTCCAATAATTCTTGTTCATTCTTTTCTTGCTGGTTTTGCGATT
CTTCAATTAAGGAGTGTATTAAGCTTGTGTAATTGTTAATTTCTCTGTCCCACTCCATCCAGGTCGTGTGATTCCAAAT
CTGTTCCAGAGATTTATTACTCCAACTAGCATTCCAAGGCACAGCAGTGGTGCAAATGAGTTTTCCAGAGCAACCCCAA
ATCCCCAGGAGCTGTTGATCCTTTAGGTATCTTTCCACAGCCAGGATTCTTGCCTGGAGCTGCTTGATGCCCCAGACTG
TGAGTTGCAACAGATGCTGTTGCGCCTCAATAGCCCTCAGCAAATTGTTCTGCTGCTGCACTATACCAGACAATAATTG
TCTGGCCTGTACCGTCAGCGTCATTGACGCTGCGCCCATAGTGCTTCCTGCTGCTCCCAAGAACCCAAGGAACAAAGCT
CCTATTCCCACTGCTCTTTTTTCTCTCTGCACCACTCTTCTCTTTGCCTTGGTGGGTGCTACTCCTAATGGTTCAATTT
TTACTACTTTATATTTATATAATTCACTTCTCCAATTGTCCCTCATATCTCCTCCTCCAGGTCTGAAGATCAGTGGCCG
CTTGCTGTGCGGTGGTCTTACTTTTGTTTTGCTCTTCCTCTATCTTGTCTAAAGCTTCCTTGGTGTCTTTTATCTCTAT
CCTTTGATGCACACAATAGAGGGTTGCTACTGTATTATATAATGATCTAAGTTCTTCTGATCCTGTCTGAAGGGATGGT
TGTAGCTGTCCCAGTATTTGTCTACAGCCTTCTGATGTTTCTAACAGGCCAGGATTAACTGCGAATCGTTCTAGCTCCC
TGCTTGCCCATACTATATGTTTTAATTTATATTTTTTCTTTCCCCCTGGCCTTAACCGAATTTTTTCCCATCGCGATCT
AATTCTCCCCCGCTTAATACTGACGCTCTCGCACCCATCTCTCTCCTTCTAGCCTCCGCTAGTCAAAATTTTTGGCGTA
CTCACCAGTCGCCGCCCCTCGCCTCTTGCCGTGCGCGCTTCAGCAAGCCGAGTCCTGCGTCGAGAGAGCTCCTCTGGTT
TCCCTTTCGCTTTCAAGTCCCTGTTCGGGCGCCACTGCTAGAGATTTTCCACACTGACTAAAAGGGTCTGAGGGATCTC
TAGTTACCAGAGTCACACAACAGACGGGCACACACTACTTGAAGCACTCAAGGCAAGCTTTATTGAGGCTTAAGCAGTG
GGTTCCCTAGTTAGCCAGAGAGCTCCCAGGCTCAGATCTGGTCTAACCAGAGAGACCCAGTACAAGCAAAAAGCAGATC
TTATCTTCGTTGGGAGTGAATTAGCCCTTCCA
>IS3_VCN3_chr1:23307863
ACAGGTGCTAGAGATTTTCCACACTGACTAAAAGGGTCTGAGGGATCTCTAGTTACCAGAGTCACACAACAGACGGGCA
CACACTACTTGAAGCACTCAAGGCAAGCTTTATTGAGGCTTAAGCAGTGGGTTCCCTAGTTAGCCAGAGAGCTCCCAGG
CTCAGATCTGGTCTAACCAGAGAGACCCAGTACAAGCAAAAAGCAGATCTTATCTTCGTTGGGAGTGAATTAGCCCTTC
CAGTCCCCCCTTTTCTTTTAAAAAGTGGCTAAGATCTACAGCTGCCTTGTAAGTCATTGGTCTTAAATTATCAGTCCTG
CTCCTCTGCCACAAAGTGCACGCAGTTGCCGGCCGGGTCGCGCAGGGCGAACTCCCGCCCCCACGGCTGCTCGCCGATC
TCGGTCATGGCCGGCCCGGAGGCGTCCCGGAAGTTCGTGGACACGACCTCCGACCACTCGGCGTACAGCTCGTCCAGGC
CGCGCACCCACACCCAGGCCAGGGTGTTGTCCGGCACCACCTGGTCCTGGACCGCGCTGATGAACAGGGTCACGTCGTC
CCGGACCACACCGGCGAAGTCGTCCTCCACGAAGTCCCGGGAGAACCCGAGCCGGTCGGTCCAGAACTCGACCGCTCCG
GCGACGTCGCGCGCGGTGAGCACCGGAACGGCACTGGTCAACTTGGCCATAGGACCGGGGTTTTCTTCCACGTCTCCTG
CTTGCTTTAACAGAGAGAAGTTCGTGGCTCCAGAACCCTTGTACAGCTCGTCCATGCCGAGAGTGATCCCGGCGGCGGT
CACGAACTCCAGCAGGACCATGTGATCGCGCTTCTCGTTGGGGTCTTTGCTCAGGGCGGACTGGGTGCTCAGGTAGTGG
TTGTCGGGCAGCAGCACGGGGCCGTCGCCGATGGGGGTGTTCTGCTGGTAGTGGTCGGCGAGCTGCACGCTGCCGTCCT
CGATGTTGTGGCGGATCTTGAAGTTCACCTTGATGCCGTTCTTCTGCTTGTCGGCCATGATATAGACGTTGTGGCTGTT
GTAGTTGTACTCCAGCTTGTGCCCCAGGATGTTGCCGTCCTCCTTGAAGTCGATGCCCTTCAGCTCGATGCGGTTCACC
AGGGTGTCGCCCTCGAACTTCACCTCGGCGCGGGTCTTGTAGTTGCCGTCGTCCTTGAAGAAGATGGTGCGCTCCTGGA
CGTAGCCTTCGGGCATGGCGGACTTGAAGAAGTCGTGCTGCTTCATGTGGTCGGGGTAGCGGCTGAAGCACTGCACGCC
GTAGGTCAGGGTGGTCACGAGGGTGGGCCAGGGCACGGGCAGCTTGCCGGTGGTGCAGATGAACTTCAGGGTCAGCTTG
CCGTAGGTGGCATCGCCCTCGCCCTCGCCGGACACGCTGAACTTGTGGCCGTTTACGTCGCCGTCCAGCTCGACCAGGA

TGGGCACCACCCCGGTGAACAGCTCCTCGCCCTTGCTCACCATGGTGGCAAGCTTGGGCTGCAGGTCGAAAGGCCCGGA
GATGAGGAAGAGGAGAACAGCGCGGCAGACGTGCGCTTTTGAAGCGTGCAGAATGCCGGGCCTCCGGAGGACCTTCGGG
CGCCCGCCCCGCCCCTGAGCCCGCCCCTGAGCCCGCCCCCGGACCCACCCCTTCCCAGCCTCTGAGCCCAGAAAGCGAA
GGAGCAAAGCTGCTATTGGCCGCTGCCCCAAAGGCCTACCCGCTTCCATTGCTCAGCGGTGCTGTCCATCTGCACGAGA
CTAGTGAGACGTGCTACTTCCATTTGTCACGTCCTGCACGACGCGAGCTGCGGGGCGGGGGGGAACTTCCTGACTAGGG
GAGGAGTAGAAGGTGGCGCGAAGGGGCCACCAAAGAACGGAGCCGGTTGGCGCCTACCGGTGGATGTGGAATGTGTGCG
AGGCCAGAGGCCACTTGTGTAGCGCCAAGTGCCCAGCGGGGCTGCTAAAGCGCATGCTCCAGACTGCCTTGGGAAAAGC
GCCTCCCCTACCCGGTAGAATTGGTACCAGGCGGGGAGGCGGCCCAAAGGGAGATCCGACTCGTCTGAGGGCGAAGGCG
AAGACGCGGAAGAGGCCGCAGAGCCGGCAGCAGGCCGCGGGAAGGAAGGTCCGCTGGATTGAGGGCCGAAGGGACGTAG
CAGAAGGACGTCCCGCGCAGAATCCAGGTGGCAACACAGGCGAGCAGCCAAGGAAAGGACGATGATTTCCCCGACAACA
CCACGGAATTGTCAGTGCCCAACAGCCGAGCCCCTGTCCAGCAGCGGGCAAGGCAGGCGGCGATGAGTTCCGCCGTGGC
AATAGGGAGGGGGAAAGCGAAAGTCCCGGAAAGGAGCTGACAGGTGGTGGCAATGCCCCAACCAGTGGGGGTTGCGTCA
GCAAACACAGTGCACACCACGCCACGTTGCCTGACAACGGGCCACAACTCCTCATAAAGAGACAGCAACCAGGATTTAT
ACAAGGAGGAGAAAATGAAAGCCATACGGGAAGCAATAGCATGATACAAAGGCATTAAAGCAGCGTATCCACATAGCGT
AAAAGGAGCAACATAGTTAAGAATACCAGTCAATCTTTCACAAATTTTGTAATCCAGAGGTTGATTTCTAGATTATTAG
CGAGGGGGCAGGGCCTGCATGTGAAGGGCGTCGTAGGTGTCCTTGGTGGCTGTACTGAGACCCTGGTAAAGGCCATCGT
GCCCCTTGCCCCTCCGGCGCTCGCCTTTCATCCCAATCTCACTGTAGGCCTCCGCCATCTTATCTTTCTGCAGTTCATT
GTACAGGCCTTCCTGAGGGTTCTTCCTTCTCGGCTTTCCCCCCATCTCAGGGTCCCGGCCACGTCTCTTGTCCAAAACA
TCGTACTCCTCTCTTCGTCCTAGATTGAGCTCGTTATAGAGCTGGTTCTGGCCCTGCTTGTACGCGGGGGCGTCTGCGC
TCCTGCTGAACTTCACTCTCAGTTCACATCCTCCTTCTTCTTCTTCTGGAAATCGGCAGCTACAGCCATCTTCCTCTTG
AGTAGTTTGTACTGGTCTCATAAATGGTTGTTTGAATATATACAGGAGTTTCTTTCTGCCCCGTTTGCTCCGGTAGGCG
GCGAAATCCCTGGGAGGGGCGTAGGGCTGGTAGTGCTTCCGGGTGGGGCCAGGCCTCCGGGGGGTCATGTTCATGTAGT
CGCTGTGCAGCAGTCTGCTCCGCTTGCTCCTCACCCAAAAGATGATGAAGGCCACTGTCACCAGCAGGCTGTAGCAGGC
CAGCACGCCGCCCACAACGACCAGCACCCAGAAATCACAGGCGAAGTCCAGCCCCCTCGTGTGCACTGCGCCCCCCGCC
GCTGGCCGGCACGCTTCTGGGCGCAGGGACAGGGGCTGCGACGCGATGGTGGGCGCCGGTGTTGGTGGTCGCGGCGCTG
GCGTCGTGGTAGAAGAAACGGTCACGCTGGTGCCCTGGCCCCAATAATCCATGGCGTAGCTGCCGCCGTAGTAGTAGTG
CTTGGCGCAGTAGTAGATGGCGGTGTCGTCGGTCTGCAGGCTGTTCATCTTCAGGAACACCTGGCTCTTGCTGTTGTCC
TTGATGATGGTCAGCCGGGACTTCAGGGCGCTGTTGTAGTAGGTTGTCTCGCTGCCCCAGATCACTCCCAGCCATTCCA
GGCCTTTCCGAGGAGGCTGTCTGATCCAGGACACGCCGTAATCAGGCAGGGACACTCCGCTGACTGTACAGGTCACGCT
CAGAGACTGAGATGGGGCCACCAGTCCAGGGCCAGACTCTTGCAGCTTCACTTCAGAACCGCCTCCGCCACTTCCTCCG
CCACCAGATCCTCCGCCTCCGGTGATTTCCAGCTTGGTGCCTCCGCCAAAGGTGTAAGGCAGGGTGTTGCCTTGCTGGC
AGAAGTAGGTAGCGATATCCTCTTGTTCCAGGTTGCTGATTGTCAGGCTGTAGTCGGTGCCAGAGCCGCTGCCAGAAAA
TCTGCTTGGCACGCCGCTGTGCAGTCTGCTGGTGTGGTAGATCAGCAGCTTCACGGTGCCGTCGGGTTTCTGCTGATAC
CAGTTCAGGTACTTGCTGATGTCCTGGCTGGCTCTACAGCTGATGGTCACTCTATCGCCCAGGCTGGCAGACAGGCTGC
TTGTGGTCTGGGTCATCTGGATGTCGGGCCGAGCGGCGTGGAGCAGAAGAGCCAGCGGAAGCAGCAGGGCGGTGACAGG
GAGGGCCATGGTGGCGGCTTCGAATCACGACACCTGAAATGGAAGAAAAAAACTTTGAACCACTGTCTGAGGCTTGAGA
ATGAACCAAGATCCAAACTCAAAAAGGGCAAATTCCAAGGAGAATTACATCAAGTGCCAAGCTGGCCTAACTTCAGTCT
CCACCCACTCAGTGTGGGGAAACTCCATCGCATAAAACCCCTCCCCCCAACCTAAAGACGACGTACTCCAAAAGCTCGA
GAACTAATCGAGGTGCCTGGACGGCGCCCGGTACTCCGTGGAGTCACATGAAGCGACGGCTGAGGACGGAAAGGCCCTT
TTCCTTTGTGTGGGTGACTCACCCGCCCGCTCTCCCGAGCGCCGCGTCCTCCATTTTGAGCTCCCTGCAGCAGGGCCGG
GAAGCGGCCATCTTTCCGCTCACGCAACTGGTGCCGACCGGGCCAGCCTTGCCGCCCAGGGCGGGGCGATACACGGCGG
CGCGAGGCCAGGCACCAGAGCAGGCCGGCCAGCTTGAGACTACCCCCGTCCGATTCTCGGTGGCCGCGCTCGCAGGCCC
CGCCTCGCCGAACATGTGCGCTGGGACGCACGGGCCCCGTCGCCGCCCGCGGCCCCAAAAACCGAAATACCAGTGTGCA
GATCTTGGCCCGCATTTACAAGACTATCTTGCCAGAAAAAAAGCGTCGCAGCAGGTCATCAAAAATTTTAAATGGCTAG
AGACTTATCGAAAGCAGCGAGACAGGCGCGAAGGTGCCACCAGATTCGCACGCGGCGGCCCCAGCGCCCAAGCCAGGCC
TCAACTCAAGCACGAGGCGAAGGGGCTCCTTAAGCGCAAGGCCTCGAACTCTCCCACCCACTTCCAACCCGAAGCTCGG
GATCAAGAATCACGTACTGCAGCCAGGGGCGTGGAAGTAATTCAAGGCACGCAAGGGCCATAACCCGTAAAGAGGCCAG
GCCCGCGGGAACCACACACGGCACTTACCTGTGTTCTGGCGGCAAACCCGTTGCGAAAAAGAACGTTCACGGCGACTAC
TGCACTTATATACGGTTCTCCCCCACCCTCGGGAAAAAGGCGGAGCCAGTACACGACATCACTTTCCCAGTTTACCCCG
CGCCACCTTCTCTAGGCACCGGTTCAATTGCCGACCCCTCCCCCCAACTTCTCGGGGACTGTGGGCGATGTGCGCTCTG
CCCACTGACGGGCACCGGAGCCTCACGCATGCTCTTCTCCACCTCAGTGATGACGAGAGCGGGCGGGTGAGGGGGCGGG

AACGCAGCGATCTCTGGGTTCTACGTTAGTGGGAGTTTAACGACGGTCCCTGGGATTCCCCAAGGCAGGGGCGAGTCCT
TTTGTATGAATTACTCCATGGTAATAGCGATGACTAATACGTAGATGTACTGCCAAGTAGGAAAGTCCCATAAGGTCAT
GTACTGGGCATAATACTAGTATCGATAAAATTTTGAATTTTGTAATTTGTTTTTGTAATTCTTTAGTTTGTATGTCTGT
TGCTATTATGTCTACTATTCTTTCCCCTGCACTGTACCCCCCAATCCCCCCTTTTCTTTTAAAAGTTAACCGATACCGT
CGAGATCCGTTCACTAATCGAATGGATCTGTCTCTGTCTCTCTCTCCACCTTCTTCTTCTATTCCTTCGGGCCTGTCGG
GTCCCCTCGGGGTTGGGAGGTGGGTCTGAAACGATAATGGTGAATATCCCTGCCTAACTCTATTCACTATAGAAAGTAC
AGCATAAACTATTCTTAAACCTACCAAGCCTCCTACTATCATTATGAATAATTTTATATACCACAGCCAATTTGTTATG
TTAAACCAATTCCACAAACTTGCCCATTTATCTAATTCCAATAATTCTTGTTCATTCTTTTCTTGCTGGTTTTGCGATT
CTTCAATTAAGGAGTGTATTAAGCTTGTGTAATTGTTAATTTCTCTGTCCCACTCCATCCAGGTCGTGTGATTCCAAAT
CTGTTCCAGAGATTTATTACTCCAACTAGCATTCCAAGGCACAGCAGTGGTGCAAATGAGTTTTCCAGAGCAACCCCAA
ATCCCCAGGAGCTGTTGATCCTTTAGGTATCTTTCCACAGCCAGGATTCTTGCCTGGAGCTGCTTGATGCCCCAGACTG
TGAGTTGCAACAGATGCTGTTGCGCCTCAATAGCCCTCAGCAAATTGTTCTGCTGCTGCACTATACCAGACAATAATTG
TCTGGCCTGTACCGTCAGCGTCATTGACGCTGCGCCCATAGTGCTTCCTGCTGCTCCCAAGAACCCAAGGAACAAAGCT
CCTATTCCCACTGCTCTTTTTTCTCTCTGCACCACTCTTCTCTTTGCCTTGGTGGGTGCTACTCCTAATGGTTCAATTT
TTACTACTTTATATTTATATAATTCACTTCTCCAATTGTCCCTCATATCTCCTCCTCCAGGTCTGAAGATCAGTGGCCG
CTTGCTGTGCGGTGGTCTTACTTTTGTTTTGCTCTTCCTCTATCTTGTCTAAAGCTTCCTTGGTGTCTTTTATCTCTAT
CCTTTGATGCACACAATAGAGGGTTGCTACTGTATTATATAATGATCTAAGTTCTTCTGATCCTGTCTGAAGGGATGGT
TGTAGCTGTCCCAGTATTTGTCTACAGCCTTCTGATGTTTCTAACAGGCCAGGATTAACTGCGAATCGTTCTAGCTCCC
TGCTTGCCCATACTATATGTTTTAATTTATATTTTTTCTTTCCCCCTGGCCTTAACCGAATTTTTTCCCATCGCGATCT
AATTCTCCCCCGCTTAATACTGACGCTCTCGCACCCATCTCTCTCCTTCTAGCCTCCGCTAGTCAAAATTTTTGGCGTA
CTCACCAGTCGCCGCCCCTCGCCTCTTGCCGTGCGCGCTTCAGCAAGCCGAGTCCTGCGTCGAGAGAGCTCCTCTGGTT
TCCCTTTCGCTTTCAAGTCCCTGTTCGGGCGCCACTGCTAGAGATTTTCCACACTGACTAAAAGGGTCTGAGGGATCTC
TAGTTACCAGAGTCACACAACAGACGGGCACACACTACTTGAAGCACTCAAGGCAAGCTTTATTGAGGCTTAAGCAGTG
GGTTCCCTAGTTAGCCAGAGAGCTCCCAGGCTCAGATCTGGTCTAACCAGAGAGACCCAGTACAAGCAAAAAGCAGATC
TTATCTTCGTTGGGAGTGAATTAGCCCTTCCAGTAAAGGACGATGATTTCCCCGACAACACCACGGAATTGTCAGTGCC
CAACAGCCGAGCCCCTGTCCAGCAGCGGGCAAGGCAGGCGGCGATGAGTTCCGCCGTGGCAATAGGGAGGGGGAAAGCG
AAAGTCCCGGAAAGGAGCTGACAGGTGGTGGCAATGCCCCAACCAGTGGGGGTTGCGTCAGCAAACACAGTGCACACCA
CGCCACGTTGCCTGACAACGGGCCACAACTCCTCATAAAGAGACAGCAACCAGGATTTATACAAGGAGGAGAAAATGAA
AGCCATACGGGAAGCAATAGCATGATACAAAGGCATTAAAGCAGCGTATCCACATAGCGTAAAAGGAGCAACATAGTTA
AGAATACCAGTCAATCTTTCACAAATTTTGTAATCCAGAGGTTGATTTCTAGATTATTAGCGAGGGGGCAGGGCCTGCA
TGTGAAGGGCGTCGTAGGTGTCCTTGGTGGCTGTACTGAGACCCTGGTAAAGGCCATCGTGCCCCTTGCCCCTCCGGCG
CTCGCCTTTCATCCCAATCTCACTGTAGGCCTCCGCCATCTTATCTTTCTGCAGTTCATTGTACAGGCCTTCCTGAGGG
TTCTTCCTTCTCGGCTTTCCCCCCATCTCAGGGTCCCGGCCACGTCTCTTGTCCAAAACATCGTACTCCTCTCTTCGTC
CTAGATTGAGCTCGTTATAGAGCTGGTTCTGGCCCTGCTTGTACGCGGGGGCGTCTGCGCTCCTGCTGAACTTCACTCT
CAGTTCACATCCTCCTTCTTCTAGTCAGTGTGGAAAATCTCTAGCA
>IS4_VCN3_chr8:99830407
AAACTGCTAGAGATTTTCCACACTGACTAAAAGGGTCTGAGGGATCTCTAGTTACCAGAGTCACACAACAGACGGGCAC
ACACTACTTGAAGCACTCAAGGCAAGCTTTATTGAGGCTTAAGCAGTGGGTTCCCTAGTTAGCCAGAGAGCTCCCAGGC
TCAGAGAGCTCCAGAGAGAGCTCCCAGGCTCCCAGGCTCAGAGAGCTCCCAGGCTCAGAGAGCTCCCAGGCCCAGAGAG
CTCCCAGCCAGAGAGCTCCCAGGCTC
>IS5_VCN3_chrX:103930456
AATACTGCTAGAGATTTTCCACACTGACTAAAAGGGTCTGAGGGATCTCTAGTTACCAGAGTCACACAACAGACGGGCA
CACACTACTTGAAGCACTCAAGGCAAGCTTTATTGAGGCTTAAGCAGTGGGTTCCCTAGTTAGCCAGAGAGCTCCCAGG
CTCAGATCTGGTCTAACCAGAGAGACCCAGTACAAGCAAAAAGCAGATCTTATCTTCGTTGGGAGTGAATTAGCCCTTC
CAGTCCCCCCTTTTCTTTTAAAAAGTGGCTAAGATCTACAGCTGCCTTGTAAGTCATTGGTCTTAAATTATCAGTCCTG
CTCCTCTGCCACAAAGTGCACGCAGTTGCCGGCCGGGTCGCGCAGGGCGAACTCCCGCCCCCACGGCTGCTCGCCGATC
TCGGTCATGGCCGGCCCGGAGGCGTCCCGGAAGTTCGTGGACACGACCTCCGACCACTCGGCGTACAGCTCGTCCAGGC
CGCGCACCCACACCCAGGCCAGGGTGTTGTCCGGCACCACCTGGTCCTGGACCGCGCTGATGAACAGGGTCACGTCGTC
CCGGACCACACCGGCGAAGTCGTCCTCCACGAAGTCCCGGGAGAACCCGAGCCGGTCGGTCCAGAACTCGACCGCTCCG
GCGACGTCGCGCGCGGTGAGCACCGGAACGGCACTGGTCAACTTGGCCATAGGACCGGGGTTTTCTTCCACGTCTCCTG
CTTGCTTTAACAGAGAGAAGTTCGTGGCTCCAGAACCCTTGTACAGCTCGTCCATGCCGAGAGTGATCCCGGCGGCGGT

CACGAACTCCAGCAGGACCATGTGATCGCGCTTCTCGTTGGGGTCTTTGCTCAGGGCGGACTGGGTGCTCAGGTAGTGG
TTGTCGGGCAGCAGCACGGGGCCGTCGCCGATGGGGGTGTTCTGCTGGTAGTGGTCGGCGAGCTGCACGCTGCCGTCCT
CGATGTTGTGGCGGATCTTGAAGTTCACCTTGATGCCGTTCTTCTGCTTGTCGGCCATGATATAGACGTTGTGGCTGTT
GTAGTTGTACTCCAGCTTGTGCCCCAGGATGTTGCCGTCCTCCTTGAAGTCGATGCCCTTCAGCTCGATGCGGTTCACC
AGGGTGTCGCCCTCGAACTTCACCTCGGCGCGGGTCTTGTAGTTGCCGTCGTCCTTGAAGAAGATGGTGCGCTCCTGGA
CGTAGCCTTCGGGCATGGCGGACTTGAAGAAGTCGTGCTGCTTCATGTGGTCGGGGTAGCGGCTGAAGCACTGCACGCC
GTAGGTCAGGGTGGTCACGAGGGTGGGCCAGGGCACGGGCAGCTTGCCGGTGGTGCAGATGAACTTCAGGGTCAGCTTG
CCGTAGGTGGCATCGCCCTCGCCCTCGCCGGACACGCTGAACTTGTGGCCGTTTACGTCGCCGTCCAGCTCGACCAGGA
TGGGCACCACCCCGGTGAACAGCTCCTCGCCCTTGCTCACCATGGTGGCAAGCTTGGGCTGCAGGTCGAAAGGCCCGGA
GATGAGGAAGAGGAGAACAGCGCGGCAGACGTGCGCTTTTGAAGCGTGCAGAATGCCGGGCCTCCGGAGGACCTTCGGG
CGCCCGCCCCGCCCCTGAGCCCGCCCCTGAGCCCGCCCCCGGACCCACCCCTTCCCAGCCTCTGAGCCCAGAAAGCGAA
GGAGCAAAGCTGCTATTGGCCGCTGCCCCAAAGGCCTACCCGCTTCCATTGCTCAGCGGTGCTGTCCATCTGCACGAGA
CTAGTGAGACGTGCTACTTCCATTTGTCACGTCCTGCACGACGCGAGCTGCGGGGCGGGGGGAACTTCCTGACTAGGG
GAGGAGTAGAAGGTGGCGCGAAGGGGCCACCAAAGAACGGAGCCGGTTGGCGCCTACCGGTGGATGTGGAATGTGTGCG
AGGCCAGAGGCCACTTGTGTAGCGCCAAGTGCCCAGCGGGGCTGCTAAAGCGCATGCTCCAGACTGCCTTGGGAAAAGC
GCCTCCCCTACCCGGTAGAATTGGTACCAGGCGGGGAGGCGGCCCAAAGGGAGATCCGACTCGTCTGAGGGCGAAGGCG
AAGACGCGGAAGAGGCCGCAGAGCCGGCAGCAGGCCGCGGGAAGGAAGGTCCGCTGGATTGAGGGCCGAAGGGACGTAG
CAGAAGGACGTCCCGCGCAGAATCCAGGTGGCAACACAGGCGAGCAGCCAAGGAAAGGACGATGATTTCCCCGACAACA
CCACGGAATTGTCAGTGCCCAACAGCCGAGCCCCTGTCCAGCAGCGGGCAAGGCAGGCGGCGATGAGTTCCGCCGTGGC
AATAGGGAGGGGGAAAGCGAAAGTCCCGGAAAGGAGCTGACAGGTGGTGGCAATGCCCCAACCAGTGGGGGTTGCGTCA
GCAAACACAGTGCACACCACGCCACGTTGCCTGACAACGGGCCACAACTCCTCATAAAGAGACAGCAACCAGGATTTAT
ACAAGGAGGAGAAAATGAAAGCCATACGGGAAGCAATAGCATGATACAAAGGCATTAAAGCAGCGTATCCACATAGCGT
AAAAGGAGCAACATAGTTAAGAATACCAGTCAATCTTTCACAAATTTTGTAATCCAGAGGTTGATTTCTAGATTATTAG
CGAGGGGGCAGGGCCTGCATGTGAAGGGCGTCGTAGGTGTCCTTGGTGGCTGTACTGAGACCCTGGTAAAGGCCATCGT
GCCCCTTGCCCCTCCGGCGCTCGCCTTTCATCCCAATCTCACTGTAGGCCTCCGCCATCTTATCTTTCTGCAGTTCATT
GTACAGGCCTTCCTGAGGGTTCTTCCTTCTCGGCTTTCCCCCCATCTCAGGGTCCCGGCCACGTCTCTTGTCCAAAACA
TCGTACTCCTCTCTTCGTCCTAGATTGAGCTCGTTATAGAGCTGGTTCTGGCCCTGCTTGTACGCGGGGGCGTCTGCGC
TCCTGCTGAACTTCACTCTCAGTTCACATCCTCCTTCTTCTTCTTCTGGAAATCGGCAGCTACAGCCATCTTCCTCTTG
AGTAGTTTGTACTGGTCTCATAAATGGTTGTTTGAATATATACAGGAGTTTCTTTCTGCCCCGTTTGCTCCGGTAGGCG
GCGAAATCCCTGGGAGGGGCGTAGGGCTGGTAGTGCTTCCGGGTGGGGCCAGGCCTCCGGGGGGTCATGTTCATGTAGT
CGCTGTGCAGCAGTCTGCTCCGCTTGCTCCTCACCCAAAAGATGATGAAGGCCACTGTCACCAGCAGGCTGTAGCAGGC
CAGCACGCCGCCCACAACGACCAGCACCCAGAAATCACAGGCGAAGTCCAGCCCCCTCGTGTGCACTGCGCCCCCCGCC
GCTGGCCGGCACGCTTCTGGGCGCAGGGACAGGGGCTGCGACGCGATGGTGGGCGCCGGTGTTGGTGGTCGCGGCGCTG
GCGTCGTGGTAGAAGAAACGGTCACGCTGGTGCCCTGGCCCCAATAATCCATGGCGTAGCTGCCGCCGTAGTAGTAGTG
CTTGGCGCAGTAGTAGATGGCGGTGTCGTCGGTCTGCAGGCTGTTCATCTTCAGGAACACCTGGCTCTTGCTGTTGTCC
TTGATGATGGTCAGCCGGGACTTCAGGGCGCTGTTGTAGTAGGTTGTCTCGCTGCCCCAGATCACTCCCAGCCATTCCA
GGCCTTTCCGAGGAGGCTGTCTGATCCAGGACACGCCGTAATCAGGCAGGGACACTCCGCTGACTGTACAGGTCACGCT
CAGAGACTGAGATGGGGCCACCAGTCCAGGGCCAGACTCTTGCAGCTTCACTTCAGAACCGCCTCCGCCACTTCCTCCG
CCACCAGATCCTCCGCCTCCGGTGATTTCCAGCTTGGTGCCTCCGCCAAAGGTGTAAGGCAGGGTGTTGCCTTGCTGGC
AGAAGTAGGTAGCGATATCCTCTTGTTCCAGGTTGCTGATTGTCAGGCTGTAGTCGGTGCCAGAGCCGCTGCCAGAAAA
TCTGCTTGGCACGCCGCTGTGCAGTCTGCTGGTGTGGTAGATCAGCAGCTTCACGGTGCCGTCGGGTTTCTGCTGATAC
CAGTTCAGGTACTTGCTGATGTCCTGGCTGGCTCTACAGCTGATGGTCACTCTATCGCCCAGGCTGGCAGACAGGCTGC
TTGTGGTCTGGGTCATCTGGATGTCGGGCCGAGCGGCGTGGAGCAGAAGAGCCAGCGGAAGCAGCAGGGCGGTGACAGG
GAGGGCCATGGTGGCGGCTTCGAATCACGACACCTGAAATGGAAGAAAAAAACTTTGAACCACTGTCTGAGGCTTGAGA
ATGAACCAAGATCCAAACTCAAAAAGGGCAAATTCCAAGGAGAATTACATCAAGTGCCAAGCTGGCCTAACTTCAGTCT
CCACCCACTCAGTGTGGGGAAACTCCATCGCATAAAACCCCTCCCCCCAACCTAAAGACGACGTACTCCAAAAGCTCGA
GAACTAATCGAGGTGCCTGGACGGCGCCCGGTACTCCGTGGAGTCACATGAAGCGACGGCTGAGGACGGAAAGGCCCTT
TTCCTTTGTGTGGGTGACTCACCCGCCCGCTCTCCCGAGCGCCGCGTCCTCCATTTTGAGCTCCCTGCAGCAGGGCCGG
GAAGCGGCCATCTTTCCGCTCACGCAACTGGTGCCGACCGGGCCAGCCTTGCCGCCCAGGGCGGGGCGATACACGGCGG
CGCGAGGCCAGGCACCAGAGCAGGCCGGCCAGCTTGAGACTACCCCCGTCCGATTCTCGGTGGCCGCGCTCGCAGGCCC
CGCCTCGCCGAACATGTGCGCTGGGACGCACGGGCCCCGTCGCCGCCCGCGGCCCCAAAAACCGAAATACCAGTGTGCA

GATCTTGGCCCGCATTTACAAGACTATCTTGCCAGAAAAAAAGCGTCGCAGCAGGTCATCAAAAATTTTAAATGGCTAG
AGACTTATCGAAAGCAGCGAGACAGGCGCGAAGGTGCCACCAGATTCGCACGCGGCGGCCCCAGCGCCCAAGCCAGGCC
TCAACTCAAGCACGAGGCGAAGGGGCTCCTTAAGCGCAAGGCCTCGAACTCTCCCACCCACTTCCAACCCGAAGCTCGG
GATCAAGAATCACGTACTGCAGCCAGGGGCGTGGAAGTAATTCAAGGCACGCAAGGGCCATAACCCGTAAAGAGGCCAG
GCCCGCGGGAACCACACACGGCACTTACCTGTGTTCTGGCGGCAAACCCGTTGCGAAAAAGAACGTTCACGGCGACTAC
TGCACTTATATACGGTTCTCCCCCACCCTCGGGAAAAAGGCGGAGCCAGTACACGACATCACTTTCCCAGTTTACCCCG
CGCCACCTTCTCTAGGCACCGGTTCAATTGCCGACCCCTCCCCCCAACTTCTCGGGGACTGTGGGCGATGTGCGCTCTG
CCCACTGACGGGCACCGGAGCCTCACGCATGCTCTTCTCCACCTCAGTGATGACGAGAGCGGGCGGGTGAGGGGGCGGG
AACGCAGCGATCTCTGGGTTCTACGTTAGTGGGAGTTTAACGACGGTCCCTGGGATTCCCCAAGGCAGGGGCGAGTCCT
TTTGTATGAATTACTCCATGGTAATAGCGATGACTAATACGTAGATGTACTGCCAAGTAGGAAAGTCCCATAAGGTCAT
GTACTGGGCATAATACTAGTATCGATAAAATTTTGAATTTTGTAATTTGTTTTTGTAATTCTTTAGTTTGTATGTCTGT
TGCTATTATGTCTACTATTCTTTCCCCTGCACTGTACCCCCCAATCCCCCCTTTTCTTTTAAAAGTTAACCGATACCGT
CGAGATCCGTTCACTAATCGAATGGATCTGTCTCTGTCTCTCTCTCCACCTTCTTCTTCTATTCCTTCGGGCCTGTCGG
GTCCCCTCGGGGTTGGGAGGTGGGTCTGAAACGATAATGGTGAATATCCCTGCCTAACTCTATTCACTATAGAAAGTAC
AGCAAAAACTATTCTTAAACCTACCAAGCCTCCTACTATCATTATGAATAATTTTATATACCACAGCCAATTTGTTATG
TTAAACCAATTCCACAAACTTGCCCATTTATCTAATTCCAATAATTCTTGTTCATTCTTTTCTTGCTGGTTTTGCGATT
CTTCAATTAAGGAGTGTATTAAGCTTGTGTAATTGTTAATTTCTCTGTCCCACTCCATCCAGGTCGTGTGATTCCAAAT
CTGTTCCAGAGATTTATTACTCCAACTAGCATTCCAAGGCACAGCAGTGGTGCAAATGAGTTTTCCAGAGCAACCCCAA
ATCCCCAGGAGCTGTTGATCCTTTAGGTATCTTTCCACAGCCAGGATTCTTGCCTGGAGCTGCTTGATGCCCCAGACTG
TGAGTTGCAACAGATGCTGTTGCGCCTCAATAGCCCTCAGCAAATTGTTCTGCTGCTGCACTATACCAGACAATAATTG
TCTGGCCTGTACCGTCAGCGTCATTGACGCTGCGCCCATAGTGCTTCCTGCTGCTCCCAAGAACCCAAGGAACAAAGCT
CCTATTCCCACTGCTCTTTTTTCTCTCTGCACCACTCTTCTCTTTGCCTTGGTGGGTGCTACTCCTAATGGTTCAATTT
TTACTACTTTATATTTATATAATTCACTTCTCCAATTGTCCCTCATATCTCCTCCTCCAGGTCTGAAGATCAGTGGCCG
CTTGCTGTGCGGTGGTCTTACTTTTGTTTTGCTCTTCCTCTATCTTGTCTAAAGCTTCCTTGGTGTCTTTTATCTCTAT
CCTTTGATGCACACAATAGAGGGTTGCTACTGTATTATATAATGATCTAAGTTCTTCTGATCCTGTCTGAAGGGATGGT
TGTAGCTGTCCCAGTATTTGTCTACAGCCTTCTGATGTTTCTAACAGGCCAGGATTAACTGCGAATCGTTCTAGCTCCC
TGCTTGCCCATACTATATGTTTTAATTTATATTTTTTCTTTCCCCCTGGCCTTAACCGAATTTTTTCCCATCGCGATCT
AATTCTCCCCCGCTTAATACTGACGCTCTCGCACCCATCTCTCTCCTTCTAGCCTCCGCTAGTCAAAATTTTTGGCGTA
CTCACCAGTCGCCGCCCCTCGCCTCTTGCCGTGCGCGCTTCAGCAAGCCGAGTCCTGCGTCGAGAGAGCTCCTCTGGTT
TCCCTTTCGCTTTCAAGTCCCTGTTCGGGCGCCACTGCTAGAGATTTTCCACACTGACTAAAAGGGTCTGAGGGATCTC
TAGTTACCAGAGTCACACAACAGACGGGCACACACTACTTGAAGCACTCAAGGCAAGCTTTATTGAGGCTTAAGCAGTG
GGTTCCCTAGTTAGCCAGAGAGCTCCCAGGCTCAGATCTGGTCTAACCAGAGAGACCCAGTACAAGCAAAAAGCAGATC
TTATCTTCGTTGGGAGTGAATTAGCCCTTCCA
>IS6_VCN4-VCN5_chr1:243273502
ACCCCTGGAAGGGCTAATTCACTCCCAACGAAGATAAGATCTGCTTTTTGCTTGTACTGGGTCTCTCTGGTTAGACCAG
ATCTGAGCCTGGGAGCTCTCTGGCTAACTAGGGAACCCACTGCTTAAGCCTCAATAAAGCTTGCCTTGAGTGCTTCAAG
TAGTGTGTGCCCGTCTGTTGTGTGACTCTGGTAACTAGAGATCCCTCAGACCCTTTTAGTCAGTGTGGAAAATCTCTAG
CAGTGGCGCCCGAACAGGGACTTGAAAGCGAAAGGGAAACCAGAGGAGCTCTCTCGACGCAGGACTCGGCTTGCTGAAG
CGCGCACGGCAAGAGGCGAGGGGCGGCGACTGGTGAGTACGCCAAAAATTTTGACTAGCGGAGGCTAGAAGGAGAGAGA
TGGGTGCGAGAGCGTCAGTATTAAGCGGGGGAGAATTAGATCGCGATGGGAAAAAATTCGGTTAAGGCCAGGGGGAAAG
AAAAAATATAAATTAAAACATATAGTATGGGCAAGCAGGGAGCTAGAACGATTCGCAGTTAATCCTGGCCTGTTAGAAA
CATCAGAAGGCTGTAGACAAATACTGGGACAGCTACAACCATCCCTTCAGACAGGATCAGAAGAACTTAGATCATTATA
TAATACAGTAGCAACCCTCTATTGTGTGCATCAAAGGATAGAGATAAAAGACACCAAGGAAGCTTTAGACAAGATAGAG
GAAGAGCAAAACAAAAGTAAGACCACCGCACAGCAAGCGGCCACTGATCTTCAGACCTGGAGGAGGAGATATGAGGGAC
AATTGGAGAAGTGAATTATATAAATATAAAGTAGTAAAAATTGAACCATTAGGAGTAGCACCCACCAAGGCAAAGAGAA
GAGTGGTGCAGAGAGAAAAAAGAGCAGTGGGAATAGGAGCTTTGTTCCTTGGGTTCTTGGGAGCAGCAGGAAGCACTAT
GGGCGCAGCGTCAATGACGCTGACGGTACAGGCCAGACAATTATTGTCTGGTATAGTGCAGCAGCAGAACAATTTGCTG
AGGGCTATTGAGGCGCAACAGCATCTGTTGCAACTCACAGTCTAGGGCATCAAGCAGCTCCAGGCAAGAATCCTGGCTG
TGGAAAGATACCTAAAGGATCAACAGCTCCTGGGGATTTGGGGTTGCTCTGGAAAACTCATTTGCACCACTGCTGTGCC
TTGGAATGCTAGTTGGAGTAATAAATCTCTGGAACAGATTTGGAATCACACGACCTGGATGGAGTGGGACAGAGAAATT
AACAATTACACAAGCTTAATACACTCCTTAATTGAAGAATCGCAAAACCAGCAAGAAAAGAATGAACAAGAATTATTGG

AATTAGATAAATGGGCAAGTTTGTGGAATTGGTTTAACATAACAAATTGGCTGTGGTATATAAAATTATTCATAATGAT
AGTAGGAGGCTTGGTAGGTTTAAGAATAGTTTTTGCTGTACTTTCTATAGTGAATAGAGTTAGGCAGGGATATTCACCA
TTATCGTTTCAGACCCACCTCCCAACCCCGAGGGGACCCGACAGGCCCGAAGGAATAGAAGAAGAAGGTGGAGAGAGAG
ACAGAGACAGATCCATTCGATTAGTGAACGGATCTCGACGGTATCGGTTAACTTTTAAAAGAAAAGGGGGGATTGGGGG
GTACAGTGCAGGGGAAAGAATAGTAGACATAATAGCAACAGACATACAAACTAAAGAATTACAAAAACAAATTACAAAA
TTCAAAATTTTATCGATACTAGTATTATGCCCAGTACATGACCTTATGGGACTTTCCTACTTGGCAGTACATCTACGTA
TTAGTCATCGCTATTACCATGGAGTAATTCATACAAAAGGACTCGCCCCTGCCTTGGGGAATCCCAGGGACCGTCGTTA
AACTCCCACTAACGTAGAACCCAGAGATCGCTGCGTTCCCGCCCCCTCACCCGCCCGCTCTCGTCATCACTGAGGTGGA
GAAGAGCATGCGTGAGGCTCCGGTGCCCGTCAGTGGGCAGAGCGCACATCGCCCACAGTCCCCGAGAAGTTGGGGGGAG
GGGTCGGCAATTGAACCGGTGCCTAGAGAAGGTGGCGCGGGGTAAACTGGGAAAGTGATGTCGTGTACTGGCTCCGCCT
TTTTCCCGAGGGTGGGGGAGAACCGTATATAAGTGCAGTAGTCGCCGTGAACGTTCTTTTTCGCAACGGGTTTGCCGCC
AGAACACAGGTAAGTGCCGTGTGTGGTTCCCGCGGGCCTGGCCTCTTTACGGGTTATGGCCCTTGCGTGCCTTGAATTA
CTTCCACGCCCCTGGCTGCAGTACGTGATTCTTGATCCCGAGCTTCGGGTTGGAAGTGGGTGGGAGAGTTCGAGGCCTT
GCGCTTAAGGAGCCCCTTCGCCTCGTGCTTGAGTTGAGGCCTGGCTTGGGCGCTGGGGCCGCCGCGTGCGAATCTGGTG
GCACCTTCGCGCCTGTCTCGCTGCTTTCGATAAGTCTCTAGCCATTTAAAATTTTTGATGACCTGCTGCGACGCTTTTT
TTCTGGCAAGATAGTCTTGTAAATGCGGGCCAAGATCTGCACACTGGTATTTCGGTTTTTGGGGCCGCGGGCGGCGACG
GGGCCCGTGCGTCCCAGCGCACATGTTCGGCGAGGCGGGGCCTGCGAGCGCGGCCACCGAGAATCGGACGGGGGTAGTC
TCAAGCTGGCCGGCCTGCTCTGGTGCCTGGCCTCGCGCCGCCGTGTATCGCCCCGCCCTGGGCGGCAAGGCTGGCCCGG
TCGGCACCAGTTGCGTGAGCGGAAAGATGGCCGCTTCCCGGCCCTGCTGCAGGGAGCTCAAAATGGAGGACGCGGCGCT
CGGGAGAGCGGGCGGGTGAGTCACCCACACAAAGGAAAAGGGCCTTTCCGTCCTCAGCCGTCGCTTCATGTGACTCCAC
GGAGTACCGGGCGCCGTCCAGGCACCTCGATTAGTTCTCGAGCTTTTGGAGTACGTCGTCTTTAGGTTGGGGGGAGGGG
TTTTATGCGATGGAGTTTCCCCACACTGAGTGGGTGGAGACTGAAGTTAGGCCAGCTTGGCACTTGATGTAATTCTCCT
TGGAATTTGCCCTTTTTGAGTTTGGATCTTGGTTCATTCTCAAGCCTCAGACAGTGGTTCAAAGTTTTTTTCTTCCATT
TCAGGTGTCGTGATTCGAAGCCGCCACCATGGCCCTCCCTGTCACCGCCCTGCTGCTTCCGCTGGCTCTTCTGCTCCAC
GCCGCTCGGCCCGACATCCAGATGACCCAGACCACAAGCAGCCTGTCTGCCAGCCTGGGCGATAGAGTGACCATCAGCT
GTAGAGCCAGCCAGGACATCAGCAAGTACCTGAACTGGTATCAGCAGAAACCCGACGGCACCGTGAAGCTGCTGATCTA
CCACACCAGCAGACTGCACAGCGGCGTGCCAAGCAGATTTTCTGGCAGCGGCTCTGGCACCGACTACAGCCTGACAATC
AGCAACCTGGAACAAGAGGATATCGCTACCTACTTCTGCCAGCAAGGCAACACCCTGCCTTACACCTTTGGCGGAGGCA
CCAAGCTGGAAATCACCGGAGGCGGAGGATCTGGTGGCGGAGGAAGTGGCGGAGGCGGTTCTGAAGTGAAGCTGCAAGA
GTCTGGCCCTGGACTGGTGGCCCCATCTCAGTCTCTGAGCGTGACCTGTACAGTCAGCGGAGTGTCCCTGCCTGATTAC
GGCGTGTCCTGGATCAGACAGCCTCCTCGGAAAGGCCTGGAATGGCTGGGAGTGATCTGGGGCAGCGAGACAACCTACT
ACAACAGCGCCCTGAAGTCCCGGCTGACCATCATCAAGGACAACAGCAAGAGCCAGGTGTTCCTGAAGATGAACAGCCT
GCAGACCGACGACACCGCCATCTACTACTGCGCCAAGCACTACTACTACGGCGGCAGCTACGCCATGGATTATTGGGGC
CAGGGCACCAGCGTGACCGTTTCTTCTACCACGACGCCAGCGCCGCGACCACCAACACCGGCGCCCACCATCGCGTCGC
AGCCCCTGTCCCTGCGCCCAGAAGCGTGCCGGCCAGCGGCGGGGGGCGCAGTGCACACGAGGGGGCTGGACTTCGCCTG
TGATTTCTGGGTGCTGGTCGTTGTGGGCGGCGTGCTGGCCTGCTACAGCCTGCTGGTGACAGTGGCCTTCATCATCTTT
TGGGTGAGGAGCAAGCGGAGCAGACTGCTGCACAGCGACTACATGAACATGACCCCCCGGAGGCCTGGCCCCACCCGGA
AGCACTACCAGCCCTACGCCCCTCCCAGGGATTTCGCCGCCTACCGGAGCAAACGGGGCAGAAAGAAACTCCTGTATAT
ATTCAAACAACCATTTATGAGACCAGTACAAACTACTCAAGAGGAAGATGGCTGTAGCTGCCGATTTCCAGAAGAAGAA
GAAGGAGGATGTGAACTGAGAGTGAAGTTCAGCAGGAGCGCAGACGCCCCCGCGTACAAGCAGGGCCAGAACCAGCTCT
ATAACGAGCTCAATCTAGGACGAAGAGAGGAGTACGATGTTTTGGACAAGAGACGTGGCCGGGACCCTGAGATGGGGGG
AAAGCCGAGAAGGAAGAACCCTCAGGAAGGCCTGTACAATGAACTGCAGAAAGATAAGATGGCGGAGGCCTACAGTGAG
ATTGGGATGAAAGGCGAGCGCCGGAGGGGCAAGGGGCACGATGGCCTTTACCAGGGTCTCAGTACAGCCACCAAGGACA
CCTACGACGCCCTTCACATGCAGGCCCTGCCCCCTCGCTAATAATCTAGAAATCAACCTCTGGATTACAAAATTTGTGA
AAGATTGACTGGTATTCTTAACTATGTTGCTCCTTTTACGCTATGTGGATACGCTGCTTTAATGCCTTTGTATCATGCT
ATTGCTTCCCGTATGGCTTTCATTTTCTCCTCCTTGTATAAATCCTGGTTGCTGTCTCTTTATGAGGAGTTGTGGCCCG
TTGTCAGGCAACGTGGCGTGGTGTGCACTGTGTTTGCTGACGCAACCCCCACTGGTTGGGGCATTGCCACCACCTGTCA
GCTCCTTTCCGGGACTTTCGCTTTCCCCCTCCCTATTGCCACGGCGGAACTCATCGCCGCCTGCCTTGCCCGCTGCTGG
ACAGGGGCTCGGCTGTTGGGCACTGACAATTCCGTGGTGTTGTCGGGGAAATCATCGTCCTTTCCTTGGCTGCTCGCCT
GTGTTGCCACCTGGATTCTGCGCGGGACGTCCTTCTGCTACGTCCCTTCGGCCCTCAATCCAGCGGACCTTCCTTCCCG
CGGCCTGCTGCCGGCTCTGCGGCCTCTTCCGCGTCTTCGCCTTCGCCCTCAGACGAGTCGGATCTCCCTTTGGGCCGCC

TCCCCGCCTGGTACCAATTCTACCGGGTAGGGGAGGCGCTTTTCCCAAGGCAGTCTGGAGCATGCGCTTTAGCAGCCCC
GCTGGGCACTTGGCGCTACACAAGTGGCCTCTGGCCTCGCACACATTCCACATCCACCGGTAGGCGCCAACCGGCTCCG
TTCTTTGGTGGCCCCTTCGCGCCACCTTCTACTCCTCCCCTAGTCAGGAAGTTCCCCCCCGCCCCGCAGCTCGCGTCGT
GCAGGACGTGACAAATGGAAGTAGCACGTCTCACTAGTCTCGTGCAGATGGACAGCACCGCTGAGCAATGGAAGCGGGT
AGGCCTTTGGGGCAGCGGCCAATAGCAGCTTTGCTCCTTCGCTTTCTGGGCTCAGAGGCTGGGAAGGGGTGGGTCCGGG
GGCGGGCTCAGGGGCGGGCTCAGGGGCGGGGCGGGCGCCCGAAGGTCCTCCGGAGGCCCGGCATTCTGCACGCTTCAAA
AGCGCACGTCTGCCGCGCTGTTCTCCTCTTCCTCATCTCCGGGCCTTTCGACCTGCAGCCCAAGCTTGCCACCATGGTG
AGCAAGGGCGAGGAGCTGTTCACCGGGGTGGTGCCCATCCTGGTCGAGCTGGACGGCGACGTAAACGGCCACAAGTTCA
GCGTGTCCGGCGAGGGCGAGGGCGATGCCACCTACGGCAAGCTGACCCTGAAGTTCATCTGCACCACCGGCAAGCTGCC
CGTGCCCTGGCCCACCCTCGTGACCACCCTGACCTACGGCGTGCAGTGCTTCAGCCGCTACCCCGACCACATGAAGCAG
CACGACTTCTTCAAGTCCGCCATGCCCGAAGGCTACGTCCAGGAGCGCACCATCTTCTTCAAGGACGACGGCAACTACA
AGACCCGCGCCGAGGTGAAGTTCGAGGGCGACACCCTGGTGAACCGCATCGAGCTGAAGGGCATCGACTTCAAGGAGGA
CGGCAACATCCTGGGGCACAAGCTGGAGTACAACTACAACAGCCACAACGTCTATATCATGGCCGACAAGCAGAAGAAC
GGCATCAAGGTGAACTTCAAGATCCGCCACAACATCGAGGACGGCAGCGTGCAGCTCGCCGACCACTACCAGCAGAACA
CCCCCATCGGCGACGGCCCCGTGCTGCTGCCCGACAACCACTACCTGAGCACCCAGTCCGCCCTGAGCAAAGACCCCAA
CGAGAAGCGCGATCACATGGTCCTGCTGGAGTTCGTGACCGCCGCCGGGATCACTCTCGGCATGGACGAGCTGTACAAG
GGTTCTGGAGCCACGAACTTCTCTCTGTTAAAGCAAGCAGGAGACGTGGAAGAAAACCCCGGTCCTATGGCCAAGTTGA
CCAGTGCCGTTCCGGTGCTCACCGCGCGCGACGTCGCCGGAGCGGTCGAGTTCTGGACCGACCGGCTCGGGTTCTCCCG
GGACTTCGTGGAGGACGACTTCGCCGGTGTGGTCCGGGACGACGTGACCCTGTTCATCAGCGCGGTCCAGGACCAGGTG
GTGCCGGACAACACCCTGGCCTGGGTGTGGGTGCGCGGCCTGGACGAGCTGTACGCCGAGTGGTCGGAGGTCGTGTCCA
CGAACTTCCGGGACGCCTCCGGGCCGGCCATGACCGAGATCGGCGAGCAGCCGTGGGGGCGGGAGTTCGCCCTGCGCGA
CCCGGCCGGCAACTGCGTGCACTTTGTGGCAGAGGAGCAGGACTGATAATTTAAGACCAATGACTTACAAGGCAGCTGT
AGATCTTAGCCACTTTTTAAAAGAAAAGGGGGGACTGGAAGGGCTAATTCACTCCCAACGAAGATAAGATCTGCTTTTT
GCTTGTACTGGGTCTCTCTGGTTAGACCAGATCTGAGCCTGGGAGCTCTCTGGCTAACTAGGGAACCCACTGCTTAAGC
CTCAATAAAGCTTGCCTTGAGTGCTTCAAGTAGTGTGTGCCCGTCTGTTGTGTGACTCTGGTAACTAGAGATCCCTCAG
ACCCTTTTAGTCAGTGTGGAAAATCTCTAGCAGTAAATCCTGCTACCTCGTTGGGAGTGAATTAGCCCTTCCA
>IS7_VCN4-VCN5_chr3:18115277
TACTCTGCTAGAGATTTTCCACACTGACTAAAAGGGTCTGAGGGATCTCTAGTTACCAGAGTCACACAACAGACGGGCA
CACACTACTTGAAGCACTCAAGGCAAGCTTTATTGAGGCTTAAGCAGTGGGTTCCCTAGTTAGCCAGAGAGCTCCCAGG
CTCAGATCTGGTCTAACCAGAGAGACCCAGTACAAGCAAAAAGCAGATCTTATCTTCGTTGGGAGTGAATTAGCCCTTC
CAGTCCCCCCTTTTCTTTTAAAAAGTGGCTAAGATCTACAGCTGCCTTGTAAGTCATTGGTCTTAAATTATCAGTCCTG
CTCCTCTGCCACAAAGTGCACGCAGTTGCCGGCCGGGTCGCGCAGGGCGAACTCCCGCCCCCACGGCTGCTCGCCGATC
TCGGTCATGGCCGGCCCGGAGGCGTCCCGGAAGTTCGTGGACACGACCTCCGACCACTCGGCGTACAGCTCGTCCAGGC
CGCGCACCCACACCCAGGCCAGGGTGTTGTCCGGCACCACCTGGTCCTGGACCGCGCTGATGAACAGGGTCACGTCGTC
CCGGACCACACCGGCGAAGTCGTCCTCCACGAAGTCCCGGGAGAACCCGAGCCGGTCGGTCCAGAACTCGACCGCTCCG
GCGACGTCGCGCGCGGTGAGCACCGGAACGGCACTGGTCAACTTGGCCATAGGACCGGGGTTTTCTTCCACGTCTCCTG
CTTGCTTTAACAGAGAGAAGTTCGTGGCTCCAGAACCCTTGTACAGCTCGTCCATGCCGAGAGTGATCCCGGCGGCGGT
CACGAACTCCAGCAGGACCATGTGATCGCGCTTCTCGTTGGGGTCTTTGCTCAGGGCGGACTGGGTGCTCAGGTAGTGG
TTGTCGGGCAGCAGCACGGGGCCGTCGCCGATGGGGGTGTTCTGCTGGTAGTGGTCGGCGAGCTGCACGCTGCCGTCCT
CGATGTTGTGGCGGATCTTGAAGTTCACCTTGATGCCGTTCTTCTGCTTGTCGGCCATGATATAGACGTTGTGGCTGTT
GTAGTTGTACTCCAGCTTGTGCCCCAGGATGTTGCCGTCCTCCTTGAAGTCGATGCCCTTCAGCTCGATGCGGTTCACC
AGGGTGTCGCCCTCGAACTTCACCTCGGCGCGGGTCTTGTAGTTGCCGTCGTCCTTGAAGAAGATGGTGCGCTCCTGGA
CGTAGCCTTCGGGCATGGCGGACTTGAAGAAGTCGTGCTGCTTCATGTGGTCGGGGTAGCGGCTGAAGCACTGCACGCC
GTAGGTCAGGGTGGTCACGAGGGTGGGCCAGGGCACGGGCAGCTTGCCGGTGGTGCAGATGAACTTCAGGGTCAGCTTG
CCGTAGGTGGCATCGCCCTCGCCCTCGCCGGACACGCTGAACTTGTGGCCGTTTACGTCGCCGTCCAGCTCGACCAGGA
TGGGCACCACCCCGGTGAACAGCTCCTCGCCCTTGCTCACCATGGTGGCAAGCTTGGGCTGCAGGTCGAAAGGCCCGGA
GATGAGGAAGAGGAGAACAGCGCGGCAGACGTGCGCTTTTGAAGCGTGCAGAATGCCGGGCCTCCGGAGGACCTTCGGG
CGCCCGCCCCGCCCCTGAGCCCGCCCCTGAGCCCGCCCCCGGACCCACCCCTTCCCAGCCTCTGAGCCCAGAAAGCGAA
GGAGCAAAGCTGCTATTGGCCGCTGCCCCAAAGGCCTACCCGCTTCCATTGCTCAGCGGTGCTGTCCATCTGCACGAGA
CTAGTGAGACGTGCTACTTCCATTTGTCACGTCCTGCACGACGCGAGCTGCGGGGCGGGGGGGAACTTCCTGACTAGGG
GAGGAGTAGAAGGTGGCGCGAAGGGGCCACCAAAGAACGGAGCCGGTTGGCGCCTACCGGTGGATGTGGAATGTGTGCG

AGGCCAGAGGCCACTTGTGTAGCGCCAAGTGCCCAGCGGGGCTGCTAAAGCGCATGCTCCAGACTGCCTTGGGAAAAGC
GCCTCCCCTACCCGGTAGAATTGGTACCAGGCGGGGAGGCGGCCCAAAGGGAGATCCGACTCGTCTGAGGGCGAAGGCG
AAGACGCGGAAGAGGCCGCAGAGCCGGCAGCAGGCCGCGGGAAGGAAGGTCCGCTGGATTGAGGGCCGAAGGGACGTAG
CAGAAGGACGTCCCGCGCAGAATCCAGGTGGCAACACAGGCGAGCAGCCAAGGAAAGGACGATGATTTCCCCGACAACA
CCACGGAATTGTCAGTGCCCAACAGCCGAGCCCCTGTCCAGCAGCGGGCAAGGCAGGCGGCGATGAGTTCCGCCGTGGC
AATAGGGAGGGGGAAAGCGAAAGTCCCGGAAAGGAGCTGACAGGTGGTGGCAATGCCCCAACCAGTGGGGGTTGCGTCA
GCAAACACAGTGCACACCACGCCACGTTGCCTGACAACGGGCCACAACTCCTCATAAAGAGACAGCAACCAGGATTTAT
ACAAGGAGGAGAAAATGAAAGCCATACGGGAAGCAATAGCATGATACAAAGGCATTAAAGCAGCGTATCCACATAGCGT
AAAAGGAGCAACATAGTTAAGAATACCAGTCAATCTTTCACAAATTTTGTAATCCAGAGGTTGATTTCTAGATTATTAG
CGAGGGGGCAGGGCCTGCATGTGAAGGGCGTCGTAGGTGTCCTTGGTGGCTGTACTGAGACCCTGGTAAAGGCCATCGT
GCCCCTTGCCCCTCCGGCGCTCGCCTTTCATCCCAATCTCACTGTAGGCCTCCGCCATCTTATCTTTCTGCAGTTCATT
GTACAGGCCTTCCTGAGGGTTCTTCCTTCTCGGCTTTCCCCCCATCTCAGGGTCCCGGCCACGTCTCTTGTCCAAAACA
TCGTACTCCTCTCTTCGTCCTAGATTGAGCTCGTTATAGAGCTGGTTCTGGCCCTGCTTGTACGCGGGGGCGTCTGCGC
TCCTGCTGAACTTCACTCTCAGTTCACATCCTCCTTCTTCTTCTTCTGGAAATCGGCAGCTACAGCCATCTTCCTCTTG
AGTAGTTTGTACTGGTCTCATAAATGGTTGTTTGAATATATACAGGAGTTTCTTTCTGCCCCGTTTGCTCCGGTAGGCG
GCGAAATCCCTGGGAGGGGCGTAGGGCTGGTAGTGCTTCCGGGTGGGGCCAGGCCTCCGGGGGGTCATGTTCATGTAGT
CGCTGTGCAGCAGTCTGCTCCGCTTGCTCCTCACCCAAAAGATGATGAAGGCCACTGTCACCAGCAGGCTGTAGCAGGC
CAGCACGCCGCCCACAACGACCAGCACCCAGAAATCACAGGCGAAGTCCAGCCCCCTCGTGTGCACTGCGCCCCCCGCC
GCTGGCCGGCACGCTTCTGGGCGCAGGGACAGGGGCTGCGACGCGATGGTGGGCGCCGGTGTTGGTGGTCGCGGCGCTG
GCGTCGTGGTAGAAGAAACGGTCACGCTGGTGCCCTGGCCCCAATAATCCATGGCGTAGCTGCCGCCGTAGTAGTAGTG
CTTGGCGCAGTAGTAGATGGCGGTGTCGTCGGTCTGCAGGCTGTTCATCTTCAGGAACACCTGGCTCTTGCTGTTGTCC
TTGATGATGGTCAGCCGGGACTTCAGGGCGCTGTTGTAGTAGGTTGTCTCGCTGCCCCAGATCACTCCCAGCCATTCCA
GGCCTTTCCGAGGAGGCTGTCTGATCCAGGACACGCCGTAATCAGGCAGGGACACTCCGCTGACTGTACAGGTCACGCT
CAGAGACTGAGATGGGGTCACCAGTCCAGGGCCAGACTCTTGCAGCTTCACTTCAGAACCGCCTCCGCCACTTCCTCCG
CCACCAGATCCTCCGCCTCCGGTGATTTCCAGCTTGGTGCCTCCGCCAAAGGTGTAAGGCAGGGTGTTGCCTTGCTGGC
AGAAGTAGGTAGCGATATCCTCTTGTTCCAGGTTGCTGATTGTCAGGCTGTAGTCGGTGCCAGAGCCGCTGCCAGAAAA
TCTGCTTGGCACGCCGCTGTGCAGTCTGCTGGTGTGGTAGATCAGCAGCTTCACGGTGCCGTCGGGTTTCTGCTGATAC
CAGTTCAGGTACTTGCTGATGTCCTGGCTGGCTCTACAGCTGATGGTCACTCTATCGCCCAGGCTGGCAGACAGGCTGC
TTGTGGTCTGGGTCATCTGGATGTCGGGCCGAGCGGCGTGGAGCAGAAGAGCCAGCGGAAGCAGCAGGGCGGTGACAGG
GAGGGCCATGGTGGCGGCTTCGAATCACGACACCTGAAATGGAAGAAAAAAACTTTGAACCACTGTCTGAGGCTTGAGA
ATGAACCAAGATCCAAACTCAAAAAGGGCAAATTCCAAGGAGAATTACATCAAGTGCCAAGCTGGCCTAACTTCAGTCT
CCACCCACTCAGTGTGGGGAAACTCCATCGCATAAAACCCCTCCCCCCAACCTAAAGACGACGTACTCCAAAAGCTCGA
GAACTAATCGAGGTGCCTGGACGGCGCCCGGTACTCCGTGGAGTCACATGAAGCGACGGCTGAGGACGGAAAGGCCCTT
TTCCTTTGTGTGGGTGACTCACCCGCCCGCTCTCCCGAGCGCCGCGTCCTCCATTTTGAGCTCCCTGCAGCAGGGCCGG
GAAGCGGCCATCTTTCCGCTCACGCAACTGGTGCCGACCGGGCCAGCCTTGCCGCCCAGGGCGGGGCGATACACGGCGG
CGCGAGGCCAGGCACCAGAGCAGGCCGGCCAGCTTGAGACTACCCCCGTCCGATTCTCGGTGGCCGCGCTCGCAGGCCC
CGCCTCGCCGAACATGTGCGCTGGGACGCACGGGCCCCGTCGCCGCCCGCGGCCCCAAAAACCGAAATACCAGTGTGCA
GATCTTGGCCCGCATTTACAAGACTATCTTGCCAGAAAAAAAGCGTCGCAGCAGGTCATCAAAAATTTTAAATGGCTAG
AGACTTATCGAAAGCAGCGAGACAGGCGCGAAGGTGCCACCAGATTCGCACGCGGCGGCCCCAGCGCCCAAGCCAGGCC
TCAACTCAAGCACGAGGCGAAGGGGCTCCTTAAGCGCAAGGCCTCGAACTCTCCCACCCACTTCCAACCCGAAGCTCGG
GATCAAGAATCACGTACTGCAGCCAGGGGCGTGGAAGTAATTCAAGGCACGCAAGGGCCATAACCCGTAAAGAGGCCAG
GCCCGCGGGAACCACACACGGCACTTACCTGTGTTCTGGCGGCAAACCCGTTGCGAAAAAGAACGTTCACGGCGACTAC
TGCACTTATATACGGTTCTCCCCCACCCTCGGGAAAAAGGCGGAGCCAGTACACGACATCACTTTCCCAGTTTACCCCG
CGCCACCTTCTCTAGGCACCGGTTCAATTGCCGACCCCTCCCCCCAACTTCTCGGGGACTGTGGGCGATGTGCGCTCTG
CCCACTGACGGGCACCGGAGCCTCACGCATGCTCTTCTCCACCTCAGTGATGACGAGAGCGGGCGGGTGAGGGGGCGGG
AACGCAGCGATCTCTGGGTTCTACGTTAGTGGGAGTTTAACGACGGTCCCTGGGATTCCCCAAGGCAGGGGCGAGTCCT
TTTGTATGAATTACTCCATGGTAATAGCGATGACTAATACGTAGATGTACTGCCAAGTAGGAAAGTCCCATAAGGTCAT
GTACTGGGCATAATACTAGTATCGATAAAATTTTGAATTTTGTAATTTGTTTTTGTAATTCTTTAGTTTGTATGTCTGT
TGCTATTATGTCTACTATTCTTTCCCCTGCACTGTACCCCCCAATCCCCCCTTTTCTTTTAAAAGTTAACCGATACCGT
CGAGATCCGTTCACTAATCGAATGGATCTGTCTCTGTCTCTCTCTCCACCTTCTTCTTCTATTCCTTCGGGCCTGTCGG
GTCCCCTCGGGGTTGGGAGGTGGGTCTGAAACGATAATGGTGAATATCCCTGCCTAACTCTATTCACTATAGAAAGTAC

AGCAAAAACTATTCTTAAACCTACCAAGCCTCCTACTATCATTATGAATAATTTTATATACCACAGCCAATTTGTTATG
TTAAACCAATTCCACAAACTTGCCCATTTATCTAATTCCAATAATTCTTGTTCATTCTTTTCTTGCTGGTTTTGCGATT
CTTCAATTAAGGAGTGTATTAAGCTTGTGTAATTGTTAATTTCTCTGTCCCACTCCATCCAGGTCGTGTGATTCCAAAT
CTGTTCCAGAGATTTATTACTCCAACTAGCATTCCAAGGCACAGCAGTGGTGCAAATGAGTTTTCCAGAGCAACCCCAA
ATCCCCAGGAGCTGTTGATCCTTTAGGTATCTTTCCACAGCCAGGATTCTTGCCTGGAGCTGCTTGATGCCCCAGACTG
TGAGTTGCAACAGATGCTGTTGCGCCTCAATAGCCCTCAGCAAATTGTTCTGCTGCTGCACTATACCAGACAATAATTG
TCTGGCCTGTACCGTCAGCGTCATTGACGCTGCGCCCATAGTGCTTCCTGCTGCTCCCAAGAACCCAAGGAACAAAGCT
CCTATTCCCACTGCTCTTTTTTCTCTCTGCACCACTCTTCTCTTTGCCTTGGTGGGTGCTACTCCTAATGGTTCAATTT
TTACTACTTTATATTTATATAATTCACTTCTCCAATTGTCCCTCATATCTCCTCCTCCAGGTCTGAAGATCAGTGGCCG
CTTGCTGTGCGGTGGTCTTACTTTTGTTTTGCTCTTCCTCTATCTTGTCTAAAGCTTCCTTGGTGTCTTTTATCTCTAT
CCTTTGATGCACACAATAGAGGGTTGCTACTGTATTATATAATGATCTAAGTTCTTCTGATCCTGTCTGAAGGGATGGT
TGTAGCTGTCCCAGTATTTGTCTACAGCCTTCTGATGTTTCTAACAGGCCAGGATTAACTGCGAATCGTTCTAGCTCCC
TGCTTGCCCATACTATATGTTTTAATTTATATTTTTTCTTTCCCCCTGGCCTTAACCGAATTTTTTCCCATCGCGATCT
AATTCTCCCCCGCTTAATACTGACGCTCTCGCACCCATCTCTCTCCTTCTAGCCTCCGCTAGTCAAAATTTTTGGCGTA
CTCACCAGTCGCCGCCCCTCGCCTCTTGCCGTGCGCGCTTCAGCAAGCCGAGTCCTGCGTCGAGAGAGCTCCTCTGGTT
TCCCTTTCGCTTTCAAGTCCCTGTTCGGGCGCCACTGCTAGAGATTTTCCACACTGACTAAAAGGGTCTGAGGGATCTC
TAGTTACCAGAGTCACACAACAGACGGGCACACACTACTTGAAGCACTCAAGGCAAGCTTTATTGAGGCTTAAGCAGTG
GGTTCCCTAGTTAGCCAGAGAGCTCCCAGGCTCAGATCTGGTCTAACCAGAGAGACCCAGTACAAGCAAAAAGCAGATC
TTATCTTCGTTGGGAGTGAATTAGCCCTTCCA
>IS8_VCN4-VCN5_chr6:15601040
TGCTAGAGATTTTCCACACTGACTAAAAGGGTCTGAGGGATCTCTAGTTACCAGAGTCACACAACAGACGGGCACACAC
TACTTGAAGCACTCAAGGCAAGCTTTATTGAGGCTTAAGCAGTGGGTTCCCTAGTTAGCCAGAGAGCTCCCAGGCTCAG
ATCTGGTCTAACCAGAGAGACCCAGTACAAGCAAAAAGCAGATCTTATCTTCGTTGGGAGTGAATTAGCCCTTCCAGTC
CCCCCTTTTCTTTTAAAAAGTGGCTAAGATCTACAGCTGCCTTGTAAGTCATTGGTCTTAAATTATCAGTCCTGCTCCT
CTGCCACAAAGTGCACGCAGTTGCCGGCCGGGTCGCGCAGGGCGAACTCCCGCCCCCACGGCTGCTCGCCGATCTCGGT
CATGGCCGGCCCGGAGGCGTCCCGGAAGTTCGTGGACACGACCTCCGACCACTCGGCGTACAGCTCGTCCAGGCCGCGC
ACCCACACCCAGGCCAGGGTGTTGTCCGGCACCACCTGGTCCTGGACCGCGCTGATGAACAGGGTCACGTCGTCCCGGA
CCACACCGGCGAAGTCGTCCTCCACGAAGTCCCGGGAGAACCCGAGCCGGTCGGTCCAGAACTCGACCGCTCCGGCGAC
GTCGCGCGCGGTGAGCACCGGAACGGCACTGGTCAACTTGGCCATAGGACCGGGGTTTTCTTCCACGTCTCCTGCTTGC
TTTAACAGAGAGAAGTTCGTGGCTCCAGAACCCTTGTACAGCTCGTCCATGCCGAGAGTGATCCCGGCGGCGGTCACGA
ACTCCAGCAGGACCATGTGATCGCGCTTCTCGTTGGGGTCTTTGCTCAGGGCGGACTGGGTGCTCAGGTAGTGGTTGTC
GGGCAGCAGCACGGGGCCGTCGCCGATGGGGGTGTTCTGCTGGTAGTGGTCGGCGAGCTGCACGCTGCCGTCCTCGATG
TTGTGGCGGATCTTGAAGTTCACCTTGATGCCGTTCTTCTGCTTGTCGGCCATGATATAGACGTTGTGGCTGTTGTAGT
TGTACTCCAGCTTGTGCCCCAGGATGTTGCCGTCCTCCTTGAAGTCGATGCCCTTCAGCTCGATGCGGTTCACCAGGGT
GTCGCCCTCGAACTTCACCTCGGCGCGGGTCTTGTAGTTGCCGTCGTCCTTGAAGAAGATGGTGCGCTCCTGGACGTAG
CCTTCGGGCATGGCGGACTTGAAGAAGTCGTGCTGCTTCATGTGGTCGGGGTAGCGGCTGAAGCACTGCACGCCGTAGG
TCAGGGTGGTCACGAGGGTGGGCCAGGGCACGGGCAGCTTGCCGGTGGTGCAGATGAACTTCAGGGTCAGCTTGCCGTA
GGTGGCATCGCCCTCGCCCTCGCCGGACACGCTGAACTTGTGGCCGTTTACGTCGCCGTCCAGCTCGACCAGGATGGGC
ACCACCCCGGTGAACAGCTCCTCGCCCTTGCTCACCATGGTGGCAAGCTTGGGCTGCAGGTCGAAAGGCCCGGAGATGA
GGAAGAGGAGAACAGCGCGGCAGACGTGCGCTTTTGAAGCGTGCAGAATGCCGGGCCTCCGGAGGACCTTCGGGCGCCC
GCCCCGCCCCTGAGCCCGCCCCTGAGCCCGCCCCCGGACCCACCCCTTCCCAGCCTCTGAGCCCAGAAAGCGAAGGAGC
AAAGCTGCTATTGGCCGCTGCCCCAAAGGCCTACCCGCTTCCATTGCTCAGCGGTGCTGTCCATCTGCACGAGACTAGT
GAGACGTGCTACTTCCATTTGTCACGTCCTGCACGACGCGAGCTGCGGGGCGGGGGGAACTTCCTGACTAGGGGAGGAG
TAGAAGGTGGCGCGAAGGGGCCACCAAAGAACGGAGCCGGTTGGCGCCTACCGGTGGATGTGGAATGTGTGCGAGGCCA
GAGGCCACTTGTGTAGCGCCAAGTGCCCAGCGGGGCTGCTAAAGCGCATGCTCCAGACTGCCTTGGGAAAAGCGCCTCC
CCTACCCGGTAGAATTGGTACCAGGCGGGGAGGCGGCCCAAAGGGAGATCCGACTCGTCTGAGGGCGAAGGCGAAGACG
CGGAAGAGGCCGCAAG
>IS9_VCN4-VCN5_chr6:24825065
GTACTTGGAAGGGCTAATTCACTCCCAACGAAGATAAGATCTGCTTTTTGCTTGTACTGGGTCTCTCTGGTTAGACCAG
ATCTGAGCCTGGGAGCTCTCTGGCTAACTAGGGAACCCACTGCTTAAGCCTCAATAAAGCTTGCCTTGAGTGCTTCAAG
TAGTGTGTGCCCGTCTGTTGTGTGACTCTGGTAACTAGAGATCCCTCAGACCCTTTTAGTCAGTGTGGAAAATCTCTAG

CAGTGGCGCCCGAACAGGGACTTGAAAGCGAAAGGGAAACCAGAGGAGCTCTCTCGACGCAGGACTCGGCTTGCTGAAG
CGCGCACGGCAAGAGGCGAGGGGCGGCGACTGGTGAGTACGCCAAAAATTTTGACTAGCGGAGGCTAGAAGGAGAGAGA
TGGGTGCGAGAGCGTCAGTATTAAGCGGGGGAGAATTAGATCGCGATGGGAAAAAATTCGGTTAAGGCCAGGGGGAAAG
AAAAAATATAAATTAAAACATATAGTATGGGCAAGCAGGGAGCTAGAACGATTCGCAGTTAATCCTGGCCTGTTAGAAA
CATCAGAAGGCTGTAGACAAATACTGGGACAGCTACAACCATCCCTTCAGACAGGATCAGAAGAACTTAGATCATTATA
TAATACAGTAGCAACCCTCTATTGTGTGCATCAAAGGATAGAGATAAAAGACACCAAGGAAGCTTTAGACAAGATAGAG
GAAGAGCAAAACAAAAGTAAGACCACCGCACAGCAAGCGGCCACTGATCTTCAGACCTGGAGGAGGAGATATGAGGGAC
AATTGGAGAAGTGAATTATATAAATATAAAGTAGTAAAAATTGAACCATTAGGAGTAGCACCCACCAAGGCAAAGAGAA
GAGTGGTGCAGAGAGAAAAAAGAGCAGTGGGAATAGGAGCTTTGTTCCTTGGGTTCTTGGGAGCAGCAGGAAGCACTAT
GGGCGCAGCGTCAATGACGCTGACGGTACAGGCCAGACAATTATTGTCTGGTATAGTGCAGCAGCAGAACAATTTGCTG
AGGGCTATTGAGGCGCAACAGCATCTGTTGCAACTCACAGTCTGGGGCATCAAGCAGCTCCAGGCAAGAATCCTGGCTG
TGGAAAGATACCTAAAGGATCAACAGCTCCTGGGGATTTGGGGTTGCTCTGGAAAACTCATTTGCACCACTGCTGTGCC
TTGGAATGCTAGTTGGAGTAATAAATCTCTGGAACAGATTTGGAATCACACGACCTGGATGGAGTGGGACAGAGAAATT
AACAATTACACAAGCTTAATACACTCCTTAATTGAAGAATCGCAAAACCAGCAAGAAAAGAATGAACAAGAATTATTGG
AATTAGATAAATGGGCAAGTTTGTGGAATTGGTTTAACATAACAAATTGGCTGTGGTATATAAAATTATTCATAATGAT
AGTAGGAGGCTTGGTAGGTTTAAGAATAGTTTTTGCTGTACTTTCTATAGTGAATAGAGTTAGGCAGGGATATTCACCA
TTATCGTTTCAGACCCACCTCCCAACCCCGAGGGGACCCGACAGGCCCGAAGGAATAGAAGAAGAAGGTGGAGAGAGAG
ACAGAGACAGATCCATTCGATTAGTGAACGGATCTCGACGGTATCGGTTAACTTTTAAAAGAAAAGGGGGGATTGGGGG
GTACAGTGCAGGGGAAAGAATAGTAGACATAATAGCAACAGACATACAAACTAAAGAATTACAAAAACAAATTACAAAA
TTCAAAATTTTATCGATACTAGTATTATGCCCAGTACATGACCTTATGGGACTTTCCTACTTGGCAGTACATCTACGTA
TTAGTCATCGCTATTACCATGGAGTAATTCATACAAAAGGACTCGCCCCTGCCTTGGGGAATCCCAGGGACCGTCGTTA
AACTCCCACTAACGTAGAACCCAGAGATCGCTGCGTTCCCGCCCCCTCACCCGCCCGCTCTCGTCATCACTGAGGTGGA
GAAGAGCATGCGTGAGGCTCCGGTGCCCGTCAGTGGGCAGAGCGCACATCGCCCACAGTCCCCGAGAAGTTGGGGGGAG
GGGTCGGCAATTGAACCGGTGCCTAGAGAAGGTGGCGCGGGGTAAACTGGGAAAGTGATGTCGTGTACTGGCTCCGCCT
TTTTCCCGAGGGTGGGGGAGAACCGTATATAAGTGCAGTAGTCGCCGTGAACGTTCTTTTTCGCAACGGGTTTGCCGCC
AGAACACAGGTAAGTGCCGTGTGTGGTTCCCGCGGGCCTGGCCTCTTTACGGGTTATGGCCCTTGCGTGCCTTGAATTA
CTTCCACGCCCCTGGCTGCAGTACGTGATTCTTGATCCCGAGCTTCGGGTTGGAAGTGGGTGGGAGAGTTCGAGGCCTT
GCGCTTAAGGAGCCCCTTCGCCTCGTGCTTGAGTTGAGGCCTGGCTTGGGCGCTGGGGCCGCCGCGTGCGAATCTGGTG
GCACCTTCGCGCCTGTCTCGCTGCTTTCGATAAGTCTCTAGCCATTTAAAATTTTTGATGACCTGCTGCGACGCTTTTT
TTCTGGCAAGATAGTCTTGTAAATGCGGGCCAAGATCTGCACACTGGTATTTCGGTTTTTGGGGCCGCGGGCGGCGACG
GGGCCCGTGCGTCCCAGCGCACATGTTCGGCGAGGCGGGGCCTGCGAGCGCGGCCACCGAGAATCGGACGGGGGTAGTC
TCAAGCTGGCCGGCCTGCTCTGGTGCCTGGCCTCGCGCCGCCGTGTATCGCCCCGCCCTGGGCGGCAAGGCTGGCCCGG
TCGGCACCAGTTGCGTGAGCGGAAAGATGGCCGCTTCCCGGCCCTGCTGCAGGGAGCTCAAAATGGAGGACGCGGCGCT
CGGGAGAGCGGGCGGGTGAGTCACCCACACAAAGGAAAAGGGCCTTTCCGTCCTCAGCCGTCGCTTCATGTGACTCCAC
GGAGTACCGGGCGCCGTCCAGGCACCTCGATTAGTTCTCGAGCTTTTGGAGTACGTCGTCTTTAGGTTGGGGGGAGGGG
TTTTATGCGATGGAGTTTCCCCACACTGAGTGGGTGGAGACTGAAGTTAGGCCAGCTTGGCACTTGATGTAATTCTCCT
TGGAATTTGCCCTTTTTGAGTTTGGATCTTGGTTCATTCTCAAGCCTCAGACAGTGGTTCAAAGTTTTTTTCTTCCATT
TCAGGTGTCGTGATTCGAAGCCGCCACCATGGCCCTCCCTGTCACCGCCCTGCTGCTTCCGCTGGCTCTTCTGCTCCAC
GCCGCTCGGCCCGACATCCAGATGACCCAGACCACAAGCAGCCTGTCTGCCAGCCTGGGCGATAGAGTGACCATCAGCT
GTAGAGCCAGCCAGGACATCAGCAAGTACCTGAACTGGTATCAGCAGAAACCCGACGGCACCGTGAAGCTGCTGATCTA
CCACACCAGCAGACTGCACAGCGGCGTGCCAAGCAGATTTTCTGGCAGCGGCTCTGGCACCGACTACAGCCTGACAATC
AGCAACCTGGAACAAGAGGATATCGCTACCTACTTCTGCCAGCAAGGCAACACCCTGCCTTACACCTTTGGCGGAGGCA
CCAAGCTGGAAATCACCGGAGGCGGAGGATCTGGTGGCGGAGGAAGTGGCGGAGGCGGTTCTGAAGTGAAGCTGCAAGA
GTCTGGCCCTGGACTGGTGGCCCCATCTCAGTCTCTGAGCGTGACCTGTACAGTCAGCGGAGTGTCCCTGCCTGATTAC
GGCGTGTCCTGGATCAGACAGCCTCCTCGGAAAGGCCTGGAATGGCTGGGAGTGATCTGGGGCAGCGAGACAACCTACT
ACAACAGCGCCCTGAAGTCCCGGCTGACCATCATCAAGGACAACAGCAAGAGCCAGGTGTTCCTGAAGATGAACAGCCT
GCAGACCGACGACACCGCCATCTACTACTGCGCCAAGCACTACTACTACGGCGGCAGCTACGCCATGGATTATTGGGGC
CAGGGCACCAGCGTGACCGTTTCTTCTACCACGACGCCAGCGCCGCGACCACCAACACCGGCGCCCACCATCGCGTCGC
AGCCCCTGTCCCTGCGCCCAGAAGCGTGCCGGCCAGCGGCGGGGGGCGCAGTGCACACGAGGGGGCTGGACTTCGCCTG
TGATTTCTGGGTGCTGGTCGTTGTGGGCGGCGTGCTGGCCTGCTACAGCCTGCTGGTGACAGTGGCCTTCATCATCTTT
TGGGTGAGGAGCAAGCGGAGCAGACTGCTGCACAGCGACTACATGAACATGACCCCCCGGAGGCCTGGCCCCACCCGGA

AGCACTACCAGCCCTACGCCCCTCCCAGGGATTTCGCCGCCTACCGGAGCAAACGGGGCAGAAAGAAACTCCTGTATAT
ATTCAAACAACCATTTATGAGACCAGTACAAACTACTCAAGAGGAAGATGGCTGTAGCTGCCGATTTCCAGAAGAAGAA
GAAGGAGGATGTGAACTGAGAGTGAAGTTCAGCAGGAGCGCAGACGCCCCCGCGTACAAGCAGGGCCAGAACCAGCTCT
ATAACGAGCTCAATCTAGGACGAAGAGAGGAGTACGATGTTTTGGACAAGAGACGTGGCCGGGACCCTGAGATGGGGGG
AAAGCCGAGAAGGAAGAACCCTCAGGAAGGCCTGTACAATGAACTGCAGAAAGATAAGATGGCGGAGGCCTACAGTGAG
ATTGGGATGAAAGGCGAGCGCCGGAGGGGCAAGGGGCACGATGGCCTTTACCAGGGTCTCAGTACAGCCACCAAGGACA
CCTACGACGCCCTTCACATGCAGGCCCTGCCCCCTCGCTAATAATCTAGAAATCAACCTCTGGATTACAAAATTTGTGA
AAGATTGACTGGTATTCTTAACTATGTTGCTCCTTTTACGCTATGTGGATACGCTGCTTTAATGCCTTTGTATCATGCT
ATTGCTTCCCGTATGGCTTTCATTTTCTCCTCCTTGTATAAATCCTGGTTGCTGTCTCTTTATGAGGAGTTGTGGCCCG
TTGTCAGGCAACGTGGCGTGGTGTGCACTGTGTTTGCTGACGCAACCCCCACTGGTTGGGGCATTGCCACCACCTGTCA
GCTCCTTTCCGGGACTTTCGCTTTCCCCCTCCCTATTGCCACGGCGGAACTCATCGCCGCCTGCCTTGCCCGCTGCTGG
ACAGGGGCTCGGCTGTTGGGCACTGACAATTCCGTGGTGTTGTCGGGGAAATCATCGTCCTTTCCTTGGCTGCTCGCCT
GTGTTGCCACCTGGATTCTGCGCGGGACGTCCTTCTGCTACGTCCCTTCGGCCCTCAATCCAGCGGACCTTCCTTCCCG
CGGCCTGCTGCCGGCTCTGCGGCCTCTTCCGCGTCTTCGCCTTCGCCCTCAGACGAGTCGGATCTCCCTTTGGGCCGCC
TCCCCGCCTGGTACCAATTCTACCGGGTAGGGGAGGCGCTTTTCCCAAGGCAGTCTGGAGCATGCGCTTTAGCAGCCCC
GCTGGGCACTTGGCGCTACACAAGTGGCCTCTGGCCTCGCACACATTCCACATCCACCGGTAGGCGCCAACCGGCTCCG
TTCTTTGGTGGCCCCTTCGCGCCACCTTCTACTCCTCCCCTAGTCAGGAAGTTCCCCCCCGCCCCGCAGCTCGCGTCGT
GCAGGACGTGACAAATGGAAGTAGCACGTCTCACTAGTCTCGTGCAGATGGACAGCACCGCTGAGCAATGGAAGCGGGT
AGGCCTTTGGGGCAGCGGCCAATAGCAGCTTTGCTCCTTCGCTTTCTGGGCTCAGAGGCTGGGAAGGGGTGGGTCCGGG
GGCGGGCTCAGGGGCGGGCTCAGGGGCGGGGCGGGCGCCCGAAGGTCCTCCGGAGGCCCGGCATTCTGCACGCTTCAAA
AGCGCACGTCTGCCGCGCTGTTCTCCTCTTCCTCATCTCCGGGCCTTTCGACCTGCAGCCCAAGCTTGCCACCATGGTG
AGCAAGGGCGAGGAGCTGTTCACCGGGGTGGTGCCCATCCTGGTCGAGCTGGACGGCGACGTAAACGGCCACAAGTTCA
GCGTGTCCGGCGAGGGCGAGGGCGATGCCACCTACGGCAAGCTGACCCTGAAGTTCATCTGCACCACCGGCAAGCTGCC
CGTGCCCTGGCCCACCCTCGTGACCACCCTGACCTACGGCGTGCAGTGCTTCAGCCGCTACCCCGACCACATGAAGCAG
CACGACTTCTTCAAGTCCGCCATGCCCGAAGGCTACGTCCAGGAGCGCACCATCTTCTTCAAGGACGACGGCAACTACA
AGACCCGCGCCGAGGTGAAGTTCGAGGGCGACACCCTGGTGAACCGCATCGAGCTGAAGGGCATCGACTTCAAGGAGGA
CGGCAACATCCTGGGGCACAAGCTGGAGTACAACTACAACAGCCACAACGTCTATATCATGGCCGACAAGCAGAAGAAC
GGCATCAAGGTGAACTTCAAGATCCGCCACAACATCGAGGACGGCAGCGTGCAGCTCGCCGACCACTACCAGCAGAACA
CCCCCATCGGCGACGGCCCCGTGCTGCTGCCCGACAACCACTACCTGAGCACCCAGTCCGCCCTGAGCAAAGACCCCAA
CGAGAAGCGCGATCACATGGTCCTGCTGGAGTTCGTGACCGCCGCCGGGATCACTCTCGGCATGGACGAGCTGTACAAG
GGTTCTGGAGCCACGAACTTCTCTCTGTTAAAGCAAGCAGGAGACGTGGAAGAAAACCCCGGTCCTATGGCCAAGTTGA
CCAGTGCCGTTCCGGTGCTCACCGCGCGCGACGTCGCCGGAGCGGTCGAGTTCTGGACCGACCGGCTCGGGTTCTCCCG
GGACTTCGTGGAGGACGACTTCGCCGGTGTGGTCCGGGACGACGTGACCCTGTTCATCAGCGCGGTCCAGGACCAGGTG
GTGCCGGACAACACCCTGGCCTGGGTGTGGGTGCGCGGCCTGGACGAGCTGTACGCCGAGTGGTCGGAGGTCGTGTCCA
CGAACTTCCGGGACGCCTCCGGGCCGGCCATGACCGAGATCGGCGAGCAGCCGTGGGGGCGGGAGTTCGCCCTGCGCGA
CCCGGCCGGCAACTGCGTGCACTTTGTGGCAGAGGAGCAGGACTGATAATTTAAGACCAATGACTTACAAGGCAGCTGT
AGATCTTAGCCACTTTTTAAAAGAAAAGGGGGGACTGGAAGGGCTAATTCACTCCCAACGAAGATAAGATCTGCTTTTT
GCTTGTACTGGGTCTCTCTGGTTAGACCAGATCTGAGCCTGGGAGCTCTCTGGCTAACTAGGGAACCCACTGCTTAAGC
CTCAATAAAGCTTGCCTTGAGTGCTTCAAGTAGTGTGTGCCCGTCTGTTGTGTGACTCTGGTAACTAGAGATCCCTCAG
ACCCTTTTAGTCAGTGTGGAAAATCTCTAGCA
>IS10_VCN5_chr5:39044696
TTCACTGCTAGAGATTTTCCACACTGACTAAAAGGGTCTGAGGGATCTCTAGTTACCAGAGTCACACAACAGACGGGCA
CACACTACTTGAAGCACTCAAGGCAAGCTTTATTGAGGCTTAAGCAGTGGGTTCCCTAGTTAGCCAGAGAGCTCCCAGG
CTCAGATCTGGTCTAACCAGAGAGACCCAGTACAAGCAAAAAGCAGATCTTATCTTCGTTGGGAGTGAATTAGCCCTTC
CAGTCCCCCCTTTTCTTTTAAAAAGTGGCTAAGATCTACAGCTGCCTTGTAAGTCATTGGTCTTAAATTATCAGTCCTG
CTCCTCTGCCACAAAGTGCACGCAGTTGCCGGCCGGGTCGCGCAGGGCGAACTCCCGCCCCCACGGCTGCTCGCCGATC
TCGGTCATGGCCGGCCCGGAGGCGTCCCGGAAGTTCGTGGACACGACCTCCGACCACTCGGCGTACAGCTCGTCCAGGC
CGCGCACCCACACCCAGGCCAGGGTGTTGTCCGGCACCACCTGGTCCTGGACCGCGCTGATGAACAGGGTCACGTCGTC
CCGGACCACACCGGCGAAGTCGTCCTCCACGAAGTCCCGGGAGAACCCGAGCCGGTCGGTCCAGAACTCGACCGCTCCG
GCGACGTCGCGCGCGGTGAGCACCGGAACGGCACTGGTCAACTTGGCCATAGGACCGGGGTTTTCTTCCACGTCTCCTG
CTTGCTTTAACAGAGAGAAGTTCGTGGCTCCAGAACCCTTGTACAGCTCGTCCATGCCGAGAGTGATCCCGGCGGCGGT

CACGAACTCCAGCAGGACCATGTGATCGCGCTTCTCGTTGGGGTCTTTGCTCAGGGCGGACTGGGTGCTCAGGTAGTGG
TTGTCGGGCAGCAGCACGGGGCCGTCGCCGATGGGGGTGTTCTGCTGGTAGTGGTCGGCGAGCTGCACGCTGCCGTCCT
CGATGTTGTGGCGGATCTTGAAGTTCACCTTGATGCCGTTCTTCTGCTTGTCGGCCATGATATAGACGTTGTGGCTGTT
GTAGTTGTACTCCAGCTTGTGCCCCAGGATGTTGCCGTCCTCCTTGAAGTCGATGCCCTTCAGCTCGATGCGGTTCACC
AGGGTGTCGCCCTCGAACTTCACCTCGGCGCGGGTCTTGTAGTTGCCGTCGTCCTTGAAGAAGATGGTGCGCTCCTGGA
CGTAGCCTTCGGGCATGGCGGACTTGAAGAAGTCGTGCTGCTTCATGTGGTCGGGGTAGCGGCTGAAGCACTGCACGCC
GTAGGTCAGGGTGGTCACGAGGGTGGGCCAGGGCACGGGCAGCTTGCCGGTGGTGCAGATGAACTTCAGGGTCAGCTTG
CCGTAGGTGGCATCGCCCTCGCCCTCGCCGGACACGCTGAACTTGTGGCCGTTTACGTCGCCGTCCAGCTCGACCAGGA
TGGGCACCACCCCGGTGAACAGCTCCTCGCCCTTGCTCACCATGGTGGCAAGCTTGGGCTGCAGGTCGAAAGGCCCGGA
GATGAGGAAGAGGAGAACAGCGCGGCAGACGTGCGCTTTTGAAGCGTGCAGAATGCCGGGCCTCCGGAGGACCTTCGGG
CGCCCGCCCCGCCCCTGAGCCCGCCCCTGAGCCCGCCCCCGGACCCACCCCTTCCCAGCCTCTGAGCCCAGAAAGCGAA
GGAGCAAAGCTGCTATTGGCCGCTGCCCCAAAGGCCTACCCGCTTCCATTGCTCAGCGGTGCTGTCCATCTGCACGAGA
CTAGTGAGACGTGCTACTTCCATTTGTCACGTCCTGCACGACGCGAGCTGCGGGGCGGGGGGGAACTTCCTGACTAGGG
GAGGAGTAGAAGGTGGCGCGAAGGGGCCACCAAAGAACGGAGCCGGTTGGCGCCTACCGGTGGATGTGGAATGTGTGCG
AGGCCAGAGGCCACTTGTGTAGCGCCAAGTGCCCAGCGGGGCTGCTAAAGCGCATGCTCCAGACTGCCTTGGGAAAAGC
GCCTCCCCTACCCGGTAGAATTGGTACCAGGCGGGGAGGCGGCCCAAAGGGAGATCCGACTCGTCTGAGGGCGAAGGCG
AAGACGCGGAAGAGGCCGCAGAGCCGGCAGCAGGCCGCGGGAAGGAAGGTCCGCTGGATTGAGGGCCGAAGGGACGTAG
CAGAAGGACGTCCCGCGCAGAATCCAGGTGGCAACACAGGCGAGCAGCCAAGGAAAGGACGATGATTTCCCCGACAACA
CCACGGAATTGTCAGTGCCCAACAGCCGAGCCCCTGTCCAGCAGCGGGCAAGGCAGGCGGCGATGAGTTCCGCCGTGGC
AATAGGGAGGGGGAAAGCGAAAGTCCCGGAAAGGAGCTGACAGGTGGTGGCAATGCCCCAACCAGTGGGGGTTGCGTCA
GCAAACACAGTGCACACCACGCCACGTTGCCTGACAACGGGCCACAACTCCTCATAAAGAGACAGCAACCAGGATTTAT
ACAAGGAGGAGAAAATGAAAGCCATACGGGAAGCAATAGCATGATACAAAGGCATTAAAGCAGCGTATCCACATAGCGT
AAAAGGAGCAACATAGTTAAGAATACCAGTCAATCTTTCACAAATTTTGTAATCCAGAGGTTGATTTCTAGATTATTAG
CGAGGGGGCAGGGCCTGCATGTGAAGGGCGTCGTAGGTGTCCTTGGTGGCTGTACTGAGACCCTGGTAAAGGCCATCGT
GCCCCTTGCCCCTCCGGCGCTCGCCTTTCATCCCAATCTCACTGTAGGCCTCCGCCATCTTATCTTTCTGCAGTTCATT
GTACAGGCCTTCCTGAGGGTTCTTCCTTCTCGGCTTTCCCCCCATCTCAGGGTCCCGGCCACGTCTCTTGTCCAAAACA
TCGTACTCCTCTCTTCGTCCTAGATTGAGCTCGTTATAGAGCTGGTTCTGGCCCTGCTTGTACGCGGGGGCGTCTGCGC
TCCTGCTGAACTTCACTCTCAGTTCACATCCTCCTTCTTCTTCTTCTGGAAATCGGCAGCTACAGCCATCTTCCTCTTG
AGTAGTTTGTACTGGTCTCATAAATGGTTGTTTGAATATATACAGGAGTTTCTTTCTGCCCCGTTTGCTCCGGTAGGCG
GCGAAATCCCTGGGAGGGGCGTAGGGCTGGTAGTGCTTCCGGGTGGGGCCAGGCCTCCGGGGGGTCATGTTCATGTAGT
CGCTGTGCAGCAGTCTGCTCCGCTTGCTCCTCACCCAAAAGATGATGAAGGCCACTGTCACCAGCAGGCTGTAGCAGGC
CAGCACGCCGCCCACAACGACCAGCACCCAGAAATCACAGGCGAAGTCCAGCCCCCTCGTGTGCACTGCGCCCCCCGCC
GCTGGCCGGCACGCTTCTGGGCGCAGGGACAGGGGCTGCGACGCGATGGTGGGCGCCGGTGTTGGTGGTCGCGGCGCTG
GCGTCGTGGTAGAAGAAACGGTCACGCTGGTGCCCTGGCCCCAATAATCCATGGCGTAGCTGCCGCCGTAGTAGTAGTG
CTTGGCGCAGTAGTAGATGGCGGTGTCGTCGGTCTGCAGGCTGTTCATCTTCAGGAACACCTGGCTCTTGCTGTTGTCC
TTGATGATGGTCAGCCGGGACTTCAGGGCGCTGTTGTAGTAGGTTGTCTCGCTGCCCCAGATCACTCCCAGCCATTCCA
GGCCTTTCCGAGGAGGCTGTCTGATCCAGGACACGCCGTAATCAGGCAGGGACACTCCGCTGACTGTACAGGTCACGCT
CAGAGACTGAGATGGGGCCACCAGTCCAGGGCCAGACTCTTGCAGCTTCACTTCAGAACCGCCTCCGCCACTTCCTCCG
CCACCAGATCCTCCGCCTCCGGTGATTTCCAGCTTGGTGCCTCCGCCAAAGGTGTAAGGCAGGGTGTTGCCTTGCTGGC
AGAAGTAGGTAGCGATATCCTCTTGTTCCAGGTTGCTGATTGTCAGGCTGTAGTCGGTGCCAGAGCCGCTGCCAGAAAA
TCTGCTTGGCACGCCGCTGTGCAGTCTGCTGGTGTGGTAGATCAGCAGCTTCACGGTGCCGTCGGGTTTCTGCTGATAC
CAGTTCAGGTACTTGCTGATGTCCTGGCTGGCTCTACAGCTGATGGTCACTCTATCGCCCAGGCTGGCAGACAGGCTGC
TTGTGGTCTGGGTCATCTGGATGTCGGGCCGAGCGGCGTGGAGCAGAAGAGCCAGCGGAAGCAGCAGGGCGGTGACAGG
GAGGGCCATGGTGGCGGCTTCGAATCACGACACCTGAAATGGAAGAAAAAAACTTTGAACCACTGTCTGAGGCTTGAGA
ATGAACCAAGATCCAAACTCAAAAAGGGCAAATTCCAAGGAGAATTACATCAAGTGCCAAGCTGGCCTAACTTCAGTCT
CCACCCACTCAGTGTGGGGAAACTCCATCGCATAAAACCCCTCCCCCCAACCTAAAGACGACGTACTCCAAAAGCTCGA
GAACTAATCGAGGTGCCTGGACGGCGCCCGGTACTCCGTGGAGTCACATGAAGCGACGGCTGAGGACGGAAAGGCCCTT
TTCCTTTGTGTGGGTGACTCACCCGCCCGCTCTCCCGAGCGCCGCGTCCTCCATTTTGAGCTCCCTGCAGCAGGGCCGG
GAAGCGGCCATCTTTCCGCTCACGCAACTGGTGCCGACCGGGCCAGCCTTGCCGCCCAGGGCGGGGCGATACACGGCGG
CGCGAGGCCAGGCACCAGAGCAGGCCGGCCAGCTTGAGACTACCCCCGTCCGATTCTCGGTGGCCGCGCTCGCAGGCCC
CGCCTCGCCGAACATGTGCGCTGGGACGCACGGGCCCCGTCGCCGCCCGCGGCCCCAAAAACCGAAATACCAGTGTGCA

```
GATCTTGGCCCGCATTTACAAGACTATCTTGCCAGAAAAAAAGCGTCGCAGCAGGTCATCAAAAATTTTAAATGGCTAG
AGACTTATCGAAAGCAGCGAGACAGGCGCGAAGGTGCCACCAGATTCGCACGCGGCGGCCCCAGCGCCCAAGCCAGGCC
TCAACTCAAGCACGAGGCGAAGGGGCTCCTTAAGCGCAAGGCCTCGAACTCTCCCACCCACTTCCAACCCGAAGCTCGG
GATCAAGAATCACGTACTGCAGCCAGGGGCGTGGAAGTAATTCAAGGCACGCAAGGGCCATAACCCGTAAAGAGGCCAG
GCCCGCGGGAACCACACACGGCACTTACCTGTGTTCTGGCGGCAAACCCGTTGCGAAAAAGAACGTTCACGGCGACTAC
TGCACTTATATACGGTTCTCCCCCACCCTCGGGAAAAAGGCGGAGCCAGTACACGACATCACTTTCCCAGTTTACCCCG
CGCCACCTTCTCTAGGCACCGGTTCAATTGCCGACCCCTCCCCCCAACTTCTCGGGGACTGTGGGCGATGTGCGCTCTG
CCCACTGACGGGCACCGGAGCCTCACGCATGCTCTTCTCCACCTCAGTGATGACGAGAGCGGGCGGGTGAGGGGGCGGG
AACGCAGCGATCTCTGGGTTCTACGTTAGTGGGAGTTTAACGACGGTCCCTGGGATTCCCCAAGGCAGGGGCGAGTCCT
TTTGTATGAATTACTCCATGGTAATAGCGATGACTAATACGTAGATGTACTGCCAAGTAGGAAAGTCCCATAAGGTCAT
GTACTGGGCATAATACTAGTATCGATAAAATTTTGAATTTTGTAATTTGTTTTTGTAATTCTTTAGTTTGTATGTCTGT
TGCTATTATGTCTACTATTCTTTCCCCTGCACTGTACCCCCCAATCCCCCCTTTTCTTTTAAAAGTTAACCGATACCGT
CGAGATCCGTTCACTAATCGAATGGATCTGTCTCTGTCTCTCTCTCCACCTTCTTCTTCTATTCCTTCGGGCCTGTCGG
GTCCCCTCGGGGTTGGGAGGTGGGTCTGAAACGATAATGGTGAATATCCCTGCCTAACTCTATTCACTATAGAAAGTAC
AGCAAAAACTATTCTTAAACCTACCAAGCCTCCTACTATCATTATGAATAATTTTATATACCACAGCCAATTTGTTATG
TTAAACCAATTCCACAAACTTGCCCATTTATCTAATTCCAATAATTCTTGTTCATTCTTTTCTTGCTGGTTTTGCGATT
CTTCAATTAAGGAGTGTATTAAGCTTGTGTAATTGTTAATTTCTCTGTCCCACTCCATCCAGGTCGTGTGATTCCAAAT
CTGTTCCAGAGATTTATTACTCCAACTAGCATTCCAAGGCACAGCAGTGGTGCAAATGAGTTTTCCAGAGCAACCCCAA
ATCCCCAGGAGCTGTTGATCCTTTAGGTATCTTTCCACAGCCAGGATTCTTGCCTGGAGCTGCTTGATGCCCCAGACTG
TGAGTTGCAACAGATGCTGTTGCGCCTCAATAGCCCTCAGCAAATTGTTCTGCTGCTGCACTATACCAGACAATAATTG
TCTGGCCTGTACCGTCAGCGTCATTGACGCTGCGCCCATAGTGCTTCCTGCTGCTCCCAAGAACCCAAGGAACAAAGCT
CCTATTCCCACTGCTCTTTTTTCTCTCTGCACCACTCTTCTCTTTGCCTTGGTGGGTGCTACTCCTAATGGTTCAATTT
TTACTACTTTATATTTATATAATTCACTTCTCCAATTGTCCCTCATATCTCCTCCTCCAGGTCTGAAGATCAGTGGCCG
CTTGCTGTGCGGTGGTCTTACTTTTGTTTTGCTCTTCCTCTATCTTGTCTAAAGCTTCCTTGGTGTCTTTTATCTCTAT
CCTTTGATGCACACAATAGAGGGTTGCTACTGTATTATATAATGATCTAAGTTCTTCTGATCCTGTCTGAAGGGATGGT
TGTAGCTGTCCCAGTATTTGTCTACAGCCTTCTGATGTTTCTAACAGGCCAGGATTAACTGCGAATCGTTCTAGCTCCC
TGCTTGCCCATACTATATGTTTTAATTTATATTTTTTCTTTCCCCCTGGCCTTAACCGAATTTTTTCCCATCGCGATCT
AATTCTCCCCCGCTTAATACTGACGCTCTCGCACCCATCTCTCTCCTTCTAGCCTCCGCTAGTCAAAATTTTTGGCGTA
CTCACCAGTCGCCGCCCCTCGCCTCTTGCCGTGCGCGCTTCAGCAAGCCGAGTCCTGCGTCGAGAGAGCTCCTCTGGTT
TCCCTTTCGCTTTCAAGTCCCTGTTCGGGCGCCACTGCTAGAGATTTTCCACACTGACTAAAAGGGTCTGAGGGATCTC
TAGTTACCAGAGTCACACAACAGACGGGCACACACTACTTGAAGCACTCAAGGCAAGCTTTATTGAGGCTTAAGCAGTG
GGTTCCCTAGTTAGCCAGAGAGCTCCCAGGCTCAGATCTGGTCTAACCAGAGAGACCCAGTACAAGCAAAAAGCAGATC
TTATCTTCGTTGGGAGTGAATTAGCCCTTCCA
>expected_full_provirus_sequence_7374bp
TGGAAGGGCTAATTCACTCCCAACGAAGATAAGATCTGCTTTTTGCTTGTACTGGGTCTCTCTGGTTAGACCAGATCTG
AGCCTGGGAGCTCTCTGGCTAACTAGGGAACCCACTGCTTAAGCCTCAATAAAGCTTGCCTTGAGTGCTTCAAGTAGTG
TGTGCCCGTCTGTTGTGTGACTCTGGTAACTAGAGATCCCTCAGACCCTTTTAGTCAGTGTGGAAAATCTCTAGCAGTG
GCGCCCGAACAGGGACTTGAAAGCGAAAGGGAAACCAGAGGAGCTCTCTCGACGCAGGACTCGGCTTGCTGAAGCGCGC
ACGGCAAGAGGCGAGGGGCGGCGACTGGTGAGTACGCCAAAAATTTTGACTAGCGGAGGCTAGAAGGAGAGAGATGGGT
GCGAGAGCGTCAGTATTAAGCGGGGGAGAATTAGATCGCGATGGGAAAAAATTCGGTTAAGGCCAGGGGGAAAGAAAAA
ATATAAATTAAAACATATAGTATGGGCAAGCAGGGAGCTAGAACGATTCGCAGTTAATCCTGGCCTGTTAGAAACATCA
GAAGGCTGTAGACAAATACTGGGACAGCTACAACCATCCCTTCAGACAGGATCAGAAGAACTTAGATCATTATATAATA
CAGTAGCAACCCTCTATTGTGTGCATCAAAGGATAGAGATAAAAGACACCAAGGAAGCTTTAGACAAGATAGAGGAAGA
GCAAAACAAAAGTAAGACCACCGCACAGCAAGCGGCCACTGATCTTCAGACCTGGAGGAGGAGATATGAGGGACAATTG
GAGAAGTGAATTATATAAATATAAAGTAGTAAAAATTGAACCATTAGGAGTAGCACCCACCAAGGCAAAGAGAAGAGTG
GTGCAGAGAGAAAAAAGAGCAGTGGGAATAGGAGCTTTGTTCCTTGGGTTCTTGGGAGCAGCAGGAAGCACTATGGGCG
CAGCGTCAATGACGCTGACGGTACAGGCCAGACAATTATTGTCTGGTATAGTGCAGCAGCAGAACAATTTGCTGAGGGC
TATTGAGGCGCAACAGCATCTGTTGCAACTCACAGTCTGGGGCATCAAGCAGCTCCAGGCAAGAATCCTGGCTGTGGAA
AGATACCTAAAGGATCAACAGCTCCTGGGGATTTGGGGTTGCTCTGGAAAACTCATTTGCACCACTGCTGTGCCTTGGA
ATGCTAGTTGGAGTAATAAATCTCTGGAACAGATTTGGAATCACACGACCTGGATGGAGTGGGACAGAGAAATTAACAA
TTACACAAGCTTAATACACTCCTTAATTGAAGAATCGCAAAACCAGCAAGAAAAGAATGAACAAGAATTATTGGAATTA
```

GATAAATGGGCAAGTTTGTGGAATTGGTTTAACATAACAAATTGGCTGTGGTATATAAAATTATTCATAATGATAGTAG
GAGGCTTGGTAGGTTTAAGAATAGTTTTTGCTGTACTTTCTATAGTGAATAGAGTTAGGCAGGGATATTCACCATTATC
GTTTCAGACCCACCTCCCAACCCCGAGGGGACCCGACAGGCCCGAAGGAATAGAAGAAGAAGGTGGAGAGAGAGACAGA
GACAGATCCATTCGATTAGTGAACGGATCTCGACGGTATCGGTTAACTTTTAAAAGAAAAGGGGGGATTGGGGGGTACA
GTGCAGGGGAAAGAATAGTAGACATAATAGCAACAGACATACAAACTAAAGAATTACAAAAACAAATTACAAAATTCAA
AATTTTATCGATACTAGTATTATGCCCAGTACATGACCTTATGGGACTTTCCTACTTGGCAGTACATCTACGTATTAGT
CATCGCTATTACCATGGAGTAATTCATACAAAAGGACTCGCCCCTGCCTTGGGGAATCCCAGGGACCGTCGTTAAACTC
CCACTAACGTAGAACCCAGAGATCGCTGCGTTCCCGCCCCCTCACCCGCCCGCTCTCGTCATCACTGAGGTGGAGAAGA
GCATGCGTGAGGCTCCGGTGCCCGTCAGTGGGCAGAGCGCACATCGCCCACAGTCCCCGAGAAGTTGGGGGGAGGGGTC
GGCAATTGAACCGGTGCCTAGAGAAGGTGGCGCGGGGTAAACTGGGAAAGTGATGTCGTGTACTGGCTCCGCCTTTTTC
CCGAGGGTGGGGGAGAACCGTATATAAGTGCAGTAGTCGCCGTGAACGTTCTTTTTCGCAACGGGTTTGCCGCCAGAAC
ACAGGTAAGTGCCGTGTGTGGTTCCCGCGGGCCTGGCCTCTTTACGGGTTATGGCCCTTGCGTGCCTTGAATTACTTCC
ACGCCCCTGGCTGCAGTACGTGATTCTTGATCCCGAGCTTCGGGTTGGAAGTGGGTGGGAGAGTTCGAGGCCTTGCGCT
TAAGGAGCCCCTTCGCCTCGTGCTTGAGTTGAGGCCTGGCTTGGGCGCTGGGGCCGCCGCGTGCGAATCTGGTGGCACC
TTCGCGCCTGTCTCGCTGCTTTCGATAAGTCTCTAGCCATTTAAAATTTTTGATGACCTGCTGCGACGCTTTTTTTCTG
GCAAGATAGTCTTGTAAATGCGGGCCAAGATCTGCACACTGGTATTTCGGTTTTTGGGGCCGCGGGCGGCGACGGGGCC
CGTGCGTCCCAGCGCACATGTTCGGCGAGGCGGGGCCTGCGAGCGCGGCCACCGAGAATCGGACGGGGGTAGTCTCAAG
CTGGCCGGCCTGCTCTGGTGCCTGGCCTCGCGCCGCCGTGTATCGCCCCGCCCTGGGCGGCAAGGCTGGCCCGGTCGGC
ACCAGTTGCGTGAGCGGAAAGATGGCCGCTTCCCGGCCCTGCTGCAGGGAGCTCAAAATGGAGGACGCGGCGCTCGGGA
GAGCGGGCGGGTGAGTCACCCACACAAAGGAAAAGGGCCTTTCCGTCCTCAGCCGTCGCTTCATGTGACTCCACGGAGT
ACCGGGCGCCGTCCAGGCACCTCGATTAGTTCTCGAGCTTTTGGAGTACGTCGTCTTTAGGTTGGGGGGAGGGGTTTTA
TGCGATGGAGTTTCCCCACACTGAGTGGGTGGAGACTGAAGTTAGGCCAGCTTGGCACTTGATGTAATTCTCCTTGGAA
TTTGCCCTTTTTGAGTTTGGATCTTGGTTCATTCTCAAGCCTCAGACAGTGGTTCAAAGTTTTTTTCTTCCATTTCAGG
TGTCGTGATTCGAAGCCGCCACCATGGCCCTCCCTGTCACCGCCCTGCTGCTTCCGCTGGCTCTTCTGCTCCACGCCGC
TCGGCCCGACATCCAGATGACCCAGACCACAAGCAGCCTGTCTGCCAGCCTGGGCGATAGAGTGACCATCAGCTGTAGA
GCCAGCCAGGACATCAGCAAGTACCTGAACTGGTATCAGCAGAAACCCGACGGCACCGTGAAGCTGCTGATCTACCACA
CCAGCAGACTGCACAGCGGCGTGCCAAGCAGATTTTCTGGCAGCGGCTCTGGCACCGACTACAGCCTGACAATCAGCAA
CCTGGAACAAGAGGATATCGCTACCTACTTCTGCCAGCAAGGCAACACCCTGCCTTACACCTTTGGCGGAGGCACCAAG
CTGGAAATCACCGGAGGCGGAGGATCTGGTGGCGGAGGAAGTGGCGGAGGCGGTTCTGAAGTGAAGCTGCAAGAGTCTG
GCCCTGGACTGGTGGCCCCATCTCAGTCTCTGAGCGTGACCTGTACAGTCAGCGGAGTGTCCCTGCCTGATTACGGCGT
GTCCTGGATCAGACAGCCTCCTCGGAAAGGCCTGGAATGGCTGGGAGTGATCTGGGGCAGCGAGACAACCTACTACAAC
AGCGCCCTGAAGTCCCGGCTGACCATCATCAAGGACAACAGCAAGAGCCAGGTGTTCCTGAAGATGAACAGCCTGCAGA
CCGACGACACCGCCATCTACTACTGCGCCAAGCACTACTACTACGGCGGCAGCTACGCCATGGATTATTGGGGCCAGGG
CACCAGCGTGACCGTTTCTTCTACCACGACGCCAGCGCCGCGACCACCAACACCGGCGCCCACCATCGCGTCGCAGCCC
CTGTCCCTGCGCCCAGAAGCGTGCCGGCCAGCGGCGGGGGGCGCAGTGCACACGAGGGGGCTGGACTTCGCCTGTGATT
TCTGGGTGCTGGTCGTTGTGGGCGGCGTGCTGGCCTGCTACAGCCTGCTGGTGACAGTGGCCTTCATCATCTTTTGGGT
GAGGAGCAAGCGGAGCAGACTGCTGCACAGCGACTACATGAACATGACCCCCCGGAGGCCTGGCCCCACCCGGAAGCAC
TACCAGCCCTACGCCCCTCCCAGGGATTTCGCCGCCTACCGGAGCAAACGGGGCAGAAAGAAACTCCTGTATATATTCA
AACAACCATTTATGAGACCAGTACAAACTACTCAAGAGGAAGATGGCTGTAGCTGCCGATTTCCAGAAGAAGAAGAAGG
AGGATGTGAACTGAGAGTGAAGTTCAGCAGGAGCGCAGACGCCCCCGCGTACAAGCAGGGCCAGAACCAGCTCTATAAC
GAGCTCAATCTAGGACGAAGAGAGGAGTACGATGTTTTGGACAAGAGACGTGGCCGGGACCCTGAGATGGGGGGAAAGC
CGAGAAGGAAGAACCCTCAGGAAGGCCTGTACAATGAACTGCAGAAAGATAAGATGGCGGAGGCCTACAGTGAGATTGG
GATGAAAGGCGAGCGCCGGAGGGGCAAGGGGCACGATGGCCTTTACCAGGGTCTCAGTACAGCCACCAAGGACACCTAC
GACGCCCTTCACATGCAGGCCCTGCCCCCTCGCTAATAATCTAGAAATCAACCTCTGGATTACAAAATTTGTGAAAGAT
TGACTGGTATTCTTAACTATGTTGCTCCTTTTACGCTATGTGGATACGCTGCTTTAATGCCTTTGTATCATGCTATTGC
TTCCCGTATGGCTTTCATTTTCTCCTCCTTGTATAAATCCTGGTTGCTGTCTCTTTATGAGGAGTTGTGGCCCGTTGTC
AGGCAACGTGGCGTGGTGTGCACTGTGTTTGCTGACGCAACCCCCACTGGTTGGGGCATTGCCACCACCTGTCAGCTCC
TTTCCGGGACTTTCGCTTTCCCCCTCCCTATTGCCACGGCGGAACTCATCGCCGCCTGCCTTGCCCGCTGCTGGACAGG
GGCTCGGCTGTTGGGCACTGACAATTCCGTGGTGTTGTCGGGGAAATCATCGTCCTTTCCTTGGCTGCTCGCCTGTGTT
GCCACCTGGATTCTGCGCGGGACGTCCTTCTGCTACGTCCCTTCGGCCCTCAATCCAGCGGACCTTCCTTCCCGCGGCC
TGCTGCCGGCTCTGCGGCCTCTTCCGCGTCTTCGCCTTCGCCCTCAGACGAGTCGGATCTCCCTTTGGGCCGCCTCCCC

GCCTGGTACCAATTCTACCGGGTAGGGGAGGCGCTTTTCCCAAGGCAGTCTGGAGCATGCGCTTTAGCAGCCCCGCTGG
GCACTTGGCGCTACACAAGTGGCCTCTGGCCTCGCACACATTCCACATCCACCGGTAGGCGCCAACCGGCTCCGTTCTT
TGGTGGCCCCTTCGCGCCACCTTCTACTCCTCCCCTAGTCAGGAAGTTCCCCCCCGCCCCGCAGCTCGCGTCGTGCAGG
ACGTGACAAATGGAAGTAGCACGTCTCACTAGTCTCGTGCAGATGGACAGCACCGCTGAGCAATGGAAGCGGGTAGGCC
TTTGGGGCAGCGGCCAATAGCAGCTTTGCTCCTTCGCTTTCTGGGCTCAGAGGCTGGGAAGGGGTGGGTCCGGGGGCGG
GCTCAGGGGCGGGCTCAGGGGCGGGGCGGGCGCCCGAAGGTCCTCCGGAGGCCCGGCATTCTGCACGCTTCAAAAGCGC
ACGTCTGCCGCGCTGTTCTCCTCTTCCTCATCTCCGGGCCTTTCGACCTGCAGCCCAAGCTTGCCACCATGGTGAGCAA
GGGCGAGGAGCTGTTCACCGGGGTGGTGCCCATCCTGGTCGAGCTGGACGGCGACGTAAACGGCCACAAGTTCAGCGTG
TCCGGCGAGGGCGAGGGCGATGCCACCTACGGCAAGCTGACCCTGAAGTTCATCTGCACCACCGGCAAGCTGCCCGTGC
CCTGGCCCACCCTCGTGACCACCCTGACCTACGGCGTGCAGTGCTTCAGCCGCTACCCCGACCACATGAAGCAGCACGA
CTTCTTCAAGTCCGCCATGCCCGAAGGCTACGTCCAGGAGCGCACCATCTTCTTCAAGGACGACGGCAACTACAAGACC
CGCGCCGAGGTGAAGTTCGAGGGCGACACCCTGGTGAACCGCATCGAGCTGAAGGGCATCGACTTCAAGGAGGACGGCA
ACATCCTGGGGCACAAGCTGGAGTACAACTACAACAGCCACAACGTCTATATCATGGCCGACAAGCAGAAGAACGGCAT
CAAGGTGAACTTCAAGATCCGCCACAACATCGAGGACGGCAGCGTGCAGCTCGCCGACCACTACCAGCAGAACACCCCC
ATCGGCGACGGCCCCGTGCTGCTGCCCGACAACCACTACCTGAGCACCCAGTCCGCCCTGAGCAAAGACCCCAACGAGA
AGCGCGATCACATGGTCCTGCTGGAGTTCGTGACCGCCGCCGGGATCACTCTCGGCATGGACGAGCTGTACAAGGGTTC
TGGAGCCACGAACTTCTCTCTGTTAAAGCAAGCAGGAGACGTGGAAGAAAACCCCGGTCCTATGGCCAAGTTGACCAGT
GCCGTTCCGGTGCTCACCGCGCGCGACGTCGCCGGAGCGGTCGAGTTCTGGACCGACCGGCTCGGGTTCTCCCGGGACT
TCGTGGAGGACGACTTCGCCGGTGTGGTCCGGGACGACGTGACCCTGTTCATCAGCGCGGTCCAGGACCAGGTGGTGCC
GGACAACACCCTGGCCTGGGTGTGGGTGCGCGGCCTGGACGAGCTGTACGCCGAGTGGTCGGAGGTCGTGTCCACGAAC
TTCCGGGACGCCTCCGGGCCGGCCATGACCGAGATCGGCGAGCAGCCGTGGGGGCGGGAGTTCGCCCTGCGCGACCCGG
CCGGCAACTGCGTGCACTTTGTGGCAGAGGAGCAGGACTGATAATTTAAGACCAATGACTTACAAGGCAGCTGTAGATC
TTAGCCACTTTTTAAAAGAAAAGGGGGGACTGGAAGGGCTAATTCACTCCCAACGAAGATAAGATCTGCTTTTTGCTTG
TACTGGGTCTCTCTGGTTAGACCAGATCTGAGCCTGGGAGCTCTCTGGCTAACTAGGGAACCCACTGCTTAAGCCTCAA
TAAAGCTTGCCTTGAGTGCTTCAAGTAGTGTGTGCCCGTCTGTTGTGTGACTCTGGTAACTAGAGATCCCTCAGACCCT
TTTAGTCAGTGTGGAAAATCTCTAGCA

Table S1: Support for the putative SNVs or deletion in PacBio and Illumina reads

The observed fraction of PacBio and Illumina reads supporting each variant is generally consistent with the expected fraction based on the fraction of insertions in the respective VCN clone containing the variant. For example, in VCN2, each SNV is in one of two integrations in the clone, so we would expect approximately 50 % of reads for that integration site to contain a SNV at that position. SNVs in both VCN4 and VCN5 are based on expected ALT read support for five integrations (VCN5). The VCN4/5 deletion at 5579 occurs in a region covered by a partial integration (so that the denominator is 6 total integrations). Differences from expectation may reflect limited read depth, mapping ambiguity, and platform-specific error profiles. SNVs all came from reads with supplementary alignments to the expected chromosomes. For the deletion at 5579, the expected percent support for ALT is based on VCN5 (as VCN4 was not sequenced with PacBio); the 2 non-chr6 reads supporting this deletion originated from insertions on chr1 and chr5, likely due to the known higher indel error rate in PacBio. Overall, the concordance between expected and observed read evidence provides support for variants inside integrated sequences.

**VCN clone**	**VCN2**	**VCN2**	**VCN2**	**VCN2**	**VCN3**	**VCN4/5**	**VCN4/5**	**VCN4/5**
**position in expected proviral sequence**	**1692**	**1693**	**4878**	**6601**	**1450**	**1066**	**3649**	**5579**
chromosome of integrated sequence with SNV or DEL in pbsv call	chr19	chr19	chr19	chr19	chr1	chr1	chr3	chr1/5/6
REF	A	C	G	G	T	G	G	C
ALT	T	T	C	A	A	A	A	DEL
**PACBIO - PBMM2**								
read support for ALT	10	10	7	6	12	7	7	1/1/4
total reads	16	16	10	10	32	42	38	32
**expected % support for ALT**	**50**	**50**	**50**	**50**	**33**	**20**	**20**	**17**
**observed % support for ALT**	**63**	**63**	**70**	**60**	**38**	**17**	**18**	**19**
**ILLUMINA - BWA**								
read support for ALT	8	8	15	17	20	4	14	6
total reads	28	28	35	34	54	44	47	58
**expected % support for ALT**	**50**	**50**	**50**	**50**	**33**	**20**	**20**	**17**
**observed % support for ALT**	**29**	**29**	**43**	**50**	**37**	**9**	**30**	**10**

Table S2: Summary of integration sites for each VCN clone

Table shows summary of all integration sites by VCN clone. The IS_ID corresponds to the chromosome integration site; IS_IDs can be shared across VCN clones. Integration positions on the chromosome (start/end) are the first and last base of the integration site, they are not left aligned. Positions of abnormalities are relative to the expected proviral sequence, not the position within the sequence reported in Supplemental Text. All integrations should begin with the 4 - 5 bp integration site sequence. This sequence is duplicated upon integration which can be seen as increased coverage in IGV. Homology between integration site and proviral sequence was observed as increased coverage making integration site sequences appear longer.

VCN	alignments performed	mosdepth median autosome coverage (BWA - pbmm2)	IS_ID	chromosome	start	end	integration site sequence	orientation	Integrated sequence length	integrated sequence fasta header name (Supplemental Text)
VCN0	ILMN (BWA)	28.5x - NA	NA	NA	NA	NA	NA	NA	0	expected_full_provirus_sequence_7374bp
VCN1	ILMN (BWA)	27x - NA	IS1	chr17	78,730,345	78,730,349	GAAAC	-	6655	IS1_VCN1-VCN2-VCN3-VCN4-VCN5_chr17:78730345
VCN2	HiFi (pbmm2), ILMN (BWA), pbsvINS (pbmm2)	28.7x - 44.4x	IS1	chr17	78,730,345	78,730,349	GAAAC	-	6655	IS1_VCN1-VCN2-VCN3-VCN4-VCN5_chr17:78730345
VCN2	HiFi (pbmm2), ILMN (BWA), pbsvINS (pbmm2)	28.7x - 44.4x	IS2	chr19	21,610,872	21,610,876	CCCCC	-	7379	IS2_VCN2_chr19:21610872
VCN3	HiFi (pbmm2), ILMN (BWA), pbsvINS (pbmm2)	27.2x - 37.9x	IS3	chr1	23,307,863	23,307,867	ACAGG	-	8183	IS3_VCN3_chr1:23307863
VCN3	HiFi (pbmm2), ILMN (BWA), pbsvINS (pbmm2)	27.2x - 37.9x	IS4	chr8	99,830,407	99,830,410	AAAC	-	263	IS4_VCN3_chr8:99830407
VCN3	HiFi (pbmm2), ILMN (BWA), pbsvINS (pbmm2)	27.2x - 37.9x	IS1	chr17	78,730,345	78,730,349	GAAAC	-	6655	IS1_VCN1-VCN2-VCN3-VCN4-VCN5_chr17:78730345
VCN3	HiFi (pbmm2), ILMN (BWA), pbsvINS (pbmm2)	27.2x - 37.9x	IS5	chrX	103,930,456	103,930,460	AATAC	-	7379	IS5_VCN3_chrX:103930456
VCN4	ILMN (BWA)	25.3x - NA	IS6	chr1	243,273,502	243,273,506	ACCCC	+	7420	IS6_VCN4-VCN5_chr1:243273502
VCN4	ILMN (BWA)	25.3x - NA	IS7	chr3	18,115,277	18,115,281	TACTC	-	7379	IS7_VCN4-VCN5_chr3:18115277
VCN4	ILMN (BWA)	25.3x - NA	IS8	chr6	15,601,040	15,601,062	possibly AG	-	2070	IS8_VCN4-VCN5_chr6:15601040
VCN4	ILMN (BWA)	25.3x - NA	IS9	chr6	24,825,065	24,825,069	GTACT	+	7379	IS9_VCN4-VCN5_chr6:24825065
VCN4	ILMN (BWA)	25.3x - NA	IS1	chr17	78,730,345	78,730,349	GAAAC	-	6655	IS1_VCN1-VCN2-VCN3-VCN4-VCN5_chr17:78730345

VCN5	HiFi (pbmm2), ILMN (BWA), pbsvINS (pbmm2)	24.7x - 31.3x	IS6	chr1	243,273,502	243,273,506	ACCCC	+	7420	IS6_VCN4-VCN5_chr1:243273502
VCN5	HiFi (pbmm2), ILMN (BWA), pbsvINS (pbmm2)	24.7x - 31.3x	IS7	chr3	18,115,277	18,115,281	TACTC	-	7379	IS7_VCN4-VCN5_chr3:18115277
VCN5	HiFi (pbmm2), ILMN (BWA), pbsvINS (pbmm2)	24.7x - 31.3x	IS10	chr5	39,044,696	39,044,700	TTCAC	-	7379	IS10_VCN5_chr5:39044696
VCN5	HiFi (pbmm2), ILMN (BWA), pbsvINS (pbmm2)	24.7x - 31.3x	IS8	chr6	15,601,040	15,601,062	possibly AG	-	2070	IS8_VCN4-VCN5_chr6:15601040
VCN5	HiFi (pbmm2), ILMN (BWA), pbsvINS (pbmm2)	24.7x - 31.3x	IS9	chr6	24,825,065	24,825,069	GTACT	+	7379	IS9_VCN4-VCN5_chr6:24825065
VCN5	HiFi (pbmm2), ILMN (BWA), pbsvINS (pbmm2)	24.7x - 31.3x	IS1	chr17	78,730,345	78,730,349	GAAAC	-	6655	IS1_VCN1-VCN2-VCN3-VCN4-VCN5_chr17:78730345

Table S3: Summary of integration site observations

This table builds upon the genomic loci identified in Table S2 to describe the precise composition and sequence integrity of each specific integration event. It includes the VCN clone identifier and the unique integration site identifier (IS_ID), allowing for direct cross-referencing with the genomic coordinates and sequencing metrics in Table S2. The table characterizes integration site , specifically noting the expected length. Furthermore, it provides a high-level categorization of the proviral integration state—such as full-length integration, incomplete integration, or integration with structural abnormality— alongside detailed descriptions of any sequence variations.

VCN	IS_ID	integration site observations	integration integrity	integrity notes
VCN0	NA	NA	NA	control, jurkat genome with no integrations
VCN1	IS1	5 bp as expected	integration with structural abnormality	Structural abnormality with 724 bp deletion of expected sequence, inferred from HiFi sequencing of VCN2, VCN3 and VCN5. VCN2-5 derives from VCN1.
VCN2	IS1	5 bp as expected	integration with structural abnormality	Large 724 bp deletion in middle of the integrated sequence (bases 1917-2640 of expected sequence) in the EF1α promoter region.
VCN2	IS2	5 bp as expected	full length integration	Four SNVs identified in integrated sequence at expected sequence positions 1692: A -> T, 1693: C -> T, 4878: G -> C and 6601: G -> A. Other than SNVs, no other abnormalities.
VCN3	IS3	5 bp as expected	integration with structural abnormality	Structural abnormality at the 3' end of a full-length integration. The abnormality contains additional sequence composed of a large duplication, corresponding to expected integration sequence positions 4416 - 5193 (778 bp), followed by 2bp (GT) then 24 bps of the 3' LTR sequence (AGTCAGTGTGGAAAATCTCTAGCA). Within the full-length portion of this integration, there is a SNV T->A at position 1450.
VCN3	IS4	4 bp integration site sequence	incomplete integration	A short 263 bp integrated sequence primarily composed of LTR sequence was identified. The sequence begins with a four base-pair duplication of the chr8 integration site. Following this duplication, 159 bp correspond to LTR positions 7216–7374 of the expected integration. The remaining 100 bp are less clearly defined but appear to include a mixture of unknown and LTR-derived sequence (33 bp unknown, 21 bp possible LTR, and 46 bp unknown using BLAST). BLAST of the 79 bp of "unknown" sequence was performed and no significant similarities were found.
VCN3	IS1	5 bp as expected	integration with structural abnormality	Large 724 bp deletion in middle of the integrated sequence (bases 1917-2640 of expected sequence) in the EF1α promoter region.
VCN3	IS5	5 bp as expected	full length integration	none
VCN4	IS6	**ACCCC**T: homology ("T") between chromosome and 1st base of integrated sequence. This resulted in "T" appearing duplicated in coverage plot at integration site, but it is not part of the duplicated integration site sequence.	integration with structural abnormality	Structural abnormality inferred from VCN5 HiFi sequencing. VCN5 derives from VCN4.
VCN4	IS7	**TACTC**T: homology ("T") between chromosome and 1st base of integrated sequence. This resulted in "T" appearing duplicated in coverage plot at integration site, but it is not part of the duplicated integration site sequence.	full length integration	Full length integration with SNV G->A at integrated sequence position 3649 inferred from VCN5 HiFi sequencing. VCN5 was derived from VCN4. There is some evidence for SNV in VCN4 ILMN reads.
VCN4	IS8	integration site sequence is unclear	incomplete integration	Incomplete integration inferred from VCN5 HiFi sequencing. VCN5 derives from VCN4.
VCN4	IS9	**GTACT**T: homology ("T") between chromosome and 1st base of integrated sequence. This resulted in "T" appearing duplicated in coverage plot at integration site, but it is not part of the duplicated integration site sequence.	full length integration	full length integration inferred from VCN5, which was sequenced with HiFi. VCN5 derives from VCN4.
VCN4	IS1	5 bp as expected	integration with structural abnormality	Structural abnormality with 724 bp deletion of expected sequence inferred from HiFi sequencing of VCN2, VCN3 and VCN5. VCN4 derives from VCN1, where IS1 originated. VCN4 was then used to generate VCN5.
VCN5	IS6	**ACCCC**T: homology ("T") between chromosome and 1st base of integrated sequence. This resulted in "T" appearing	integration with structural abnormality	SNV G->A at position 1066 in full integrated sequence. End of full integrated sequence has an additional 41 bp. This sequence is somewhat unclear and but appears to be

		duplicated in coverage plot at integration site, but it is not part of the duplicated integration site sequence.		comprised of 15 bases of unknown origin followed by the first 26bp of the LTR sequence in reverse orientation.
VCN5	IS7	**TACTC**T: homology ("T") between chromosome and 1st base of integrated sequence. This resulted in "T" appearing duplicated in coverage plot at integration site, but it is not part of the duplicated integration site sequence.	full length integration	SNV G->A at full integrated sequence position 3649. Other than SNV, no other abnormalities.
VCN5	IS10	CA**TTCAC:** homology ("CA") between chromosome and last two bases of integrated sequence. This resulted in "CA" appearing duplicated in coverage plot at integration site, but it is not part of the duplicated integration site sequence.	full length integration	none
VCN5	IS8	integration site sequence is unclear	incomplete integration	Integrated sequence is incomplete (2070 bp) and only contains bases corresponding to positions 5306 - 7374 of the expected integration sequence. It appears that 22 bases of chr6 were deleted at the same time as the insertion, from chr6:15,601,040 - 15,601,062. This 22bp region on chr6 has reduced coverage, likely as part of deletion, and numerous SNVs relative to chr6 due to misalignment of the integrated sequence. The pbsv call appears to be 24bp shorter compared with aligned read (clipped sequence) due to imperfect alignments to the deleted region. 22/24 bases, "CGAAGACGCGGAAGAGGCCGCA", align to the expected integration sequence, but the final two bases, "AG", do not. These two bases are part of chr6 sequence adjacent to deleted portion of chr6. It is possible these two bases are duplicated upon integration. There is also a deletion of a "C" at position 5579 relative to the expected sequence; this "C" is part of a string of Cs.
VCN5	IS9	**GTACT**T: homology ("T") between chromosome and 1st base of integrated sequence. This resulted in "T" appearing duplicated in coverage plot at integration site, but it is not part of the duplicated integration site sequence.	full length integration	none
VCN5	IS1	5 bp as expected	integration with structural abnormality	Large 724 bp deletion in middle of the integrated sequence (bases 1917-2640 of expected sequence) in the EF1α promoter region.

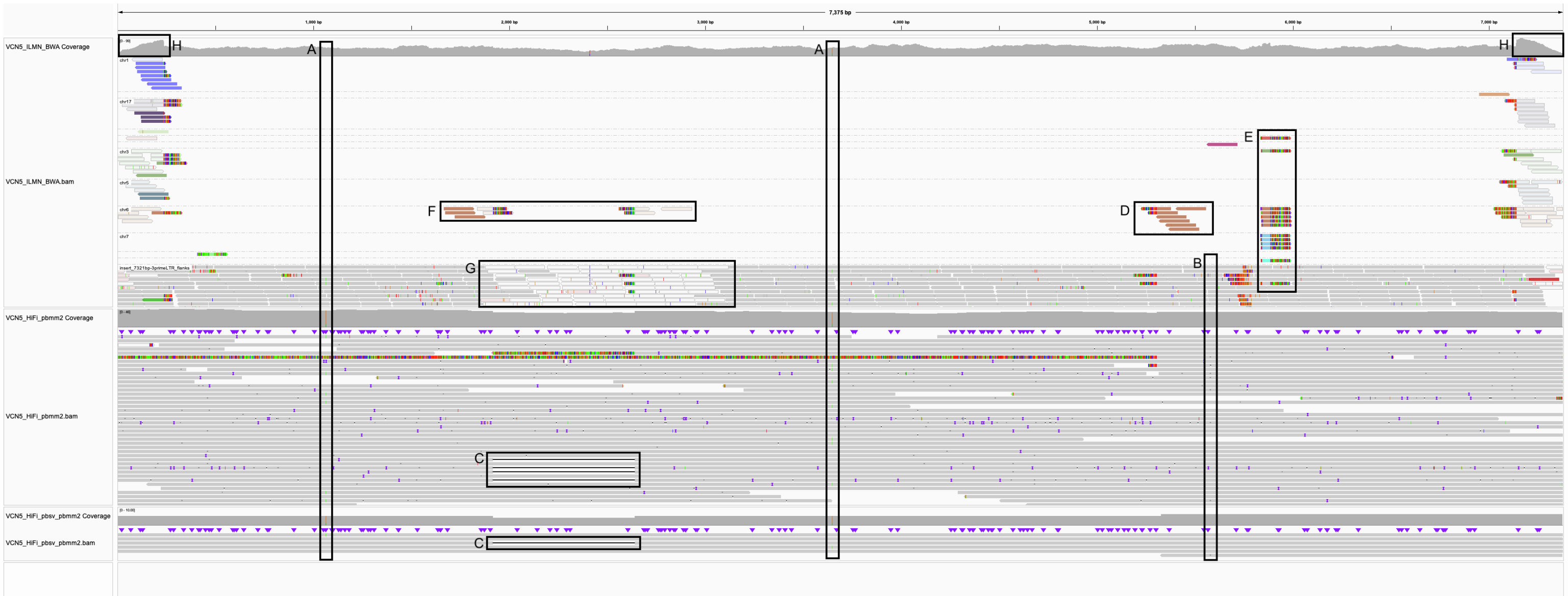

Figure S1: Alignments of Illumina (ILMN) reads, PacBio HiFi reads, and PacBio pbsv variant calls to the provirus sequence for VCN5

The Illumina track is grouped by ”chromosome of mate” to highlight integration sites. Visualization with IGV identified specific variants and mapping artifacts annotated as follows: A) SNVs observed on integrated sequence in chr1 and chr3. Note, IGV "coverage allele fraction threshold" = 0.15. B) DEL observed on integrated sequence in chr6. Given the extra insertion (IS8) in VCN5, it was expected that the DEL would be observed in 17% of reads. While not visible in the screen shot, 10 % (6/58) of Illumina reads contained the DEL. The DEL can be seen in HiFi reads and was observed in 19 % (6/32) of reads. With HiFi reads it was possible to see supplementary alignments of the reads supporting the deletion, with four correctly on chr6 but two from other chromosomes, likely due to indel errors in HiFi. C) 724 bp deletion of promoter in integration sequence on chr17. D) Partial insertion (2070 bp) on chr6. E) VCN5, but not other VCNs, have Illumina reads partially aligned to ~60 bp of provirus sequence, but this is not seen in HiFi reads in any VCN. F) Illumina read mates map incorrectly with MQ0 to the *EEF1A1* (chr6:73.49-73.53) gene promoter in GRCh38 because this human gene promoter was used in the provirus. This was observed with all VCN Illumina reads. G) Region has homology with *EEF1A1* gene in GRCh38, causing zero mapping quality in Illumina data but not long reads. This was observed with all VCN Illumina reads. H) Flanking LTR regions have high coverage of Illumina reads with low MQ because these sequences are identical.

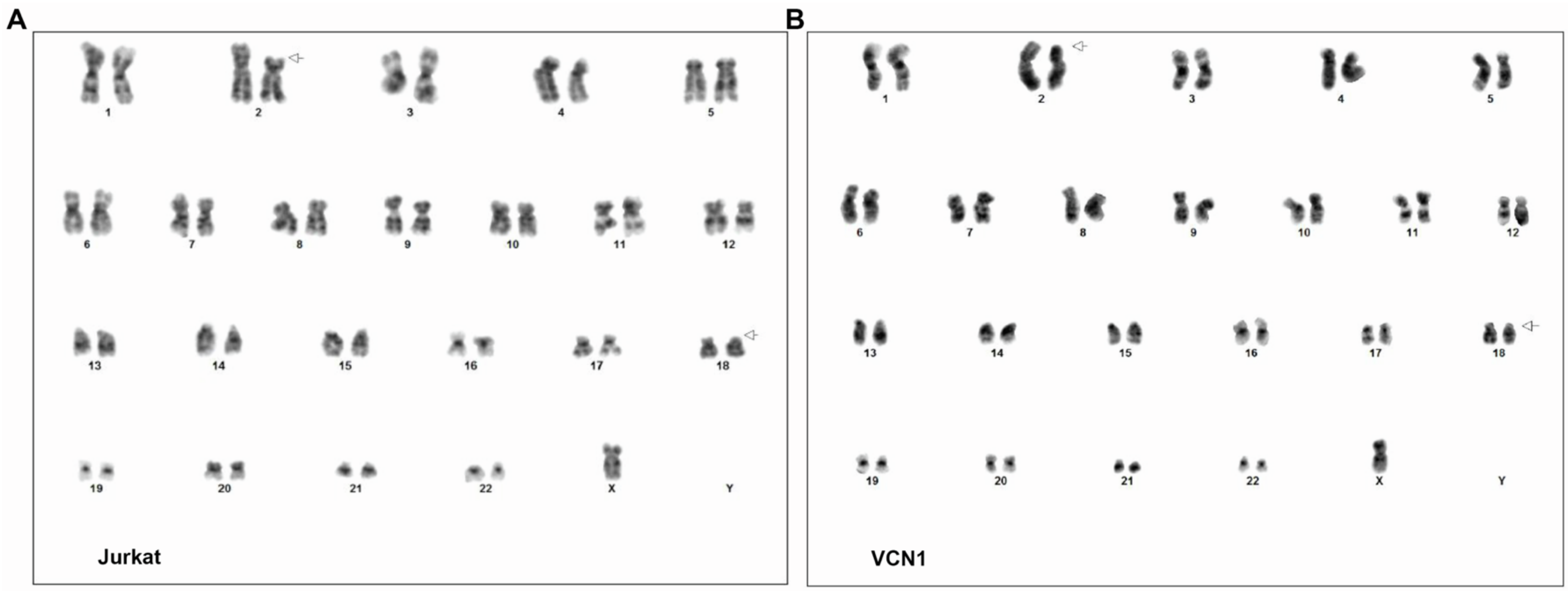

Figure S2: G-band Karyograms of Jurkat cells and clonal cell line
A subpopulation of Jurkat cells and the clonal cell line VCN1 lacked the Y chromosome. A) G-band karyotype analysis revealed that a subpopulation (7 out of 20 metaphase spreads) of Jurkat cells lacked the Y chromosome. B) All VCN1 metaphase spreads exhibited a lack of the Y chromosome

IS1_VCN1-VCN2-VCN3-VCN4-VCN5_chr17:78730345

PacBio HiFi

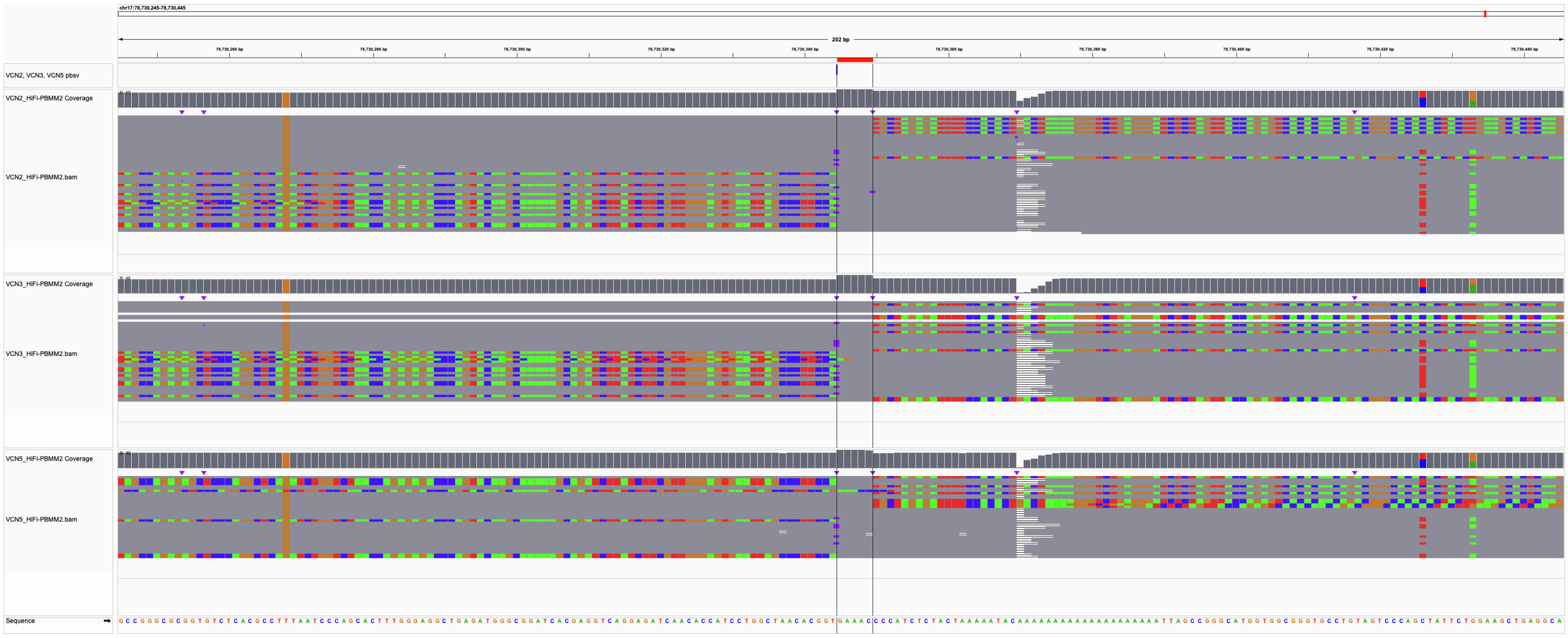

Illumina

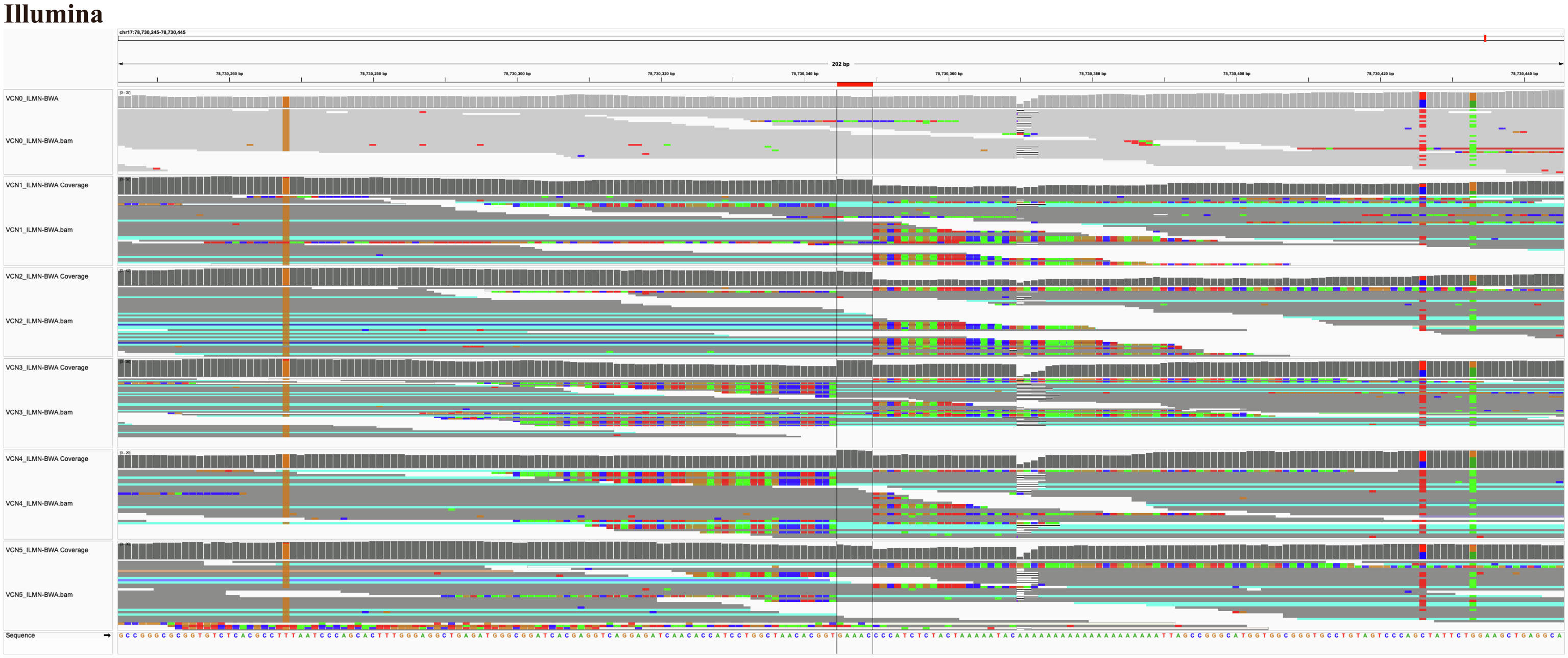

IS2_VCN2_chr19:21610872

PacBio HiFi

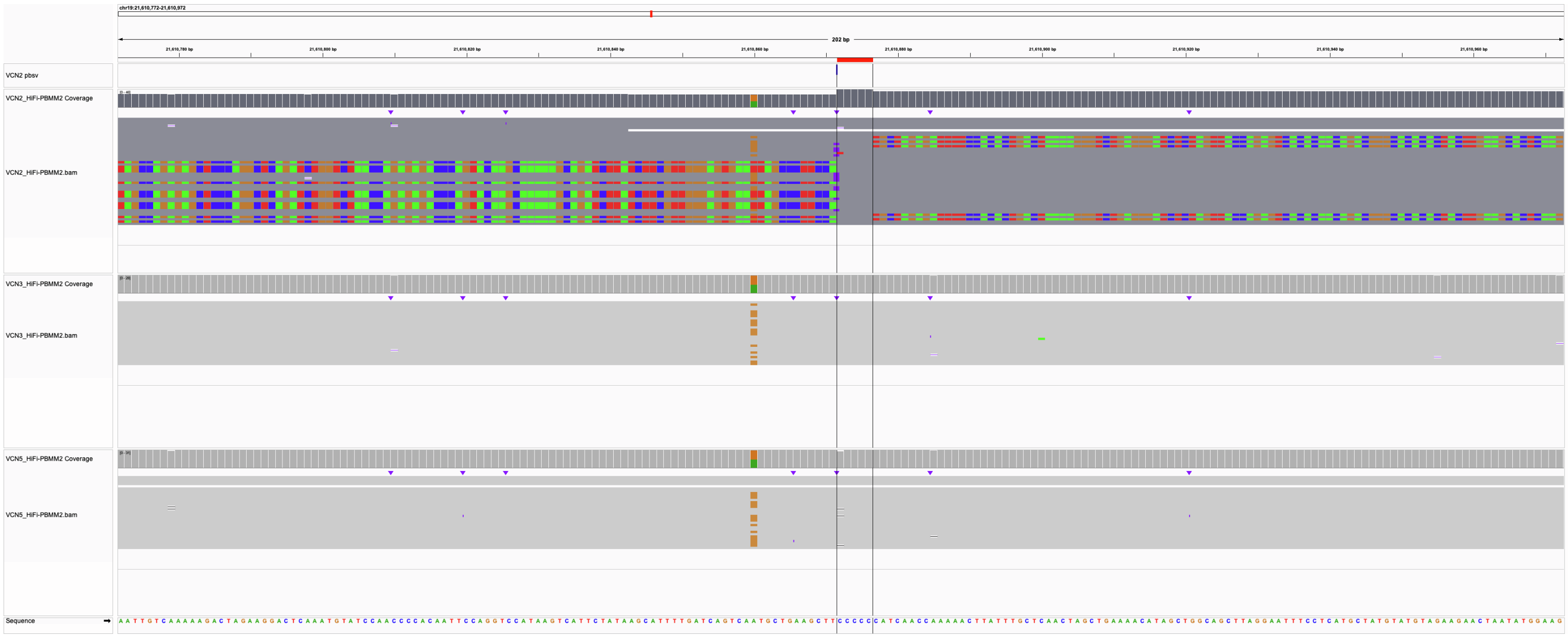

Illumina

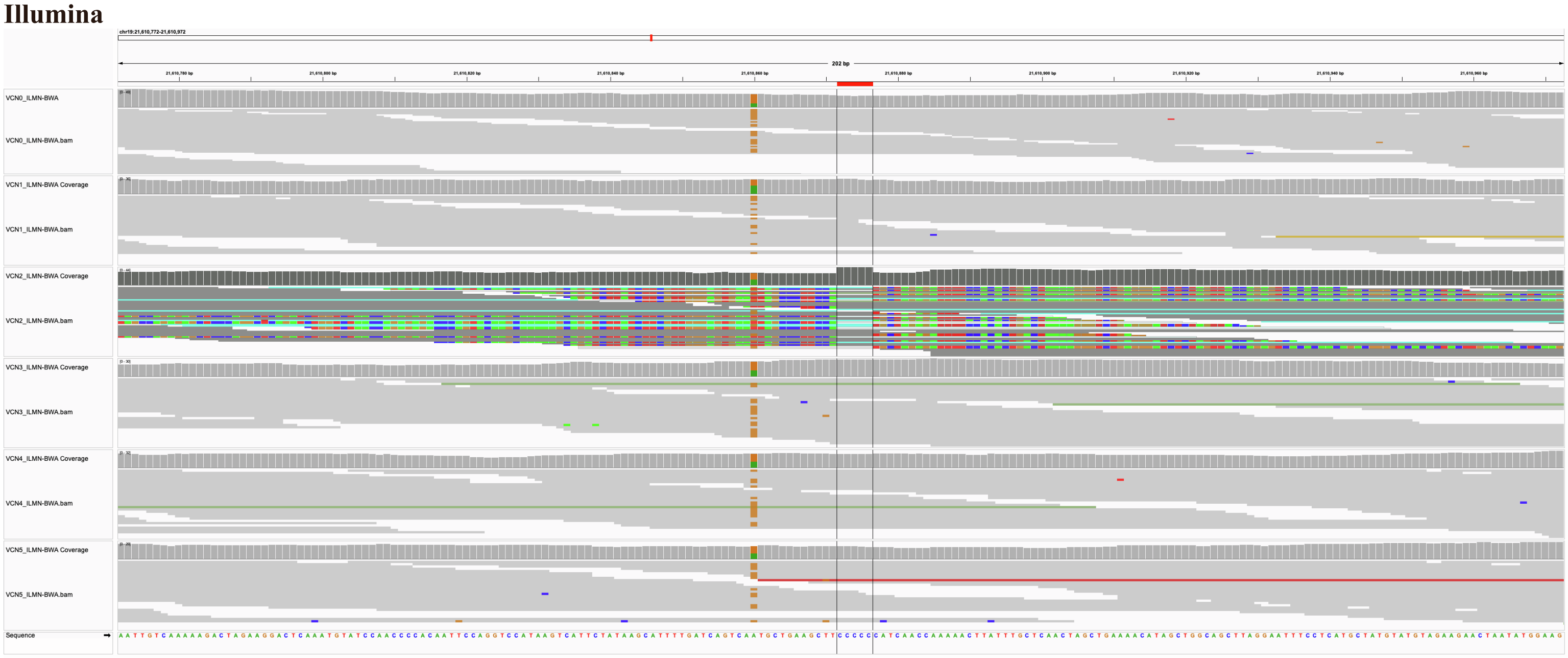

IS3_VCN3_chr1:23307863

HiFi

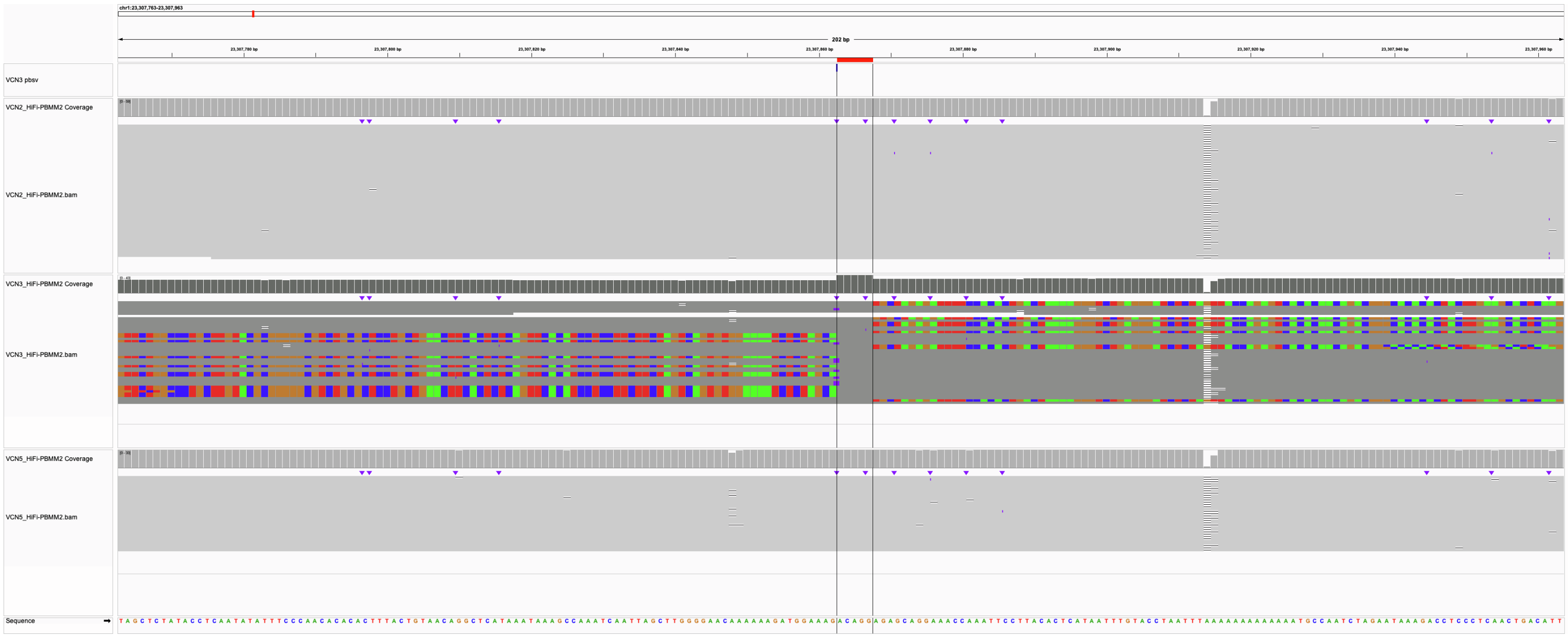

Illumina

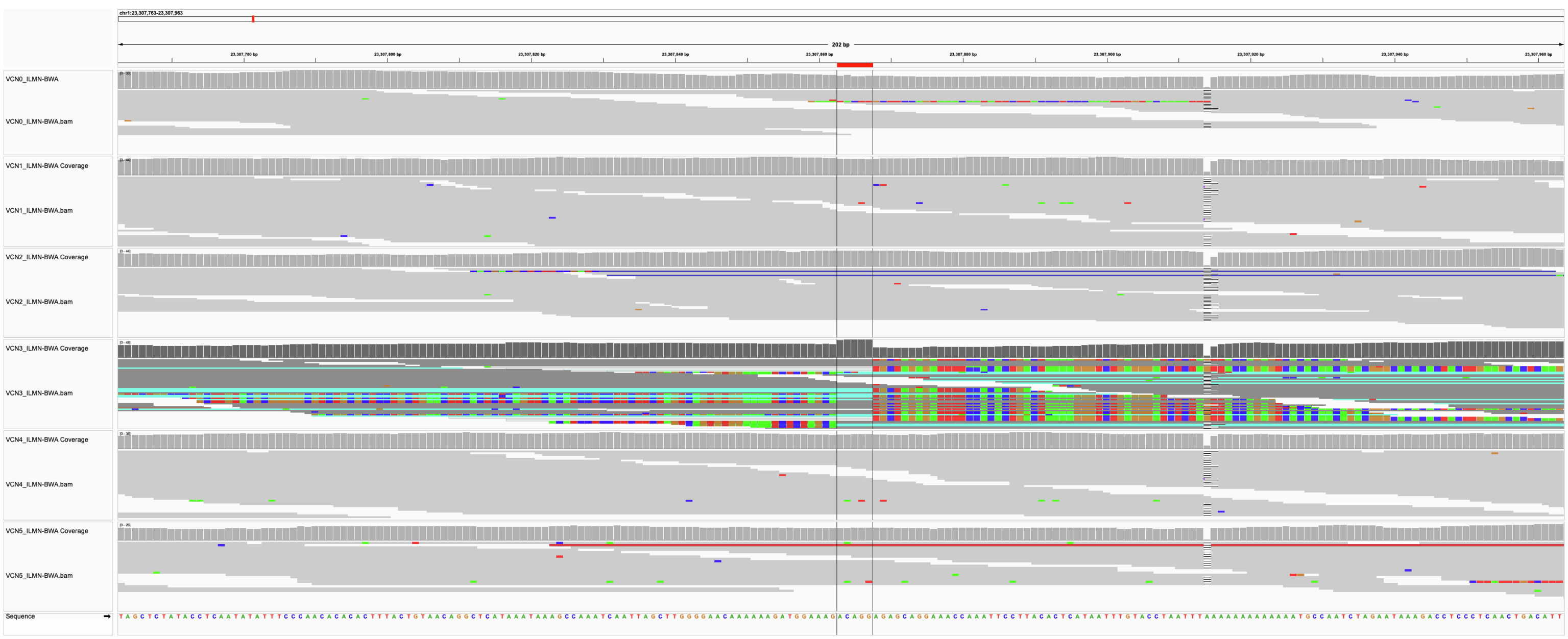

IS4_VCN3_chr8:99830407

HiFi

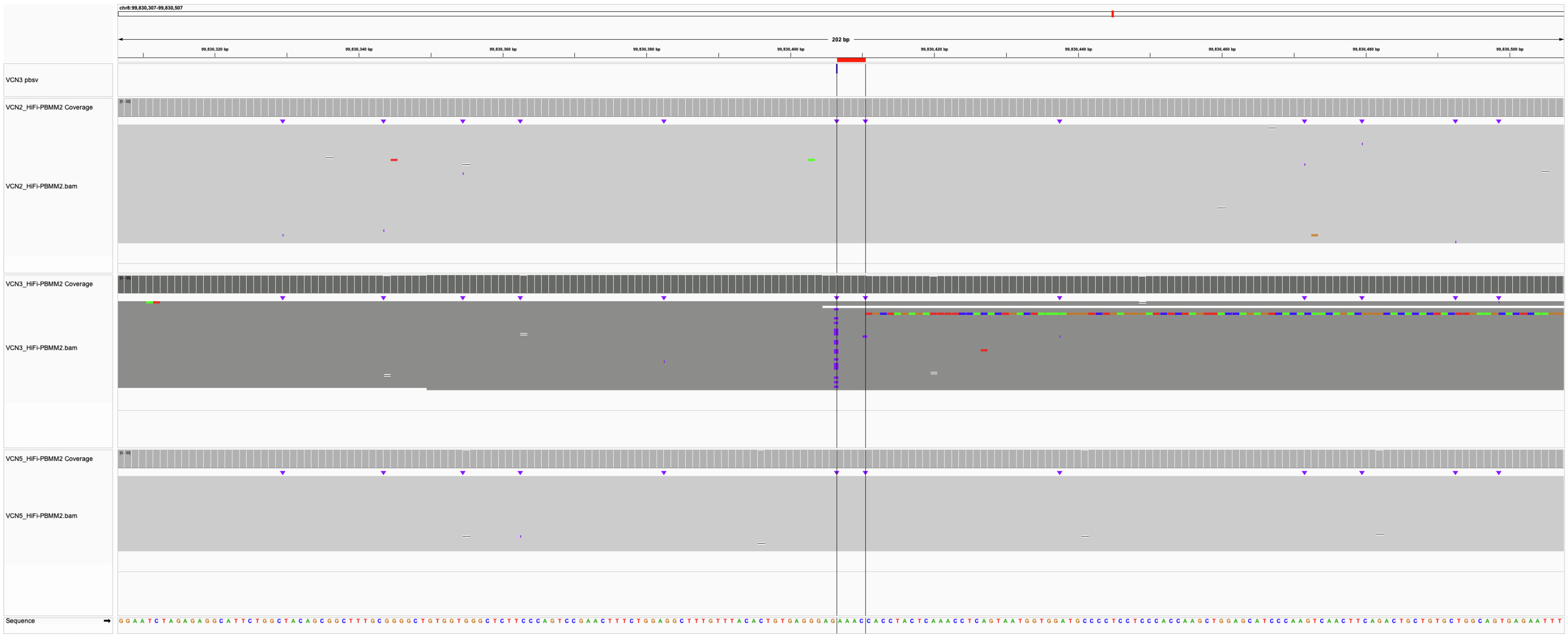

Illumina

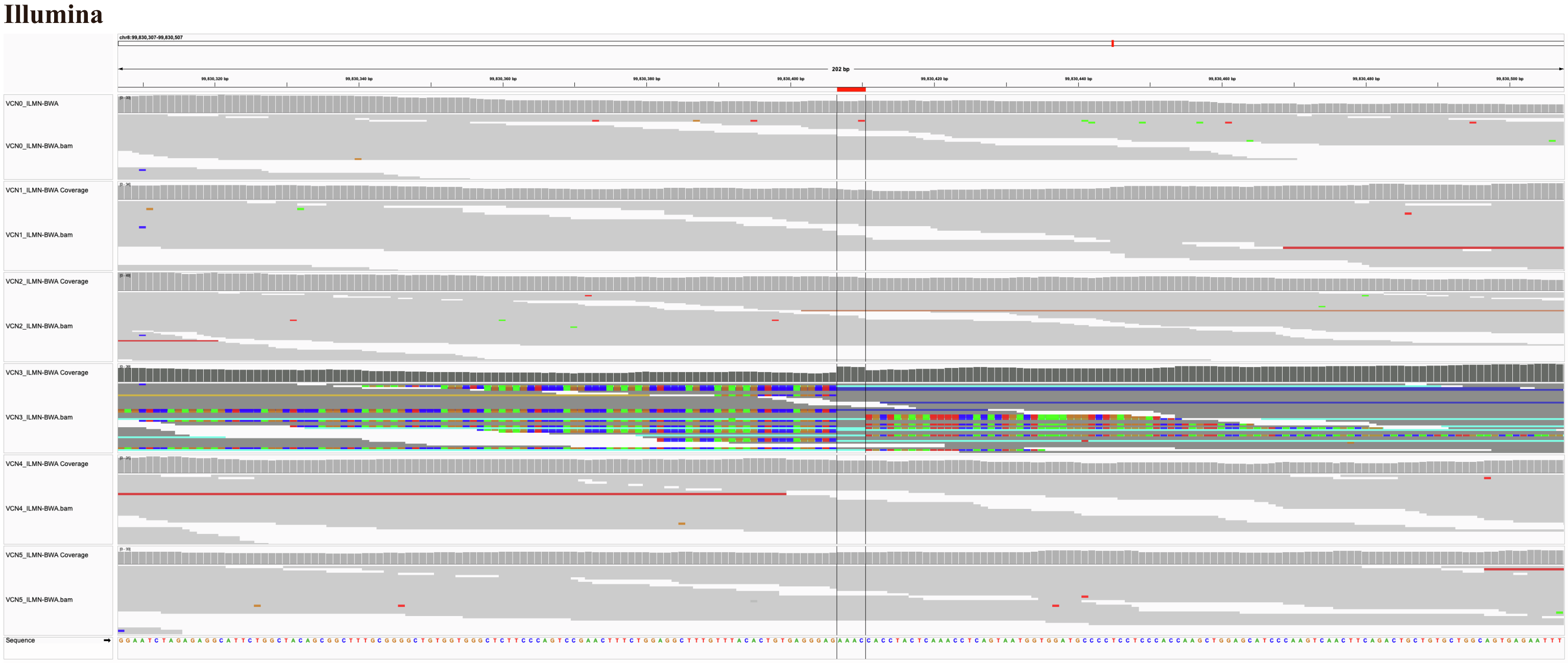

IS5_VCN3_chrX:103930456

HiFi

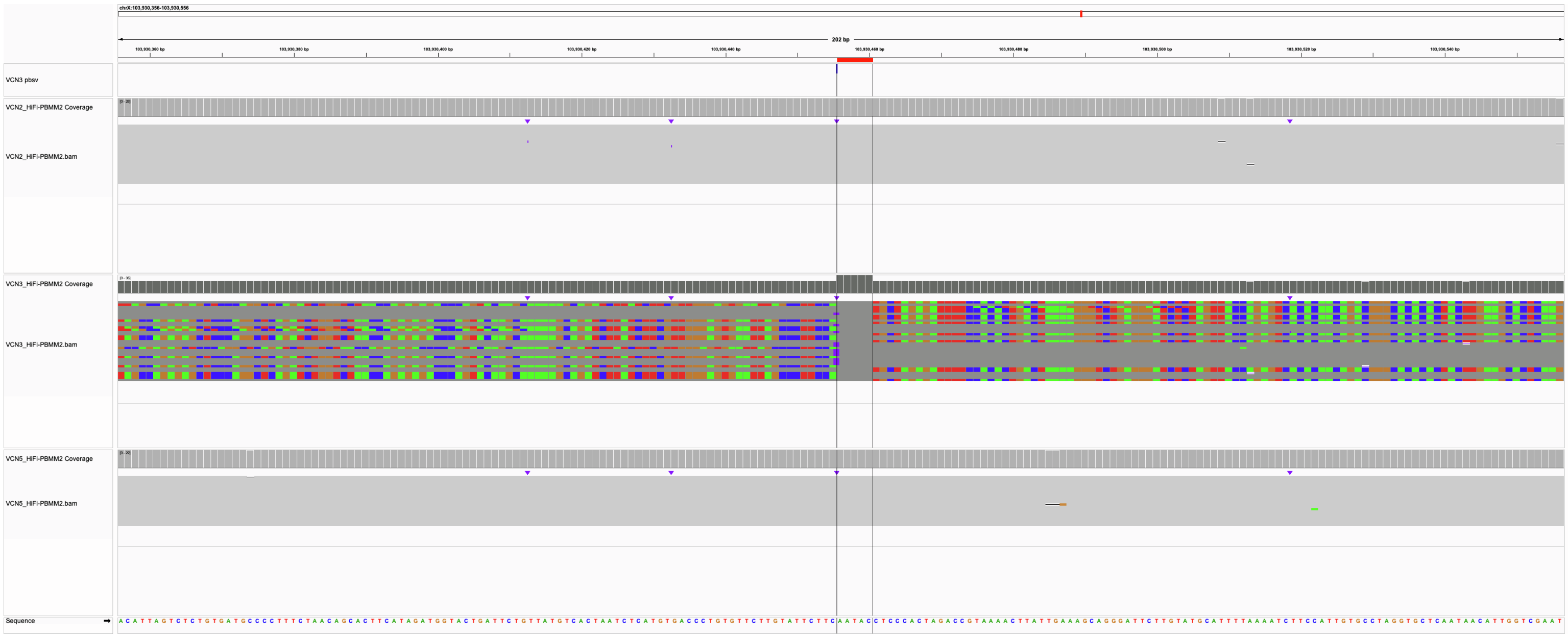

Illumina

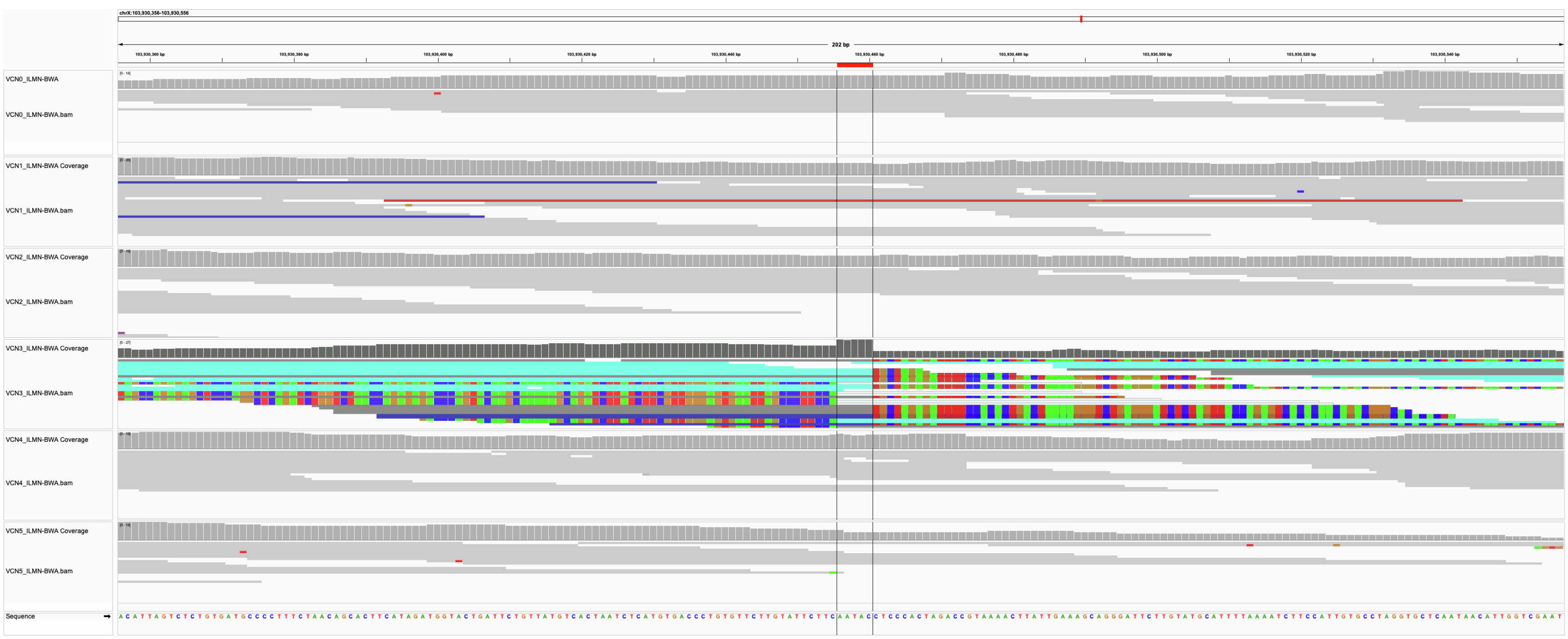

IS6_VCN4-VCN5_chr1:243273502

HiFi

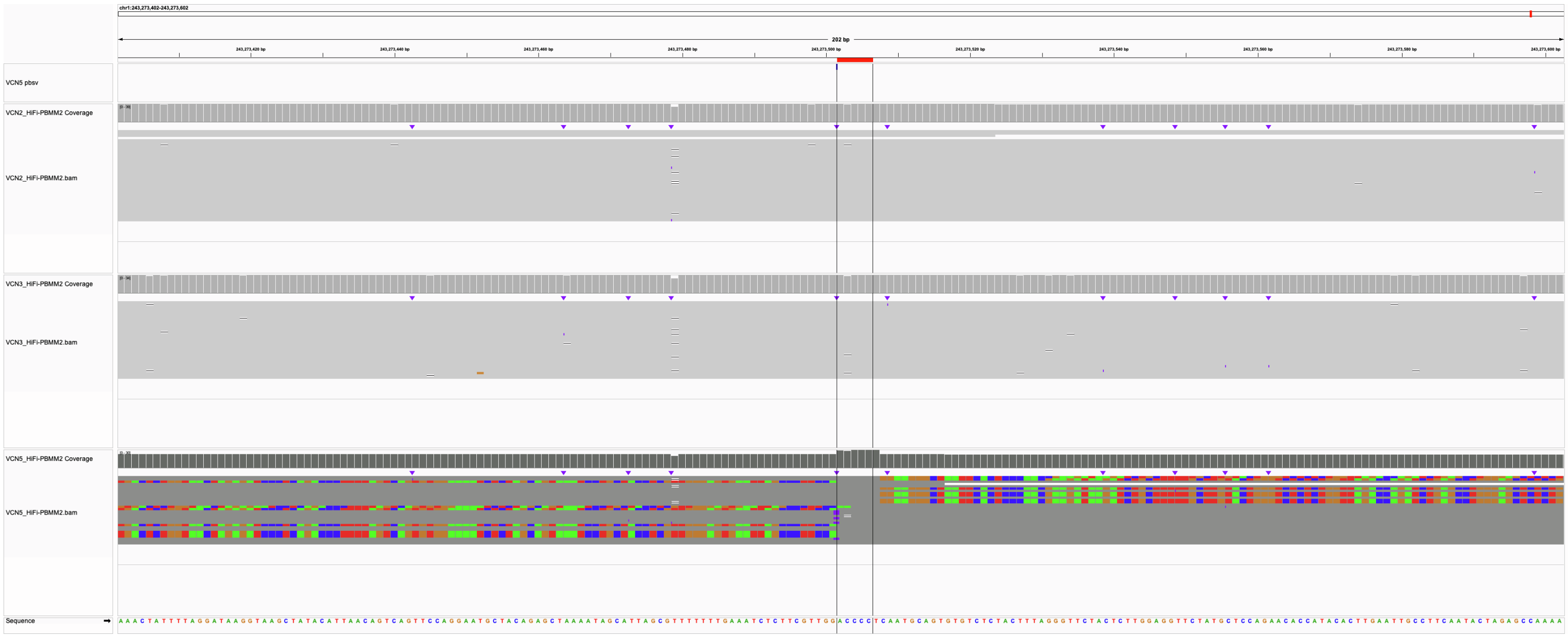

Illumina

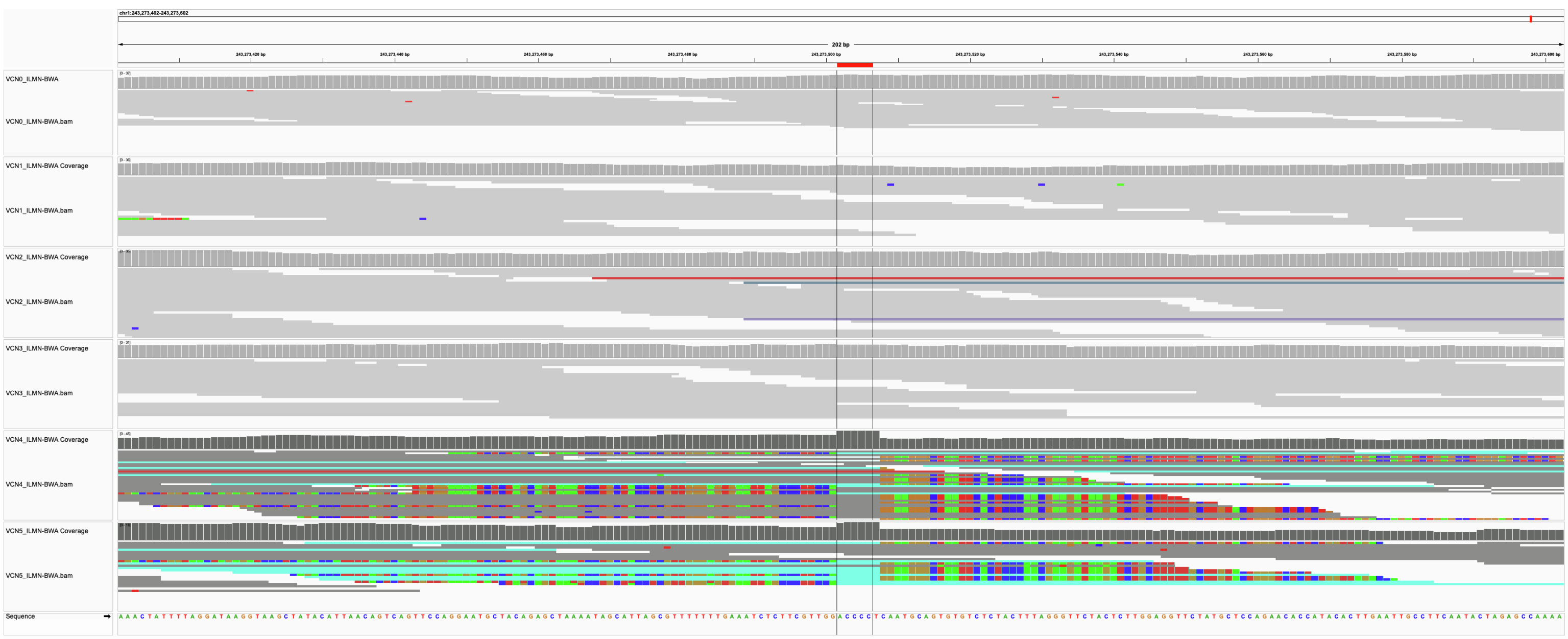

IS7_VCN4-VCN5_chr3:18115277

HiFi

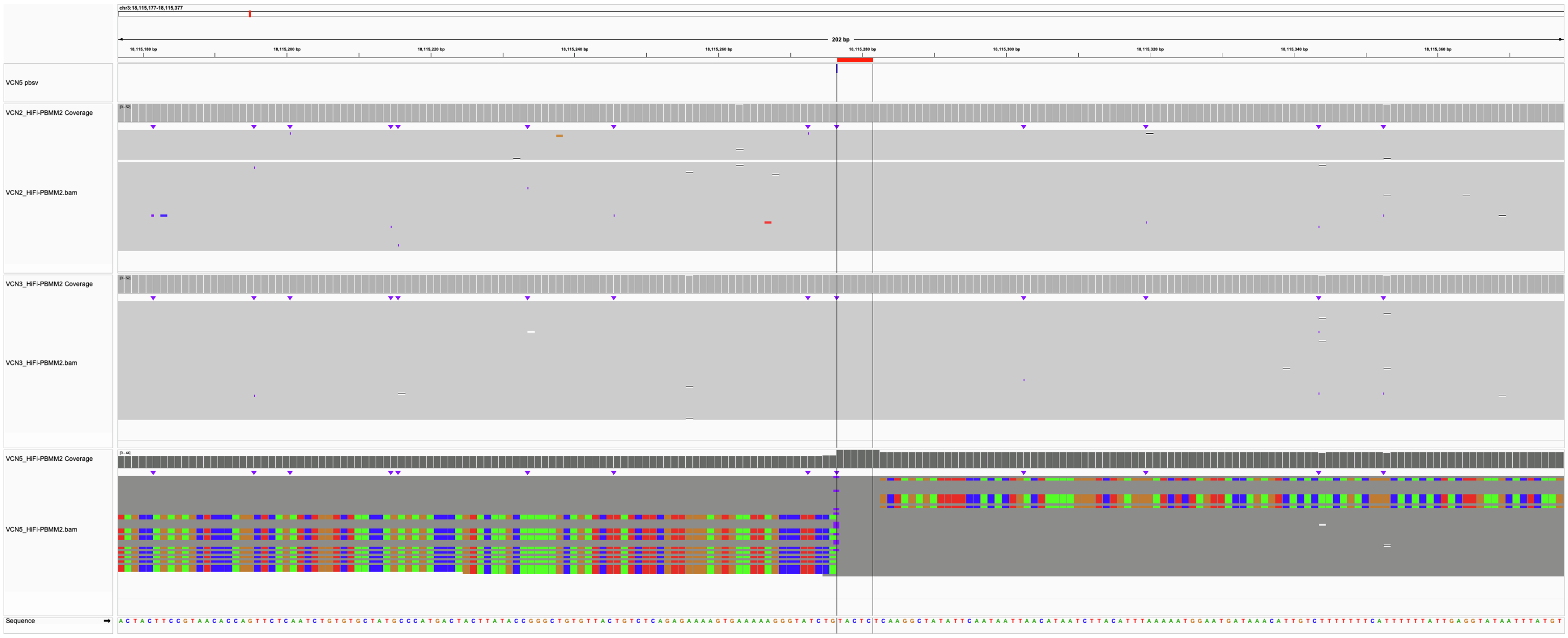

Illumina

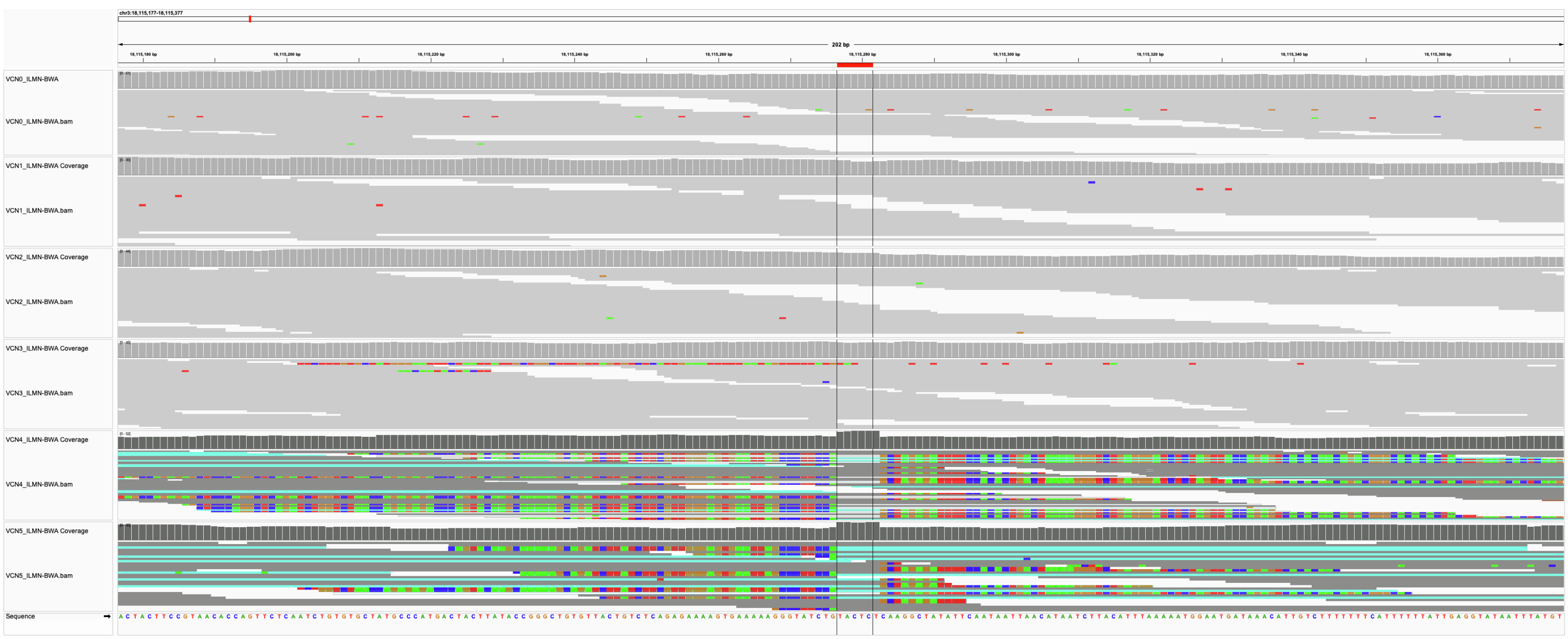

IS8_VCN4-VCN5_chr6:15601040

HiFi

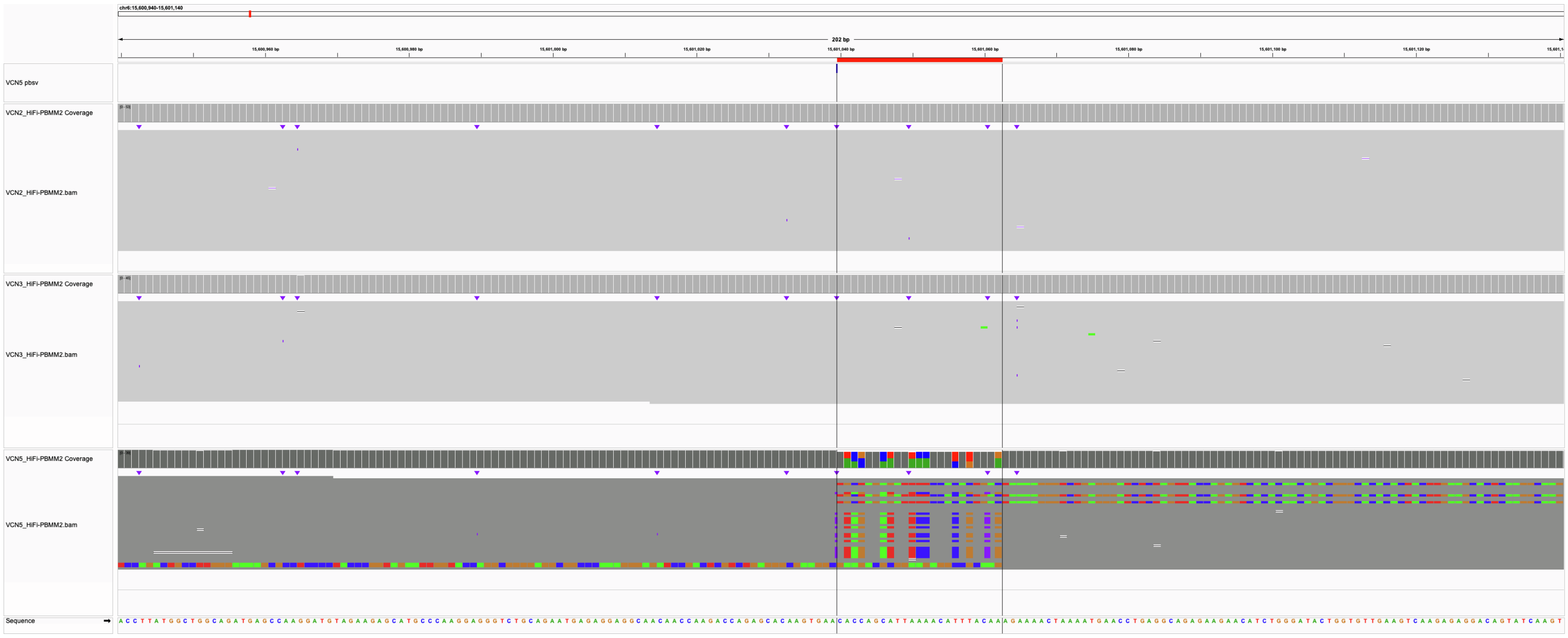

Illumina

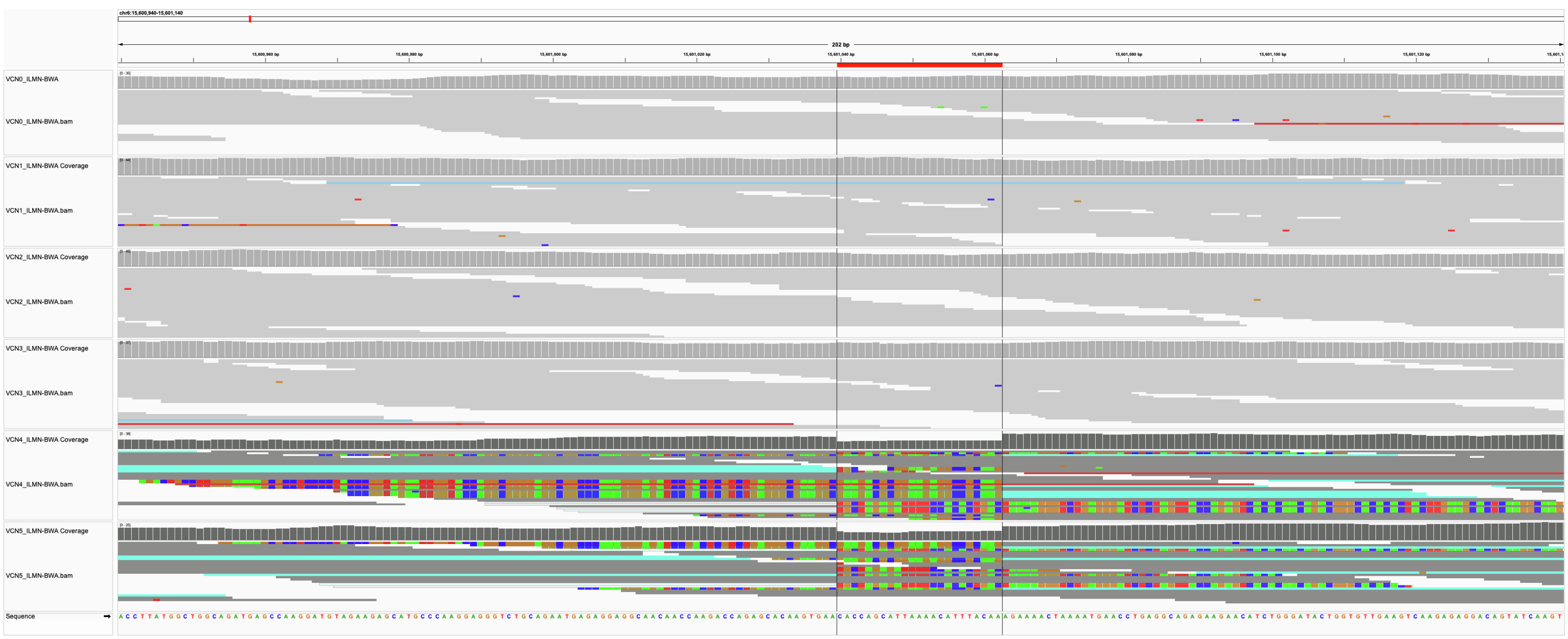

IS9_VCN4-VCN5_chr6:24825065

HiFi

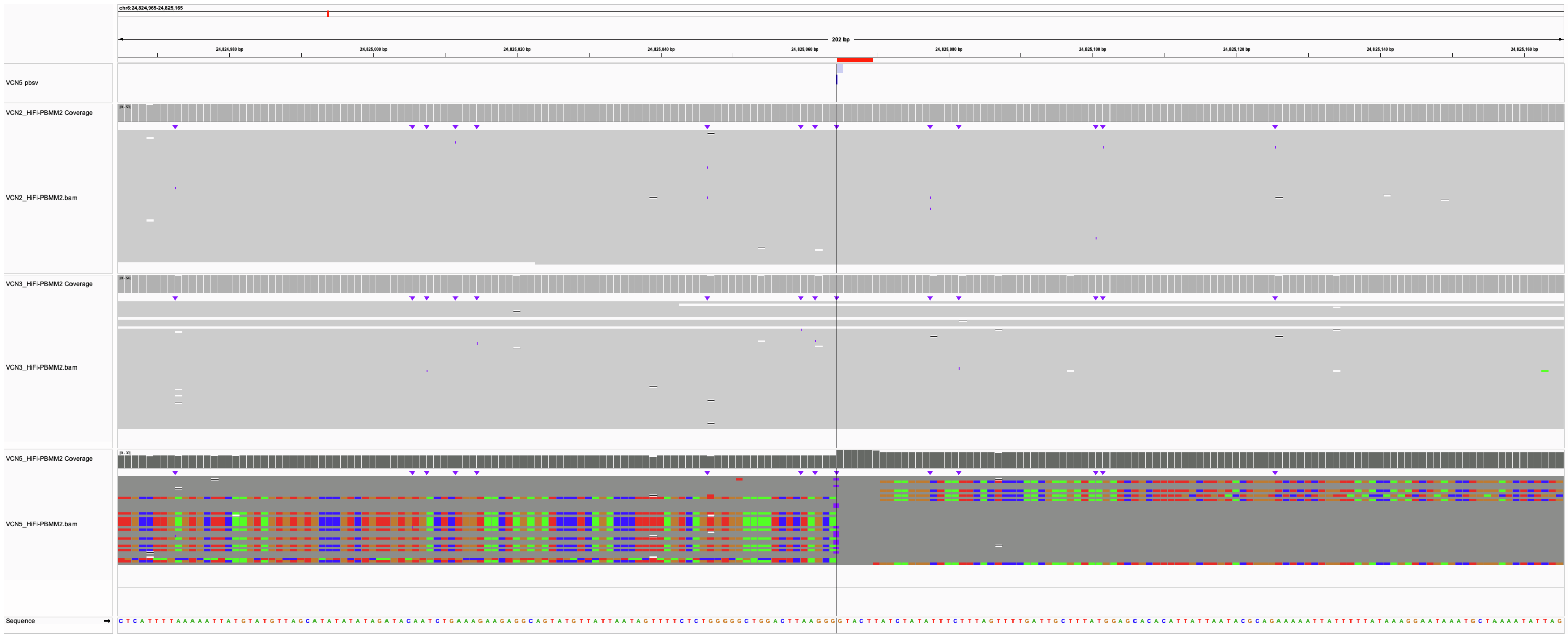

Illumina

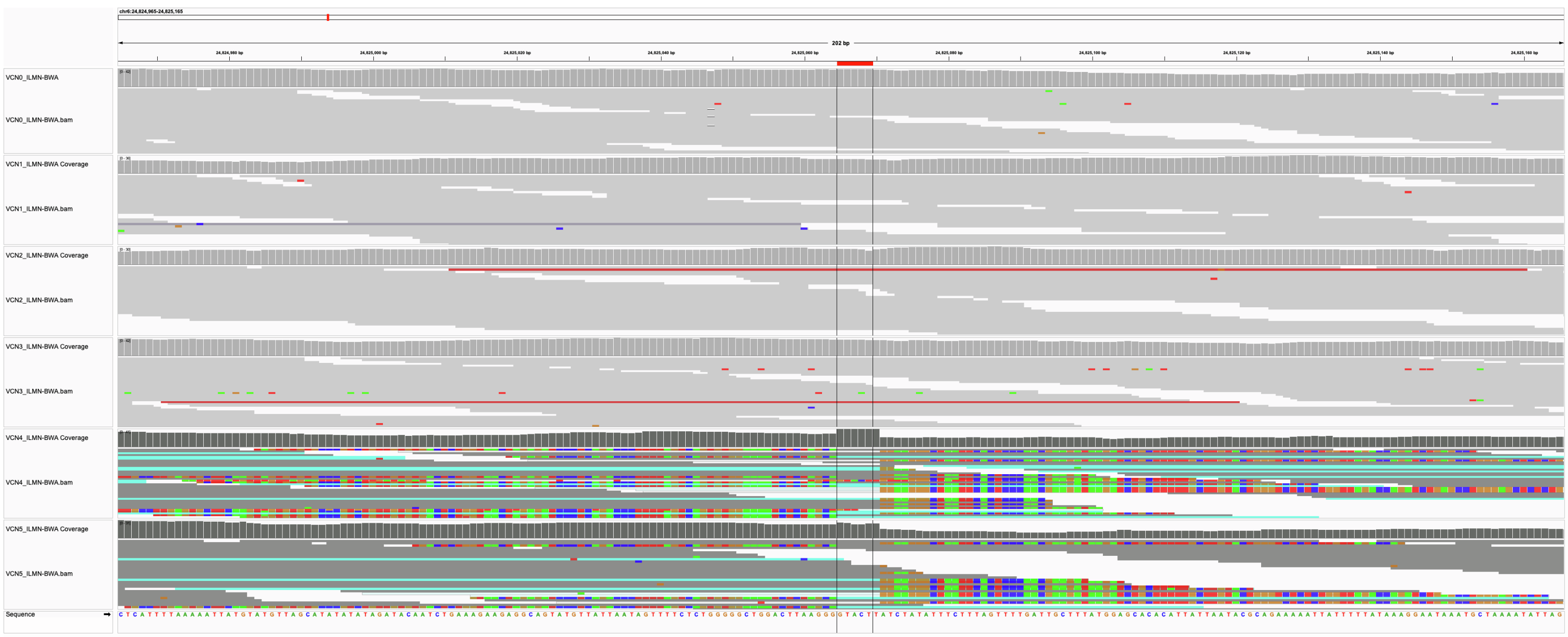

IS10_VCN5_chr5:39044696

HiFi

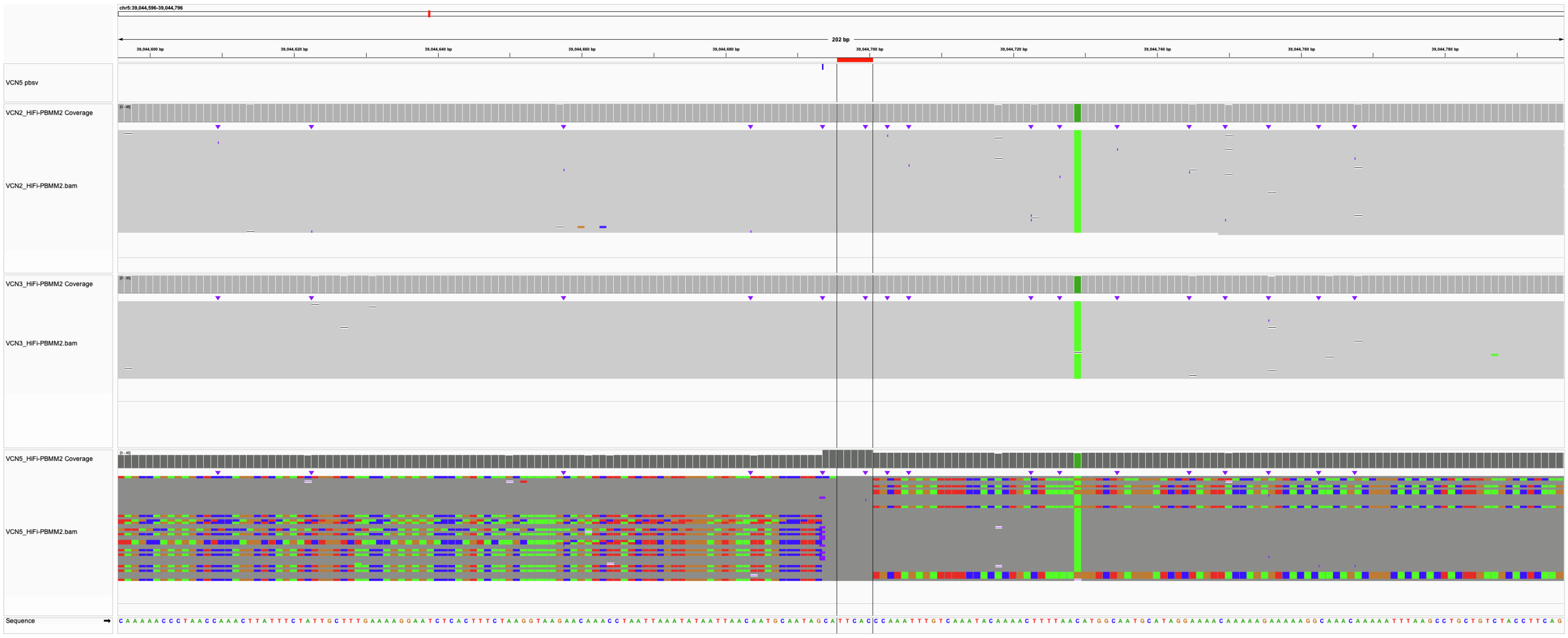

Illumina

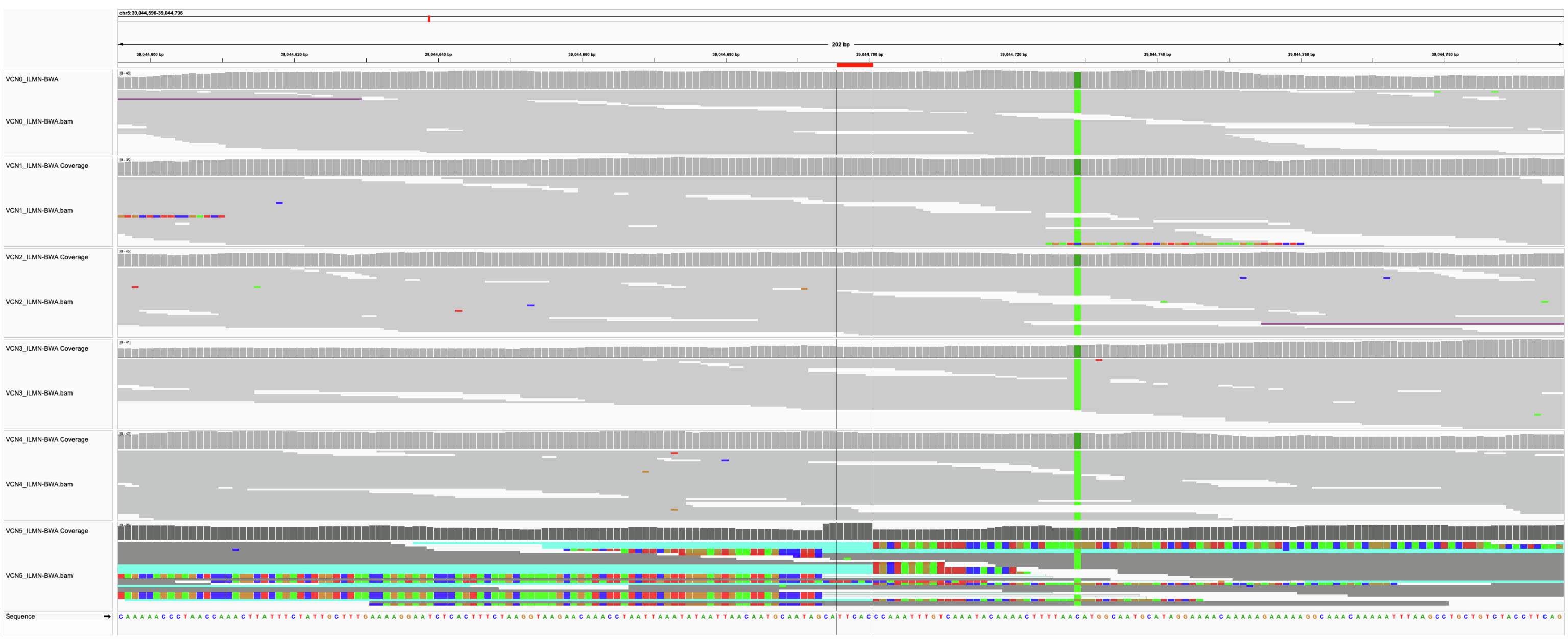

Figure S3: IGV screenshots of integration sites in Jurkat genome

Screenshots are grouped by integration site (IS1 through IS10, further described in Table 1). For each integration site, two representative screenshots are provided, corresponding to Figure 1: one for long-read PacBio HiFi data (aligned with pbmm2) and one for short-read Illumina data (aligned with BWA-mem). Tracks for all clones sequenced (VCN0–VCN5 with Illumina and VCN2, VCN3 and VCN5 with HiFi) are displayed for every locus, regardless of whether a specific clone harbors an integration at that site. Clones lacking the integration serve as additional baseline controls alongside VCN0, representing the unaltered Jurkat genome. In each panel, a red bar and black bounding box highlight the precise integration site within the Jurkat genome. To aid visual identification, tracks are shaded darker grey for VCN clones confirmed to contain the specified integration. For clones analyzed with long-read HiFi sequencing, a pbsv insertion call marks the integration site (typically on the left side of the duplicated bases), and read alignments successfully spanning the locus display a large insertion. Conversely, for HiFi read alignments that do not fully span the insertion, as well as for all short-read Illumina data, read alignments terminate abruptly on either side of the

duplicated bases at the integration site. At these boundaries, multi-colored soft-clipped bases are observed, and colored Illumina reads indicate the other read in pair maps to the proviral sequence.
