# Supplementary material for: Long-read genome sequencing reveals complex variability in lentiviral provirus insertions in clonal CD19 CAR-T VCN reference cell lines
Source: Mol Ther Adv. 2026 Oct 21;34(4):201873. doi: 10.1016/j.omta.2026.201873 (PMC13769313; doi:10.1016/j.omta.2026.201873)
Supplement: Supplementary file 2 [file mmc2.pdf]

Molecular Therapy
Advances
Original Article

# Long-read genome sequencing reveals complex variability in lentiviral provirus insertions in clonal CD19 CAR-T VCN reference cell lines

Zhiyong He,[1] Jennifer H. McDaniel,[1] Linhua Tian,[1] Mahir Mohiuddin,[1] Ningchun Xu,[1] Lili Wang,[1] Sheng Lin-Gibson,[1] Justin M. Zook,[1] and Hua-Jun He[1]

[1]Materials Measurement Laboratory, National Institute of Standards and Technology, 100 Bureau Dr., Gaithersburg, MD 20899, USA

**Characterizing proviral insertions in chimeric antigen receptor (CAR)-T cells is critical for cell therapy product safety and efficacy. We generated clonal CD19 CAR-T cell lines designated VCN1–VCN5 based on RRE copies per cell measured by droplet digital PCR (ddPCR). Some clones also contained additional partial vector-derived integrations not reflected in this designation. Short- and long-read whole-genome sequencing (WGS) were used for characterizing the insertions. Long reads proved essential, fully resolving each insertion and revealing that only 3 of 10 insertions matched the expected sequence. The remainder exhibited structural variants and/or abnormalities. One particularly important structural abnormality resolved only by long reads is a 724-bp deletion of the EF-1α promoter disrupting expression of the CD19 CAR. Standard short-read and ddPCR approaches miss this deletion due to this promoter being in the unengineered human genome. These results demonstrate that these cell lines are suitable VCN candidate reference standards for 1–5 copies of full proviral DNA insertions (VCN4 and VCN5 harboring an extra *EGFP* copy) and highlight the utility of long-read sequencing in characterizing both quantity and quality of insertions in lentiviral-engineered cells.**

## INTRODUCTION

Chimeric antigen receptor (CAR)-T cell therapy is a revolutionary novel cancer therapy and has shown promise in leukemias, with emerging but more limited evidence in solid tumors.[1–5] A significant milestone for CAR-T cell therapy was US Food and Drug Administration (FDA) approval of tisagenlecleucel and axicabtagene ciloleucel, two CD19-specific CAR-T cell products.[4,5] Currently, there are 7 CAR-T cell therapy products approved by the FDA for treating blood cancers such as acute lymphoblastic leukemia (ALL), large B cell lymphoma, and multiple myeloma (https://www.fda.gov/vaccines-blood-biologics/cellular-gene-therapy-products/approved-cellular-and-gene-therapy-products). Many other cancer-related cell surface markers were targeted by CAR-T cell therapy in preclinical and clinical stages, such as CD22,[6] HER2,[7] and EGFR,[8] inspired by the success of these CAR-T cell therapies. A CAR molecule typically contains four functional domains: an extracellular antigen-binding domain, a spacer, a transmembrane domain, and an intracellular activation domain.[9] The T cells for therapy (either autologous or allogeneic) are genetically engineered to express the CAR protein.

Currently, all FDA-approved CAR-T cells are primarily engineered using viral transduction, such as lentiviral and gammaretroviral vectors.[5,10] More pre-clinical research reported using CRISPR-Cas9 to engineer the T cells at specific sites, but none are yet FDA approved.[11–14] The commonly used method for CAR-T production of therapies approved by the FDA is the lentivirus vector (LVV) transduction. LVV is a retrovirus vector derived from HIV and is able to insert the target gene into the cell genome.[15] Thus far, 141 CAR-T and 6 CAR-NK cell therapies utilize lentivirus as the gene delivery method (www.clinicaltrials.gov as of May 2026). Advantages of the LVV method include relatively reliable and efficient gene transfer. However, LVV insertion into the target cell genome is uncontrolled, which could lead to aberrant RNA splicing.[16] High copies of LVV integration may also cause insertional mutagenesis and/or oncogenicity.[17,18] Multiple insertions in the same chromosome close to each other may cause the fragment between the insertions to be inverted or deleted through homologous recombination. Furthermore, the biological process of lentiviral integration (including reverse transcription of LV RNA genome and double-stranded proviral DNA insertion) is inherently error prone.[15,19,20] Therefore, to reduce safety concerns associated with high vector copy number (VCN), it is

Received 24 June 2026; accepted 19 October 2026;
https://doi.org/10.1016/j.omta.2026.201873.

**Correspondence:** Zhiyong He, Materials Measurement Laboratory, National Institute of Standards and Technology, 100 Bureau Dr., Gaithersburg, MD 20899, USA.
**E-mail:** zhiyong.he@nist.gov
**Correspondence:** Justin M. Zook, Materials Measurement Laboratory, National Institute of Standards and Technology, 100 Bureau Dr. Gaithersburg, MD 20899, USA.
**E-mail:** justin.zook@nist.gov
**Correspondence:** Hua-Jun He, Materials Measurement Laboratory, National Institute of Standards and Technology, 100 Bureau Dr. Gaithersburg, MD 20899, USA.
**E-mail:** hua-jun.he@nist.gov


This is an open access article under the CC BY license (http://creativecommons.org/licenses/by/4.0/).

carefully controlled during product development. Further studies of a gene therapy for hematologic cancer identified insertions within oncogenes in patients with subsequent cancer,[21] which suggested that the identification of LVV insertion sites is important for safety. Several case reports prompted an FDA investigation and longitudinal study, which did not find evidence directly linking CAR-T cells to these cancers, though follow-up data remain limited.[22–25]

Common measurement methods for integrated LVV copy number include qPCR, droplet digital PCR (ddPCR), and next-generation sequencing (NGS), each with its own limitations. For example, an earlier report showed that the integrated VCN was often underestimated by the qPCR method.[26] In addition, the assays developed based on each method vary from laboratory to laboratory. Targeted NGS of integration sites has been used to measure integration with short reads[27] and long reads,[28] but these targeted methods did not measure the full insertion sequences. Long reads have detected large structural variants at CRISPR-Cas9-engineered sites,[29] leading the FDA to mention the use of long-reads or alternative methods for large variants (https://www.fda.gov/media/191966/download). Although CRISPR-Cas9 results in different genomic changes than LVVs, long reads also can resolve LVV insertion events difficult to see with short reads. For example, targeted long reads identified HIV-associated internal proviral deletions, and a modified approach recently found promoter deletions in LVVs as well by sequencing full integration sequences.[20,30] For these reasons, the community has called for reference samples for assay validation, optimization, and benchmarking.[31,32]

We previously developed NIST RGTM 10268 using an LVV containing only a cassette for GFP expression.[33] The clonal cell lines contained 0–4 copies of proviral DNA per cell, and all insertion sites were identified.[33] Here, we developed an additional set of VCN candidate reference material for the following reasons. First, the actual lentiviruses used in the clinical setting such as cell and gene therapy usually have longer RNA genomes. Second, since higher VCN samples make it more difficult to reach consensus measurement results,[34,35] higher VCN clonal cell lines need to be included in the candidate reference materials for benchmarking the measurements. In a World Health Organization (WHO) interlaboratory study, measurements of cells with an estimated VCN of 10.67 varied widely from 6.15 to 13.6, and few insertion sites were identified.[35] This broad range highlighted the challenges of measuring VCN for higher copies of high copy number integrated vectors. Moreover, the FDA recommends maintaining a VCN of fewer than 5 copies per cell. Third, there is no CAR expression cassette in the previous candidate reference materials for potential functional CAR cytotoxicity assays. Finally, the proviral DNA sequence of previous candidate reference materials could not be disclosed due to intellectual property restrictions.

To address this unmet need, we developed a set of clonal cell lines with 1–5 copies of integrated CD19 CAR LVV and deeply characterized the integration sites using orthogonal methods. The precise sequences of each LVV insertion in at least one clone were measured with long-read whole-genome sequencing, revealing functionally important structural anomalies in the LVV insertion sequences in the host genome relative to the expected provirus DNA sequence. These clonal cell lines could serve as reference materials for CAR-T cell VCN measurements, insertion site identification, and identification of potential random insertions.

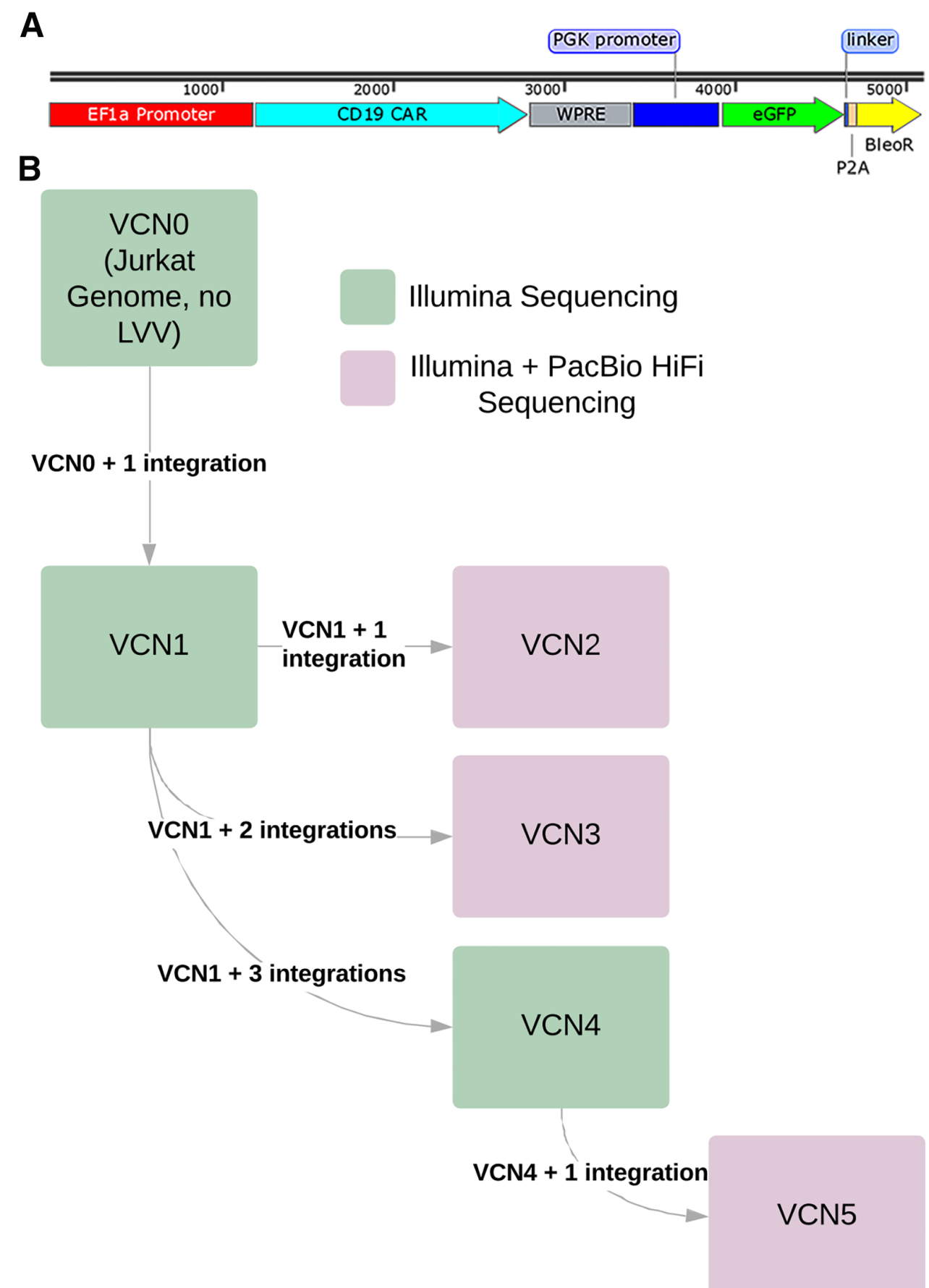


**Figure 1. CD19 CAR vector design and schematic view of VCN clonal cell line generation strategy**

(A) Schematic view of CD19 CAR transfer vector design. (B) Schematic view of VCN clonal cell line generation strategy. VCN was designated by the detected copies of RRE per cell. Short-read sequencing (Illumina) identified integration loci across all clones, whereas long-read sequencing (PacBio) captured the integration loci while also resolving the complete integrated sequence and its integrity. Direct long-read sequencing was performed selectively on VCN2, VCN3, and VCN5. The full integration structures for the intermediate clones, VCN1 and VCN4, were inferred from the shared long-read sequencing data of their respective descendant clones, because no differences were seen in the short-read data for these integration sequences.

## RESULTS

### Stable clonal cell lines with 1–5 copies of integrated CD19 CAR

CD19 CAR lentivirus-transduced Jurkat cells were selected with 10 μg/mL of bleomycin for the first 3 passages and then with 5 μg/mL of bleomycin. The design of CD19 CAR lentivirus is illustrated in Figure 1A. The bleomycin-resistant cells were subjected to single-cell clone printing and screening. The single-cell clones were

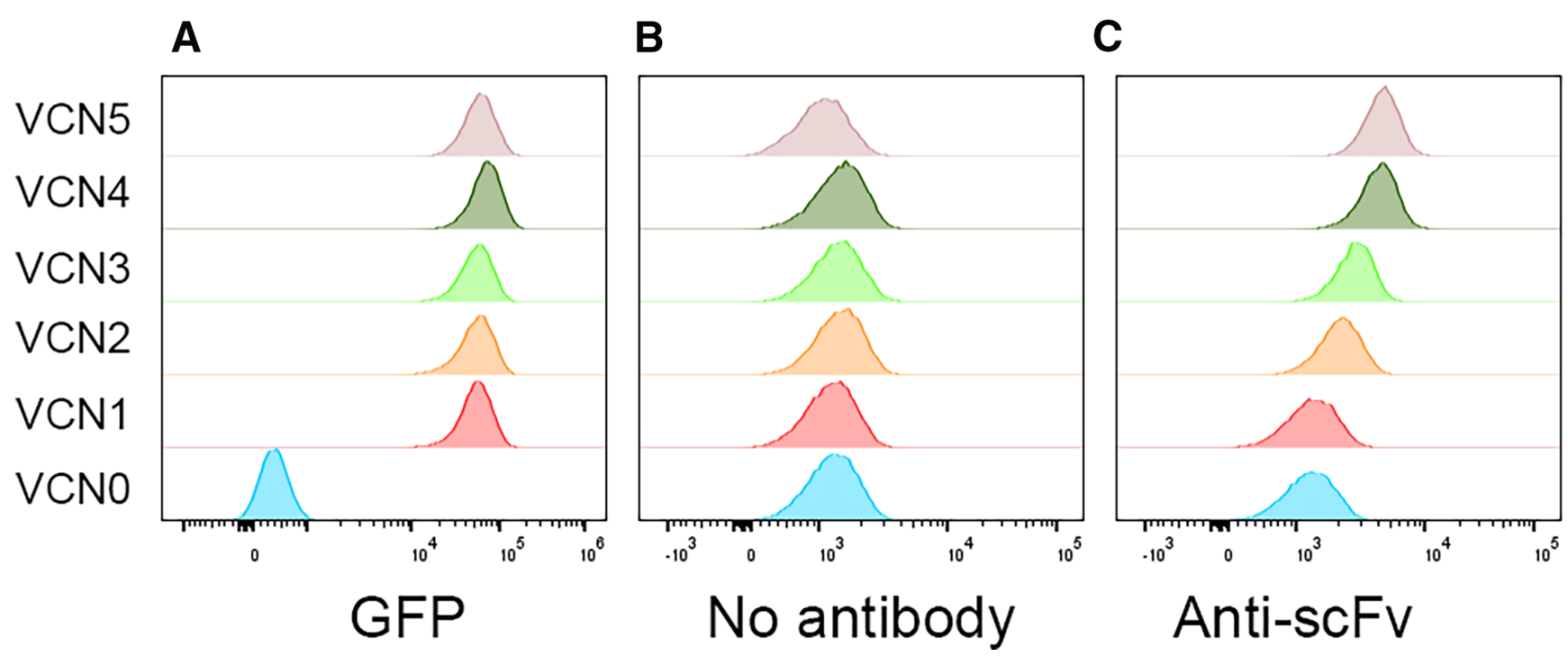


**Figure 2. GFP and CD19 CAR expression in VCN clonal cell lines**

(A) GFP expression of each clonal cell line analyzed by flow cytometry. (B) No primary antibody controls for analyzing CD19 CAR expression by flow cytometry. (C) CD19 CAR surface expression on each clonal cell line. Data are representative of 3 independent experiments.

screened by ddPCR using RRE assay and S2 as single-copy control gene. Initial transduction using MOI = 1 and 5 resulted in mostly VCN1 clonal cell lines (detected copy number of RRE/detected copy number of S2 $\times$ 2 = 1). We only obtained two VCN2 (detected copy number of RRE/detected copy number of S2 $\times$ 2 = 2) clonal cell lines with no GFP expression. We chose one of the GFP-positive VCN1 clonal cell lines for lentiviral transduction to generate higher VCN clonal cell lines (as shown in Figure 1B). Finally, we used VCN4 clonal cell line to generate VCN5 clonal cell line (Figure 1B). We analyzed the cells by flow cytometry to detect the GFP and CD19 CAR expression level. The first-round screening resulted in many clones that either lost GFP expression after a few passages or did not show correlation of CD19 CAR expression with the integrated VCN (data not shown). Thus, we did a second lentivirus transduction on the selected VCN1 clone based on GFP expression. With the same screening process, we obtained VCN2, VCN3, and VCN4 clones that had GFP and CD19 CAR expression correlated with the integrated VCN. We further transduced VCN4 with the same LVV to obtain VCN5 with one additional integration. The GFP expression levels were similar between all the cell lines (Figure 2A); however, the CD19 CAR expression levels were correlated with the integrated VCN (Figures 2B and 2C) except VCN1 cells, which exhibited GFP-positive but no detectable CD19 CAR expression.

### Measurement of VCN by different PCR targets

We utilized the assays developed based on the lentivirus elements (woodchuck hepatitis virus posttranscriptional regulatory element [WPRE], RRE, Psi, and long terminal repeat [LTR] assays were published previously)[36] and the CD19 CAR. Single-copy reference locus S2 and *RPL32* (ribosomal protein L32)[37] were used as single-copy reference genes. Most of the assays resulted in the expected copy number for each VCN cell line (Figure 3). However, VCN3 appeared to have an additional copy for WPRE, and both VCN4 and VCN5 appeared to have an extra copy of *EGFP*, results corroborated below by long-read NGS.

### Stability of the clonal cell lines

The stability of the cell lines was assayed by passaging the cells up to passage 42. Genomic DNA was extracted from the cells every week (3 passages per week) for 8 weeks and was analyzed by ddPCR to quantify the VCNs based on RRE and CD19 CAR. As shown in Figure 4, the VCN cell lines exhibited minimal variations for atleast 8 weeks, which indicated that the cell lines remained stable for the VCN measurements.

### Long-read sequencing identifies structural and small variants in provirus integrations

A combination of long-read and short-read whole-genome sequencing identified and confirmed precise integration sites, target site duplicated sequences, and variation within each inserted sequence in the Jurkat cell genome (Figure 5B). All insertions occurred in introns except for one in the 3′ UTR of *HNRNPR*. Interestingly, of the 10 unique integration events, only 3 contained the expected sequence, with others containing substitutions, deletions, and/or duplications relative to the expected sequence. VCN1 clonal cell line showed one copy of proviral DNA inserted in the minus strand of chromosome 17 at position 78,730,345. Long-read sequencing revealed a 724-bp deletion in the EF-1α promoter region (Figure 5B), which explained why VCN1 cells did not express CD19 CAR (Figure 2C). This deletion is not visible by short-read sequencing due to the low mapping quality caused by high identity with the EF-1α promoter in the reference. Since VCN2–5 clonal cell lines were all derived from VCN1, all the cell lines had a copy of the 724-bp-deletion EF-1α promoter proviral DNA on chromosome 17 (Figure 5B). As expected, VCN2 cells contained an additional insertion. However, it harbored four unexpected substitutions. Although both short reads and long reads detected these substitutions, only long-read data provided sufficient context to confirm that all four were located within the second insertion (Figure 5B, yellow lines). In VCN3 cells, one of the new proviral insertions had the precise expected sequence. However, the other proviral insertion had a mostly expected sequence except for one single-nucleotide variant (SNV), and an additional 804-bp sequence on the 3′ end of the proviral insertion. This additional sequence contains a 778-bp duplication of a portion of the WPRE sequence, followed by 2 bp (GT) and 24 bp of inverted 3′ LTR sequence. This 778-bp duplication accounts for the additional WPRE copy detected by the WPRE ddPCR assay in the VCN3 cell line (Figure 3). Interestingly, both short- and long-read whole-genome sequencing data revealed an additional non-functional 263-bp insertion of predominantly LTR sequence that

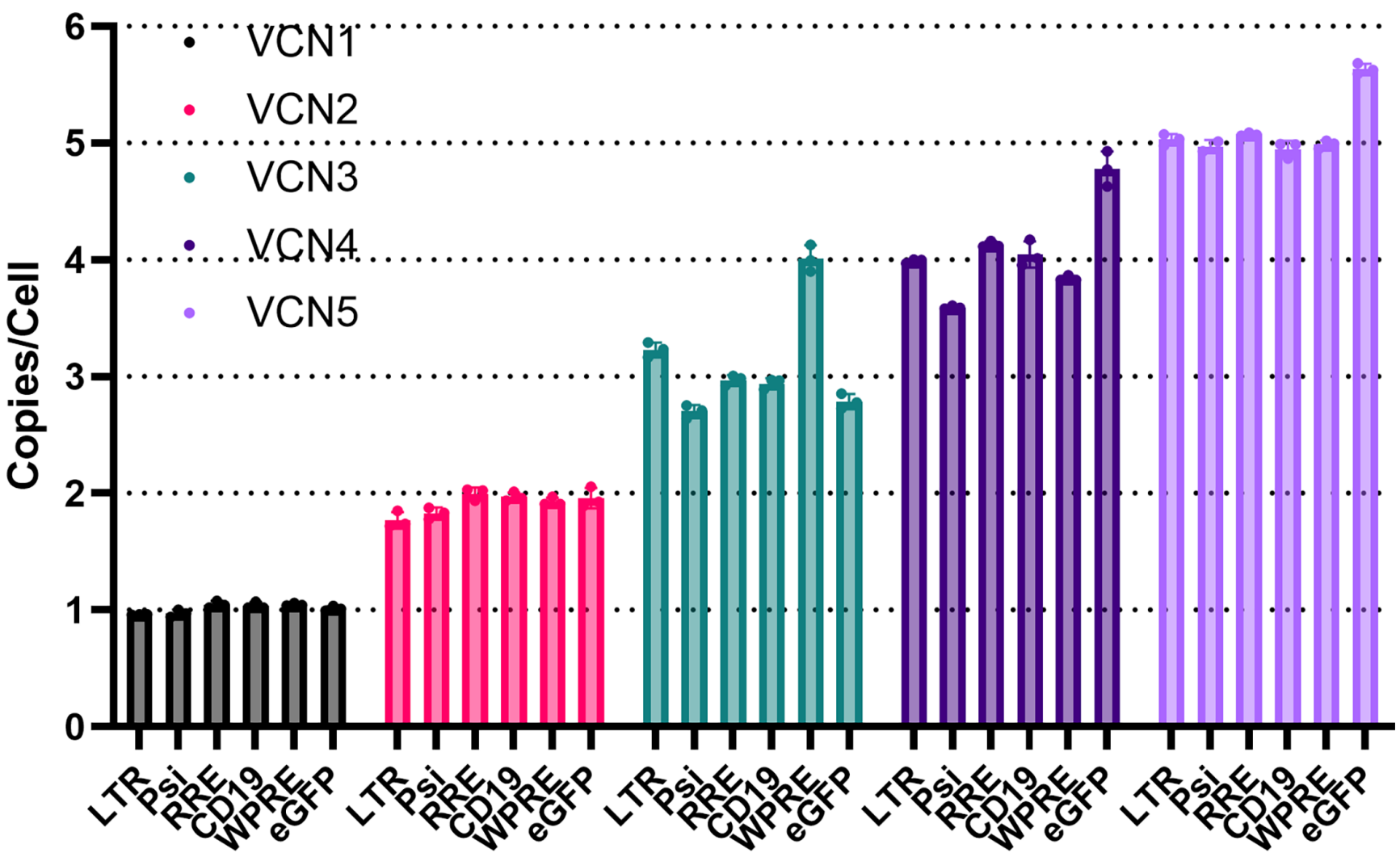


**Figure 3. VCN detected by 6 different assays in each clonal cell line**

Representative of 3 experiments. Error bars represent standard deviation ($n$ = 3). $y$ axis: vector copy number (copies/cell).

was not detected by ddPCR assay. Analysis of VCN4 revealed one new proviral insertion exhibiting the expected sequence, a second containing a single-nucleotide substitution, and a third harboring both a substitution and an additional 41-bp sequence downstream of the 3′ LTR. This additional 41-bp sequence comprises 15 bp of unknown origin and 26 bp derived from the 3′ LTR (Figure 5B). Furthermore, short- and long-read sequencing data identified an additional 2,070-bp integration corresponding to the 3′ region of the proviral DNA. This fragment contained a 1-bp deletion and encompassed the *EGFP* gene, thereby accounting for the extra copy of *EGFP* detected by ddPCR assay in the VCN4 cells (Figure 3). The VCN5 cell line contained all the insertions previously identified in VCN4, alongside one additional insertion exhibiting the precise expected sequence (a representative visualization utilized for manual curation is provided in Figure S1). Furthermore, the allele frequencies of SNVs and insertions/deletions (indels) within the LVV sequences, as determined by both short- and long-read sequencing, corresponded with the expected fraction of variant-containing copies (Table S1). All reported integrated sequences derive from the HiFi pbmm2-pbsv calls, starting with the 4–5 bp of duplicated integration site sequence, and are available in supplemental information. In summary, whole-genome sequencing revealed alterations within multiple inserted sequences, thereby elucidating the unexpected PCR results.

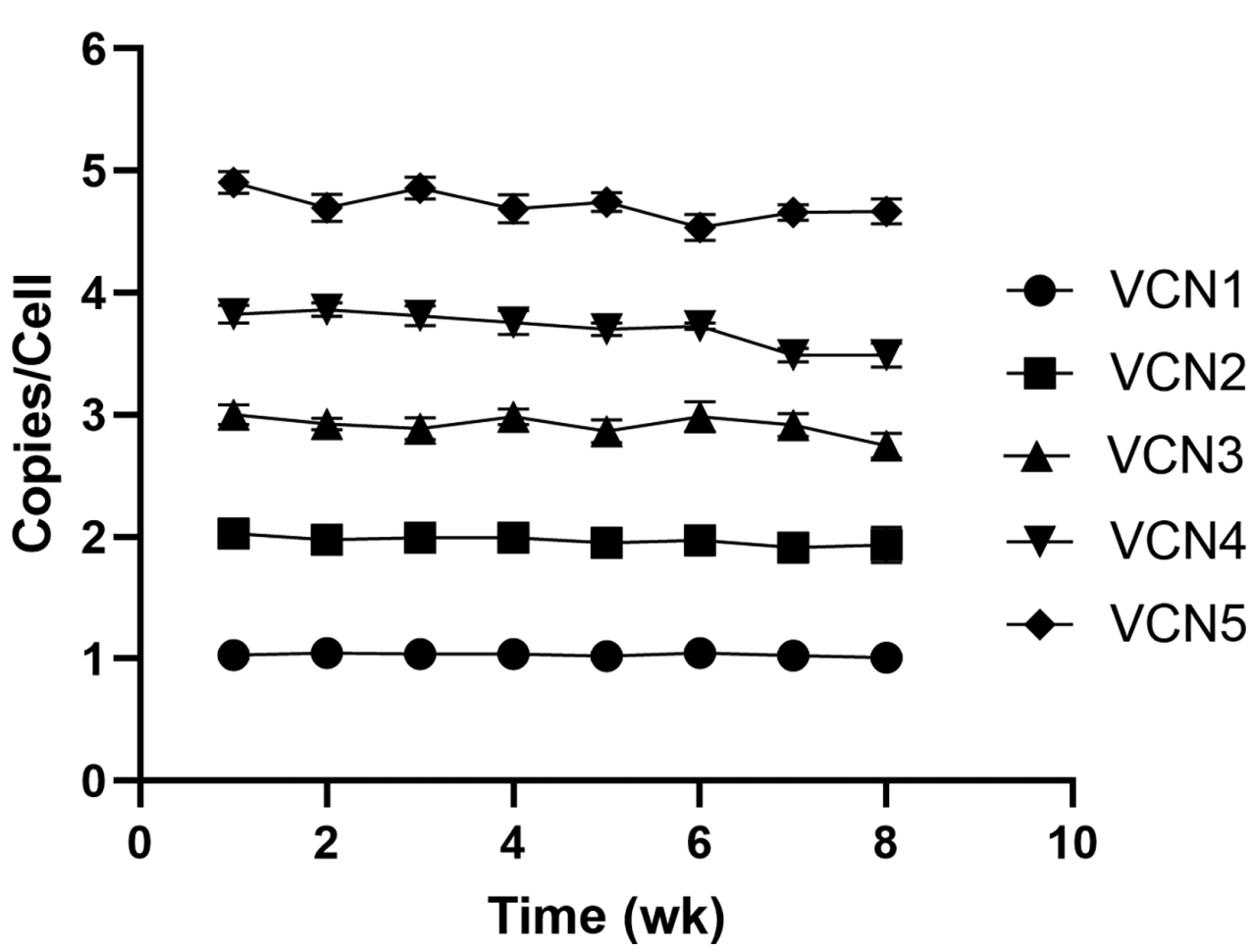


**Figure 4. Stability of clonal cell lines**

VCN of each cell line was determined by ddPCR using RRE and CD19 CAR target assays. Data are representative of three independent experiments. Plotted values represent the mean ± SD of technical triplicates for both assays within a single experiment ($n$ = 6 total technical replicates per timepoint). $x$ axis: time (weeks).

Analysis of both short- and long-read whole-genome sequencing data also revealed the absence of chromosome Y in VCN1–VCN5 cell lines. A previous report[38] and our G-band karyotype analysis (Figure S2A) indicated that a subpopulation of Jurkat cells lacks chromosome Y (7 of 20 metaphase spreads). In this work, sequencing and G-band karyotyping confirmed this absence of chromosome Y in the VCN1 cell line (Figure S2B). Because all the other VCN cell lines were derived directly from VCN1, this shared lineage accounts for the absence of chromosome Y across all the VCN1–VCN5 clonal cell lines.

## DISCUSSION

In this study, we generated deeply characterized clonal cell lines as reference samples for LVV integration site, quality, and copy number. With short- and long-read whole-genome sequencing, we identified all LVV integration sites and sequences, including some small segments of the LVV in the cell lines (Figure 5).[39] Long-read WGS (whole-genome sequencing) uncovered the structural complexity of LVV integrations that were invisible to standard short-read methods. While short-read sequencing is effective for identifying general integration sites, it often fails to resolve full insertion sequences or detect large internal rearrangements due to low mapping quality in repetitive or high-identity regions and an inability to resolve full insertions. A primary example in this study is the characterization of all the integration sites present in the clonal cell lines, where long-read sequencing revealed a 724-bp deletion in the EF-1α promoter region. This specific deletion explained why the VCN1 cells failed to express the CD19 CAR protein despite the presence of the proviral DNA. This was invisible to short-read sequencing because the deletion occurred in a region with high identity to the native EF-1α promoter in the human

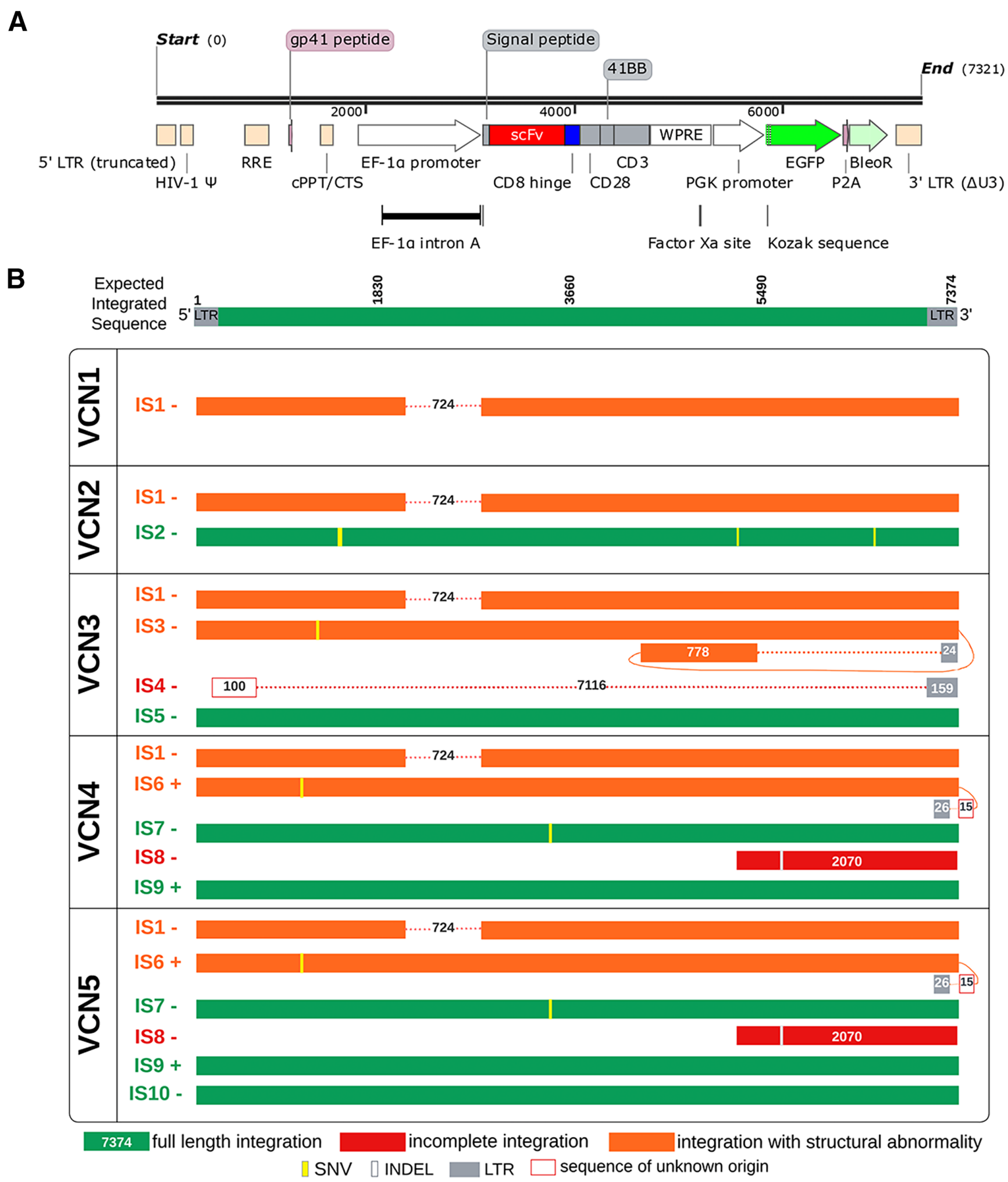


**Figure 5. Schematic diagram of lentiviral DNA insertions and variants inside the insertions in each clonal cell line**

(A) Schematic diagram of each element of the lentiviral DNA insertion. The truncated 5′ LTR is replaced by the 3′ LTR during reverse transcription. (B) Schematic diagram of proviral DNA integration sites across VCN1–VCN5 characterized using short- and long-read sequencing. Integrations are categorized as full length (green), structurally abnormal (orange), or incomplete (red), with genomic orientation indicated by +/− symbols. Horizontal dotted lines signify missing regions, while vertical yellow and white lines denote SNVs and INDELs, respectively. Gray boxes represent LTR sequence. Curved orange lines connect unexpected sequence post expected sequence. More information about each integration is in Table 1 and Tables S2 and S3.

reference genome. Beyond simple deletions, long-read data identified complex variants across the clones, revealing that only 3 of 10 unique integration events actually contained the precise expected sequence. The remaining integrations featured unexpected substitutions, deletions, and duplications, such as the 778-bp duplication found in a VCN3 insertion and a 2,070-bp partial insertion in VCN4 that included an extra copy of the *EGFP* gene. These findings highlight that LVV integration is not always a clean, full-length process and that long-read sequencing is essential for the high-resolution characterization required to validate reference materials and may help ensure the safety of CAR-T cell therapies. One of the possible mechanisms of the deletion of 724 bp in the EF-1α promoter is that the host-recognized cryptic splicing during viral RNA transcription in packaging cells generates truncated genomes that are subsequently packaged and integrated. If such truncated species constitute a certain subpopulation in the given LVV preparation batch, similar deletion patterns would likely recur in independent transductions using the same LVV preparation. It is unclear whether the cryptic splicing is specific to promoter sequence since Kaiser et al. revealed deletions in both the human ubiquitin C promoter and the EF-1α promoter.[30] Whether vector optimization can eliminate the cryptic splicing motifs in the LVV warrants further investigation. Ultimately, these findings further support developing a standardized reference LVV backbone along with genomic reference cell lines, which could enable the field to systematically decouple vector-intrinsic sequence instability from host cell- or assay-driven variations.

To echo the findings in these CAR VCN materials, we also found SNVs in the proviral DNA regions in VCN4, VCN2, and VCN1 samples of NIST Research Grade Test Material (RGTM) 10268[33] and a 648-kb genomic deletion near the integration site Chr14: 22 564 447 in the VCN3 sample (unpublished data from the RGTM 10268).

There are some limitations of this study. First, we generated our clonal VCN cell lines using Jurkat cells, a leukemia cell line known for inherent genomic instability, including frequent mutations, chromosome rearrangements, and single-nucleotide polymorphisms.[40] Because of this instability, it is possible that the variant lentiviral DNA insertions we observed were a by-product of the host cells' tendency to rearrange or truncate DNA during or after integration. However, we did not observe partial lentiviral DNA insertions in our previous study,[33] which indicated that the likelihood of Jurkat cell genome causing these specific partial insertions is low. In addition, a new targeted long-read approach identified similar promoter deletions in a variety of non-clonal CAR-T products.[30] In the future, since we established a novel method for immortalizing human primary T cells,[41] we plan to collect explicitly consented patient primary cells to establish immortalized T cell

**Table 1. Summary of all integration sites and integration observations**

| Integration identifier (IS_ID) | VCN | Chromosome | Start | Integration site sequence | Integrated sequence integrity | Integrated sequence length |
|---|---|---|---|---|---|---|
| IS1 | VCN1, VCN2, VCN3, VCN4, VCN5 | chr17 | 78,730,345 | GAAAC | large 724-bp deletion in the middle of the integrated sequence (bases 1,917–2,640 of the expected sequence) in the EF-1α promoter region. | 6,655 |
| IS2 | VCN2 | chr19 | 21,610,872 | CCCCC | four SNVs identified in integrated sequence at expected sequence positions 1,692:A>T, 1,693: C>T, 4,878: G>C, and 6,601: G>A. Other than SNVs, no other abnormalities. | 7,379 |
| IS3 | VCN3 | chr1 | 23,307,863 | ACAGG | structural abnormality at the 3′ end of a full-length integration. The abnormality contains additional sequence composed of a large duplication, corresponding to expected integration sequence positions 4,416–5,193 (778 bp), followed by 2 bp (GT), and then 24 bp of the 3’ LTR sequence (AGTCAGTGTGGAAAATCTCTAGCA). Within the full-length portion of this integration, there is an SNV T>A at position 1,450. | 8,183 |
| IS4 | VCN3 | chr8 | 99,830,407 | AAAC | a short 263-bp integrated sequence primarily composed of LTR sequence was identified. The sequence begins with a 4-bp duplication of the chr8 integration site. Following this duplication, 159 bp correspond to LTR positions 7,216–7,374 of the expected integration. The remaining 100 bp are less clearly defined but appear to include a mixture of unknown and LTR-derived sequences (33 bp unknown, 21 bp possible LTR, and 46 bp unknown using BLAST). BLAST of the 79-bp “unknown” sequence was performed, and no significant similarities were found. | 263 |
| IS5 | VCN3 | chrX | 103,930,456 | AATAC | expected integration sequence | 7,379 |
| IS6 | VCN4, VCN5 | chr1 | 243,273,502 | ACCCC | SNV G>A at position 1,066 in full integrated sequence. End of full integrated sequence has an additional 41 bp. This sequence appears to be 15 bases of unknown origin followed by the first 26 bp of the LTR sequence in reverse orientation. | 7,420 |
| IS7 | VCN4, VCN5 | chr3 | 18,115,277 | TACTC | SNV G>A at full integrated sequence position 3,649. Other than SNV, no other abnormalities. | 7,379 |
| IS8 | VCN4, VCN5 | chr6 | 15,601,040 | unclear, possibly AG | integrated sequence is incomplete (2,070 bp) and only contains bases corresponding to positions 5,306–7,374 of the expected integration sequence. It appears that 22 bases of chr6 were deleted due to the insertion (chr6:15,601,040–15,601,062). 22 bases out of 24 bases, “CGAAGACGCGGAAGAGGCCGCA,” align to the expected integration sequence, but the final two bases, “AG,” do not. These two bases are part of chr6 sequence adjacent to the deleted portion of chr6, so they may have been duplicated upon | 2,070 |

(*Continued on next page*)

**Table 1. Continued**

| Integration identifier (IS_ID) | VCN | Chromosome | Start | Integration site sequence | Integrated sequence integrity | Integrated sequence length |
|---|---|---|---|---|---|---|
| | | | | | integration. There is also deletion of a "C" at position 5,579 in a C homopolymer. | |
| IS9 | VCN4, VCN5 | chr6 | 24,825,065 | GTACT | expected integration sequence | 7,379 |
| IS10 | VCN5 | chr5 | 39,044,696 | TTCAC | expected integration sequence | 7,379 |

The IS_ID corresponds to chromosome integration site; IS_IDs can be shared across VCN. Integration positions on the chromosome (start) are the first base of the integration site; they are not left aligned. Positions of abnormalities are relative to the expected proviral sequence, not the position within the sequence reported in the fasta format. A full summary of all integrations and visualization of the integration sites are listed in Tables S2 and S3 and Figure S3, respectively.

lines, which would minimize the possibility of rearrangements or mutations in the cell line. Second, this set of materials has yet to be validated by multiple laboratories to prove its utility and commutability.

### Conclusion

We developed a set of VCN clonal cell lines as candidate reference samples, aimed to assure confidence in measurements of one to five LVV copies, as well as integration sites and sequences to evaluate insertion quality.

## MATERIALS AND METHODS

### Establishment of CD19 CAR-T VCN standard cell lines

Anti-CD19 CAR-T lentivirus transfer vector was designed at NIST based on the third generation of lentivirus packaging system.[1] CD19 CAR vector design is illustrated in Figure 1A. Briefly, the *CD19* CAR coding sequence was driven by an EF-1α promoter. A WPRE sequence was placed downstream of the *CD19* CAR coding sequence. The *EGFP* coding sequence and bleomycin-resistant sequence were separated by a P2A sequence under the same PGK promoter. The transfer vector sequence was validated by Sanger sequencing. The lentivirus particles were packaged by VectorBuilder (Malvern, USA). Jurkat cell line (E6-1), purchased from ATCC (Cat# TIB-152, Manassas, VA), was transduced at different multiplicity of infection (MOI = 1, 5, and 10) to generate the VCN cell lines. The transduced cells were then selected with a bleomycin mass concentration of 10 μg/mL in RPMI-1640 growth medium supplemented with 10 % fetal bovine serum (FBS, Thermo Fisher, Cat# A5670402) for selecting the positive integration for the first 3 passages and then with 5 μg/mL of bleomycin. The selected positive single cells were deposited into each well of 96-well plates by using Cytena Single-Cell Printer (Cytena) to generate the clonal cell lines. The clonal cell lines were screened by ddPCR using RRE ddPCR assay[36] and S2 as single-copy reference locus or *RPL32* as the reference gene. The VCN cell lines were then further expanded and characterized.

The clonal CD19 CAR vector copy-number candidate reference cell lines were generated from ATCC TIB-152 (Jurkat E6-1) and are therefore subject to applicable ATCC material-transfer restrictions. A request to deposit the VCN1–VCN5 clones with ATCC has been submitted and is currently under review, with the goal of making these reference cell lines broadly available through ATCC.

### ddPCR

Integrated VCN was measured by ddPCR using the common third-generation lentiviral vector elements, LTR, Psi, RRE, and WPRE (target elements). The primer and probe sequences are listed in our previous publication.[36] Additional target genes *EGFP* and *CD19* CAR were also used to measure the VCNs. Bio-Rad ddPCR Supermix for probes (no dUTP) was purchased from Bio-Rad (Cat# 186–3024, Hercules, CA). Genomic DNA (gDNA) was extracted from each cell line using Quick-DNA Miniprep Kit (Zymo Research, Cat # D3024). 5–20 ng of gDNA was used per ddPCR

reaction; the same amount of DNA template was used for the target genes and reference genes (S2 or *RPL32*) in each reaction. VCN was calculated as the ratio of the lentiviral vector elements to the reference gene determined with the Bio-Rad QX200 ddPCR system following manufacturer's instructions.[33] VCNs were calculated by the following formula:

$$VCN = \frac{Copies\ of\ target\ gene}{Copies\ of\ reference\ gene} \times 2$$

VCN results at the screening stage using RRE and S2 assays were assigned to the clonal cell lines. For example, if a clonal cell line had copies of RRE/copies of S2 × 2 = 3, this clonal cell line was designated as VCN3.

#### Flow cytometry analysis

The expression of EGFP and CD19 CAR in the clonal VCN cell lines was analyzed by flow cytometry. The cells ($5 \times 10^5$ cells per sample) were washed with PBS containing 10 % FBS (fluorescence-activated cell sorting [FACS] buffer) once and resuspended in 100 μL of FACS buffer. The cells were then incubated with 2 ng of antibody, biotinylated anti-FMC63 scFV antibody (ACROBiosystems, Cat# FM3-BY45), on ice for 30 min. After 30 min, the cells were washed with 1 mL of FACS buffer once and resuspended in 100 μL of FACS buffer and 100 ng of APC-conjugated streptavidin was added to each sample and further incubated on ice for 30 min. The cells were washed once with FACS buffer, resuspended in 300 μL of FACS buffer, and then subjected to flow cytometry on a CytoFLEX LX Flow Cytometer. Cells incubated exclusively with streptavidin-APC in the absence of primary antibody incubation served as no-antibody controls. The experiment was repeated three times to confirm the repeatability of the results.

#### NGS

The lentiviral vector integration sites in parental Jurkat cell line genome were determined by whole-genome sequencing analysis. Genomic DNA extracted from each cell line was sent to Novogene for short-read Illumina library preparation and whole-genome sequencing. Whole-genome long-read PacBio sequencing of VCN2, VCN3, and VCN5 was performed by Maryland Genomics. The raw sequencing data were analyzed at NIST to identify copy numbers of the integrated provirus sequence and their precise integration sites. Whole-genome sequencing data were deposited at the NIH/NCBI/SRA BioProject: PRJNA1282768 (Biosamples: SAMN 49572894, VCN0; SAMN49572895, VCN1; SAMN49572896, VCN2; SAMN49572897, VCN3; SAMN49572898, VCN4; and SAMN 49572899, VCN5.)

#### NGS read alignment to reference genome

We used short- and long-read whole-genome sequencing (Illumina 2 × 150 bp and PacBio HiFi, N50 range of 18,751–22,133 bp) to identify LVV integration sites (ISs) in the Jurkat cell genome. Reads were mapped to a modified reference genome (GRCh38-GIABv3_plusInsert-with-3primeLTR_flanks.fasta). Specifically, the expected 7,374-bp LVV sequence was added as an additional chromosome to the GIABv3 (https://ftp-trace.ncbi.nlm.nih.gov/ReferenceSamples/giab/release/references/GRCh38/) GRCh38 human reference genome, which masks false duplications and adds decoy sequences from CHM13.[42] Unlike the original LVV RNA sequence (7,321 bases), the LVV sequence in our reference has identical 5′ and 3′ LTRs because during reverse transcription, the U3 region of the viral genome is copied to the 5′ LTR (7,374 bp).[43] Both long and short reads were mapped to the modified reference using BWA-MEM (v.0.7.17, https://arxiv.org/abs/1303.3997) with default parameters. The resulting alignment files (.sam) were then converted to .bam files, sorted, and indexed with Samtools (v.1.9)[44] view, sort, and index, respectively. Duplicates were then marked with Picard (v.2.18.29) MarkDuplicates (https://broadinstitute.github.io/picard/) for short reads. Alignment post processing steps used tool default parameters. Additionally, long reads were mapped to the modified reference using pbmm2 (v.1.13.1, https://github.com/PacificBiosciences/pbmm2), a minimap2[45] wrapper for PacBio reads, using default parameters. BWA alignments of Illumina and PacBio reads resulted in approximately 27× coverage. Pbmm2 alignments of PacBio reads resulted in approximately 38× coverage.

#### Variant calling

Variant calling was performed to confirm the expected number of integration sites (ISs) in the Jurkat cell genome, identify the integration locations in GRCh38, and compare the actual inserted sequence to the expected LVV sequence. Insertions were identified using two distinct structural variant (SV) callers: Delly (v.1.2.6)[46] for short reads data and PacBio pbsv (v.2.9.0, https://github.com/PacificBiosciences/pbsv) for long read sequencing data. Variant calling was performed with the BWA short-read alignments and pbmm2 long-read alignments against the modified reference.

#### Identification and confirmation of putative integration sites using NGS

To identify putative integration sites, Delly was used to identify translocations and breakpoints when variant calling with BWA-aligned short reads resulted in passing SVTYPE=BND (breakend) on the LVV chromosome with ALT sequence identified on a different chromosome. Putative integration sites were then confirmed by manually curating sites using all alignments and variant calls with the Integrative Genome Viewer (IGV v.2.16.2). For a given VCN, the VCN alignments, variant calls, and modified reference genome were loaded into an IGV session. VCN0 alignments were also loaded in to serve as a negative control, as no integrations are expected in this sample. All alignments to the LVV chromosome were first examined to identify reads that were partially or fully aligned to the expected LVV sequence. BWA short-read alignments were first "grouped by chromosome" to quickly visualize where short-read paired mates map to other chromosomes. It was expected that this grouping would align with ALT chromosomes identified *as BND calls with* Delly. Each integration site had an increase in short-read coverage for the 4–5 bp that are duplicated around the LVV inserted sequence during repair of the staggered cuts. While paired-end short-read alignments were useful for identifying integration sites, they *cannot generally confirm the*

expected full integration of the LVV sequence. To confirm full integration, PacBio long-read alignments and variant calls were used. To cover all possible integrations, VCN2, VCN3, and VCN5 were sequenced with PacBio. Karyotyping demonstrated that this Jurkat clone was near diploid in most cells except for deletions of chromosome Y and the p arm of chromosome 2; so all reads from one haplotype will support the integration at a given site. For PacBio, BWA alignments generally have split reads at integration sites, where part of the read maps to the GRCh38 reference chromosome and the remainder to the LVV chromosome as a supplementary alignment. In contrast, pbmm2 has more reads aligning across the integration site with an insertion that corresponds to the LVV inserted sequence, with no supplementary alignments to the LVV chromosome if the read contains the insertion. Pbsv insertion variant calls at the pbmm2 PacBio alignment integration sites were used to confirm complete insertion of the LVV sequence. To confirm integration of the LVV sequence and that no partial integrations were missed, all pbsv identified insertions greater than 50 bp were aligned back to the modified reference to determine if they aligned to the LVV sequence. These alignments were visualized in IGV. All previously identified integrations were confirmed, and no additional integrations were present.

SNVs and a 1-bp deletion were identified within the integrated sequences when pbsv insertion calls aligned to the LVV chromosome in the modified reference. We evaluated support for the putative variants in PacBio and Illumina reads. Generally, if a variant was present, the fraction of reads supporting the variant should be consistent with the fraction of insertions within the genome containing the variant.

## DATA AND CODE AVAILABILITY

Whole-genome sequencing data are available on public databases (NCBI Bioproject accession: PRJNA1282768). The National Institute of Standards and Technology Research Protections Office reviewed the protocol (MML-2024-0377) for this project and determined it is "not human subjects research" as defined in 15CFR27, the Common Rule for the Protection of Human Subjects. The Jurkat cell line was derived from tissues from a 14-year-old with acute T cell leukemia in the 1970s. The cell line was established prior to the current research use consent process. In 2013, The Cancer Genome Atlas (TCGA) steering committee administered by NCI and NHGRI at the NIH decided that the benefits outweighed the risks for making genomic data fully public without controlled access for Jurkat and other widely used, commercially available cell lines in the Cancer Cell Line Encyclopedia (https://www.cancer.gov/ccg/research/structural-genomics/tcga/history/policies/ccle-open-release-justification.pdf). For the Jurkat cell line, short-read whole-genome sequencing data were previously published (https://bmcgenomics.biomedcentral.com/articles/10.1186/s12864-018-4718-6) without controlled access in public databases, so there is minimal additional risk of reidentification for publishing the long-read whole-genome sequencing data in this manuscript. To make progress toward similar cell lines with explicit consent for public genome data sharing and cell line development, NIST is currently working toward establishing T cell lines and other types of cell lines from individuals who have provided consent under the Personal Genome Project (https://www.pnas.org/doi/abs/10.1073/pnas.1201904109). This consent includes permission to share genomic data and cell lines publicly for open research and commercial use, understanding the risks associated with this. For these reasons, the NIST Research Protections Office determined that the whole-genome sequencing data generated in this work could be made publicly available.

## ACKNOWLEDGMENTS

This work is solely funded by NIST intramural fund. We thank Dr. Jerilyn R. Izac and Dr. Simona Patange for their critical reading, insightful comments, and valuable suggestions.

## AUTHOR CONTRIBUTIONS

Conceptualization, Z.H., J.H.M., L.W., S.L.-G., J.M.Z., and H.-J.H.; methodology, Z.H., J.H.M.., L.T., M.M., N.X., S.L.-G., J.M.Z., and H.-J.H.; investigation, Z.H., J.H.M., L.T., M.M., and N.X.; data curation, Z.H., J.H.M., L.T., and J.M.Z.; resources, Z.H., L.W., S.L.-G., J.M.Z., and H.-J.H.; writing – original draft preparation, Z.H., J.H.M., and J.M.Z.; writing – review and editing, Z.H., J.H.M., L.T., M.M., N.X., L.W., S.L.-G., J.M.Z., and H.-J.H.; visualization, Z.H., J.H.M., L.T., and N.X.

## DECLARATION OF INTERESTS

Certain commercial equipment, instruments, and materials are identified to specify the experimental procedure. In no case does such identification imply recommendation or endorsement by the National Institute of Standards and Technology, nor does it imply that the materials or equipment are necessarily the best available for the purpose.

## SUPPLEMENTAL INFORMATION

Supplemental information can be found online at https://doi.org/10.1016/j.omta.2026.201873.

## REFERENCES


1. Dull, T., Zufferey, R., Kelly, M., Mandel, R.J., Nguyen, M., Trono, D., and Naldini, L. (1998). A third-generation lentivirus vector with a conditional packaging system. J. Virol. *72*, 8463–8471. https://doi.org/10.1128/JVI.72.11.8463-8471.1998.
2. Grupp, S.A., Kalos, M., Barrett, D., Aplenc, R., Porter, D.L., Rheingold, S.R., Teachey, D.T., Chew, A., Hauck, B., Wright, J.F., et al. (2013). Chimeric antigen receptor-modified T cells for acute lymphoid leukemia. N. Engl. J. Med. *368*, 1509–1518. https://doi.org/10.1056/NEJMoa1215134.
3. Rosenbaum, L. (2017). Tragedy, Perseverance, and Chance - The Story of CAR-T Therapy. N. Engl. J. Med. *377*, 1313–1315. https://doi.org/10.1056/NEJMp1711886.
4. Maude, S.L., Laetsch, T.W., Buechner, J., Rives, S., Boyer, M., Bittencourt, H., Bader, P., Verneris, M.R., Stefanski, H.E., Myers, G.D., et al. (2018). Tisagenlecleucel in Children and Young Adults with B-Cell Lymphoblastic Leukemia. N. Engl. J. Med. *378*, 439–448. https://doi.org/10.1056/NEJMoa1709866.
5. Neelapu, S.S., Locke, F.L., Bartlett, N.L., Lekakis, L.J., Miklos, D.B., Jacobson, C.A., Braunschweig, I., Oluwole, O.O., Siddiqi, T., Lin, Y., et al. (2017). Axicabtagene Ciloleucel CAR T-Cell Therapy in Refractory Large B-Cell Lymphoma. N. Engl. J. Med. *377*, 2531–2544. https://doi.org/10.1056/NEJMoa1707447.
6. Fry, T.J., Shah, N.N., Orentas, R.J., Stetler-Stevenson, M., Yuan, C.M., Ramakrishna, S., Wolters, P., Martin, S., Delbrook, C., Yates, B., et al. (2018). CD22-targeted CAR T cells induce remission in B-ALL that is naive or resistant to CD19-targeted CAR immunotherapy. Nat. Med. *24*, 20–28. https://doi.org/10.1038/nm.4441.
7. Hegde, M., Mukherjee, M., Grada, Z., Pignata, A., Landi, D., Navai, S.A., Wakefield, A., Fousek, K., Bielamowicz, K., Chow, K.K.H., et al. (2016). Tandem CAR T cells targeting HER2 and IL13Ralpha2 mitigate tumor antigen escape. J. Clin. Invest. *126*, 3036–3052. https://doi.org/10.1172/JCI83416.
8. Li, H., Huang, Y., Jiang, D.Q., Cui, L.Z., He, Z., Wang, C., Zhang, Z.W., Zhu, H.L., Ding, Y.M., Li, L.F., et al. (2018). Antitumor activity of EGFR-specific CAR T cells against non-small-cell lung cancer cells in vitro and in mice. Cell Death Dis. *9*, 177. https://doi.org/10.1038/s41419-017-0238-6.
9. Oldham, R.A.A., and Medin, J.A. (2017). Practical considerations for chimeric antigen receptor design and delivery. Expert Opin. Biol. Ther. *17*, 961–978. https://doi.org/10.1080/14712598.2017.1339687.
10. Wang, M., Munoz, J., Goy, A., Locke, F.L., Jacobson, C.A., Hill, B.T., Timmerman, J.M., Holmes, H., Jaglowski, S., Flinn, I.W., et al. (2020). KTE-X19 CAR T-Cell Therapy in Relapsed or Refractory Mantle-Cell Lymphoma. N. Engl. J. Med. *382*, 1331–1342. https://doi.org/10.1056/NEJMoa1914347.
11. Razeghian, E., Nasution, M.K.M., Rahman, H.S., Gardanova, Z.R., Abdelbasset, W.K., Aravindhan, S., Bokov, D.O., Suksatan, W., Nakhaei, P., Shariatzadeh, S., et al. (2021). A deep insight into CRISPR/Cas9 application in CAR-T cell-based tumor immunotherapies. Stem Cell Res. Ther. *12*, 428. https://doi.org/10.1186/s13287-021-02510-7.
12. Wagner, D.L., Koehl, U., Chmielewski, M., Scheid, C., and Stripecke, R. (2022). Review: Sustainable Clinical Development of CAR-T Cells - Switching From Viral Transduction Towards CRISPR-Cas Gene Editing. Front. Immunol. *13*, 865424. https://doi.org/10.3389/fimmu.2022.865424.

13. Ureña-Bailén, G., Lamsfus-Calle, A., Daniel-Moreno, A., Raju, J., Schlegel, P., Seitz, C., Atar, D., Antony, J.S., Handgretinger, R., and Mezger, M. (2020). CRISPR/Cas9 technology: towards a new generation of improved CAR-T cells for anticancer therapies. Brief. Funct. Genomics *19*, 191–200. https://doi.org/10.1093/bfgp/elz039.

14. Dimitri, A., Herbst, F., and Fraietta, J.A. (2022). Engineering the next-generation of CAR T-cells with CRISPR-Cas9 gene editing. Mol. Cancer *21*, 78. https://doi.org/10.1186/s12943-022-01559-z.

15. Durand, S., and Cimarelli, A. (2011). The inside out of lentiviral vectors. Viruses *3*, 132–159. https://doi.org/10.3390/v3020132.

16. Cesana, D., Sgualdino, J., Rudilosso, L., Merella, S., Naldini, L., and Montini, E. (2012). Whole transcriptome characterization of aberrant splicing events induced by lentiviral vector integrations. J. Clin. Invest. *122*, 1667–1676. https://doi.org/10.1172/JCI62189.

17. Espinoza, D.A., Fan, X., Yang, D., Cordes, S.F., Truitt, L.L., Calvo, K.R., Yabe, I.M., Demirci, S., Hope, K.J., Hong, S.G., et al. (2019). Aberrant Clonal Hematopoiesis following Lentiviral Vector Transduction of HSPCs in a Rhesus Macaque. Mol. Ther. *27*, 1074–1086. https://doi.org/10.1016/j.ymthe.2019.04.003.

18. Heckl, D., Schwarzer, A., Haemmerle, R., Steinemann, D., Rudolph, C., Skawran, B., Knoess, S., Krause, J., Li, Z., Schlegelberger, B., et al. (2012). Lentiviral vector induced insertional haploinsufficiency of Ebf1 causes murine leukemia. Mol. Ther. *20*, 1187–1195. https://doi.org/10.1038/mt.2012.59.

19. Yin, H., and Wei, X. (2025). The design of retroviral vectors used in the CAR-T products, risk management, and future perspective. MedComm *6*, e70067. https://doi.org/10.1002/mco2.70067.

20. Kaiser, C.W., Rouchka, E.C., and Smith, M.L. (2026). Adaptation of lentiviral vectors for viral gene therapy and their impact on host cell biology. J. Transl. Med. *24*, 272. https://doi.org/10.1186/s12967-025-07626-5.

21. Duncan, C.N., Bledsoe, J.R., Grzywacz, B., Beckman, A., Bonner, M., Eichler, F.S., Kühl, J.S., Harris, M.H., Slauson, S., Colvin, R.A., et al. (2024). Hematologic Cancer after Gene Therapy for Cerebral Adrenoleukodystrophy. N. Engl. J. Med. *391*, 1287–1301. https://doi.org/10.1056/NEJMoa2405541.

22. Ozdemirli, M., Loughney, T.M., Deniz, E., Chahine, J.J., Albitar, M., Pittaluga, S., Sadigh, S., Armand, P., Uren, A., and Anderson, K.C. (2024). Indolent CD4+ CAR T-Cell Lymphoma after Cilta-cel CAR T-Cell Therapy. N. Engl. J. Med. *390*, 2074–2082. https://doi.org/10.1056/NEJMoa2401530.

23. Kobbe, G., Brüggemann, M., Baermann, B.N., Wiegand, L., Trautmann, H., Yousefian, S., Libertini, S., Menssen, H.D., Maier, H.J., Ulrich, P., et al. (2024). Aggressive Lymphoma after CD19 CAR T-Cell Therapy. N. Engl. J. Med. *391*, 1217–1226. https://doi.org/10.1056/NEJMoa2402730.

24. Guiraud, V., Denis, J.A., Benhafoun, G., Ablin, E., Sayon, S., Souchet, L., Azar, N., Grenier, A., Metz, C., Legrand, R., et al. (2025). Longitudinal analysis of lentiviral and retroviral chimeric antigen receptors' integration sites reveals distinct clonal evolutionary patterns. Br. J. Haematol. *206*, 1173–1177. https://doi.org/10.1111/bjh.20020.

25. Jadlowsky, J.K., Hexner, E.O., Marshall, A., Grupp, S.A., Frey, N.V., Riley, J.L., Veloso, E., McConville, H., Rogal, W., Czuczman, C., et al. (2025). Long-term safety of lentiviral or gammaretroviral gene-modified T cell therapies. Nat. Med. *31*, 1134–1144. https://doi.org/10.1038/s41591-024-03478-6.

26. Charrier, S., Ferrand, M., Zerbato, M., Précigout, G., Viornery, A., Bucher-Laurent, S., Benkhelifa-Ziyyat, S., Merten, O.W., Perea, J., and Galy, A. (2011). Quantification of lentiviral vector copy numbers in individual hematopoietic colony-forming cells shows vector dose-dependent effects on the frequency and level of transduction. Gene Ther. *18*, 479–487. https://doi.org/10.1038/gt.2010.163.

27. Kim, J., Park, M., Baek, G., Kim, J.I., Kwon, E., Kang, B.C., Kim, J.I., and Kang, H.J. (2023). Tagmentation-based analysis reveals the clonal behavior of CAR-T cells in association with lentivector integration sites. Mol. Ther. Oncolytics *30*, 1–13. https://doi.org/10.1016/j.omto.2023.05.004.

28. van Haasteren, J., Munis, A.M., Gill, D.R., and Hyde, S.C. (2021). Genome-wide integration site detection using Cas9 enriched amplification-free long-range sequencing. Nucleic Acids Res. *49*, e16. https://doi.org/10.1093/nar/gkaa1152.

29. Kosicki, M., Tomberg, K., and Bradley, A. (2018). Repair of double-strand breaks induced by CRISPR-Cas9 leads to large deletions and complex rearrangements. Nat. Biotechnol. *36*, 765–771. https://doi.org/10.1038/nbt.4192.

30. Kaiser, C.W., Elliott, E.M., Mehs, G.S., Mroczkowska, J.E., Murry, C.S., Jain, A., Ferguson, M., Garfall, A.L., Bushman, F.D., Fraietta, J.A., Rouchka, E.C., and Smith, M.L. (2026). LVV SMRTcap reveals extensive proviral variation in lentiviral vector-transduced CAR T cells. Mol. Ther. Advances, 201863. https://doi.org/10.1016/j.omta.2026.201863.

31. Dropulić, B. (2017). Reference Standards for Gene and Cell Therapy Products. Mol. Ther. *25*, 1259–1260. https://doi.org/10.1016/j.ymthe.2017.05.002.

32. White, M., Whittaker, R., Gándara, C., and Stoll, E.A. (2017). A Guide to Approaching Regulatory Considerations for Lentiviral-Mediated Gene Therapies. Hum. Gene Ther. Methods *28*, 163–176. https://doi.org/10.1089/hgtb.2017.096.

33. He, H.J., He, Z., Lund, S.P., Turner, L., Fan, Y., Qiu, Y., Corney, D.C., Dropulic, B., Orentas, R., Slessareva, O., et al. (2025). Interlaboratory assessment of candidate reference materials for lentiviral vector copy number and integration site measurements. Mol. Ther. Methods Clin. Dev. *33*, 101472. https://doi.org/10.1016/j.omtm.2025.101472.

34. Zhao, Y., Stepto, H., and Schneider, C.K. (2017). Development of the First World Health Organization Lentiviral Vector Standard: Toward the Production Control and Standardization of Lentivirus-Based Gene Therapy Products. Hum. Gene Ther. Methods *28*, 205–214. https://doi.org/10.1089/hgtb.2017.078.

35. Zhao, Y., Traylen, C., and Rigsby, P. (2019). Report on a Collaborative Study for the Proposed WHO 1st International Reference Panel (19/158) for the Quantitation of Lentiviral Vector Integration Copy Numbers (World Health Organization), p. 84.

36. He, Z., Kwee, E.J., Cleveland, M.H., Cole, K.D., Lin-Gibson, S., and He, H.J. (2023). Quantitation and integrity evaluation of RNA genome in lentiviral vectors by direct reverse transcription-droplet digital PCR (direct RT-ddPCR). Sci. Rep. *13*, 14470. https://doi.org/10.1038/s41598-023-41644-x.

37. Paugh, B.S., Baranyi, L., Roy, A., He, H.J., Harris, L., Cole, K.D., Artlip, M., Raimund, C., Langan, P.S., Jana, S., et al. (2021). Reference standards for accurate validation and optimization of assays that determine integrated lentiviral vector copy number in transduced cells. Sci. Rep. *11*, 389. https://doi.org/10.1038/s41598-020-79698-w.

38. Wilson, A., Dasyam, N., O'Hagan, F., Farrell, D., Turner, C., Jay, A., Schmidt, A., Beddow, R., Lillis, T., Chin, T., et al. (2025). Jurkat T-cell lines exhibit marked genomic instability affecting karyotype, mutational profile, gene expression, immunophenotype and function. Sci. Rep. *15*, 22426. https://doi.org/10.1038/s41598-025-95903-0.

39. Ciuffi, A. (2008). Mechanisms governing lentivirus integration site selection. Curr. Gene Ther. *8*, 419–429. https://doi.org/10.2174/156652308786848021.

40. Gioia, L., Siddique, A., Head, S.R., Salomon, D.R., and Su, A.I. (2018). A genome-wide survey of mutations in the Jurkat cell line. BMC Genom. *19*, 334. https://doi.org/10.1186/s12864-018-4718-6.

41. He, Z., Cole, K.D., and He, H.J. (2025). A novel immortalization method for immortalizing human primary CD8(+) T cells by inserting a single copy of human telomerase reverse transcriptase via CRISPR/Cas9. Tissue Cell *95*, 102908. https://doi.org/10.1016/j.tice.2025.102908.

42. Behera, S., LeFaive, J., Orchard, P., Mahmoud, M., Paulin, L.F., Farek, J., Soto, D.C., Parker, S.C.J., Smith, A.V., Dennis, M.Y., et al. (2023). FixItFelix: improving genomic analysis by fixing reference errors. Genome Biol. *24*, 31. https://doi.org/10.1186/s13059-023-02863-7.

43. Johnson, N.M., Alvarado, A.F., Moffatt, T.N., Edavettal, J.M., Swaminathan, T.A., and Braun, S.E. (2021). HIV-based lentiviral vectors: origin and sequence differences. Mol. Ther. Methods Clin. Dev. *21*, 451–465. https://doi.org/10.1016/j.omtm.2021.03.018.

44. Danecek, P., Bonfield, J.K., Liddle, J., Marshall, J., Ohan, V., Pollard, M.O., Whitwham, A., Keane, T., McCarthy, S.A., Davies, R.M., and Li, H. (2021). Twelve years of SAMtools and BCFtools. Gigascience *10*, giab008. https://doi.org/10.1093/gigascience/giab008.

45. Li, H. (2018). Minimap2: pairwise alignment for nucleotide sequences. Bioinformatics *34*, 3094–3100. https://doi.org/10.1093/bioinformatics/bty191.

46. Rausch, T., Zichner, T., Schlattl, A., Stütz, A.M., Benes, V., and Korbel, J.O. (2012). DELLY: structural variant discovery by integrated paired-end and split-read analysis. Bioinformatics *28*, i333–i339. https://doi.org/10.1093/bioinformatics/bts378.

**OMTA, Volume *34***

## Supplemental information

# Long-read genome sequencing reveals complex variability in lentiviral provirus insertions in clonal CD19 CAR-T VCN reference cell lines

**Zhiyong He, Jennifer H. McDaniel, Linhua Tian, Mahir Mohiuddin, Ningchun Xu, Lili Wang, Sheng Lin-Gibson, Justin M. Zook, and Hua-Jun He**

**Supplemental Text**

Observed integrated sequence for all integrations sites (IS) and expected proviral sequence.

```
>IS1_VCN1-VCN2-VCN3-VCN4-VCN5_chr17:78730345
GAAACTGCTAGAGATTTTCCACACTGACTAAAAGGGTCTGAGGGATCTCTAGTTACCAGAGTCACACAACAGACGGGCA
CACACTACTTGAAGCACTCAAGGCAAGCTTTATTGAGGCTTAAGCAGTGGGTTCCCTAGTTAGCCAGAGAGCTCCCAGG
CTCAGATCTGGTCTAACCAGAGAGACCCAGTACAAGCAAAAAGCAGATCTTATCTTCGTTGGGAGTGAATTAGCCCTTC
CAGTCCCCCCTTTTCTTTTAAAAAGTGGCTAAGATCTACAGCTGCCTTGTAAGTCATTGGTCTTAAATTATCAGTCCTG
CTCCTCTGCCACAAAGTGCACGCAGTTGCCGGCCGGGTCGCGCAGGGCGAACTCCCGCCCCCACGGCTGCTCGCCGATC
TCGGTCATGGCCGGCCCGGAGGCGTCCCGGAAGTTCGTGGACACGACCTCCGACCACTCGGCGTACAGCTCGTCCAGGC
CGCGCACCCACACCCAGGCCAGGGTGTTGTCCGGCACCACCTGGTCCTGGACCGCGCTGATGAACAGGGTCACGTCGTC
CCGGACCACACCGGCGAAGTCGTCCTCCACGAAGTCCCGGGAGAACCCGAGCCGGTCGGTCCAGAACTCGACCGCTCCG
GCGACGTCGCGCGCGGTGAGCACCGGAACGGCACTGGTCAACTTGGCCATAGGACCGGGGTTTTCTTCCACGTCTCCTG
CTTGCTTTAACAGAGAGAAGTTCGTGGCTCCAGAACCCTTGTACAGCTCGTCCATGCCGAGAGTGATCCCGGCGGCGGT
CACGAACTCCAGCAGGACCATGTGATCGCGCTTCTCGTTGGGGTCTTTGCTCAGGGCGGACTGGGTGCTCAGGTAGTGG
TTGTCGGGCAGCAGCACGGGGCCGTCGCCGATGGGGGTGTTCTGCTGGTAGTGGTCGGCGAGCTGCACGCTGCCGTCCT
CGATGTTGTGGCGGATCTTGAAGTTCACCTTGATGCCGTTCTTCTGCTTGTCGGCCATGATATAGACGTTGTGGCTGTT
GTAGTTGTACTCCAGCTTGTGCCCCAGGATGTTGCCGTCCTCCTTGAAGTCGATGCCCTTCAGCTCGATGCGGTTCACC
AGGGTGTCGCCCTCGAACTTCACCTCGGCGCGGGTCTTGTAGTTGCCGTCGTCCTTGAAGAAGATGGTGCGCTCCTGGA
CGTAGCCTTCGGGCATGGCGGACTTGAAGAAGTCGTGCTGCTTCATGTGGTCGGGGTAGCGGCTGAAGCACTGCACGCC
GTAGGTCAGGGTGGTCACGAGGGTGGGCCAGGGCACGGGCAGCTTGCCGGTGGTGCAGATGAACTTCAGGGTCAGCTTG
CCGTAGGTGGCATCGCCCTCGCCCTCGCCGGACACGCTGAACTTGTGGCCGTTTACGTCGCCGTCCAGCTCGACCAGGA
TGGGCACCACCCCGGTGAACAGCTCCTCGCCCTTGCTCACCATGGTGGCAAGCTTGGGCTGCAGGTCGAAAGGCCCGGA
GATGAGGAAGAGGAGAACAGCGCGGCAGACGTGCGCTTTTGAAGCGTGCAGAATGCCGGGCCTCCGGAGGACCTTCGGG
CGCCCGCCCCGCCCCTGAGCCCGCCCCTGAGCCCGCCCCCGGACCCACCCCTTCCCAGCCTCTGAGCCCAGAAAGCGAA
GGAGCAAAGCTGCTATTGGCCGCTGCCCCAAAGGCCTACCCGCTTCCATTGCTCAGCGGTGCTGTCCATCTGCACGAGA
CTAGTGAGACGTGCTACTTCCATTTGTCACGTCCTGCACGACGCGAGCTGCGGGGCGGGGGGGAACTTCCTGACTAGGG
GAGGAGTAGAAGGTGGCGCGAAGGGGCCACCAAAGAACGGAGCCGGTTGGCGCCTACCGGTGGATGTGGAATGTGTGCG
AGGCCAGAGGCCACTTGTGTAGCGCCAAGTGCCCAGCGGGGCTGCTAAAGCGCATGCTCCAGACTGCCTTGGGAAAAGC
GCCTCCCCTACCCGGTAGAATTGGTACCAGGCGGGGAGGCGGCCCAAAGGGAGATCCGACTCGTCTGAGGGCGAAGGCG
AAGACGCGGAAGAGGCCGCAGAGCCGGCAGCAGGCCGCGGGAAGGAAGGTCCGCTGGATTGAGGGCCGAAGGGACGTAG
CAGAAGGACGTCCCGCGCAGAATCCAGGTGGCAACACAGGCGAGCAGCCAAGGAAAGGACGATGATTTCCCCGACAACA
CCACGGAATTGTCAGTGCCCAACAGCCGAGCCCCTGTCCAGCAGCGGGCAAGGCAGGCGGCGATGAGTTCCGCCGTGGC
AATAGGGAGGGGGAAAGCGAAAGTCCCGGAAAGGAGCTGACAGGTGGTGGCAATGCCCCAACCAGTGGGGGTTGCGTCA
GCAAACACAGTGCACACCACGCCACGTTGCCTGACAACGGGCCACAACTCCTCATAAAGAGACAGCAACCAGGATTTAT
ACAAGGAGGAGAAAATGAAAGCCATACGGGAAGCAATAGCATGATACAAAGGCATTAAAGCAGCGTATCCACATAGCGT
AAAAGGAGCAACATAGTTAAGAATACCAGTCAATCTTTCACAAATTTTGTAATCCAGAGGTTGATTTCTAGATTATTAG
CGAGGGGGCAGGGCCTGCATGTGAAGGGCGTCGTAGGTGTCCTTGGTGGCTGTACTGAGACCCTGGTAAAGGCCATCGT
GCCCCTTGCCCCTCCGGCGCTCGCCTTTCATCCCAATCTCACTGTAGGCCTCCGCCATCTTATCTTTCTGCAGTTCATT
GTACAGGCCTTCCTGAGGGTTCTTCCTTCTCGGCTTTCCCCCCATCTCAGGGTCCCGGCCACGTCTCTTGTCCAAAACA
TCGTACTCCTCTCTTCGTCCTAGATTGAGCTCGTTATAGAGCTGGTTCTGGCCCTGCTTGTACGCGGGGGCGTCTGCGC
TCCTGCTGAACTTCACTCTCAGTTCACATCCTCCTTCTTCTTCTTCTGGAAATCGGCAGCTACAGCCATCTTCCTCTTG
AGTAGTTTGTACTGGTCTCATAAATGGTTGTTTGAATATATACAGGAGTTTCTTTCTGCCCCGTTTGCTCCGGTAGGCG
GCGAAATCCCTGGGAGGGGCGTAGGGCTGGTAGTGCTTCCGGGTGGGGCCAGGCCTCCGGGGGGTCATGTTCATGTAGT
CGCTGTGCAGCAGTCTGCTCCGCTTGCTCCTCACCCAAAAGATGATGAAGGCCACTGTCACCAGCAGGCTGTAGCAGGC
CAGCACGCCGCCCACAACGACCAGCACCCAGAAATCACAGGCGAAGTCCAGCCCCCTCGTGTGCACTGCGCCCCCCGCC
GCTGGCCGGCACGCTTCTGGGCGCAGGGACAGGGGCTGCGACGCGATGGTGGGCGCCGGTGTTGGTGGTCGCGGCGCTG
GCGTCGTGGTAGAAGAAACGGTCACGCTGGTGCCCTGGCCCCAATAATCCATGGCGTAGCTGCCGCCGTAGTAGTAGTG
CTTGGCGCAGTAGTAGATGGCGGTGTCGTCGGTCTGCAGGCTGTTCATCTTCAGGAACACCTGGCTCTTGCTGTTGTCC
TTGATGATGGTCAGCCGGGACTTCAGGGCGCTGTTGTAGTAGGTTGTCTCGCTGCCCCAGATCACTCCCAGCCATTCCA
```

GGCCTTTCCGAGGAGGCTGTCTGATCCAGGACACGCCGTAATCAGGCAGGGACACTCCGCTGACTGTACAGGTCACGCT
CAGAGACTGAGATGGGGCCACCAGTCCAGGGCCAGACTCTTGCAGCTTCACTTCAGAACCGCCTCCGCCACTTCCTCCG
CCACCAGATCCTCCGCCTCCGGTGATTTCCAGCTTGGTGCCTCCGCCAAAGGTGTAAGGCAGGGTGTTGCCTTGCTGGC
AGAAGTAGGTAGCGATATCCTCTTGTTCCAGGTTGCTGATTGTCAGGCTGTAGTCGGTGCCAGAGCCGCTGCCAGAAAA
TCTGCTTGGCACGCCGCTGTGCAGTCTGCTGGTGTGGTAGATCAGCAGCTTCACGGTGCCGTCGGGTTTCTGCTGATAC
CAGTTCAGGTACTTGCTGATGTCCTGGCTGGCTCTACAGCTGATGGTCACTCTATCGCCCAGGCTGGCAGACAGGCTGC
TTGTGGTCTGGGTCATCTGGATGTCGGGCCGAGCGGCGTGGAGCAGAAGAGCCAGCGGAAGCAGCAGGGCGGTGACAGG
GAGGGCCATGGTGGCGGCTTCGAATCACGACACCTGAAATGGAAGAAAAAAACTTTGAACCACTGTCTGAGGCTTGAGA
ATGAACCAAGATCCAAACTCAAAAAGGGCAAATTCCAAGGAGAATTACATCAAGTGCCAAGCTGGCCTAACTTCAGTCT
CCACCCACTCAGTGTGGGGAAACTCCATCGCATAAAACCCCTCCCCCCAACCTAAAGACGACGTACTCCAAAAGCTCGA
GAACTAATCGAGGTGCCTGGACGGCGCCCGGTACTCCGTGGAGTCACATGAAGCGACGGCTGAGGACGGAAAGGCCCTT
TTCCTTTGTGTGGGTGACTCACCCGCCCGCTCTCCCGAGCGCCGCGTCCTCCATTTTGAGCTCCCTGCAGCAGGGCCGG
GAAGCGGCCATCTTTCCGCTCACGCAACTGGTGCCGACCGGGCCAGCCTTGCCGCCCAGGGCGGGGCGATACACGGCGG
CGCGAGGCCAGGCACCAGAGCAGGCCGGCCAGCTTGAGACTACCCCCGTCCGATTCTCGGTGGCCGCGCTCGCAGGCCT
CTGGGTTCTACGTTAGTGGGAGTTTAACGACGGTCCCTGGGATTCCCCAAGGCAGGGGCGAGTCCTTTTGTATGAATTA
CTCCATGGTAATAGCGATGACTAATACGTAGATGTACTGCCAAGTAGGAAAGTCCCATAAGGTCATGTACTGGGCATAA
TACTAGTATCGATAAAATTTTGAATTTTGTAATTTGTTTTTGTAATTCTTTAGTTTGTATGTCTGTTGCTATTATGTCT
ACTATTCTTTCCCCTGCACTGTACCCCCCAATCCCCCCTTTTCTTTTAAAAGTTAACCGATACCGTCGAGATCCGTTCA
CTAATCGAATGGATCTGTCTCTGTCTCTCTCTCCACCTTCTTCTTCTATTCCTTCGGGCCTGTCGGGTCCCCTCGGGGT
TGGGAGGTGGGTCTGAAACGATAATGGTGAATATCCCTGCCTAACTCTATTCACTATAGAAAGTACAGCAAAAACTATT
CTTAAACCTACCAAGCCTCCTACTATCATTATGAATAATTTTATATACCACAGCCAATTTGTTATGTTAAACCAATTCC
ACAAACTTGCCCATTTATCTAATTCCAATAATTCTTGTTCATTCTTTTCTTGCTGGTTTTGCGATTCTTCAATTAAGGA
GTGTATTAAGCTTGTGTAATTGTTAATTTCTCTGTCCCACTCCATCCAGGTCGTGTGATTCCAAATCTGTTCCAGAGAT
TTATTACTCCAACTAGCATTCCAAGGCACAGCAGTGGTGCAAATGAGTTTTCCAGAGCAACCCCAAATCCCCAGGAGCT
GTTGATCCTTTAGGTATCTTTCCACAGCCAGGATTCTTGCCTGGAGCTGCTTGATGCCCCAGACTGTGAGTTGCAACAG
ATGCTGTTGCGCCTCAATAGCCCTCAGCAAATTGTTCTGCTGCTGCACTATACCAGACAATAATTGTCTGGCCTGTACC
GTCAGCGTCATTGACGCTGCGCCCATAGTGCTTCCTGCTGCTCCCAAGAACCCAAGGAACAAAGCTCCTATTCCCACTG
CTCTTTTTTCTCTCTGCACCACTCTTCTCTTTGCCTTGGTGGGTGCTACTCCTAATGGTTCAATTTTTACTACTTTATA
TTTATATAATTCACTTCTCCAATTGTCCCTCATATCTCCTCCTCCAGGTCTGAAGATCAGTGGCCGCTTGCTGTGCGGT
GGTCTTACTTTTGTTTTGCTCTTCCTCTATCTTGTCTAAAGCTTCCTTGGTGTCTTTTATCTCTATCCTTTGATGCACA
CAATAGAGGGTTGCTACTGTATTATATAATGATCTAAGTTCTTCTGATCCTGTCTGAAGGGATGGTTGTAGCTGTCCCA
GTATTTGTCTACAGCCTTCTGATGTTTCTAACAGGCCAGGATTAACTGCGAATCGTTCTAGCTCCCTGCTTGCCCATAC
TATATGTTTTAATTTATATTTTTTCTTTCCCCCTGGCCTTAACCGAATTTTTTCCCATCGCGATCTAATTCTCCCCCGC
TTAATACTGACGCTCTCGCACCCATCTCTCTCCTTCTAGCCTCCGCTAGTCAAAATTTTTGGCGTACTCACCAGTCGCC
GCCCCTCGCCTCTTGCCGTGCGCGCTTCAGCAAGCCGAGTCCTGCGTCGAGAGAGCTCCTCTGGTTTCCCTTTCGCTTT
CAAGTCCCTGTTCGGGCGCCACTGCTAGAGATTTTCCACACTGACTAAAAGGGTCTGAGGGATCTCTAGTTACCAGAGT
CACACAACAGACGGGCACACACTACTTGAAGCACTCAAGGCAAGCTTTATTGAGGCTTAAGCAGTGGGTTCCCTAGTTA
GCCAGAGAGCTCCCAGGCTCAGATCTGGTCTAACCAGAGAGACCCAGTACAAGCAAAAAGCAGATCTTATCTTCGTTGG
GAGTGAATTAGCCCTTCCA
>IS2_VCN2_chr19:21610872
CCCCCTGCTAGAGATTTTCCACACTGACTAAAAGGGTCTGAGGGATCTCTAGTTACCAGAGTCACACAACAGACGGGCA
CACACTACTTGAAGCACTCAAGGCAAGCTTTATTGAGGCTTAAGCAGTGGGTTCCCTAGTTAGCCAGAGAGCTCCCAGG
CTCAGATCTGGTCTAACCAGAGAGACCCAGTACAAGCAAAAAGCAGATCTTATCTTCGTTGGGAGTGAATTAGCCCTTC
CAGTCCCCCCTTTTCTTTTAAAAAGTGGCTAAGATCTACAGCTGCCTTGTAAGTCATTGGTCTTAAATTATCAGTCCTG
CTCCTCTGCCACAAAGTGCACGCAGTTGCCGGCCGGGTCGCGCAGGGCGAACTCCCGCCCCCACGGCTGCTCGCCGATC
TCGGTCATGGCCGGCCCGGAGGCGTCCCGGAAGTTCGTGGACACGACCTCCGACCACTCGGCGTACAGCTCGTCCAGGC
CGCGCACCCACACCCAGGCCAGGGTGTTGTCCGGCACCACCTGGTCCTGGACCGCGCTGATGAACAGGGTCACGTCGTC
CCGGACCACACCGGCGAAGTCGTCCTCCACGAAGTCCCGGGAGAACCCGAGCCGGTCGGTCCAGAACTCGACCGCTCCG
GCGACGTCGCGCGCGGTGAGCACCGGAACGGCACTGGTCAACTTGGCCATAGGACCGGGGTTTTCTTCCACGTCTCCTG
CTTGCTTTAACAGAGAGAAGTTCGTGGCTCCAGAACCCTTGTACAGCTCGTCCATGCCGAGAGTGATTCCGGCGGCGGT
CACGAACTCCAGCAGGACCATGTGATCGCGCTTCTCGTTGGGGTCTTTGCTCAGGGCGGACTGGGTGCTCAGGTAGTGG

TTGTCGGGCAGCAGCACGGGGCCGTCGCCGATGGGGGTGTTCTGCTGGTAGTGGTCGGCGAGCTGCACGCTGCCGTCCT
CGATGTTGTGGCGGATCTTGAAGTTCACCTTGATGCCGTTCTTCTGCTTGTCGGCCATGATATAGACGTTGTGGCTGTT
GTAGTTGTACTCCAGCTTGTGCCCCAGGATGTTGCCGTCCTCCTTGAAGTCGATGCCCTTCAGCTCGATGCGGTTCACC
AGGGTGTCGCCCTCGAACTTCACCTCGGCGCGGGTCTTGTAGTTGCCGTCGTCCTTGAAGAAGATGGTGCGCTCCTGGA
CGTAGCCTTCGGGCATGGCGGACTTGAAGAAGTCGTGCTGCTTCATGTGGTCGGGGTAGCGGCTGAAGCACTGCACGCC
GTAGGTCAGGGTGGTCACGAGGGTGGGCCAGGGCACGGGCAGCTTGCCGGTGGTGCAGATGAACTTCAGGGTCAGCTTG
CCGTAGGTGGCATCGCCCTCGCCCTCGCCGGACACGCTGAACTTGTGGCCGTTTACGTCGCCGTCCAGCTCGACCAGGA
TGGGCACCACCCCGGTGAACAGCTCCTCGCCCTTGCTCACCATGGTGGCAAGCTTGGGCTGCAGGTCGAAAGGCCCGGA
GATGAGGAAGAGGAGAACAGCGCGGCAGACGTGCGCTTTTGAAGCGTGCAGAATGCCGGGCCTCCGGAGGACCTTCGGG
CGCCCGCCCCGCCCCTGAGCCCGCCCCTGAGCCCGCCCCCGGACCCACCCCTTCCCAGCCTCTGAGCCCAGAAAGCGAA
GGAGCAAAGCTGCTATTGGCCGCTGCCCCAAAGGCCTACCCGCTTCCATTGCTCAGCGGTGCTGTCCATCTGCACGAGA
CTAGTGAGACGTGCTACTTCCATTTGTCACGTCCTGCACGACGCGAGCTGCGGGGCGGGGGGGAACTTCCTGACTAGGG
GAGGAGTAGAAGGTGGCGCGAAGGGGCCACCAAAGAACGGAGCCGGTTGGCGCCTACCGGTGGATGTGGAATGTGTGCG
AGGCCAGAGGCCACTTGTGTAGCGCCAAGTGCCCAGCGGGGCTGCTAAAGCGCATGCTCCAGACTGCCTTGGGAAAAGC
GCCTCCCCTACCCGGTAGAATTGGTACCAGGCGGGGAGGCGGCCCAAAGGGAGATCCGACTCGTCTGAGGGCGAAGGCG
AAGACGCGGAAGAGGCCGCAGAGCCGGCAGCAGGCCGCGGGAAGGAAGGTCCGCTGGATTGAGGGCCGAAGGGACGTAG
CAGAAGGACGTCCCGCGCAGAATCCAGGTGGCAACACAGGCGAGCAGCCAAGGAAAGGACGATGATTTCCCCGACAACA
CCACGGAATTGTCAGTGCCCAACAGCCGAGCCCCTGTCCAGCAGCGGGCAAGGCAGGCGGCGATGAGTTCCGCCGTGGC
AATAGGGAGGGGGAAAGCGAAAGTCCCGGAAAGGAGCTGACAGGTGGTGGCAATGCCCCAACCAGTGGGGGTTGCGTCA
GCAAACACAGTGCACACCACGCCACGTTGCCTGACAACGGGCCACAACTCCTCATAAAGAGACAGCAACCAGGATTTAT
ACAAGGAGGAGAAAATGAAAGCCATACGGGAAGCAATAGCATGATACAAAGGGATTAAAGCAGCGTATCCACATAGCGT
AAAAGGAGCAACATAGTTAAGAATACCAGTCAATCTTTCACAAATTTTGTAATCCAGAGGTTGATTTCTAGATTATTAG
CGAGGGGGCAGGGCCTGCATGTGAAGGGCGTCGTAGGTGTCCTTGGTGGCTGTACTGAGACCCTGGTAAAGGCCATCGT
GCCCCTTGCCCCTCCGGCGCTCGCCTTTCATCCCAATCTCACTGTAGGCCTCCGCCATCTTATCTTTCTGCAGTTCATT
GTACAGGCCTTCCTGAGGGTTCTTCCTTCTCGGCTTTCCCCCCATCTCAGGGTCCCGGCCACGTCTCTTGTCCAAAACA
TCGTACTCCTCTCTTCGTCCTAGATTGAGCTCGTTATAGAGCTGGTTCTGGCCCTGCTTGTACGCGGGGGCGTCTGCGC
TCCTGCTGAACTTCACTCTCAGTTCACATCCTCCTTCTTCTTCTTCTGGAAATCGGCAGCTACAGCCATCTTCCTCTTG
AGTAGTTTGTACTGGTCTCATAAATGGTTGTTTGAATATATACAGGAGTTTCTTTCTGCCCCGTTTGCTCCGGTAGGCG
GCGAAATCCCTGGGAGGGGCGTAGGGCTGGTAGTGCTTCCGGGTGGGGCCAGGCCTCCGGGGGGTCATGTTCATGTAGT
CGCTGTGCAGCAGTCTGCTCCGCTTGCTCCTCACCCAAAAGATGATGAAGGCCACTGTCACCAGCAGGCTGTAGCAGGC
CAGCACGCCGCCCACAACGACCAGCACCCAGAAATCACAGGCGAAGTCCAGCCCCCTCGTGTGCACTGCGCCCCCCGCC
GCTGGCCGGCACGCTTCTGGGCGCAGGGACAGGGGCTGCGACGCGATGGTGGGCGCCGGTGTTGGTGGTCGCGGCGCTG
GCGTCGTGGTAGAAGAAACGGTCACGCTGGTGCCCTGGCCCCAATAATCCATGGCGTAGCTGCCGCCGTAGTAGTAGTG
CTTGGCGCAGTAGTAGATGGCGGTGTCGTCGGTCTGCAGGCTGTTCATCTTCAGGAACACCTGGCTCTTGCTGTTGTCC
TTGATGATGGTCAGCCGGGACTTCAGGGCGCTGTTGTAGTAGGTTGTCTCGCTGCCCCAGATCACTCCCAGCCATTCCA
GGCCTTTCCGAGGAGGCTGTCTGATCCAGGACACGCCGTAATCAGGCAGGGACACTCCGCTGACTGTACAGGTCACGCT
CAGAGACTGAGATGGGGCCACCAGTCCAGGGCCAGACTCTTGCAGCTTCACTTCAGAACCGCCTCCGCCACTTCCTCCG
CCACCAGATCCTCCGCCTCCGGTGATTTCCAGCTTGGTGCCTCCGCCAAAGGTGTAAGGCAGGGTGTTGCCTTGCTGGC
AGAAGTAGGTAGCGATATCCTCTTGTTCCAGGTTGCTGATTGTCAGGCTGTAGTCGGTGCCAGAGCCGCTGCCAGAAAA
TCTGCTTGGCACGCCGCTGTGCAGTCTGCTGGTGTGGTAGATCAGCAGCTTCACGGTGCCGTCGGGTTTCTGCTGATAC
CAGTTCAGGTACTTGCTGATGTCCTGGCTGGCTCTACAGCTGATGGTCACTCTATCGCCCAGGCTGGCAGACAGGCTGC
TTGTGGTCTGGGTCATCTGGATGTCGGGCCGAGCGGCGTGGAGCAGAAGAGCCAGCGGAAGCAGCAGGGCGGTGACAGG
GAGGGCCATGGTGGCGGCTTCGAATCACGACACCTGAAATGGAAGAAAAAAACTTTGAACCACTGTCTGAGGCTTGAGA
ATGAACCAAGATCCAAACTCAAAAAGGGCAAATTCCAAGGAGAATTACATCAAGTGCCAAGCTGGCCTAACTTCAGTCT
CCACCCACTCAGTGTGGGGAAACTCCATCGCATAAAACCCCTCCCCCCAACCTAAAGACGACGTACTCCAAAAGCTCGA
GAACTAATCGAGGTGCCTGGACGGCGCCCGGTACTCCGTGGAGTCACATGAAGCGACGGCTGAGGACGGAAAGGCCCTT
TTCCTTTGTGTGGGTGACTCACCCGCCCGCTCTCCCGAGCGCCGCGTCCTCCATTTTGAGCTCCCTGCAGCAGGGCCGG
GAAGCGGCCATCTTTCCGCTCACGCAACTGGTGCCGACCGGGCCAGCCTTGCCGCCCAGGGCGGGGCGATACACGGCGG
CGCGAGGCCAGGCACCAGAGCAGGCCGGCCAGCTTGAGACTACCCCCGTCCGATTCTCGGTGGCCGCGCTCGCAGGCCC
CGCCTCGCCGAACATGTGCGCTGGGACGCACGGGCCCCGTCGCCGCCCGCGGCCCCAAAAACCGAAATACCAGTGTGCA
GATCTTGGCCCGCATTTACAAGACTATCTTGCCAGAAAAAAAGCGTCGCAGCAGGTCATCAAAAATTTTAAATGGCTAG

AGACTTATCGAAAGCAGCGAGACAGGCGCGAAGGTGCCACCAGATTCGCACGCGGCGGCCCCAGCGCCCAAGCCAGGCC
TCAACTCAAGCACGAGGCGAAGGGGCTCCTTAAGCGCAAGGCCTCGAACTCTCCCACCCACTTCCAACCCGAAGCTCGG
GATCAAGAATCACGTACTGCAGCCAGGGGCGTGGAAGTAATTCAAGGCACGCAAGGGCCATAACCCGTAAAGAGGCCAG
GCCCGCGGGAACCACACACGGCACTTACCTGTGTTCTGGCGGCAAACCCGTTGCGAAAAAGAACGTTCACGGCGACTAC
TGCACTTATATACGGTTCTCCCCCACCCTCGGGAAAAAGGCGGAGCCAGTACACGACATCACTTTCCCAGTTTACCCCG
CGCCACCTTCTCTAGGCACCGGTTCAATTGCCGACCCCTCCCCCCAACTTCTCGGGGACTGTGGGCGATGTGCGCTCTG
CCCACTGACGGGCACCGGAGCCTCACGCATGCTCTTCTCCACCTCAGTGATGACGAGAGCGGGCGGGTGAGGGGGCGGG
AACGCAGCGATCTCTGGGTTCTACGTTAGTGGGAGTTTAACGACGGTCCCTGGGATTCCCCAAGGCAGGGGCGAGTCCT
TTTGTATGAATTACTCCATGGTAATAGCGATGACTAATACGTAGATGTACTGCCAAGTAGGAAAGTCCCATAAGGTCAT
GTACTGGGCATAATACTAGTATCGATAAAATTTTGAATTTTGTAATTTGTTTTTGTAATTCTTTAGTTTGTATGTCTAA
TGCTATTATGTCTACTATTCTTTCCCCTGCACTGTACCCCCCAATCCCCCCTTTTCTTTTAAAAGTTAACCGATACCGT
CGAGATCCGTTCACTAATCGAATGGATCTGTCTCTGTCTCTCTCTCCACCTTCTTCTTCTATTCCTTCGGGCCTGTCGG
GTCCCCTCGGGGTTGGGAGGTGGGTCTGAAACGATAATGGTGAATATCCCTGCCTAACTCTATTCACTATAGAAAGTAC
AGCAAAAACTATTCTTAAACCTACCAAGCCTCCTACTATCATTATGAATAATTTTATATACCACAGCCAATTTGTTATG
TTAAACCAATTCCACAAACTTGCCCATTTATCTAATTCCAATAATTCTTGTTCATTCTTTTCTTGCTGGTTTTGCGATT
CTTCAATTAAGGAGTGTATTAAGCTTGTGTAATTGTTAATTTCTCTGTCCCACTCCATCCAGGTCGTGTGATTCCAAAT
CTGTTCCAGAGATTTATTACTCCAACTAGCATTCCAAGGCACAGCAGTGGTGCAAATGAGTTTTCCAGAGCAACCCCAA
ATCCCCAGGAGCTGTTGATCCTTTAGGTATCTTTCCACAGCCAGGATTCTTGCCTGGAGCTGCTTGATGCCCCAGACTG
TGAGTTGCAACAGATGCTGTTGCGCCTCAATAGCCCTCAGCAAATTGTTCTGCTGCTGCACTATACCAGACAATAATTG
TCTGGCCTGTACCGTCAGCGTCATTGACGCTGCGCCCATAGTGCTTCCTGCTGCTCCCAAGAACCCAAGGAACAAAGCT
CCTATTCCCACTGCTCTTTTTTCTCTCTGCACCACTCTTCTCTTTGCCTTGGTGGGTGCTACTCCTAATGGTTCAATTT
TTACTACTTTATATTTATATAATTCACTTCTCCAATTGTCCCTCATATCTCCTCCTCCAGGTCTGAAGATCAGTGGCCG
CTTGCTGTGCGGTGGTCTTACTTTTGTTTTGCTCTTCCTCTATCTTGTCTAAAGCTTCCTTGGTGTCTTTTATCTCTAT
CCTTTGATGCACACAATAGAGGGTTGCTACTGTATTATATAATGATCTAAGTTCTTCTGATCCTGTCTGAAGGGATGGT
TGTAGCTGTCCCAGTATTTGTCTACAGCCTTCTGATGTTTCTAACAGGCCAGGATTAACTGCGAATCGTTCTAGCTCCC
TGCTTGCCCATACTATATGTTTTAATTTATATTTTTTCTTTCCCCCTGGCCTTAACCGAATTTTTTCCCATCGCGATCT
AATTCTCCCCCGCTTAATACTGACGCTCTCGCACCCATCTCTCTCCTTCTAGCCTCCGCTAGTCAAAATTTTTGGCGTA
CTCACCAGTCGCCGCCCCTCGCCTCTTGCCGTGCGCGCTTCAGCAAGCCGAGTCCTGCGTCGAGAGAGCTCCTCTGGTT
TCCCTTTCGCTTTCAAGTCCCTGTTCGGGCGCCACTGCTAGAGATTTTCCACACTGACTAAAAGGGTCTGAGGGATCTC
TAGTTACCAGAGTCACACAACAGACGGGCACACACTACTTGAAGCACTCAAGGCAAGCTTTATTGAGGCTTAAGCAGTG
GGTTCCCTAGTTAGCCAGAGAGCTCCCAGGCTCAGATCTGGTCTAACCAGAGAGACCCAGTACAAGCAAAAAGCAGATC
TTATCTTCGTTGGGAGTGAATTAGCCCTTCCA
>IS3_VCN3_chr1:23307863
ACAGGTGCTAGAGATTTTCCACACTGACTAAAAGGGTCTGAGGGATCTCTAGTTACCAGAGTCACACAACAGACGGGCA
CACACTACTTGAAGCACTCAAGGCAAGCTTTATTGAGGCTTAAGCAGTGGGTTCCCTAGTTAGCCAGAGAGCTCCCAGG
CTCAGATCTGGTCTAACCAGAGAGACCCAGTACAAGCAAAAAGCAGATCTTATCTTCGTTGGGAGTGAATTAGCCCTTC
CAGTCCCCCCTTTTCTTTTAAAAAGTGGCTAAGATCTACAGCTGCCTTGTAAGTCATTGGTCTTAAATTATCAGTCCTG
CTCCTCTGCCACAAAGTGCACGCAGTTGCCGGCCGGGTCGCGCAGGGCGAACTCCCGCCCCCACGGCTGCTCGCCGATC
TCGGTCATGGCCGGCCCGGAGGCGTCCCGGAAGTTCGTGGACACGACCTCCGACCACTCGGCGTACAGCTCGTCCAGGC
CGCGCACCCACACCCAGGCCAGGGTGTTGTCCGGCACCACCTGGTCCTGGACCGCGCTGATGAACAGGGTCACGTCGTC
CCGGACCACACCGGCGAAGTCGTCCTCCACGAAGTCCCGGGAGAACCCGAGCCGGTCGGTCCAGAACTCGACCGCTCCG
GCGACGTCGCGCGCGGTGAGCACCGGAACGGCACTGGTCAACTTGGCCATAGGACCGGGGTTTTCTTCCACGTCTCCTG
CTTGCTTTAACAGAGAGAAGTTCGTGGCTCCAGAACCCTTGTACAGCTCGTCCATGCCGAGAGTGATCCCGGCGGCGGT
CACGAACTCCAGCAGGACCATGTGATCGCGCTTCTCGTTGGGGTCTTTGCTCAGGGCGGACTGGGTGCTCAGGTAGTGG
TTGTCGGGCAGCAGCACGGGGCCGTCGCCGATGGGGGTGTTCTGCTGGTAGTGGTCGGCGAGCTGCACGCTGCCGTCCT
CGATGTTGTGGCGGATCTTGAAGTTCACCTTGATGCCGTTCTTCTGCTTGTCGGCCATGATATAGACGTTGTGGCTGTT
GTAGTTGTACTCCAGCTTGTGCCCCAGGATGTTGCCGTCCTCCTTGAAGTCGATGCCCTTCAGCTCGATGCGGTTCACC
AGGGTGTCGCCCTCGAACTTCACCTCGGCGCGGGTCTTGTAGTTGCCGTCGTCCTTGAAGAAGATGGTGCGCTCCTGGA
CGTAGCCTTCGGGCATGGCGGACTTGAAGAAGTCGTGCTGCTTCATGTGGTCGGGGTAGCGGCTGAAGCACTGCACGCC
GTAGGTCAGGGTGGTCACGAGGGTGGGCCAGGGCACGGGCAGCTTGCCGGTGGTGCAGATGAACTTCAGGGTCAGCTTG
CCGTAGGTGGCATCGCCCTCGCCCTCGCCGGACACGCTGAACTTGTGGCCGTTTACGTCGCCGTCCAGCTCGACCAGGA

TGGGCACCACCCCGGTGAACAGCTCCTCGCCCTTGCTCACCATGGTGGCAAGCTTGGGCTGCAGGTCGAAAGGCCCGGA
GATGAGGAAGAGGAGAACAGCGCGGCAGACGTGCGCTTTTGAAGCGTGCAGAATGCCGGGCCTCCGGAGGACCTTCGGG
CGCCCGCCCCGCCCCTGAGCCCGCCCCTGAGCCCGCCCCCGGACCCACCCCTTCCCAGCCTCTGAGCCCAGAAAGCGAA
GGAGCAAAGCTGCTATTGGCCGCTGCCCCAAAGGCCTACCCGCTTCCATTGCTCAGCGGTGCTGTCCATCTGCACGAGA
CTAGTGAGACGTGCTACTTCCATTTGTCACGTCCTGCACGACGCGAGCTGCGGGGCGGGGGGGAACTTCCTGACTAGGG
GAGGAGTAGAAGGTGGCGCGAAGGGGCCACCAAAGAACGGAGCCGGTTGGCGCCTACCGGTGGATGTGGAATGTGTGCG
AGGCCAGAGGCCACTTGTGTAGCGCCAAGTGCCCAGCGGGGCTGCTAAAGCGCATGCTCCAGACTGCCTTGGGAAAAGC
GCCTCCCCTACCCGGTAGAATTGGTACCAGGCGGGGAGGCGGCCCAAAGGGAGATCCGACTCGTCTGAGGGCGAAGGCG
AAGACGCGGAAGAGGCCGCAGAGCCGGCAGCAGGCCGCGGGAAGGAAGGTCCGCTGGATTGAGGGCCGAAGGGACGTAG
CAGAAGGACGTCCCGCGCAGAATCCAGGTGGCAACACAGGCGAGCAGCCAAGGAAAGGACGATGATTTCCCCGACAACA
CCACGGAATTGTCAGTGCCCAACAGCCGAGCCCCTGTCCAGCAGCGGGCAAGGCAGGCGGCGATGAGTTCCGCCGTGGC
AATAGGGAGGGGGAAAGCGAAAGTCCCGGAAAGGAGCTGACAGGTGGTGGCAATGCCCCAACCAGTGGGGGTTGCGTCA
GCAAACACAGTGCACACCACGCCACGTTGCCTGACAACGGGCCACAACTCCTCATAAAGAGACAGCAACCAGGATTTAT
ACAAGGAGGAGAAAATGAAAGCCATACGGGAAGCAATAGCATGATACAAAGGCATTAAAGCAGCGTATCCACATAGCGT
AAAAGGAGCAACATAGTTAAGAATACCAGTCAATCTTTCACAAATTTTGTAATCCAGAGGTTGATTTCTAGATTATTAG
CGAGGGGGCAGGGCCTGCATGTGAAGGGCGTCGTAGGTGTCCTTGGTGGCTGTACTGAGACCCTGGTAAAGGCCATCGT
GCCCCTTGCCCCTCCGGCGCTCGCCTTTCATCCCAATCTCACTGTAGGCCTCCGCCATCTTATCTTTCTGCAGTTCATT
GTACAGGCCTTCCTGAGGGTTCTTCCTTCTCGGCTTTCCCCCCATCTCAGGGTCCCGGCCACGTCTCTTGTCCAAAACA
TCGTACTCCTCTCTTCGTCCTAGATTGAGCTCGTTATAGAGCTGGTTCTGGCCCTGCTTGTACGCGGGGGCGTCTGCGC
TCCTGCTGAACTTCACTCTCAGTTCACATCCTCCTTCTTCTTCTTCTGGAAATCGGCAGCTACAGCCATCTTCCTCTTG
AGTAGTTTGTACTGGTCTCATAAATGGTTGTTTGAATATATACAGGAGTTTCTTTCTGCCCCGTTTGCTCCGGTAGGCG
GCGAAATCCCTGGGAGGGGCGTAGGGCTGGTAGTGCTTCCGGGTGGGGCCAGGCCTCCGGGGGGTCATGTTCATGTAGT
CGCTGTGCAGCAGTCTGCTCCGCTTGCTCCTCACCCAAAAGATGATGAAGGCCACTGTCACCAGCAGGCTGTAGCAGGC
CAGCACGCCGCCCACAACGACCAGCACCCAGAAATCACAGGCGAAGTCCAGCCCCCTCGTGTGCACTGCGCCCCCCGCC
GCTGGCCGGCACGCTTCTGGGCGCAGGGACAGGGGCTGCGACGCGATGGTGGGCGCCGGTGTTGGTGGTCGCGGCGCTG
GCGTCGTGGTAGAAGAAACGGTCACGCTGGTGCCCTGGCCCCAATAATCCATGGCGTAGCTGCCGCCGTAGTAGTAGTG
CTTGGCGCAGTAGTAGATGGCGGTGTCGTCGGTCTGCAGGCTGTTCATCTTCAGGAACACCTGGCTCTTGCTGTTGTCC
TTGATGATGGTCAGCCGGGACTTCAGGGCGCTGTTGTAGTAGGTTGTCTCGCTGCCCCAGATCACTCCCAGCCATTCCA
GGCCTTTCCGAGGAGGCTGTCTGATCCAGGACACGCCGTAATCAGGCAGGGACACTCCGCTGACTGTACAGGTCACGCT
CAGAGACTGAGATGGGGCCACCAGTCCAGGGCCAGACTCTTGCAGCTTCACTTCAGAACCGCCTCCGCCACTTCCTCCG
CCACCAGATCCTCCGCCTCCGGTGATTTCCAGCTTGGTGCCTCCGCCAAAGGTGTAAGGCAGGGTGTTGCCTTGCTGGC
AGAAGTAGGTAGCGATATCCTCTTGTTCCAGGTTGCTGATTGTCAGGCTGTAGTCGGTGCCAGAGCCGCTGCCAGAAAA
TCTGCTTGGCACGCCGCTGTGCAGTCTGCTGGTGTGGTAGATCAGCAGCTTCACGGTGCCGTCGGGTTTCTGCTGATAC
CAGTTCAGGTACTTGCTGATGTCCTGGCTGGCTCTACAGCTGATGGTCACTCTATCGCCCAGGCTGGCAGACAGGCTGC
TTGTGGTCTGGGTCATCTGGATGTCGGGCCGAGCGGCGTGGAGCAGAAGAGCCAGCGGAAGCAGCAGGGCGGTGACAGG
GAGGGCCATGGTGGCGGCTTCGAATCACGACACCTGAAATGGAAGAAAAAAACTTTGAACCACTGTCTGAGGCTTGAGA
ATGAACCAAGATCCAAACTCAAAAAGGGCAAATTCCAAGGAGAATTACATCAAGTGCCAAGCTGGCCTAACTTCAGTCT
CCACCCACTCAGTGTGGGGAAACTCCATCGCATAAAACCCCTCCCCCCAACCTAAAGACGACGTACTCCAAAAGCTCGA
GAACTAATCGAGGTGCCTGGACGGCGCCCGGTACTCCGTGGAGTCACATGAAGCGACGGCTGAGGACGGAAAGGCCCTT
TTCCTTTGTGTGGGTGACTCACCCGCCCGCTCTCCCGAGCGCCGCGTCCTCCATTTTGAGCTCCCTGCAGCAGGGCCGG
GAAGCGGCCATCTTTCCGCTCACGCAACTGGTGCCGACCGGGCCAGCCTTGCCGCCCAGGGCGGGGCGATACACGGCGG
CGCGAGGCCAGGCACCAGAGCAGGCCGGCCAGCTTGAGACTACCCCCGTCCGATTCTCGGTGGCCGCGCTCGCAGGCCC
CGCCTCGCCGAACATGTGCGCTGGGACGCACGGGCCCCGTCGCCGCCCGCGGCCCCAAAAACCGAAATACCAGTGTGCA
GATCTTGGCCCGCATTTACAAGACTATCTTGCCAGAAAAAAAGCGTCGCAGCAGGTCATCAAAAATTTTAAATGGCTAG
AGACTTATCGAAAGCAGCGAGACAGGCGCGAAGGTGCCACCAGATTCGCACGCGGCGGCCCCAGCGCCCAAGCCAGGCC
TCAACTCAAGCACGAGGCGAAGGGGCTCCTTAAGCGCAAGGCCTCGAACTCTCCCACCCACTTCCAACCCGAAGCTCGG
GATCAAGAATCACGTACTGCAGCCAGGGGCGTGGAAGTAATTCAAGGCACGCAAGGGCCATAACCCGTAAAGAGGCCAG
GCCCGCGGGAACCACACACGGCACTTACCTGTGTTCTGGCGGCAAACCCGTTGCGAAAAAGAACGTTCACGGCGACTAC
TGCACTTATATACGGTTCTCCCCCACCCTCGGGAAAAAGGCGGAGCCAGTACACGACATCACTTTCCCAGTTTACCCCG
CGCCACCTTCTCTAGGCACCGGTTCAATTGCCGACCCCTCCCCCCAACTTCTCGGGGACTGTGGGCGATGTGCGCTCTG
CCCACTGACGGGCACCGGAGCCTCACGCATGCTCTTCTCCACCTCAGTGATGACGAGAGCGGGCGGGTGAGGGGGCGGG

AACGCAGCGATCTCTGGGTTCTACGTTAGTGGGAGTTTAACGACGGTCCCTGGGATTCCCCAAGGCAGGGGCGAGTCCT
TTTGTATGAATTACTCCATGGTAATAGCGATGACTAATACGTAGATGTACTGCCAAGTAGGAAAGTCCCATAAGGTCAT
GTACTGGGCATAATACTAGTATCGATAAAATTTTGAATTTTGTAATTTGTTTTTGTAATTCTTTAGTTTGTATGTCTGT
TGCTATTATGTCTACTATTCTTTCCCCTGCACTGTACCCCCCAATCCCCCCTTTTCTTTTAAAAGTTAACCGATACCGT
CGAGATCCGTTCACTAATCGAATGGATCTGTCTCTGTCTCTCTCTCCACCTTCTTCTTCTATTCCTTCGGGCCTGTCGG
GTCCCCTCGGGGTTGGGAGGTGGGTCTGAAACGATAATGGTGAATATCCCTGCCTAACTCTATTCACTATAGAAAGTAC
AGCATAAACTATTCTTAAACCTACCAAGCCTCCTACTATCATTATGAATAATTTTATATACCACAGCCAATTTGTTATG
TTAAACCAATTCCACAAACTTGCCCATTTATCTAATTCCAATAATTCTTGTTCATTCTTTTCTTGCTGGTTTTGCGATT
CTTCAATTAAGGAGTGTATTAAGCTTGTGTAATTGTTAATTTCTCTGTCCCACTCCATCCAGGTCGTGTGATTCCAAAT
CTGTTCCAGAGATTTATTACTCCAACTAGCATTCCAAGGCACAGCAGTGGTGCAAATGAGTTTTCCAGAGCAACCCCAA
ATCCCCAGGAGCTGTTGATCCTTTAGGTATCTTTCCACAGCCAGGATTCTTGCCTGGAGCTGCTTGATGCCCCAGACTG
TGAGTTGCAACAGATGCTGTTGCGCCTCAATAGCCCTCAGCAAATTGTTCTGCTGCTGCACTATACCAGACAATAATTG
TCTGGCCTGTACCGTCAGCGTCATTGACGCTGCGCCCATAGTGCTTCCTGCTGCTCCCAAGAACCCAAGGAACAAAGCT
CCTATTCCCACTGCTCTTTTTTCTCTCTGCACCACTCTTCTCTTTGCCTTGGTGGGTGCTACTCCTAATGGTTCAATTT
TTACTACTTTATATTTATATAATTCACTTCTCCAATTGTCCCTCATATCTCCTCCTCCAGGTCTGAAGATCAGTGGCCG
CTTGCTGTGCGGTGGTCTTACTTTTGTTTTGCTCTTCCTCTATCTTGTCTAAAGCTTCCTTGGTGTCTTTTATCTCTAT
CCTTTGATGCACACAATAGAGGGTTGCTACTGTATTATATAATGATCTAAGTTCTTCTGATCCTGTCTGAAGGGATGGT
TGTAGCTGTCCCAGTATTTGTCTACAGCCTTCTGATGTTTCTAACAGGCCAGGATTAACTGCGAATCGTTCTAGCTCCC
TGCTTGCCCATACTATATGTTTTAATTTATATTTTTTCTTTCCCCCTGGCCTTAACCGAATTTTTTCCCATCGCGATCT
AATTCTCCCCCGCTTAATACTGACGCTCTCGCACCCATCTCTCTCCTTCTAGCCTCCGCTAGTCAAAATTTTTGGCGTA
CTCACCAGTCGCCGCCCCTCGCCTCTTGCCGTGCGCGCTTCAGCAAGCCGAGTCCTGCGTCGAGAGAGCTCCTCTGGTT
TCCCTTTCGCTTTCAAGTCCCTGTTCGGGCGCCACTGCTAGAGATTTTCCACACTGACTAAAAGGGTCTGAGGGATCTC
TAGTTACCAGAGTCACACAACAGACGGGCACACACTACTTGAAGCACTCAAGGCAAGCTTTATTGAGGCTTAAGCAGTG
GGTTCCCTAGTTAGCCAGAGAGCTCCCAGGCTCAGATCTGGTCTAACCAGAGAGACCCAGTACAAGCAAAAAGCAGATC
TTATCTTCGTTGGGAGTGAATTAGCCCTTCCAGTAAAGGACGATGATTTCCCCGACAACACCACGGAATTGTCAGTGCC
CAACAGCCGAGCCCCTGTCCAGCAGCGGGCAAGGCAGGCGGCGATGAGTTCCGCCGTGGCAATAGGGAGGGGGAAAGCG
AAAGTCCCGGAAAGGAGCTGACAGGTGGTGGCAATGCCCCAACCAGTGGGGGTTGCGTCAGCAAACACAGTGCACACCA
CGCCACGTTGCCTGACAACGGGCCACAACTCCTCATAAAGAGACAGCAACCAGGATTTATACAAGGAGGAGAAAATGAA
AGCCATACGGGAAGCAATAGCATGATACAAAGGCATTAAAGCAGCGTATCCACATAGCGTAAAAGGAGCAACATAGTTA
AGAATACCAGTCAATCTTTCACAAATTTTGTAATCCAGAGGTTGATTTCTAGATTATTAGCGAGGGGGCAGGGCCTGCA
TGTGAAGGGCGTCGTAGGTGTCCTTGGTGGCTGTACTGAGACCCTGGTAAAGGCCATCGTGCCCCTTGCCCCTCCGGCG
CTCGCCTTTCATCCCAATCTCACTGTAGGCCTCCGCCATCTTATCTTTCTGCAGTTCATTGTACAGGCCTTCCTGAGGG
TTCTTCCTTCTCGGCTTTCCCCCCATCTCAGGGTCCCGGCCACGTCTCTTGTCCAAAACATCGTACTCCTCTCTTCGTC
CTAGATTGAGCTCGTTATAGAGCTGGTTCTGGCCCTGCTTGTACGCGGGGGCGTCTGCGCTCCTGCTGAACTTCACTCT
CAGTTCACATCCTCCTTCTTCTAGTCAGTGTGGAAAATCTCTAGCA
>IS4_VCN3_chr8:99830407
AAACTGCTAGAGATTTTCCACACTGACTAAAAGGGTCTGAGGGATCTCTAGTTACCAGAGTCACACAACAGACGGGCAC
ACACTACTTGAAGCACTCAAGGCAAGCTTTATTGAGGCTTAAGCAGTGGGTTCCCTAGTTAGCCAGAGAGCTCCCAGGC
TCAGAGAGCTCCAGAGAGAGCTCCCAGGCTCCCAGGCTCAGAGAGCTCCCAGGCTCAGAGAGCTCCCAGGCCCAGAGAG
CTCCCAGCCAGAGAGCTCCCAGGCTC
>IS5_VCN3_chrX:103930456
AATACTGCTAGAGATTTTCCACACTGACTAAAAGGGTCTGAGGGATCTCTAGTTACCAGAGTCACACAACAGACGGGCA
CACACTACTTGAAGCACTCAAGGCAAGCTTTATTGAGGCTTAAGCAGTGGGTTCCCTAGTTAGCCAGAGAGCTCCCAGG
CTCAGATCTGGTCTAACCAGAGAGACCCAGTACAAGCAAAAAGCAGATCTTATCTTCGTTGGGAGTGAATTAGCCCTTC
CAGTCCCCCCTTTTCTTTTAAAAAGTGGCTAAGATCTACAGCTGCCTTGTAAGTCATTGGTCTTAAATTATCAGTCCTG
CTCCTCTGCCACAAAGTGCACGCAGTTGCCGGCCGGGTCGCGCAGGGCGAACTCCCGCCCCCACGGCTGCTCGCCGATC
TCGGTCATGGCCGGCCCGGAGGCGTCCCGGAAGTTCGTGGACACGACCTCCGACCACTCGGCGTACAGCTCGTCCAGGC
CGCGCACCCACACCCAGGCCAGGGTGTTGTCCGGCACCACCTGGTCCTGGACCGCGCTGATGAACAGGGTCACGTCGTC
CCGGACCACACCGGCGAAGTCGTCCTCCACGAAGTCCCGGGAGAACCCGAGCCGGTCGGTCCAGAACTCGACCGCTCCG
GCGACGTCGCGCGCGGTGAGCACCGGAACGGCACTGGTCAACTTGGCCATAGGACCGGGGTTTTCTTCCACGTCTCCTG
CTTGCTTTAACAGAGAGAAGTTCGTGGCTCCAGAACCCTTGTACAGCTCGTCCATGCCGAGAGTGATCCCGGCGGCGGT

CACGAACTCCAGCAGGACCATGTGATCGCGCTTCTCGTTGGGGTCTTTGCTCAGGGCGGACTGGGTGCTCAGGTAGTGG
TTGTCGGGCAGCAGCACGGGGCCGTCGCCGATGGGGGTGTTCTGCTGGTAGTGGTCGGCGAGCTGCACGCTGCCGTCCT
CGATGTTGTGGCGGATCTTGAAGTTCACCTTGATGCCGTTCTTCTGCTTGTCGGCCATGATATAGACGTTGTGGCTGTT
GTAGTTGTACTCCAGCTTGTGCCCCAGGATGTTGCCGTCCTCCTTGAAGTCGATGCCCTTCAGCTCGATGCGGTTCACC
AGGGTGTCGCCCTCGAACTTCACCTCGGCGCGGGTCTTGTAGTTGCCGTCGTCCTTGAAGAAGATGGTGCGCTCCTGGA
CGTAGCCTTCGGGCATGGCGGACTTGAAGAAGTCGTGCTGCTTCATGTGGTCGGGGTAGCGGCTGAAGCACTGCACGCC
GTAGGTCAGGGTGGTCACGAGGGTGGGCCAGGGCACGGGCAGCTTGCCGGTGGTGCAGATGAACTTCAGGGTCAGCTTG
CCGTAGGTGGCATCGCCCTCGCCCTCGCCGGACACGCTGAACTTGTGGCCGTTTACGTCGCCGTCCAGCTCGACCAGGA
TGGGCACCACCCCGGTGAACAGCTCCTCGCCCTTGCTCACCATGGTGGCAAGCTTGGGCTGCAGGTCGAAAGGCCCGGA
GATGAGGAAGAGGAGAACAGCGCGGCAGACGTGCGCTTTTGAAGCGTGCAGAATGCCGGGCCTCCGGAGGACCTTCGGG
CGCCCGCCCCGCCCCTGAGCCCGCCCCTGAGCCCGCCCCCGGACCCACCCCTTCCCAGCCTCTGAGCCCAGAAAGCGAA
GGAGCAAAGCTGCTATTGGCCGCTGCCCCAAAGGCCTACCCGCTTCCATTGCTCAGCGGTGCTGTCCATCTGCACGAGA
CTAGTGAGACGTGCTACTTCCATTTGTCACGTCCTGCACGACGCGAGCTGCGGGGCGGGGGGAACTTCCTGACTAGGG
GAGGAGTAGAAGGTGGCGCGAAGGGGCCACCAAAGAACGGAGCCGGTTGGCGCCTACCGGTGGATGTGGAATGTGTGCG
AGGCCAGAGGCCACTTGTGTAGCGCCAAGTGCCCAGCGGGGCTGCTAAAGCGCATGCTCCAGACTGCCTTGGGAAAAGC
GCCTCCCCTACCCGGTAGAATTGGTACCAGGCGGGGAGGCGGCCCAAAGGGAGATCCGACTCGTCTGAGGGCGAAGGCG
AAGACGCGGAAGAGGCCGCAGAGCCGGCAGCAGGCCGCGGGAAGGAAGGTCCGCTGGATTGAGGGCCGAAGGGACGTAG
CAGAAGGACGTCCCGCGCAGAATCCAGGTGGCAACACAGGCGAGCAGCCAAGGAAAGGACGATGATTTCCCCGACAACA
CCACGGAATTGTCAGTGCCCAACAGCCGAGCCCCTGTCCAGCAGCGGGCAAGGCAGGCGGCGATGAGTTCCGCCGTGGC
AATAGGGAGGGGGAAAGCGAAAGTCCCGGAAAGGAGCTGACAGGTGGTGGCAATGCCCCAACCAGTGGGGGTTGCGTCA
GCAAACACAGTGCACACCACGCCACGTTGCCTGACAACGGGCCACAACTCCTCATAAAGAGACAGCAACCAGGATTTAT
ACAAGGAGGAGAAAATGAAAGCCATACGGGAAGCAATAGCATGATACAAAGGCATTAAAGCAGCGTATCCACATAGCGT
AAAAGGAGCAACATAGTTAAGAATACCAGTCAATCTTTCACAAATTTTGTAATCCAGAGGTTGATTTCTAGATTATTAG
CGAGGGGGCAGGGCCTGCATGTGAAGGGCGTCGTAGGTGTCCTTGGTGGCTGTACTGAGACCCTGGTAAAGGCCATCGT
GCCCCTTGCCCCTCCGGCGCTCGCCTTTCATCCCAATCTCACTGTAGGCCTCCGCCATCTTATCTTTCTGCAGTTCATT
GTACAGGCCTTCCTGAGGGTTCTTCCTTCTCGGCTTTCCCCCCATCTCAGGGTCCCGGCCACGTCTCTTGTCCAAAACA
TCGTACTCCTCTCTTCGTCCTAGATTGAGCTCGTTATAGAGCTGGTTCTGGCCCTGCTTGTACGCGGGGGCGTCTGCGC
TCCTGCTGAACTTCACTCTCAGTTCACATCCTCCTTCTTCTTCTTCTGGAAATCGGCAGCTACAGCCATCTTCCTCTTG
AGTAGTTTGTACTGGTCTCATAAATGGTTGTTTGAATATATACAGGAGTTTCTTTCTGCCCCGTTTGCTCCGGTAGGCG
GCGAAATCCCTGGGAGGGGCGTAGGGCTGGTAGTGCTTCCGGGTGGGGCCAGGCCTCCGGGGGGTCATGTTCATGTAGT
CGCTGTGCAGCAGTCTGCTCCGCTTGCTCCTCACCCAAAAGATGATGAAGGCCACTGTCACCAGCAGGCTGTAGCAGGC
CAGCACGCCGCCCACAACGACCAGCACCCAGAAATCACAGGCGAAGTCCAGCCCCCTCGTGTGCACTGCGCCCCCCGCC
GCTGGCCGGCACGCTTCTGGGCGCAGGGACAGGGGCTGCGACGCGATGGTGGGCGCCGGTGTTGGTGGTCGCGGCGCTG
GCGTCGTGGTAGAAGAAACGGTCACGCTGGTGCCCTGGCCCCAATAATCCATGGCGTAGCTGCCGCCGTAGTAGTAGTG
CTTGGCGCAGTAGTAGATGGCGGTGTCGTCGGTCTGCAGGCTGTTCATCTTCAGGAACACCTGGCTCTTGCTGTTGTCC
TTGATGATGGTCAGCCGGGACTTCAGGGCGCTGTTGTAGTAGGTTGTCTCGCTGCCCCAGATCACTCCCAGCCATTCCA
GGCCTTTCCGAGGAGGCTGTCTGATCCAGGACACGCCGTAATCAGGCAGGGACACTCCGCTGACTGTACAGGTCACGCT
CAGAGACTGAGATGGGGCCACCAGTCCAGGGCCAGACTCTTGCAGCTTCACTTCAGAACCGCCTCCGCCACTTCCTCCG
CCACCAGATCCTCCGCCTCCGGTGATTTCCAGCTTGGTGCCTCCGCCAAAGGTGTAAGGCAGGGTGTTGCCTTGCTGGC
AGAAGTAGGTAGCGATATCCTCTTGTTCCAGGTTGCTGATTGTCAGGCTGTAGTCGGTGCCAGAGCCGCTGCCAGAAAA
TCTGCTTGGCACGCCGCTGTGCAGTCTGCTGGTGTGGTAGATCAGCAGCTTCACGGTGCCGTCGGGTTTCTGCTGATAC
CAGTTCAGGTACTTGCTGATGTCCTGGCTGGCTCTACAGCTGATGGTCACTCTATCGCCCAGGCTGGCAGACAGGCTGC
TTGTGGTCTGGGTCATCTGGATGTCGGGCCGAGCGGCGTGGAGCAGAAGAGCCAGCGGAAGCAGCAGGGCGGTGACAGG
GAGGGCCATGGTGGCGGCTTCGAATCACGACACCTGAAATGGAAGAAAAAAACTTTGAACCACTGTCTGAGGCTTGAGA
ATGAACCAAGATCCAAACTCAAAAAGGGCAAATTCCAAGGAGAATTACATCAAGTGCCAAGCTGGCCTAACTTCAGTCT
CCACCCACTCAGTGTGGGGAAACTCCATCGCATAAAACCCCTCCCCCCAACCTAAAGACGACGTACTCCAAAAGCTCGA
GAACTAATCGAGGTGCCTGGACGGCGCCCGGTACTCCGTGGAGTCACATGAAGCGACGGCTGAGGACGGAAAGGCCCTT
TTCCTTTGTGTGGGTGACTCACCCGCCCGCTCTCCCGAGCGCCGCGTCCTCCATTTTGAGCTCCCTGCAGCAGGGCCGG
GAAGCGGCCATCTTTCCGCTCACGCAACTGGTGCCGACCGGGCCAGCCTTGCCGCCCAGGGCGGGGCGATACACGGCGG
CGCGAGGCCAGGCACCAGAGCAGGCCGGCCAGCTTGAGACTACCCCCGTCCGATTCTCGGTGGCCGCGCTCGCAGGCCC
CGCCTCGCCGAACATGTGCGCTGGGACGCACGGGCCCCGTCGCCGCCCGCGGCCCCAAAAACCGAAATACCAGTGTGCA

GATCTTGGCCCGCATTTACAAGACTATCTTGCCAGAAAAAAAGCGTCGCAGCAGGTCATCAAAAATTTTAAATGGCTAG
AGACTTATCGAAAGCAGCGAGACAGGCGCGAAGGTGCCACCAGATTCGCACGCGGCGGCCCCAGCGCCCAAGCCAGGCC
TCAACTCAAGCACGAGGCGAAGGGGCTCCTTAAGCGCAAGGCCTCGAACTCTCCCACCCACTTCCAACCCGAAGCTCGG
GATCAAGAATCACGTACTGCAGCCAGGGGCGTGGAAGTAATTCAAGGCACGCAAGGGCCATAACCCGTAAAGAGGCCAG
GCCCGCGGGAACCACACACGGCACTTACCTGTGTTCTGGCGGCAAACCCGTTGCGAAAAAGAACGTTCACGGCGACTAC
TGCACTTATATACGGTTCTCCCCCACCCTCGGGAAAAAGGCGGAGCCAGTACACGACATCACTTTCCCAGTTTACCCCG
CGCCACCTTCTCTAGGCACCGGTTCAATTGCCGACCCCTCCCCCCAACTTCTCGGGGACTGTGGGCGATGTGCGCTCTG
CCCACTGACGGGCACCGGAGCCTCACGCATGCTCTTCTCCACCTCAGTGATGACGAGAGCGGGCGGGTGAGGGGGCGGG
AACGCAGCGATCTCTGGGTTCTACGTTAGTGGGAGTTTAACGACGGTCCCTGGGATTCCCCAAGGCAGGGGCGAGTCCT
TTTGTATGAATTACTCCATGGTAATAGCGATGACTAATACGTAGATGTACTGCCAAGTAGGAAAGTCCCATAAGGTCAT
GTACTGGGCATAATACTAGTATCGATAAAATTTTGAATTTTGTAATTTGTTTTTGTAATTCTTTAGTTTGTATGTCTGT
TGCTATTATGTCTACTATTCTTTCCCCTGCACTGTACCCCCCAATCCCCCCTTTTCTTTTAAAAGTTAACCGATACCGT
CGAGATCCGTTCACTAATCGAATGGATCTGTCTCTGTCTCTCTCTCCACCTTCTTCTTCTATTCCTTCGGGCCTGTCGG
GTCCCCTCGGGGTTGGGAGGTGGGTCTGAAACGATAATGGTGAATATCCCTGCCTAACTCTATTCACTATAGAAAGTAC
AGCAAAAACTATTCTTAAACCTACCAAGCCTCCTACTATCATTATGAATAATTTTATATACCACAGCCAATTTGTTATG
TTAAACCAATTCCACAAACTTGCCCATTTATCTAATTCCAATAATTCTTGTTCATTCTTTTCTTGCTGGTTTTGCGATT
CTTCAATTAAGGAGTGTATTAAGCTTGTGTAATTGTTAATTTCTCTGTCCCACTCCATCCAGGTCGTGTGATTCCAAAT
CTGTTCCAGAGATTTATTACTCCAACTAGCATTCCAAGGCACAGCAGTGGTGCAAATGAGTTTTCCAGAGCAACCCCAA
ATCCCCAGGAGCTGTTGATCCTTTAGGTATCTTTCCACAGCCAGGATTCTTGCCTGGAGCTGCTTGATGCCCCAGACTG
TGAGTTGCAACAGATGCTGTTGCGCCTCAATAGCCCTCAGCAAATTGTTCTGCTGCTGCACTATACCAGACAATAATTG
TCTGGCCTGTACCGTCAGCGTCATTGACGCTGCGCCCATAGTGCTTCCTGCTGCTCCCAAGAACCCAAGGAACAAAGCT
CCTATTCCCACTGCTCTTTTTTCTCTCTGCACCACTCTTCTCTTTGCCTTGGTGGGTGCTACTCCTAATGGTTCAATTT
TTACTACTTTATATTTATATAATTCACTTCTCCAATTGTCCCTCATATCTCCTCCTCCAGGTCTGAAGATCAGTGGCCG
CTTGCTGTGCGGTGGTCTTACTTTTGTTTTGCTCTTCCTCTATCTTGTCTAAAGCTTCCTTGGTGTCTTTTATCTCTAT
CCTTTGATGCACACAATAGAGGGTTGCTACTGTATTATATAATGATCTAAGTTCTTCTGATCCTGTCTGAAGGGATGGT
TGTAGCTGTCCCAGTATTTGTCTACAGCCTTCTGATGTTTCTAACAGGCCAGGATTAACTGCGAATCGTTCTAGCTCCC
TGCTTGCCCATACTATATGTTTTAATTTATATTTTTTCTTTCCCCCTGGCCTTAACCGAATTTTTTCCCATCGCGATCT
AATTCTCCCCCGCTTAATACTGACGCTCTCGCACCCATCTCTCTCCTTCTAGCCTCCGCTAGTCAAAATTTTTGGCGTA
CTCACCAGTCGCCGCCCCTCGCCTCTTGCCGTGCGCGCTTCAGCAAGCCGAGTCCTGCGTCGAGAGAGCTCCTCTGGTT
TCCCTTTCGCTTTCAAGTCCCTGTTCGGGCGCCACTGCTAGAGATTTTCCACACTGACTAAAAGGGTCTGAGGGATCTC
TAGTTACCAGAGTCACACAACAGACGGGCACACACTACTTGAAGCACTCAAGGCAAGCTTTATTGAGGCTTAAGCAGTG
GGTTCCCTAGTTAGCCAGAGAGCTCCCAGGCTCAGATCTGGTCTAACCAGAGAGACCCAGTACAAGCAAAAAGCAGATC
TTATCTTCGTTGGGAGTGAATTAGCCCTTCCA
>IS6_VCN4-VCN5_chr1:243273502
ACCCCTGGAAGGGCTAATTCACTCCCAACGAAGATAAGATCTGCTTTTTGCTTGTACTGGGTCTCTCTGGTTAGACCAG
ATCTGAGCCTGGGAGCTCTCTGGCTAACTAGGGAACCCACTGCTTAAGCCTCAATAAAGCTTGCCTTGAGTGCTTCAAG
TAGTGTGTGCCCGTCTGTTGTGTGACTCTGGTAACTAGAGATCCCTCAGACCCTTTTAGTCAGTGTGGAAAATCTCTAG
CAGTGGCGCCCGAACAGGGACTTGAAAGCGAAAGGGAAACCAGAGGAGCTCTCTCGACGCAGGACTCGGCTTGCTGAAG
CGCGCACGGCAAGAGGCGAGGGGCGGCGACTGGTGAGTACGCCAAAAATTTTGACTAGCGGAGGCTAGAAGGAGAGAGA
TGGGTGCGAGAGCGTCAGTATTAAGCGGGGGAGAATTAGATCGCGATGGGAAAAAATTCGGTTAAGGCCAGGGGGAAAG
AAAAAATATAAATTAAAACATATAGTATGGGCAAGCAGGGAGCTAGAACGATTCGCAGTTAATCCTGGCCTGTTAGAAA
CATCAGAAGGCTGTAGACAAATACTGGGACAGCTACAACCATCCCTTCAGACAGGATCAGAAGAACTTAGATCATTATA
TAATACAGTAGCAACCCTCTATTGTGTGCATCAAAGGATAGAGATAAAAGACACCAAGGAAGCTTTAGACAAGATAGAG
GAAGAGCAAAACAAAAGTAAGACCACCGCACAGCAAGCGGCCACTGATCTTCAGACCTGGAGGAGGAGATATGAGGGAC
AATTGGAGAAGTGAATTATATAAATATAAAGTAGTAAAAATTGAACCATTAGGAGTAGCACCCACCAAGGCAAAGAGAA
GAGTGGTGCAGAGAGAAAAAAGAGCAGTGGGAATAGGAGCTTTGTTCCTTGGGTTCTTGGGAGCAGCAGGAAGCACTAT
GGGCGCAGCGTCAATGACGCTGACGGTACAGGCCAGACAATTATTGTCTGGTATAGTGCAGCAGCAGAACAATTTGCTG
AGGGCTATTGAGGCGCAACAGCATCTGTTGCAACTCACAGTCTAGGGCATCAAGCAGCTCCAGGCAAGAATCCTGGCTG
TGGAAAGATACCTAAAGGATCAACAGCTCCTGGGGATTTGGGGTTGCTCTGGAAAACTCATTTGCACCACTGCTGTGCC
TTGGAATGCTAGTTGGAGTAATAAATCTCTGGAACAGATTTGGAATCACACGACCTGGATGGAGTGGGACAGAGAAATT
AACAATTACACAAGCTTAATACACTCCTTAATTGAAGAATCGCAAAACCAGCAAGAAAAGAATGAACAAGAATTATTGG

AATTAGATAAATGGGCAAGTTTGTGGAATTGGTTTAACATAACAAATTGGCTGTGGTATATAAAATTATTCATAATGAT
AGTAGGAGGCTTGGTAGGTTTAAGAATAGTTTTTGCTGTACTTTCTATAGTGAATAGAGTTAGGCAGGGATATTCACCA
TTATCGTTTCAGACCCACCTCCCAACCCCGAGGGGACCCGACAGGCCCGAAGGAATAGAAGAAGAAGGTGGAGAGAGAG
ACAGAGACAGATCCATTCGATTAGTGAACGGATCTCGACGGTATCGGTTAACTTTTAAAAGAAAAGGGGGGATTGGGGG
GTACAGTGCAGGGGAAAGAATAGTAGACATAATAGCAACAGACATACAAACTAAAGAATTACAAAAACAAATTACAAAA
TTCAAAATTTTATCGATACTAGTATTATGCCCAGTACATGACCTTATGGGACTTTCCTACTTGGCAGTACATCTACGTA
TTAGTCATCGCTATTACCATGGAGTAATTCATACAAAAGGACTCGCCCCTGCCTTGGGGAATCCCAGGGACCGTCGTTA
AACTCCCACTAACGTAGAACCCAGAGATCGCTGCGTTCCCGCCCCCTCACCCGCCCGCTCTCGTCATCACTGAGGTGGA
GAAGAGCATGCGTGAGGCTCCGGTGCCCGTCAGTGGGCAGAGCGCACATCGCCCACAGTCCCCGAGAAGTTGGGGGGAG
GGGTCGGCAATTGAACCGGTGCCTAGAGAAGGTGGCGCGGGGTAAACTGGGAAAGTGATGTCGTGTACTGGCTCCGCCT
TTTTCCCGAGGGTGGGGGAGAACCGTATATAAGTGCAGTAGTCGCCGTGAACGTTCTTTTTCGCAACGGGTTTGCCGCC
AGAACACAGGTAAGTGCCGTGTGTGGTTCCCGCGGGCCTGGCCTCTTTACGGGTTATGGCCCTTGCGTGCCTTGAATTA
CTTCCACGCCCCTGGCTGCAGTACGTGATTCTTGATCCCGAGCTTCGGGTTGGAAGTGGGTGGGAGAGTTCGAGGCCTT
GCGCTTAAGGAGCCCCTTCGCCTCGTGCTTGAGTTGAGGCCTGGCTTGGGCGCTGGGGCCGCCGCGTGCGAATCTGGTG
GCACCTTCGCGCCTGTCTCGCTGCTTTCGATAAGTCTCTAGCCATTTAAAATTTTTGATGACCTGCTGCGACGCTTTTT
TTCTGGCAAGATAGTCTTGTAAATGCGGGCCAAGATCTGCACACTGGTATTTCGGTTTTTGGGGCCGCGGGCGGCGACG
GGGCCCGTGCGTCCCAGCGCACATGTTCGGCGAGGCGGGGCCTGCGAGCGCGGCCACCGAGAATCGGACGGGGGTAGTC
TCAAGCTGGCCGGCCTGCTCTGGTGCCTGGCCTCGCGCCGCCGTGTATCGCCCCGCCCTGGGCGGCAAGGCTGGCCCGG
TCGGCACCAGTTGCGTGAGCGGAAAGATGGCCGCTTCCCGGCCCTGCTGCAGGGAGCTCAAAATGGAGGACGCGGCGCT
CGGGAGAGCGGGCGGGTGAGTCACCCACACAAAGGAAAAGGGCCTTTCCGTCCTCAGCCGTCGCTTCATGTGACTCCAC
GGAGTACCGGGCGCCGTCCAGGCACCTCGATTAGTTCTCGAGCTTTTGGAGTACGTCGTCTTTAGGTTGGGGGGAGGGG
TTTTATGCGATGGAGTTTCCCCACACTGAGTGGGTGGAGACTGAAGTTAGGCCAGCTTGGCACTTGATGTAATTCTCCT
TGGAATTTGCCCTTTTTGAGTTTGGATCTTGGTTCATTCTCAAGCCTCAGACAGTGGTTCAAAGTTTTTTTCTTCCATT
TCAGGTGTCGTGATTCGAAGCCGCCACCATGGCCCTCCCTGTCACCGCCCTGCTGCTTCCGCTGGCTCTTCTGCTCCAC
GCCGCTCGGCCCGACATCCAGATGACCCAGACCACAAGCAGCCTGTCTGCCAGCCTGGGCGATAGAGTGACCATCAGCT
GTAGAGCCAGCCAGGACATCAGCAAGTACCTGAACTGGTATCAGCAGAAACCCGACGGCACCGTGAAGCTGCTGATCTA
CCACACCAGCAGACTGCACAGCGGCGTGCCAAGCAGATTTTCTGGCAGCGGCTCTGGCACCGACTACAGCCTGACAATC
AGCAACCTGGAACAAGAGGATATCGCTACCTACTTCTGCCAGCAAGGCAACACCCTGCCTTACACCTTTGGCGGAGGCA
CCAAGCTGGAAATCACCGGAGGCGGAGGATCTGGTGGCGGAGGAAGTGGCGGAGGCGGTTCTGAAGTGAAGCTGCAAGA
GTCTGGCCCTGGACTGGTGGCCCCATCTCAGTCTCTGAGCGTGACCTGTACAGTCAGCGGAGTGTCCCTGCCTGATTAC
GGCGTGTCCTGGATCAGACAGCCTCCTCGGAAAGGCCTGGAATGGCTGGGAGTGATCTGGGGCAGCGAGACAACCTACT
ACAACAGCGCCCTGAAGTCCCGGCTGACCATCATCAAGGACAACAGCAAGAGCCAGGTGTTCCTGAAGATGAACAGCCT
GCAGACCGACGACACCGCCATCTACTACTGCGCCAAGCACTACTACTACGGCGGCAGCTACGCCATGGATTATTGGGGC
CAGGGCACCAGCGTGACCGTTTCTTCTACCACGACGCCAGCGCCGCGACCACCAACACCGGCGCCCACCATCGCGTCGC
AGCCCCTGTCCCTGCGCCCAGAAGCGTGCCGGCCAGCGGCGGGGGGCGCAGTGCACACGAGGGGGCTGGACTTCGCCTG
TGATTTCTGGGTGCTGGTCGTTGTGGGCGGCGTGCTGGCCTGCTACAGCCTGCTGGTGACAGTGGCCTTCATCATCTTT
TGGGTGAGGAGCAAGCGGAGCAGACTGCTGCACAGCGACTACATGAACATGACCCCCCGGAGGCCTGGCCCCACCCGGA
AGCACTACCAGCCCTACGCCCCTCCCAGGGATTTCGCCGCCTACCGGAGCAAACGGGGCAGAAAGAAACTCCTGTATAT
ATTCAAACAACCATTTATGAGACCAGTACAAACTACTCAAGAGGAAGATGGCTGTAGCTGCCGATTTCCAGAAGAAGAA
GAAGGAGGATGTGAACTGAGAGTGAAGTTCAGCAGGAGCGCAGACGCCCCCGCGTACAAGCAGGGCCAGAACCAGCTCT
ATAACGAGCTCAATCTAGGACGAAGAGAGGAGTACGATGTTTTGGACAAGAGACGTGGCCGGGACCCTGAGATGGGGGG
AAAGCCGAGAAGGAAGAACCCTCAGGAAGGCCTGTACAATGAACTGCAGAAAGATAAGATGGCGGAGGCCTACAGTGAG
ATTGGGATGAAAGGCGAGCGCCGGAGGGGCAAGGGGCACGATGGCCTTTACCAGGGTCTCAGTACAGCCACCAAGGACA
CCTACGACGCCCTTCACATGCAGGCCCTGCCCCCTCGCTAATAATCTAGAAATCAACCTCTGGATTACAAAATTTGTGA
AAGATTGACTGGTATTCTTAACTATGTTGCTCCTTTTACGCTATGTGGATACGCTGCTTTAATGCCTTTGTATCATGCT
ATTGCTTCCCGTATGGCTTTCATTTTCTCCTCCTTGTATAAATCCTGGTTGCTGTCTCTTTATGAGGAGTTGTGGCCCG
TTGTCAGGCAACGTGGCGTGGTGTGCACTGTGTTTGCTGACGCAACCCCCACTGGTTGGGGCATTGCCACCACCTGTCA
GCTCCTTTCCGGGACTTTCGCTTTCCCCCTCCCTATTGCCACGGCGGAACTCATCGCCGCCTGCCTTGCCCGCTGCTGG
ACAGGGGCTCGGCTGTTGGGCACTGACAATTCCGTGGTGTTGTCGGGGAAATCATCGTCCTTTCCTTGGCTGCTCGCCT
GTGTTGCCACCTGGATTCTGCGCGGGACGTCCTTCTGCTACGTCCCTTCGGCCCTCAATCCAGCGGACCTTCCTTCCCG
CGGCCTGCTGCCGGCTCTGCGGCCTCTTCCGCGTCTTCGCCTTCGCCCTCAGACGAGTCGGATCTCCCTTTGGGCCGCC

TCCCCGCCTGGTACCAATTCTACCGGGTAGGGGAGGCGCTTTTCCCAAGGCAGTCTGGAGCATGCGCTTTAGCAGCCCC
GCTGGGCACTTGGCGCTACACAAGTGGCCTCTGGCCTCGCACACATTCCACATCCACCGGTAGGCGCCAACCGGCTCCG
TTCTTTGGTGGCCCCTTCGCGCCACCTTCTACTCCTCCCCTAGTCAGGAAGTTCCCCCCCGCCCCGCAGCTCGCGTCGT
GCAGGACGTGACAAATGGAAGTAGCACGTCTCACTAGTCTCGTGCAGATGGACAGCACCGCTGAGCAATGGAAGCGGGT
AGGCCTTTGGGGCAGCGGCCAATAGCAGCTTTGCTCCTTCGCTTTCTGGGCTCAGAGGCTGGGAAGGGGTGGGTCCGGG
GGCGGGCTCAGGGGCGGGCTCAGGGGCGGGGCGGGCGCCCGAAGGTCCTCCGGAGGCCCGGCATTCTGCACGCTTCAAA
AGCGCACGTCTGCCGCGCTGTTCTCCTCTTCCTCATCTCCGGGCCTTTCGACCTGCAGCCCAAGCTTGCCACCATGGTG
AGCAAGGGCGAGGAGCTGTTCACCGGGGTGGTGCCCATCCTGGTCGAGCTGGACGGCGACGTAAACGGCCACAAGTTCA
GCGTGTCCGGCGAGGGCGAGGGCGATGCCACCTACGGCAAGCTGACCCTGAAGTTCATCTGCACCACCGGCAAGCTGCC
CGTGCCCTGGCCCACCCTCGTGACCACCCTGACCTACGGCGTGCAGTGCTTCAGCCGCTACCCCGACCACATGAAGCAG
CACGACTTCTTCAAGTCCGCCATGCCCGAAGGCTACGTCCAGGAGCGCACCATCTTCTTCAAGGACGACGGCAACTACA
AGACCCGCGCCGAGGTGAAGTTCGAGGGCGACACCCTGGTGAACCGCATCGAGCTGAAGGGCATCGACTTCAAGGAGGA
CGGCAACATCCTGGGGCACAAGCTGGAGTACAACTACAACAGCCACAACGTCTATATCATGGCCGACAAGCAGAAGAAC
GGCATCAAGGTGAACTTCAAGATCCGCCACAACATCGAGGACGGCAGCGTGCAGCTCGCCGACCACTACCAGCAGAACA
CCCCCATCGGCGACGGCCCCGTGCTGCTGCCCGACAACCACTACCTGAGCACCCAGTCCGCCCTGAGCAAAGACCCCAA
CGAGAAGCGCGATCACATGGTCCTGCTGGAGTTCGTGACCGCCGCCGGGATCACTCTCGGCATGGACGAGCTGTACAAG
GGTTCTGGAGCCACGAACTTCTCTCTGTTAAAGCAAGCAGGAGACGTGGAAGAAAACCCCGGTCCTATGGCCAAGTTGA
CCAGTGCCGTTCCGGTGCTCACCGCGCGCGACGTCGCCGGAGCGGTCGAGTTCTGGACCGACCGGCTCGGGTTCTCCCG
GGACTTCGTGGAGGACGACTTCGCCGGTGTGGTCCGGGACGACGTGACCCTGTTCATCAGCGCGGTCCAGGACCAGGTG
GTGCCGGACAACACCCTGGCCTGGGTGTGGGTGCGCGGCCTGGACGAGCTGTACGCCGAGTGGTCGGAGGTCGTGTCCA
CGAACTTCCGGGACGCCTCCGGGCCGGCCATGACCGAGATCGGCGAGCAGCCGTGGGGGCGGGAGTTCGCCCTGCGCGA
CCCGGCCGGCAACTGCGTGCACTTTGTGGCAGAGGAGCAGGACTGATAATTTAAGACCAATGACTTACAAGGCAGCTGT
AGATCTTAGCCACTTTTTAAAAGAAAAGGGGGGACTGGAAGGGCTAATTCACTCCCAACGAAGATAAGATCTGCTTTTT
GCTTGTACTGGGTCTCTCTGGTTAGACCAGATCTGAGCCTGGGAGCTCTCTGGCTAACTAGGGAACCCACTGCTTAAGC
CTCAATAAAGCTTGCCTTGAGTGCTTCAAGTAGTGTGTGCCCGTCTGTTGTGTGACTCTGGTAACTAGAGATCCCTCAG
ACCCTTTTAGTCAGTGTGGAAAATCTCTAGCAGTAAATCCTGCTACCTCGTTGGGAGTGAATTAGCCCTTCCA
>IS7_VCN4-VCN5_chr3:18115277
TACTCTGCTAGAGATTTTCCACACTGACTAAAAGGGTCTGAGGGATCTCTAGTTACCAGAGTCACACAACAGACGGGCA
CACACTACTTGAAGCACTCAAGGCAAGCTTTATTGAGGCTTAAGCAGTGGGTTCCCTAGTTAGCCAGAGAGCTCCCAGG
CTCAGATCTGGTCTAACCAGAGAGACCCAGTACAAGCAAAAAGCAGATCTTATCTTCGTTGGGAGTGAATTAGCCCTTC
CAGTCCCCCCTTTTCTTTTAAAAAGTGGCTAAGATCTACAGCTGCCTTGTAAGTCATTGGTCTTAAATTATCAGTCCTG
CTCCTCTGCCACAAAGTGCACGCAGTTGCCGGCCGGGTCGCGCAGGGCGAACTCCCGCCCCCACGGCTGCTCGCCGATC
TCGGTCATGGCCGGCCCGGAGGCGTCCCGGAAGTTCGTGGACACGACCTCCGACCACTCGGCGTACAGCTCGTCCAGGC
CGCGCACCCACACCCAGGCCAGGGTGTTGTCCGGCACCACCTGGTCCTGGACCGCGCTGATGAACAGGGTCACGTCGTC
CCGGACCACACCGGCGAAGTCGTCCTCCACGAAGTCCCGGGAGAACCCGAGCCGGTCGGTCCAGAACTCGACCGCTCCG
GCGACGTCGCGCGCGGTGAGCACCGGAACGGCACTGGTCAACTTGGCCATAGGACCGGGGTTTTCTTCCACGTCTCCTG
CTTGCTTTAACAGAGAGAAGTTCGTGGCTCCAGAACCCTTGTACAGCTCGTCCATGCCGAGAGTGATCCCGGCGGCGGT
CACGAACTCCAGCAGGACCATGTGATCGCGCTTCTCGTTGGGGTCTTTGCTCAGGGCGGACTGGGTGCTCAGGTAGTGG
TTGTCGGGCAGCAGCACGGGGCCGTCGCCGATGGGGGTGTTCTGCTGGTAGTGGTCGGCGAGCTGCACGCTGCCGTCCT
CGATGTTGTGGCGGATCTTGAAGTTCACCTTGATGCCGTTCTTCTGCTTGTCGGCCATGATATAGACGTTGTGGCTGTT
GTAGTTGTACTCCAGCTTGTGCCCCAGGATGTTGCCGTCCTCCTTGAAGTCGATGCCCTTCAGCTCGATGCGGTTCACC
AGGGTGTCGCCCTCGAACTTCACCTCGGCGCGGGTCTTGTAGTTGCCGTCGTCCTTGAAGAAGATGGTGCGCTCCTGGA
CGTAGCCTTCGGGCATGGCGGACTTGAAGAAGTCGTGCTGCTTCATGTGGTCGGGGTAGCGGCTGAAGCACTGCACGCC
GTAGGTCAGGGTGGTCACGAGGGTGGGCCAGGGCACGGGCAGCTTGCCGGTGGTGCAGATGAACTTCAGGGTCAGCTTG
CCGTAGGTGGCATCGCCCTCGCCCTCGCCGGACACGCTGAACTTGTGGCCGTTTACGTCGCCGTCCAGCTCGACCAGGA
TGGGCACCACCCCGGTGAACAGCTCCTCGCCCTTGCTCACCATGGTGGCAAGCTTGGGCTGCAGGTCGAAAGGCCCGGA
GATGAGGAAGAGGAGAACAGCGCGGCAGACGTGCGCTTTTGAAGCGTGCAGAATGCCGGGCCTCCGGAGGACCTTCGGG
CGCCCGCCCCGCCCCTGAGCCCGCCCCTGAGCCCGCCCCCGGACCCACCCCTTCCCAGCCTCTGAGCCCAGAAAGCGAA
GGAGCAAAGCTGCTATTGGCCGCTGCCCCAAAGGCCTACCCGCTTCCATTGCTCAGCGGTGCTGTCCATCTGCACGAGA
CTAGTGAGACGTGCTACTTCCATTTGTCACGTCCTGCACGACGCGAGCTGCGGGGCGGGGGGAACTTCCTGACTAGGG
GAGGAGTAGAAGGTGGCGCGAAGGGGCCACCAAAGAACGGAGCCGGTTGGCGCCTACCGGTGGATGTGGAATGTGTGCG

AGGCCAGAGGCCACTTGTGTAGCGCCAAGTGCCCAGCGGGGCTGCTAAAGCGCATGCTCCAGACTGCCTTGGGAAAAGC
GCCTCCCCTACCCGGTAGAATTGGTACCAGGCGGGGAGGCGGCCCAAAGGGAGATCCGACTCGTCTGAGGGCGAAGGCG
AAGACGCGGAAGAGGCCGCAGAGCCGGCAGCAGGCCGCGGGAAGGAAGGTCCGCTGGATTGAGGGCCGAAGGGACGTAG
CAGAAGGACGTCCCGCGCAGAATCCAGGTGGCAACACAGGCGAGCAGCCAAGGAAAGGACGATGATTTCCCCGACAACA
CCACGGAATTGTCAGTGCCCAACAGCCGAGCCCCTGTCCAGCAGCGGGCAAGGCAGGCGGCGATGAGTTCCGCCGTGGC
AATAGGGAGGGGGAAAGCGAAAGTCCCGGAAAGGAGCTGACAGGTGGTGGCAATGCCCCAACCAGTGGGGGTTGCGTCA
GCAAACACAGTGCACACCACGCCACGTTGCCTGACAACGGGCCACAACTCCTCATAAAGAGACAGCAACCAGGATTTAT
ACAAGGAGGAGAAAATGAAAGCCATACGGGAAGCAATAGCATGATACAAAGGCATTAAAGCAGCGTATCCACATAGCGT
AAAAGGAGCAACATAGTTAAGAATACCAGTCAATCTTTCACAAATTTTGTAATCCAGAGGTTGATTTCTAGATTATTAG
CGAGGGGGCAGGGCCTGCATGTGAAGGGCGTCGTAGGTGTCCTTGGTGGCTGTACTGAGACCCTGGTAAAGGCCATCGT
GCCCCTTGCCCCTCCGGCGCTCGCCTTTCATCCCAATCTCACTGTAGGCCTCCGCCATCTTATCTTTCTGCAGTTCATT
GTACAGGCCTTCCTGAGGGTTCTTCCTTCTCGGCTTTCCCCCCATCTCAGGGTCCCGGCCACGTCTCTTGTCCAAAACA
TCGTACTCCTCTCTTCGTCCTAGATTGAGCTCGTTATAGAGCTGGTTCTGGCCCTGCTTGTACGCGGGGGCGTCTGCGC
TCCTGCTGAACTTCACTCTCAGTTCACATCCTCCTTCTTCTTCTTCTGGAAATCGGCAGCTACAGCCATCTTCCTCTTG
AGTAGTTTGTACTGGTCTCATAAATGGTTGTTTGAATATATACAGGAGTTTCTTTCTGCCCCGTTTGCTCCGGTAGGCG
GCGAAATCCCTGGGAGGGGCGTAGGGCTGGTAGTGCTTCCGGGTGGGGCCAGGCCTCCGGGGGGTCATGTTCATGTAGT
CGCTGTGCAGCAGTCTGCTCCGCTTGCTCCTCACCCAAAAGATGATGAAGGCCACTGTCACCAGCAGGCTGTAGCAGGC
CAGCACGCCGCCCACAACGACCAGCACCCAGAAATCACAGGCGAAGTCCAGCCCCCTCGTGTGCACTGCGCCCCCCGCC
GCTGGCCGGCACGCTTCTGGGCGCAGGGACAGGGGCTGCGACGCGATGGTGGGCGCCGGTGTTGGTGGTCGCGGCGCTG
GCGTCGTGGTAGAAGAAACGGTCACGCTGGTGCCCTGGCCCCAATAATCCATGGCGTAGCTGCCGCCGTAGTAGTAGTG
CTTGGCGCAGTAGTAGATGGCGGTGTCGTCGGTCTGCAGGCTGTTCATCTTCAGGAACACCTGGCTCTTGCTGTTGTCC
TTGATGATGGTCAGCCGGGACTTCAGGGCGCTGTTGTAGTAGGTTGTCTCGCTGCCCCAGATCACTCCCAGCCATTCCA
GGCCTTTCCGAGGAGGCTGTCTGATCCAGGACACGCCGTAATCAGGCAGGGACACTCCGCTGACTGTACAGGTCACGCT
CAGAGACTGAGATGGGGTCACCAGTCCAGGGCCAGACTCTTGCAGCTTCACTTCAGAACCGCCTCCGCCACTTCCTCCG
CCACCAGATCCTCCGCCTCCGGTGATTTCCAGCTTGGTGCCTCCGCCAAAGGTGTAAGGCAGGGTGTTGCCTTGCTGGC
AGAAGTAGGTAGCGATATCCTCTTGTTCCAGGTTGCTGATTGTCAGGCTGTAGTCGGTGCCAGAGCCGCTGCCAGAAAA
TCTGCTTGGCACGCCGCTGTGCAGTCTGCTGGTGTGGTAGATCAGCAGCTTCACGGTGCCGTCGGGTTTCTGCTGATAC
CAGTTCAGGTACTTGCTGATGTCCTGGCTGGCTCTACAGCTGATGGTCACTCTATCGCCCAGGCTGGCAGACAGGCTGC
TTGTGGTCTGGGTCATCTGGATGTCGGGCCGAGCGGCGTGGAGCAGAAGAGCCAGCGGAAGCAGCAGGGCGGTGACAGG
GAGGGCCATGGTGGCGGCTTCGAATCACGACACCTGAAATGGAAGAAAAAAACTTTGAACCACTGTCTGAGGCTTGAGA
ATGAACCAAGATCCAAACTCAAAAAGGGCAAATTCCAAGGAGAATTACATCAAGTGCCAAGCTGGCCTAACTTCAGTCT
CCACCCACTCAGTGTGGGGAAACTCCATCGCATAAAACCCCTCCCCCCAACCTAAAGACGACGTACTCCAAAAGCTCGA
GAACTAATCGAGGTGCCTGGACGGCGCCCGGTACTCCGTGGAGTCACATGAAGCGACGGCTGAGGACGGAAAGGCCCTT
TTCCTTTGTGTGGGTGACTCACCCGCCCGCTCTCCCGAGCGCCGCGTCCTCCATTTTGAGCTCCCTGCAGCAGGGCCGG
GAAGCGGCCATCTTTCCGCTCACGCAACTGGTGCCGACCGGGCCAGCCTTGCCGCCCAGGGCGGGGCGATACACGGCGG
CGCGAGGCCAGGCACCAGAGCAGGCCGGCCAGCTTGAGACTACCCCCGTCCGATTCTCGGTGGCCGCGCTCGCAGGCCC
CGCCTCGCCGAACATGTGCGCTGGGACGCACGGGCCCCGTCGCCGCCCGCGGCCCCAAAAACCGAAATACCAGTGTGCA
GATCTTGGCCCGCATTTACAAGACTATCTTGCCAGAAAAAAAGCGTCGCAGCAGGTCATCAAAAATTTTAAATGGCTAG
AGACTTATCGAAAGCAGCGAGACAGGCGCGAAGGTGCCACCAGATTCGCACGCGGCGGCCCCAGCGCCCAAGCCAGGCC
TCAACTCAAGCACGAGGCGAAGGGGCTCCTTAAGCGCAAGGCCTCGAACTCTCCCACCCACTTCCAACCCGAAGCTCGG
GATCAAGAATCACGTACTGCAGCCAGGGGCGTGGAAGTAATTCAAGGCACGCAAGGGCCATAACCCGTAAAGAGGCCAG
GCCCGCGGGAACCACACACGGCACTTACCTGTGTTCTGGCGGCAAACCCGTTGCGAAAAAGAACGTTCACGGCGACTAC
TGCACTTATATACGGTTCTCCCCCACCCTCGGGAAAAAGGCGGAGCCAGTACACGACATCACTTTCCCAGTTTACCCCG
CGCCACCTTCTCTAGGCACCGGTTCAATTGCCGACCCCTCCCCCCAACTTCTCGGGGACTGTGGGCGATGTGCGCTCTG
CCCACTGACGGGCACCGGAGCCTCACGCATGCTCTTCTCCACCTCAGTGATGACGAGAGCGGGCGGGTGAGGGGGCGGG
AACGCAGCGATCTCTGGGTTCTACGTTAGTGGGAGTTTAACGACGGTCCCTGGGATTCCCCAAGGCAGGGGCGAGTCCT
TTTGTATGAATTACTCCATGGTAATAGCGATGACTAATACGTAGATGTACTGCCAAGTAGGAAAGTCCCATAAGGTCAT
GTACTGGGCATAATACTAGTATCGATAAAATTTTGAATTTTGTAATTTGTTTTTGTAATTCTTTAGTTTGTATGTCTGT
TGCTATTATGTCTACTATTCTTTCCCCTGCACTGTACCCCCCAATCCCCCCTTTTCTTTTAAAAGTTAACCGATACCGT
CGAGATCCGTTCACTAATCGAATGGATCTGTCTCTGTCTCTCTCTCCACCTTCTTCTTCTATTCCTTCGGGCCTGTCGG
GTCCCCTCGGGGTTGGGAGGTGGGTCTGAAACGATAATGGTGAATATCCCTGCCTAACTCTATTCACTATAGAAAGTAC

AGCAAAAACTATTCTTAAACCTACCAAGCCTCCTACTATCATTATGAATAATTTTATATACCACAGCCAATTTGTTATG
TTAAACCAATTCCACAAACTTGCCCATTTATCTAATTCCAATAATTCTTGTTCATTCTTTTCTTGCTGGTTTTGCGATT
CTTCAATTAAGGAGTGTATTAAGCTTGTGTAATTGTTAATTTCTCTGTCCCACTCCATCCAGGTCGTGTGATTCCAAAT
CTGTTCCAGAGATTTATTACTCCAACTAGCATTCCAAGGCACAGCAGTGGTGCAAATGAGTTTTCCAGAGCAACCCCAA
ATCCCCAGGAGCTGTTGATCCTTTAGGTATCTTTCCACAGCCAGGATTCTTGCCTGGAGCTGCTTGATGCCCCAGACTG
TGAGTTGCAACAGATGCTGTTGCGCCTCAATAGCCCTCAGCAAATTGTTCTGCTGCTGCACTATACCAGACAATAATTG
TCTGGCCTGTACCGTCAGCGTCATTGACGCTGCGCCCATAGTGCTTCCTGCTGCTCCCAAGAACCCAAGGAACAAAGCT
CCTATTCCCACTGCTCTTTTTTCTCTCTGCACCACTCTTCTCTTTGCCTTGGTGGGTGCTACTCCTAATGGTTCAATTT
TTACTACTTTATATTTATATAATTCACTTCTCCAATTGTCCCTCATATCTCCTCCTCCAGGTCTGAAGATCAGTGGCCG
CTTGCTGTGCGGTGGTCTTACTTTTGTTTTGCTCTTCCTCTATCTTGTCTAAAGCTTCCTTGGTGTCTTTTATCTCTAT
CCTTTGATGCACACAATAGAGGGTTGCTACTGTATTATATAATGATCTAAGTTCTTCTGATCCTGTCTGAAGGGATGGT
TGTAGCTGTCCCAGTATTTGTCTACAGCCTTCTGATGTTTCTAACAGGCCAGGATTAACTGCGAATCGTTCTAGCTCCC
TGCTTGCCCATACTATATGTTTTAATTTATATTTTTTCTTTCCCCCTGGCCTTAACCGAATTTTTTCCCATCGCGATCT
AATTCTCCCCCGCTTAATACTGACGCTCTCGCACCCATCTCTCTCCTTCTAGCCTCCGCTAGTCAAAATTTTTGGCGTA
CTCACCAGTCGCCGCCCCTCGCCTCTTGCCGTGCGCGCTTCAGCAAGCCGAGTCCTGCGTCGAGAGAGCTCCTCTGGTT
TCCCTTTCGCTTTCAAGTCCCTGTTCGGGCGCCACTGCTAGAGATTTTCCACACTGACTAAAAGGGTCTGAGGGATCTC
TAGTTACCAGAGTCACACAACAGACGGGCACACACTACTTGAAGCACTCAAGGCAAGCTTTATTGAGGCTTAAGCAGTG
GGTTCCCTAGTTAGCCAGAGAGCTCCCAGGCTCAGATCTGGTCTAACCAGAGAGACCCAGTACAAGCAAAAAGCAGATC
TTATCTTCGTTGGGAGTGAATTAGCCCTTCCA
>IS8_VCN4-VCN5_chr6:15601040
TGCTAGAGATTTTCCACACTGACTAAAAGGGTCTGAGGGATCTCTAGTTACCAGAGTCACACAACAGACGGGCACACAC
TACTTGAAGCACTCAAGGCAAGCTTTATTGAGGCTTAAGCAGTGGGTTCCCTAGTTAGCCAGAGAGCTCCCAGGCTCAG
ATCTGGTCTAACCAGAGAGACCCAGTACAAGCAAAAAGCAGATCTTATCTTCGTTGGGAGTGAATTAGCCCTTCCAGTC
CCCCCTTTTCTTTTAAAAAGTGGCTAAGATCTACAGCTGCCTTGTAAGTCATTGGTCTTAAATTATCAGTCCTGCTCCT
CTGCCACAAAGTGCACGCAGTTGCCGGCCGGGTCGCGCAGGGCGAACTCCCGCCCCCACGGCTGCTCGCCGATCTCGGT
CATGGCCGGCCCGGAGGCGTCCCGGAAGTTCGTGGACACGACCTCCGACCACTCGGCGTACAGCTCGTCCAGGCCGCGC
ACCCACACCCAGGCCAGGGTGTTGTCCGGCACCACCTGGTCCTGGACCGCGCTGATGAACAGGGTCACGTCGTCCCGGA
CCACACCGGCGAAGTCGTCCTCCACGAAGTCCCGGGAGAACCCGAGCCGGTCGGTCCAGAACTCGACCGCTCCGGCGAC
GTCGCGCGCGGTGAGCACCGGAACGGCACTGGTCAACTTGGCCATAGGACCGGGGTTTTCTTCCACGTCTCCTGCTTGC
TTTAACAGAGAGAAGTTCGTGGCTCCAGAACCCTTGTACAGCTCGTCCATGCCGAGAGTGATCCCGGCGGCGGTCACGA
ACTCCAGCAGGACCATGTGATCGCGCTTCTCGTTGGGGTCTTTGCTCAGGGCGGACTGGGTGCTCAGGTAGTGGTTGTC
GGGCAGCAGCACGGGGCCGTCGCCGATGGGGGTGTTCTGCTGGTAGTGGTCGGCGAGCTGCACGCTGCCGTCCTCGATG
TTGTGGCGGATCTTGAAGTTCACCTTGATGCCGTTCTTCTGCTTGTCGGCCATGATATAGACGTTGTGGCTGTTGTAGT
TGTACTCCAGCTTGTGCCCCAGGATGTTGCCGTCCTCCTTGAAGTCGATGCCCTTCAGCTCGATGCGGTTCACCAGGGT
GTCGCCCTCGAACTTCACCTCGGCGCGGGTCTTGTAGTTGCCGTCGTCCTTGAAGAAGATGGTGCGCTCCTGGACGTAG
CCTTCGGGCATGGCGGACTTGAAGAAGTCGTGCTGCTTCATGTGGTCGGGGTAGCGGCTGAAGCACTGCACGCCGTAGG
TCAGGGTGGTCACGAGGGTGGGCCAGGGCACGGGCAGCTTGCCGGTGGTGCAGATGAACTTCAGGGTCAGCTTGCCGTA
GGTGGCATCGCCCTCGCCCTCGCCGGACACGCTGAACTTGTGGCCGTTTACGTCGCCGTCCAGCTCGACCAGGATGGGC
ACCACCCCGGTGAACAGCTCCTCGCCCTTGCTCACCATGGTGGCAAGCTTGGGCTGCAGGTCGAAAGGCCCGGAGATGA
GGAAGAGGAGAACAGCGCGGCAGACGTGCGCTTTTGAAGCGTGCAGAATGCCGGGCCTCCGGAGGACCTTCGGGCGCCC
GCCCCGCCCCTGAGCCCGCCCCTGAGCCCGCCCCCGGACCCACCCCTTCCCAGCCTCTGAGCCCAGAAAGCGAAGGAGC
AAAGCTGCTATTGGCCGCTGCCCCAAAGGCCTACCCGCTTCCATTGCTCAGCGGTGCTGTCCATCTGCACGAGACTAGT
GAGACGTGCTACTTCCATTTGTCACGTCCTGCACGACGCGAGCTGCGGGGCGGGGGGAACTTCCTGACTAGGGGAGGAG
TAGAAGGTGGCGCGAAGGGGCCACCAAAGAACGGAGCCGGTTGGCGCCTACCGGTGGATGTGGAATGTGTGCGAGGCCA
GAGGCCACTTGTGTAGCGCCAAGTGCCCAGCGGGGCTGCTAAAGCGCATGCTCCAGACTGCCTTGGGAAAAGCGCCTCC
CCTACCCGGTAGAATTGGTACCAGGCGGGGAGGCGGCCCAAAGGGAGATCCGACTCGTCTGAGGGCGAAGGCGAAGACG
CGGAAGAGGCCGCAAG
>IS9_VCN4-VCN5_chr6:24825065
GTACTTGGAAGGGCTAATTCACTCCCAACGAAGATAAGATCTGCTTTTTGCTTGTACTGGGTCTCTCTGGTTAGACCAG
ATCTGAGCCTGGGAGCTCTCTGGCTAACTAGGGAACCCACTGCTTAAGCCTCAATAAAGCTTGCCTTGAGTGCTTCAAG
TAGTGTGTGCCCGTCTGTTGTGTGACTCTGGTAACTAGAGATCCCTCAGACCCTTTTAGTCAGTGTGGAAAATCTCTAG

CAGTGGCGCCCGAACAGGGACTTGAAAGCGAAAGGGAAACCAGAGGAGCTCTCTCGACGCAGGACTCGGCTTGCTGAAG
CGCGCACGGCAAGAGGCGAGGGGCGGCGACTGGTGAGTACGCCAAAAATTTTGACTAGCGGAGGCTAGAAGGAGAGAGA
TGGGTGCGAGAGCGTCAGTATTAAGCGGGGGAGAATTAGATCGCGATGGGAAAAAATTCGGTTAAGGCCAGGGGGAAAG
AAAAAATATAAATTAAAACATATAGTATGGGCAAGCAGGGAGCTAGAACGATTCGCAGTTAATCCTGGCCTGTTAGAAA
CATCAGAAGGCTGTAGACAAATACTGGGACAGCTACAACCATCCCTTCAGACAGGATCAGAAGAACTTAGATCATTATA
TAATACAGTAGCAACCCTCTATTGTGTGCATCAAAGGATAGAGATAAAAGACACCAAGGAAGCTTTAGACAAGATAGAG
GAAGAGCAAAACAAAAGTAAGACCACCGCACAGCAAGCGGCCACTGATCTTCAGACCTGGAGGAGGAGATATGAGGGAC
AATTGGAGAAGTGAATTATATAAATATAAAGTAGTAAAAATTGAACCATTAGGAGTAGCACCCACCAAGGCAAAGAGAA
GAGTGGTGCAGAGAGAAAAAAGAGCAGTGGGAATAGGAGCTTTGTTCCTTGGGTTCTTGGGAGCAGCAGGAAGCACTAT
GGGCGCAGCGTCAATGACGCTGACGGTACAGGCCAGACAATTATTGTCTGGTATAGTGCAGCAGCAGAACAATTTGCTG
AGGGCTATTGAGGCGCAACAGCATCTGTTGCAACTCACAGTCTGGGGCATCAAGCAGCTCCAGGCAAGAATCCTGGCTG
TGGAAAGATACCTAAAGGATCAACAGCTCCTGGGGATTTGGGGTTGCTCTGGAAAACTCATTTGCACCACTGCTGTGCC
TTGGAATGCTAGTTGGAGTAATAAATCTCTGGAACAGATTTGGAATCACACGACCTGGATGGAGTGGGACAGAGAAATT
AACAATTACACAAGCTTAATACACTCCTTAATTGAAGAATCGCAAAACCAGCAAGAAAAGAATGAACAAGAATTATTGG
AATTAGATAAATGGGCAAGTTTGTGGAATTGGTTTAACATAACAAATTGGCTGTGGTATATAAAATTATTCATAATGAT
AGTAGGAGGCTTGGTAGGTTTAAGAATAGTTTTTGCTGTACTTTCTATAGTGAATAGAGTTAGGCAGGGATATTCACCA
TTATCGTTTCAGACCCACCTCCCAACCCCGAGGGGACCCGACAGGCCCGAAGGAATAGAAGAAGAAGGTGGAGAGAGAG
ACAGAGACAGATCCATTCGATTAGTGAACGGATCTCGACGGTATCGGTTAACTTTTAAAAGAAAAGGGGGGATTGGGGG
GTACAGTGCAGGGGAAAGAATAGTAGACATAATAGCAACAGACATACAAACTAAAGAATTACAAAAACAAATTACAAAA
TTCAAAATTTTATCGATACTAGTATTATGCCCAGTACATGACCTTATGGGACTTTCCTACTTGGCAGTACATCTACGTA
TTAGTCATCGCTATTACCATGGAGTAATTCATACAAAAGGACTCGCCCCTGCCTTGGGGAATCCCAGGGACCGTCGTTA
AACTCCCACTAACGTAGAACCCAGAGATCGCTGCGTTCCCGCCCCCTCACCCGCCCGCTCTCGTCATCACTGAGGTGGA
GAAGAGCATGCGTGAGGCTCCGGTGCCCGTCAGTGGGCAGAGCGCACATCGCCCACAGTCCCCGAGAAGTTGGGGGGAG
GGGTCGGCAATTGAACCGGTGCCTAGAGAAGGTGGCGCGGGGTAAACTGGGAAAGTGATGTCGTGTACTGGCTCCGCCT
TTTTCCCGAGGGTGGGGGAGAACCGTATATAAGTGCAGTAGTCGCCGTGAACGTTCTTTTTCGCAACGGGTTTGCCGCC
AGAACACAGGTAAGTGCCGTGTGTGGTTCCCGCGGGCCTGGCCTCTTTACGGGTTATGGCCCTTGCGTGCCTTGAATTA
CTTCCACGCCCCTGGCTGCAGTACGTGATTCTTGATCCCGAGCTTCGGGTTGGAAGTGGGTGGGAGAGTTCGAGGCCTT
GCGCTTAAGGAGCCCCTTCGCCTCGTGCTTGAGTTGAGGCCTGGCTTGGGCGCTGGGGCCGCCGCGTGCGAATCTGGTG
GCACCTTCGCGCCTGTCTCGCTGCTTTCGATAAGTCTCTAGCCATTTAAAATTTTTGATGACCTGCTGCGACGCTTTTT
TTCTGGCAAGATAGTCTTGTAAATGCGGGCCAAGATCTGCACACTGGTATTTCGGTTTTTGGGGCCGCGGGCGGCGACG
GGGCCCGTGCGTCCCAGCGCACATGTTCGGCGAGGCGGGGCCTGCGAGCGCGGCCACCGAGAATCGGACGGGGGTAGTC
TCAAGCTGGCCGGCCTGCTCTGGTGCCTGGCCTCGCGCCGCCGTGTATCGCCCCGCCCTGGGCGGCAAGGCTGGCCCGG
TCGGCACCAGTTGCGTGAGCGGAAAGATGGCCGCTTCCCGGCCCTGCTGCAGGGAGCTCAAAATGGAGGACGCGGCGCT
CGGGAGAGCGGGCGGGTGAGTCACCCACACAAAGGAAAAGGGCCTTTCCGTCCTCAGCCGTCGCTTCATGTGACTCCAC
GGAGTACCGGGCGCCGTCCAGGCACCTCGATTAGTTCTCGAGCTTTTGGAGTACGTCGTCTTTAGGTTGGGGGGAGGGG
TTTTATGCGATGGAGTTTCCCCACACTGAGTGGGTGGAGACTGAAGTTAGGCCAGCTTGGCACTTGATGTAATTCTCCT
TGGAATTTGCCCTTTTTGAGTTTGGATCTTGGTTCATTCTCAAGCCTCAGACAGTGGTTCAAAGTTTTTTTCTTCCATT
TCAGGTGTCGTGATTCGAAGCCGCCACCATGGCCCTCCCTGTCACCGCCCTGCTGCTTCCGCTGGCTCTTCTGCTCCAC
GCCGCTCGGCCCGACATCCAGATGACCCAGACCACAAGCAGCCTGTCTGCCAGCCTGGGCGATAGAGTGACCATCAGCT
GTAGAGCCAGCCAGGACATCAGCAAGTACCTGAACTGGTATCAGCAGAAACCCGACGGCACCGTGAAGCTGCTGATCTA
CCACACCAGCAGACTGCACAGCGGCGTGCCAAGCAGATTTTCTGGCAGCGGCTCTGGCACCGACTACAGCCTGACAATC
AGCAACCTGGAACAAGAGGATATCGCTACCTACTTCTGCCAGCAAGGCAACACCCTGCCTTACACCTTTGGCGGAGGCA
CCAAGCTGGAAATCACCGGAGGCGGAGGATCTGGTGGCGGAGGAAGTGGCGGAGGCGGTTCTGAAGTGAAGCTGCAAGA
GTCTGGCCCTGGACTGGTGGCCCCATCTCAGTCTCTGAGCGTGACCTGTACAGTCAGCGGAGTGTCCCTGCCTGATTAC
GGCGTGTCCTGGATCAGACAGCCTCCTCGGAAAGGCCTGGAATGGCTGGGAGTGATCTGGGGCAGCGAGACAACCTACT
ACAACAGCGCCCTGAAGTCCCGGCTGACCATCATCAAGGACAACAGCAAGAGCCAGGTGTTCCTGAAGATGAACAGCCT
GCAGACCGACGACACCGCCATCTACTACTGCGCCAAGCACTACTACTACGGCGGCAGCTACGCCATGGATTATTGGGGC
CAGGGCACCAGCGTGACCGTTTCTTCTACCACGACGCCAGCGCCGCGACCACCAACACCGGCGCCCACCATCGCGTCGC
AGCCCCTGTCCCTGCGCCCAGAAGCGTGCCGGCCAGCGGCGGGGGGCGCAGTGCACACGAGGGGGCTGGACTTCGCCTG
TGATTTCTGGGTGCTGGTCGTTGTGGGCGGCGTGCTGGCCTGCTACAGCCTGCTGGTGACAGTGGCCTTCATCATCTTT
TGGGTGAGGAGCAAGCGGAGCAGACTGCTGCACAGCGACTACATGAACATGACCCCCCGGAGGCCTGGCCCCACCCGGA

AGCACTACCAGCCCTACGCCCCTCCCAGGGATTTCGCCGCCTACCGGAGCAAACGGGGCAGAAAGAAACTCCTGTATAT
ATTCAAACAACCATTTATGAGACCAGTACAAACTACTCAAGAGGAAGATGGCTGTAGCTGCCGATTTCCAGAAGAAGAA
GAAGGAGGATGTGAACTGAGAGTGAAGTTCAGCAGGAGCGCAGACGCCCCCGCGTACAAGCAGGGCCAGAACCAGCTCT
ATAACGAGCTCAATCTAGGACGAAGAGAGGAGTACGATGTTTTGGACAAGAGACGTGGCCGGGACCCTGAGATGGGGGG
AAAGCCGAGAAGGAAGAACCCTCAGGAAGGCCTGTACAATGAACTGCAGAAAGATAAGATGGCGGAGGCCTACAGTGAG
ATTGGGATGAAAGGCGAGCGCCGGAGGGGCAAGGGGCACGATGGCCTTTACCAGGGTCTCAGTACAGCCACCAAGGACA
CCTACGACGCCCTTCACATGCAGGCCCTGCCCCCTCGCTAATAATCTAGAAATCAACCTCTGGATTACAAAATTTGTGA
AAGATTGACTGGTATTCTTAACTATGTTGCTCCTTTTACGCTATGTGGATACGCTGCTTTAATGCCTTTGTATCATGCT
ATTGCTTCCCGTATGGCTTTCATTTTCTCCTCCTTGTATAAATCCTGGTTGCTGTCTCTTTATGAGGAGTTGTGGCCCG
TTGTCAGGCAACGTGGCGTGGTGTGCACTGTGTTTGCTGACGCAACCCCCACTGGTTGGGGCATTGCCACCACCTGTCA
GCTCCTTTCCGGGACTTTCGCTTTCCCCCTCCCTATTGCCACGGCGGAACTCATCGCCGCCTGCCTTGCCCGCTGCTGG
ACAGGGGCTCGGCTGTTGGGCACTGACAATTCCGTGGTGTTGTCGGGGAAATCATCGTCCTTTCCTTGGCTGCTCGCCT
GTGTTGCCACCTGGATTCTGCGCGGGACGTCCTTCTGCTACGTCCCTTCGGCCCTCAATCCAGCGGACCTTCCTTCCCG
CGGCCTGCTGCCGGCTCTGCGGCCTCTTCCGCGTCTTCGCCTTCGCCCTCAGACGAGTCGGATCTCCCTTTGGGCCGCC
TCCCCGCCTGGTACCAATTCTACCGGGTAGGGGAGGCGCTTTTCCCAAGGCAGTCTGGAGCATGCGCTTTAGCAGCCCC
GCTGGGCACTTGGCGCTACACAAGTGGCCTCTGGCCTCGCACACATTCCACATCCACCGGTAGGCGCCAACCGGCTCCG
TTCTTTGGTGGCCCCTTCGCGCCACCTTCTACTCCTCCCCTAGTCAGGAAGTTCCCCCCCGCCCCGCAGCTCGCGTCGT
GCAGGACGTGACAAATGGAAGTAGCACGTCTCACTAGTCTCGTGCAGATGGACAGCACCGCTGAGCAATGGAAGCGGGT
AGGCCTTTGGGGCAGCGGCCAATAGCAGCTTTGCTCCTTCGCTTTCTGGGCTCAGAGGCTGGGAAGGGGTGGGTCCGGG
GGCGGGCTCAGGGGCGGGCTCAGGGGCGGGGCGGGCGCCCGAAGGTCCTCCGGAGGCCCGGCATTCTGCACGCTTCAAA
AGCGCACGTCTGCCGCGCTGTTCTCCTCTTCCTCATCTCCGGGCCTTTCGACCTGCAGCCCAAGCTTGCCACCATGGTG
AGCAAGGGCGAGGAGCTGTTCACCGGGGTGGTGCCCATCCTGGTCGAGCTGGACGGCGACGTAAACGGCCACAAGTTCA
GCGTGTCCGGCGAGGGCGAGGGCGATGCCACCTACGGCAAGCTGACCCTGAAGTTCATCTGCACCACCGGCAAGCTGCC
CGTGCCCTGGCCCACCCTCGTGACCACCCTGACCTACGGCGTGCAGTGCTTCAGCCGCTACCCCGACCACATGAAGCAG
CACGACTTCTTCAAGTCCGCCATGCCCGAAGGCTACGTCCAGGAGCGCACCATCTTCTTCAAGGACGACGGCAACTACA
AGACCCGCGCCGAGGTGAAGTTCGAGGGCGACACCCTGGTGAACCGCATCGAGCTGAAGGGCATCGACTTCAAGGAGGA
CGGCAACATCCTGGGGCACAAGCTGGAGTACAACTACAACAGCCACAACGTCTATATCATGGCCGACAAGCAGAAGAAC
GGCATCAAGGTGAACTTCAAGATCCGCCACAACATCGAGGACGGCAGCGTGCAGCTCGCCGACCACTACCAGCAGAACA
CCCCCATCGGCGACGGCCCCGTGCTGCTGCCCGACAACCACTACCTGAGCACCCAGTCCGCCCTGAGCAAAGACCCCAA
CGAGAAGCGCGATCACATGGTCCTGCTGGAGTTCGTGACCGCCGCCGGGATCACTCTCGGCATGGACGAGCTGTACAAG
GGTTCTGGAGCCACGAACTTCTCTCTGTTAAAGCAAGCAGGAGACGTGGAAGAAAACCCCGGTCCTATGGCCAAGTTGA
CCAGTGCCGTTCCGGTGCTCACCGCGCGCGACGTCGCCGGAGCGGTCGAGTTCTGGACCGACCGGCTCGGGTTCTCCCG
GGACTTCGTGGAGGACGACTTCGCCGGTGTGGTCCGGGACGACGTGACCCTGTTCATCAGCGCGGTCCAGGACCAGGTG
GTGCCGGACAACACCCTGGCCTGGGTGTGGGTGCGCGGCCTGGACGAGCTGTACGCCGAGTGGTCGGAGGTCGTGTCCA
CGAACTTCCGGGACGCCTCCGGGCCGGCCATGACCGAGATCGGCGAGCAGCCGTGGGGGCGGGAGTTCGCCCTGCGCGA
CCCGGCCGGCAACTGCGTGCACTTTGTGGCAGAGGAGCAGGACTGATAATTTAAGACCAATGACTTACAAGGCAGCTGT
AGATCTTAGCCACTTTTTAAAAGAAAAGGGGGGACTGGAAGGGCTAATTCACTCCCAACGAAGATAAGATCTGCTTTTT
GCTTGTACTGGGTCTCTCTGGTTAGACCAGATCTGAGCCTGGGAGCTCTCTGGCTAACTAGGGAACCCACTGCTTAAGC
CTCAATAAAGCTTGCCTTGAGTGCTTCAAGTAGTGTGTGCCCGTCTGTTGTGTGACTCTGGTAACTAGAGATCCCTCAG
ACCCTTTTAGTCAGTGTGGAAAATCTCTAGCA
>IS10_VCN5_chr5:39044696
TTCACTGCTAGAGATTTTCCACACTGACTAAAAGGGTCTGAGGGATCTCTAGTTACCAGAGTCACACAACAGACGGGCA
CACACTACTTGAAGCACTCAAGGCAAGCTTTATTGAGGCTTAAGCAGTGGGTTCCCTAGTTAGCCAGAGAGCTCCCAGG
CTCAGATCTGGTCTAACCAGAGAGACCCAGTACAAGCAAAAAGCAGATCTTATCTTCGTTGGGAGTGAATTAGCCCTTC
CAGTCCCCCCTTTTCTTTTAAAAAGTGGCTAAGATCTACAGCTGCCTTGTAAGTCATTGGTCTTAAATTATCAGTCCTG
CTCCTCTGCCACAAAGTGCACGCAGTTGCCGGCCGGGTCGCGCAGGGCGAACTCCCGCCCCCACGGCTGCTCGCCGATC
TCGGTCATGGCCGGCCCGGAGGCGTCCCGGAAGTTCGTGGACACGACCTCCGACCACTCGGCGTACAGCTCGTCCAGGC
CGCGCACCCACACCCAGGCCAGGGTGTTGTCCGGCACCACCTGGTCCTGGACCGCGCTGATGAACAGGGTCACGTCGTC
CCGGACCACACCGGCGAAGTCGTCCTCCACGAAGTCCCGGGAGAACCCGAGCCGGTCGGTCCAGAACTCGACCGCTCCG
GCGACGTCGCGCGCGGTGAGCACCGGAACGGCACTGGTCAACTTGGCCATAGGACCGGGGTTTTCTTCCACGTCTCCTG
CTTGCTTTAACAGAGAGAAGTTCGTGGCTCCAGAACCCTTGTACAGCTCGTCCATGCCGAGAGTGATCCCGGCGGCGGT

CACGAACTCCAGCAGGACCATGTGATCGCGCTTCTCGTTGGGGTCTTTGCTCAGGGCGGACTGGGTGCTCAGGTAGTGG
TTGTCGGGCAGCAGCACGGGGCCGTCGCCGATGGGGGTGTTCTGCTGGTAGTGGTCGGCGAGCTGCACGCTGCCGTCCT
CGATGTTGTGGCGGATCTTGAAGTTCACCTTGATGCCGTTCTTCTGCTTGTCGGCCATGATATAGACGTTGTGGCTGTT
GTAGTTGTACTCCAGCTTGTGCCCCAGGATGTTGCCGTCCTCCTTGAAGTCGATGCCCTTCAGCTCGATGCGGTTCACC
AGGGTGTCGCCCTCGAACTTCACCTCGGCGCGGGTCTTGTAGTTGCCGTCGTCCTTGAAGAAGATGGTGCGCTCCTGGA
CGTAGCCTTCGGGCATGGCGGACTTGAAGAAGTCGTGCTGCTTCATGTGGTCGGGGTAGCGGCTGAAGCACTGCACGCC
GTAGGTCAGGGTGGTCACGAGGGTGGGCCAGGGCACGGGCAGCTTGCCGGTGGTGCAGATGAACTTCAGGGTCAGCTTG
CCGTAGGTGGCATCGCCCTCGCCCTCGCCGGACACGCTGAACTTGTGGCCGTTTACGTCGCCGTCCAGCTCGACCAGGA
TGGGCACCACCCCGGTGAACAGCTCCTCGCCCTTGCTCACCATGGTGGCAAGCTTGGGCTGCAGGTCGAAAGGCCCGGA
GATGAGGAAGAGGAGAACAGCGCGGCAGACGTGCGCTTTTGAAGCGTGCAGAATGCCGGGCCTCCGGAGGACCTTCGGG
CGCCCGCCCCGCCCCTGAGCCCGCCCCTGAGCCCGCCCCCGGACCCACCCCTTCCCAGCCTCTGAGCCCAGAAAGCGAA
GGAGCAAAGCTGCTATTGGCCGCTGCCCCAAAGGCCTACCCGCTTCCATTGCTCAGCGGTGCTGTCCATCTGCACGAGA
CTAGTGAGACGTGCTACTTCCATTTGTCACGTCCTGCACGACGCGAGCTGCGGGGCGGGGGGGAACTTCCTGACTAGGG
GAGGAGTAGAAGGTGGCGCGAAGGGGCCACCAAAGAACGGAGCCGGTTGGCGCCTACCGGTGGATGTGGAATGTGTGCG
AGGCCAGAGGCCACTTGTGTAGCGCCAAGTGCCCAGCGGGGCTGCTAAAGCGCATGCTCCAGACTGCCTTGGGAAAAGC
GCCTCCCCTACCCGGTAGAATTGGTACCAGGCGGGGAGGCGGCCCAAAGGGAGATCCGACTCGTCTGAGGGCGAAGGCG
AAGACGCGGAAGAGGCCGCAGAGCCGGCAGCAGGCCGCGGGAAGGAAGGTCCGCTGGATTGAGGGCCGAAGGGACGTAG
CAGAAGGACGTCCCGCGCAGAATCCAGGTGGCAACACAGGCGAGCAGCCAAGGAAAGGACGATGATTTCCCCGACAACA
CCACGGAATTGTCAGTGCCCAACAGCCGAGCCCCTGTCCAGCAGCGGGCAAGGCAGGCGGCGATGAGTTCCGCCGTGGC
AATAGGGAGGGGGAAAGCGAAAGTCCCGGAAAGGAGCTGACAGGTGGTGGCAATGCCCCAACCAGTGGGGGTTGCGTCA
GCAAACACAGTGCACACCACGCCACGTTGCCTGACAACGGGCCACAACTCCTCATAAAGAGACAGCAACCAGGATTTAT
ACAAGGAGGAGAAAATGAAAGCCATACGGGAAGCAATAGCATGATACAAAGGCATTAAAGCAGCGTATCCACATAGCGT
AAAAGGAGCAACATAGTTAAGAATACCAGTCAATCTTTCACAAATTTTGTAATCCAGAGGTTGATTTCTAGATTATTAG
CGAGGGGGCAGGGCCTGCATGTGAAGGGCGTCGTAGGTGTCCTTGGTGGCTGTACTGAGACCCTGGTAAAGGCCATCGT
GCCCCTTGCCCCTCCGGCGCTCGCCTTTCATCCCAATCTCACTGTAGGCCTCCGCCATCTTATCTTTCTGCAGTTCATT
GTACAGGCCTTCCTGAGGGTTCTTCCTTCTCGGCTTTCCCCCCATCTCAGGGTCCCGGCCACGTCTCTTGTCCAAAACA
TCGTACTCCTCTCTTCGTCCTAGATTGAGCTCGTTATAGAGCTGGTTCTGGCCCTGCTTGTACGCGGGGGCGTCTGCGC
TCCTGCTGAACTTCACTCTCAGTTCACATCCTCCTTCTTCTTCTTCTGGAAATCGGCAGCTACAGCCATCTTCCTCTTG
AGTAGTTTGTACTGGTCTCATAAATGGTTGTTTGAATATATACAGGAGTTTCTTTCTGCCCCGTTTGCTCCGGTAGGCG
GCGAAATCCCTGGGAGGGGCGTAGGGCTGGTAGTGCTTCCGGGTGGGGCCAGGCCTCCGGGGGGTCATGTTCATGTAGT
CGCTGTGCAGCAGTCTGCTCCGCTTGCTCCTCACCCAAAAGATGATGAAGGCCACTGTCACCAGCAGGCTGTAGCAGGC
CAGCACGCCGCCCACAACGACCAGCACCCAGAAATCACAGGCGAAGTCCAGCCCCCTCGTGTGCACTGCGCCCCCCGCC
GCTGGCCGGCACGCTTCTGGGCGCAGGGACAGGGGCTGCGACGCGATGGTGGGCGCCGGTGTTGGTGGTCGCGGCGCTG
GCGTCGTGGTAGAAGAAACGGTCACGCTGGTGCCCTGGCCCCAATAATCCATGGCGTAGCTGCCGCCGTAGTAGTAGTG
CTTGGCGCAGTAGTAGATGGCGGTGTCGTCGGTCTGCAGGCTGTTCATCTTCAGGAACACCTGGCTCTTGCTGTTGTCC
TTGATGATGGTCAGCCGGGACTTCAGGGCGCTGTTGTAGTAGGTTGTCTCGCTGCCCCAGATCACTCCCAGCCATTCCA
GGCCTTTCCGAGGAGGCTGTCTGATCCAGGACACGCCGTAATCAGGCAGGGACACTCCGCTGACTGTACAGGTCACGCT
CAGAGACTGAGATGGGGCCACCAGTCCAGGGCCAGACTCTTGCAGCTTCACTTCAGAACCGCCTCCGCCACTTCCTCCG
CCACCAGATCCTCCGCCTCCGGTGATTTCCAGCTTGGTGCCTCCGCCAAAGGTGTAAGGCAGGGTGTTGCCTTGCTGGC
AGAAGTAGGTAGCGATATCCTCTTGTTCCAGGTTGCTGATTGTCAGGCTGTAGTCGGTGCCAGAGCCGCTGCCAGAAAA
TCTGCTTGGCACGCCGCTGTGCAGTCTGCTGGTGTGGTAGATCAGCAGCTTCACGGTGCCGTCGGGTTTCTGCTGATAC
CAGTTCAGGTACTTGCTGATGTCCTGGCTGGCTCTACAGCTGATGGTCACTCTATCGCCCAGGCTGGCAGACAGGCTGC
TTGTGGTCTGGGTCATCTGGATGTCGGGCCGAGCGGCGTGGAGCAGAAGAGCCAGCGGAAGCAGCAGGGCGGTGACAGG
GAGGGCCATGGTGGCGGCTTCGAATCACGACACCTGAAATGGAAGAAAAAAACTTTGAACCACTGTCTGAGGCTTGAGA
ATGAACCAAGATCCAAACTCAAAAAGGGCAAATTCCAAGGAGAATTACATCAAGTGCCAAGCTGGCCTAACTTCAGTCT
CCACCCACTCAGTGTGGGGAAACTCCATCGCATAAAACCCCTCCCCCCAACCTAAAGACGACGTACTCCAAAAGCTCGA
GAACTAATCGAGGTGCCTGGACGGCGCCCGGTACTCCGTGGAGTCACATGAAGCGACGGCTGAGGACGGAAAGGCCCTT
TTCCTTTGTGTGGGTGACTCACCCGCCCGCTCTCCCGAGCGCCGCGTCCTCCATTTTGAGCTCCCTGCAGCAGGGCCGG
GAAGCGGCCATCTTTCCGCTCACGCAACTGGTGCCGACCGGGCCAGCCTTGCCGCCCAGGGCGGGGCGATACACGGCGG
CGCGAGGCCAGGCACCAGAGCAGGCCGGCCAGCTTGAGACTACCCCCGTCCGATTCTCGGTGGCCGCGCTCGCAGGCCC
CGCCTCGCCGAACATGTGCGCTGGGACGCACGGGCCCCGTCGCCGCCCGCGGCCCCAAAAACCGAAATACCAGTGTGCA

GATCTTGGCCCGCATTTACAAGACTATCTTGCCAGAAAAAAAGCGTCGCAGCAGGTCATCAAAAATTTTAAATGGCTAG
AGACTTATCGAAAGCAGCGAGACAGGCGCGAAGGTGCCACCAGATTCGCACGCGGCGGCCCCAGCGCCCAAGCCAGGCC
TCAACTCAAGCACGAGGCGAAGGGGCTCCTTAAGCGCAAGGCCTCGAACTCTCCCACCCACTTCCAACCCGAAGCTCGG
GATCAAGAATCACGTACTGCAGCCAGGGGCGTGGAAGTAATTCAAGGCACGCAAGGGCCATAACCCGTAAAGAGGCCAG
GCCCGCGGGAACCACACACGGCACTTACCTGTGTTCTGGCGGCAAACCCGTTGCGAAAAAGAACGTTCACGGCGACTAC
TGCACTTATATACGGTTCTCCCCCACCCTCGGGAAAAAGGCGGAGCCAGTACACGACATCACTTTCCCAGTTTACCCCG
CGCCACCTTCTCTAGGCACCGGTTCAATTGCCGACCCCTCCCCCCAACTTCTCGGGGACTGTGGGCGATGTGCGCTCTG
CCCACTGACGGGCACCGGAGCCTCACGCATGCTCTTCTCCACCTCAGTGATGACGAGAGCGGGCGGGTGAGGGGGCGGG
AACGCAGCGATCTCTGGGTTCTACGTTAGTGGGAGTTTAACGACGGTCCCTGGGATTCCCCAAGGCAGGGGCGAGTCCT
TTTGTATGAATTACTCCATGGTAATAGCGATGACTAATACGTAGATGTACTGCCAAGTAGGAAAGTCCCATAAGGTCAT
GTACTGGGCATAATACTAGTATCGATAAAATTTTGAATTTTGTAATTTGTTTTTGTAATTCTTTAGTTTGTATGTCTGT
TGCTATTATGTCTACTATTCTTTCCCCTGCACTGTACCCCCCAATCCCCCCTTTTCTTTTAAAAGTTAACCGATACCGT
CGAGATCCGTTCACTAATCGAATGGATCTGTCTCTGTCTCTCTCTCCACCTTCTTCTTCTATTCCTTCGGGCCTGTCGG
GTCCCCTCGGGGTTGGGAGGTGGGTCTGAAACGATAATGGTGAATATCCCTGCCTAACTCTATTCACTATAGAAAGTAC
AGCAAAAACTATTCTTAAACCTACCAAGCCTCCTACTATCATTATGAATAATTTTATATACCACAGCCAATTTGTTATG
TTAAACCAATTCCACAAACTTGCCCATTTATCTAATTCCAATAATTCTTGTTCATTCTTTTCTTGCTGGTTTTGCGATT
CTTCAATTAAGGAGTGTATTAAGCTTGTGTAATTGTTAATTTCTCTGTCCCACTCCATCCAGGTCGTGTGATTCCAAAT
CTGTTCCAGAGATTTATTACTCCAACTAGCATTCCAAGGCACAGCAGTGGTGCAAATGAGTTTTCCAGAGCAACCCCAA
ATCCCCAGGAGCTGTTGATCCTTTAGGTATCTTTCCACAGCCAGGATTCTTGCCTGGAGCTGCTTGATGCCCCAGACTG
TGAGTTGCAACAGATGCTGTTGCGCCTCAATAGCCCTCAGCAAATTGTTCTGCTGCTGCACTATACCAGACAATAATTG
TCTGGCCTGTACCGTCAGCGTCATTGACGCTGCGCCCATAGTGCTTCCTGCTGCTCCCAAGAACCCAAGGAACAAAGCT
CCTATTCCCACTGCTCTTTTTTCTCTCTGCACCACTCTTCTCTTTGCCTTGGTGGGTGCTACTCCTAATGGTTCAATTT
TTACTACTTTATATTTATATAATTCACTTCTCCAATTGTCCCTCATATCTCCTCCTCCAGGTCTGAAGATCAGTGGCCG
CTTGCTGTGCGGTGGTCTTACTTTTGTTTTGCTCTTCCTCTATCTTGTCTAAAGCTTCCTTGGTGTCTTTTATCTCTAT
CCTTTGATGCACACAATAGAGGGTTGCTACTGTATTATATAATGATCTAAGTTCTTCTGATCCTGTCTGAAGGGATGGT
TGTAGCTGTCCCAGTATTTGTCTACAGCCTTCTGATGTTTCTAACAGGCCAGGATTAACTGCGAATCGTTCTAGCTCCC
TGCTTGCCCATACTATATGTTTTAATTTATATTTTTTCTTTCCCCCTGGCCTTAACCGAATTTTTTCCCATCGCGATCT
AATTCTCCCCCGCTTAATACTGACGCTCTCGCACCCATCTCTCTCCTTCTAGCCTCCGCTAGTCAAAATTTTTGGCGTA
CTCACCAGTCGCCGCCCCTCGCCTCTTGCCGTGCGCGCTTCAGCAAGCCGAGTCCTGCGTCGAGAGAGCTCCTCTGGTT
TCCCTTTCGCTTTCAAGTCCCTGTTCGGGCGCCACTGCTAGAGATTTTCCACACTGACTAAAAGGGTCTGAGGGATCTC
TAGTTACCAGAGTCACACAACAGACGGGCACACACTACTTGAAGCACTCAAGGCAAGCTTTATTGAGGCTTAAGCAGTG
GGTTCCCTAGTTAGCCAGAGAGCTCCCAGGCTCAGATCTGGTCTAACCAGAGAGACCCAGTACAAGCAAAAAGCAGATC
TTATCTTCGTTGGGAGTGAATTAGCCCTTCCA
>expected_full_provirus_sequence_7374bp
TGGAAGGGCTAATTCACTCCCAACGAAGATAAGATCTGCTTTTTGCTTGTACTGGGTCTCTCTGGTTAGACCAGATCTG
AGCCTGGGAGCTCTCTGGCTAACTAGGGAACCCACTGCTTAAGCCTCAATAAAGCTTGCCTTGAGTGCTTCAAGTAGTG
TGTGCCCGTCTGTTGTGTGACTCTGGTAACTAGAGATCCCTCAGACCCTTTTAGTCAGTGTGGAAAATCTCTAGCAGTG
GCGCCCGAACAGGGACTTGAAAGCGAAAGGGAAACCAGAGGAGCTCTCTCGACGCAGGACTCGGCTTGCTGAAGCGCGC
ACGGCAAGAGGCGAGGGGCGGCGACTGGTGAGTACGCCAAAAATTTTGACTAGCGGAGGCTAGAAGGAGAGAGATGGGT
GCGAGAGCGTCAGTATTAAGCGGGGGAGAATTAGATCGCGATGGGAAAAAATTCGGTTAAGGCCAGGGGGAAAGAAAAA
ATATAAATTAAAACATATAGTATGGGCAAGCAGGGAGCTAGAACGATTCGCAGTTAATCCTGGCCTGTTAGAAACATCA
GAAGGCTGTAGACAAATACTGGGACAGCTACAACCATCCCTTCAGACAGGATCAGAAGAACTTAGATCATTATATAATA
CAGTAGCAACCCTCTATTGTGTGCATCAAAGGATAGAGATAAAAGACACCAAGGAAGCTTTAGACAAGATAGAGGAAGA
GCAAAACAAAAGTAAGACCACCGCACAGCAAGCGGCCACTGATCTTCAGACCTGGAGGAGGAGATATGAGGGACAATTG
GAGAAGTGAATTATATAAATATAAAGTAGTAAAAATTGAACCATTAGGAGTAGCACCCACCAAGGCAAAGAGAAGAGTG
GTGCAGAGAGAAAAAAGAGCAGTGGGAATAGGAGCTTTGTTCCTTGGGTTCTTGGGAGCAGCAGGAAGCACTATGGGCG
CAGCGTCAATGACGCTGACGGTACAGGCCAGACAATTATTGTCTGGTATAGTGCAGCAGCAGAACAATTTGCTGAGGGC
TATTGAGGCGCAACAGCATCTGTTGCAACTCACAGTCTGGGGCATCAAGCAGCTCCAGGCAAGAATCCTGGCTGTGGAA
AGATACCTAAAGGATCAACAGCTCCTGGGGATTTGGGGTTGCTCTGGAAAACTCATTTGCACCACTGCTGTGCCTTGGA
ATGCTAGTTGGAGTAATAAATCTCTGGAACAGATTTGGAATCACACGACCTGGATGGAGTGGGACAGAGAAATTAACAA
TTACACAAGCTTAATACACTCCTTAATTGAAGAATCGCAAAACCAGCAAGAAAAGAATGAACAAGAATTATTGGAATTA

GATAAATGGGCAAGTTTGTGGAATTGGTTTAACATAACAAATTGGCTGTGGTATATAAAATTATTCATAATGATAGTAG
GAGGCTTGGTAGGTTTAAGAATAGTTTTTGCTGTACTTTCTATAGTGAATAGAGTTAGGCAGGGATATTCACCATTATC
GTTTCAGACCCACCTCCCAACCCCGAGGGGACCCGACAGGCCCGAAGGAATAGAAGAAGAAGGTGGAGAGAGAGACAGA
GACAGATCCATTCGATTAGTGAACGGATCTCGACGGTATCGGTTAACTTTTAAAAGAAAAGGGGGGATTGGGGGGTACA
GTGCAGGGGAAAGAATAGTAGACATAATAGCAACAGACATACAAACTAAAGAATTACAAAAACAAATTACAAAATTCAA
AATTTTATCGATACTAGTATTATGCCCAGTACATGACCTTATGGGACTTTCCTACTTGGCAGTACATCTACGTATTAGT
CATCGCTATTACCATGGAGTAATTCATACAAAAGGACTCGCCCCTGCCTTGGGGAATCCCAGGGACCGTCGTTAAACTC
CCACTAACGTAGAACCCAGAGATCGCTGCGTTCCCGCCCCCTCACCCGCCCGCTCTCGTCATCACTGAGGTGGAGAAGA
GCATGCGTGAGGCTCCGGTGCCCGTCAGTGGGCAGAGCGCACATCGCCCACAGTCCCCGAGAAGTTGGGGGGAGGGGTC
GGCAATTGAACCGGTGCCTAGAGAAGGTGGCGCGGGGTAAACTGGGAAAGTGATGTCGTGTACTGGCTCCGCCTTTTTC
CCGAGGGTGGGGGAGAACCGTATATAAGTGCAGTAGTCGCCGTGAACGTTCTTTTTCGCAACGGGTTTGCCGCCAGAAC
ACAGGTAAGTGCCGTGTGTGGTTCCCGCGGGCCTGGCCTCTTTACGGGTTATGGCCCTTGCGTGCCTTGAATTACTTCC
ACGCCCCTGGCTGCAGTACGTGATTCTTGATCCCGAGCTTCGGGTTGGAAGTGGGTGGGAGAGTTCGAGGCCTTGCGCT
TAAGGAGCCCCTTCGCCTCGTGCTTGAGTTGAGGCCTGGCTTGGGCGCTGGGGCCGCCGCGTGCGAATCTGGTGGCACC
TTCGCGCCTGTCTCGCTGCTTTCGATAAGTCTCTAGCCATTTAAAATTTTTGATGACCTGCTGCGACGCTTTTTTTCTG
GCAAGATAGTCTTGTAAATGCGGGCCAAGATCTGCACACTGGTATTTCGGTTTTTGGGGCCGCGGGCGGCGACGGGGCC
CGTGCGTCCCAGCGCACATGTTCGGCGAGGCGGGGCCTGCGAGCGCGGCCACCGAGAATCGGACGGGGGTAGTCTCAAG
CTGGCCGGCCTGCTCTGGTGCCTGGCCTCGCGCCGCCGTGTATCGCCCCGCCCTGGGCGGCAAGGCTGGCCCGGTCGGC
ACCAGTTGCGTGAGCGGAAAGATGGCCGCTTCCCGGCCCTGCTGCAGGGAGCTCAAAATGGAGGACGCGGCGCTCGGGA
GAGCGGGCGGGTGAGTCACCCACACAAAGGAAAAGGGCCTTTCCGTCCTCAGCCGTCGCTTCATGTGACTCCACGGAGT
ACCGGGCGCCGTCCAGGCACCTCGATTAGTTCTCGAGCTTTTGGAGTACGTCGTCTTTAGGTTGGGGGGAGGGGTTTTA
TGCGATGGAGTTTCCCCACACTGAGTGGGTGGAGACTGAAGTTAGGCCAGCTTGGCACTTGATGTAATTCTCCTTGGAA
TTTGCCCTTTTTGAGTTTGGATCTTGGTTCATTCTCAAGCCTCAGACAGTGGTTCAAAGTTTTTTTCTTCCATTTCAGG
TGTCGTGATTCGAAGCCGCCACCATGGCCCTCCCTGTCACCGCCCTGCTGCTTCCGCTGGCTCTTCTGCTCCACGCCGC
TCGGCCCGACATCCAGATGACCCAGACCACAAGCAGCCTGTCTGCCAGCCTGGGCGATAGAGTGACCATCAGCTGTAGA
GCCAGCCAGGACATCAGCAAGTACCTGAACTGGTATCAGCAGAAACCCGACGGCACCGTGAAGCTGCTGATCTACCACA
CCAGCAGACTGCACAGCGGCGTGCCAAGCAGATTTTCTGGCAGCGGCTCTGGCACCGACTACAGCCTGACAATCAGCAA
CCTGGAACAAGAGGATATCGCTACCTACTTCTGCCAGCAAGGCAACACCCTGCCTTACACCTTTGGCGGAGGCACCAAG
CTGGAAATCACCGGAGGCGGAGGATCTGGTGGCGGAGGAAGTGGCGGAGGCGGTTCTGAAGTGAAGCTGCAAGAGTCTG
GCCCTGGACTGGTGGCCCCATCTCAGTCTCTGAGCGTGACCTGTACAGTCAGCGGAGTGTCCCTGCCTGATTACGGCGT
GTCCTGGATCAGACAGCCTCCTCGGAAAGGCCTGGAATGGCTGGGAGTGATCTGGGGCAGCGAGACAACCTACTACAAC
AGCGCCCTGAAGTCCCGGCTGACCATCATCAAGGACAACAGCAAGAGCCAGGTGTTCCTGAAGATGAACAGCCTGCAGA
CCGACGACACCGCCATCTACTACTGCGCCAAGCACTACTACTACGGCGGCAGCTACGCCATGGATTATTGGGGCCAGGG
CACCAGCGTGACCGTTTCTTCTACCACGACGCCAGCGCCGCGACCACCAACACCGGCGCCCACCATCGCGTCGCAGCCC
CTGTCCCTGCGCCCAGAAGCGTGCCGGCCAGCGGCGGGGGGCGCAGTGCACACGAGGGGGCTGGACTTCGCCTGTGATT
TCTGGGTGCTGGTCGTTGTGGGCGGCGTGCTGGCCTGCTACAGCCTGCTGGTGACAGTGGCCTTCATCATCTTTTGGGT
GAGGAGCAAGCGGAGCAGACTGCTGCACAGCGACTACATGAACATGACCCCCCGGAGGCCTGGCCCCACCCGGAAGCAC
TACCAGCCCTACGCCCCTCCCAGGGATTTCGCCGCCTACCGGAGCAAACGGGGCAGAAAGAAACTCCTGTATATATTCA
AACAACCATTTATGAGACCAGTACAAACTACTCAAGAGGAAGATGGCTGTAGCTGCCGATTTCCAGAAGAAGAAGAAGG
AGGATGTGAACTGAGAGTGAAGTTCAGCAGGAGCGCAGACGCCCCCGCGTACAAGCAGGGCCAGAACCAGCTCTATAAC
GAGCTCAATCTAGGACGAAGAGAGGAGTACGATGTTTTGGACAAGAGACGTGGCCGGGACCCTGAGATGGGGGGAAAGC
CGAGAAGGAAGAACCCTCAGGAAGGCCTGTACAATGAACTGCAGAAAGATAAGATGGCGGAGGCCTACAGTGAGATTGG
GATGAAAGGCGAGCGCCGGAGGGGCAAGGGGCACGATGGCCTTTACCAGGGTCTCAGTACAGCCACCAAGGACACCTAC
GACGCCCTTCACATGCAGGCCCTGCCCCCTCGCTAATAATCTAGAAATCAACCTCTGGATTACAAAATTTGTGAAAGAT
TGACTGGTATTCTTAACTATGTTGCTCCTTTTACGCTATGTGGATACGCTGCTTTAATGCCTTTGTATCATGCTATTGC
TTCCCGTATGGCTTTCATTTTCTCCTCCTTGTATAAATCCTGGTTGCTGTCTCTTTATGAGGAGTTGTGGCCCGTTGTC
AGGCAACGTGGCGTGGTGTGCACTGTGTTTGCTGACGCAACCCCCACTGGTTGGGGCATTGCCACCACCTGTCAGCTCC
TTTCCGGGACTTTCGCTTTCCCCCTCCCTATTGCCACGGCGGAACTCATCGCCGCCTGCCTTGCCCGCTGCTGGACAGG
GGCTCGGCTGTTGGGCACTGACAATTCCGTGGTGTTGTCGGGGAAATCATCGTCCTTTCCTTGGCTGCTCGCCTGTGTT
GCCACCTGGATTCTGCGCGGGACGTCCTTCTGCTACGTCCCTTCGGCCCTCAATCCAGCGGACCTTCCTTCCCGCGGCC
TGCTGCCGGCTCTGCGGCCTCTTCCGCGTCTTCGCCTTCGCCCTCAGACGAGTCGGATCTCCCTTTGGGCCGCCTCCCC

GCCTGGTACCAATTCTACCGGGTAGGGGAGGCGCTTTTCCCAAGGCAGTCTGGAGCATGCGCTTTAGCAGCCCCGCTGG
GCACTTGGCGCTACACAAGTGGCCTCTGGCCTCGCACACATTCCACATCCACCGGTAGGCGCCAACCGGCTCCGTTCTT
TGGTGGCCCCTTCGCGCCACCTTCTACTCCTCCCCTAGTCAGGAAGTTCCCCCCCGCCCCGCAGCTCGCGTCGTGCAGG
ACGTGACAAATGGAAGTAGCACGTCTCACTAGTCTCGTGCAGATGGACAGCACCGCTGAGCAATGGAAGCGGGTAGGCC
TTTGGGGCAGCGGCCAATAGCAGCTTTGCTCCTTCGCTTTCTGGGCTCAGAGGCTGGGAAGGGGTGGGTCCGGGGGCGG
GCTCAGGGGCGGGCTCAGGGGCGGGGCGGGCGCCCGAAGGTCCTCCGGAGGCCCGGCATTCTGCACGCTTCAAAAGCGC
ACGTCTGCCGCGCTGTTCTCCTCTTCCTCATCTCCGGGCCTTTCGACCTGCAGCCCAAGCTTGCCACCATGGTGAGCAA
GGGCGAGGAGCTGTTCACCGGGGTGGTGCCCATCCTGGTCGAGCTGGACGGCGACGTAAACGGCCACAAGTTCAGCGTG
TCCGGCGAGGGCGAGGGCGATGCCACCTACGGCAAGCTGACCCTGAAGTTCATCTGCACCACCGGCAAGCTGCCCGTGC
CCTGGCCCACCCTCGTGACCACCCTGACCTACGGCGTGCAGTGCTTCAGCCGCTACCCCGACCACATGAAGCAGCACGA
CTTCTTCAAGTCCGCCATGCCCGAAGGCTACGTCCAGGAGCGCACCATCTTCTTCAAGGACGACGGCAACTACAAGACC
CGCGCCGAGGTGAAGTTCGAGGGCGACACCCTGGTGAACCGCATCGAGCTGAAGGGCATCGACTTCAAGGAGGACGGCA
ACATCCTGGGGCACAAGCTGGAGTACAACTACAACAGCCACAACGTCTATATCATGGCCGACAAGCAGAAGAACGGCAT
CAAGGTGAACTTCAAGATCCGCCACAACATCGAGGACGGCAGCGTGCAGCTCGCCGACCACTACCAGCAGAACACCCCC
ATCGGCGACGGCCCCGTGCTGCTGCCCGACAACCACTACCTGAGCACCCAGTCCGCCCTGAGCAAAGACCCCAACGAGA
AGCGCGATCACATGGTCCTGCTGGAGTTCGTGACCGCCGCCGGGATCACTCTCGGCATGGACGAGCTGTACAAGGGTTC
TGGAGCCACGAACTTCTCTCTGTTAAAGCAAGCAGGAGACGTGGAAGAAAACCCCGGTCCTATGGCCAAGTTGACCAGT
GCCGTTCCGGTGCTCACCGCGCGCGACGTCGCCGGAGCGGTCGAGTTCTGGACCGACCGGCTCGGGTTCTCCCGGGACT
TCGTGGAGGACGACTTCGCCGGTGTGGTCCGGGACGACGTGACCCTGTTCATCAGCGCGGTCCAGGACCAGGTGGTGCC
GGACAACACCCTGGCCTGGGTGTGGGTGCGCGGCCTGGACGAGCTGTACGCCGAGTGGTCGGAGGTCGTGTCCACGAAC
TTCCGGGACGCCTCCGGGCCGGCCATGACCGAGATCGGCGAGCAGCCGTGGGGGCGGGAGTTCGCCCTGCGCGACCCGG
CCGGCAACTGCGTGCACTTTGTGGCAGAGGAGCAGGACTGATAATTTAAGACCAATGACTTACAAGGCAGCTGTAGATC
TTAGCCACTTTTTAAAAGAAAAGGGGGGACTGGAAGGGCTAATTCACTCCCAACGAAGATAAGATCTGCTTTTTGCTTG
TACTGGGTCTCTCTGGTTAGACCAGATCTGAGCCTGGGAGCTCTCTGGCTAACTAGGGAACCCACTGCTTAAGCCTCAA
TAAAGCTTGCCTTGAGTGCTTCAAGTAGTGTGTGCCCGTCTGTTGTGTGACTCTGGTAACTAGAGATCCCTCAGACCCT
TTTAGTCAGTGTGGAAAATCTCTAGCA

**Table S1: Support for the putative SNVs or deletion in PacBio and Illumina reads**

The observed fraction of PacBio and Illumina reads supporting each variant is generally consistent with the expected fraction based on the fraction of insertions in the respective VCN clone containing the variant. For example, in VCN2, each SNV is in one of two integrations in the clone, so we would expect approximately 50 % of reads for that integration site to contain a SNV at that position. SNVs in both VCN4 and VCN5 are based on expected ALT read support for five integrations (VCN5). The VCN4/5 deletion at 5579 occurs in a region covered by a partial integration (so that the denominator is 6 total integrations). Differences from expectation may reflect limited read depth, mapping ambiguity, and platform-specific error profiles. SNVs all came from reads with supplementary alignments to the expected chromosomes. For the deletion at 5579, the expected percent support for ALT is based on VCN5 (as VCN4 was not sequenced with PacBio); the 2 non-chr6 reads supporting this deletion originated from insertions on chr1 and chr5, likely due to the known higher indel error rate in PacBio. Overall, the concordance between expected and observed read evidence provides support for variants inside integrated sequences.

| **VCN clone** | **VCN2** | **VCN2** | **VCN2** | **VCN2** | **VCN3** | **VCN4/5** | **VCN4/5** | **VCN4/5** |
|---|---|---|---|---|---|---|---|---|
| **position in expected proviral sequence** | **1692** | **1693** | **4878** | **6601** | **1450** | **1066** | **3649** | **5579** |
| chromosome of integrated sequence with SNV or DEL in pbsv call | chr19 | chr19 | chr19 | chr19 | chr1 | chr1 | chr3 | chr1/5/6 |
| REF | A | C | G | G | T | G | G | C |
| ALT | T | T | C | A | A | A | A | DEL |
| **PACBIO - PBMM2** | | | | | | | | |
| read support for ALT | 10 | 10 | 7 | 6 | 12 | 7 | 7 | 1/1/4 |
| total reads | 16 | 16 | 10 | 10 | 32 | 42 | 38 | 32 |
| **expected % support for ALT** | **50** | **50** | **50** | **50** | **33** | **20** | **20** | **17** |
| **observed % support for ALT** | **63** | **63** | **70** | **60** | **38** | **17** | **18** | **19** |
| **ILLUMINA - BWA** | | | | | | | | |
| read support for ALT | 8 | 8 | 15 | 17 | 20 | 4 | 14 | 6 |
| total reads | 28 | 28 | 35 | 34 | 54 | 44 | 47 | 58 |
| **expected % support for ALT** | **50** | **50** | **50** | **50** | **33** | **20** | **20** | **17** |
| **observed % support for ALT** | **29** | **29** | **43** | **50** | **37** | **9** | **30** | **10** |

**Table S2: Summary of integration sites for each VCN clone**
Table shows summary of all integration sites by VCN clone. The IS_ID corresponds to the chromosome integration site; IS_IDs can be shared across VCN clones. Integration positions on the chromosome (start/end) are the first and last base of the integration site, they are not left aligned. Positions of abnormalities are relative to the expected proviral sequence, not the position within the sequence reported in Supplemental Text. All integrations should begin with the 4 - 5 bp integration site sequence. This sequence is duplicated upon integration which can be seen as increased coverage in IGV. Homology between integration site and proviral sequence was observed as increased coverage making integration site sequences appear longer.

| VCN | alignments performed | mosdepth median autosome coverage (BWA - pbmm2) | IS_ID | chromosome | start | end | integration site sequence | orientation | Integrated sequence length | integrated sequence fasta header name (Supplemental Text) |
|---|---|---|---|---|---|---|---|---|---|---|
| VCN0 | ILMN (BWA) | 28.5x - NA | NA | NA | NA | NA | NA | NA | 0 | expected_full_provirus_sequence_7374bp |
| VCN1 | ILMN (BWA) | 27x - NA | IS1 | chr17 | 78,730,345 | 78,730,349 | GAAAC | - | 6655 | IS1_VCN1-VCN2-VCN3-VCN4-VCN5_chr17:78730345 |
| VCN2 | HiFi (pbmm2), ILMN (BWA), pbsvINS (pbmm2) | 28.7x - 44.4x | IS1 | chr17 | 78,730,345 | 78,730,349 | GAAAC | - | 6655 | IS1_VCN1-VCN2-VCN3-VCN4-VCN5_chr17:78730345 |
| VCN2 | HiFi (pbmm2), ILMN (BWA), pbsvINS (pbmm2) | 28.7x - 44.4x | IS2 | chr19 | 21,610,872 | 21,610,876 | CCCCC | - | 7379 | IS2_VCN2_chr19:21610872 |
| VCN3 | HiFi (pbmm2), ILMN (BWA), pbsvINS (pbmm2) | 27.2x - 37.9x | IS3 | chr1 | 23,307,863 | 23,307,867 | ACAGG | - | 8183 | IS3_VCN3_chr1:23307863 |
| VCN3 | HiFi (pbmm2), ILMN (BWA), pbsvINS (pbmm2) | 27.2x - 37.9x | IS4 | chr8 | 99,830,407 | 99,830,410 | AAAC | - | 263 | IS4_VCN3_chr8:99830407 |
| VCN3 | HiFi (pbmm2), ILMN (BWA), pbsvINS (pbmm2) | 27.2x - 37.9x | IS1 | chr17 | 78,730,345 | 78,730,349 | GAAAC | - | 6655 | IS1_VCN1-VCN2-VCN3-VCN4-VCN5_chr17:78730345 |
| VCN3 | HiFi (pbmm2), ILMN (BWA), pbsvINS (pbmm2) | 27.2x - 37.9x | IS5 | chrX | 103,930,456 | 103,930,460 | AATAC | - | 7379 | IS5_VCN3_chrX:103930456 |
| VCN4 | ILMN (BWA) | 25.3x - NA | IS6 | chr1 | 243,273,502 | 243,273,506 | ACCCC | + | 7420 | IS6_VCN4-VCN5_chr1:243273502 |
| VCN4 | ILMN (BWA) | 25.3x - NA | IS7 | chr3 | 18,115,277 | 18,115,281 | TACTC | - | 7379 | IS7_VCN4-VCN5_chr3:18115277 |
| VCN4 | ILMN (BWA) | 25.3x - NA | IS8 | chr6 | 15,601,040 | 15,601,062 | possibly AG | - | 2070 | IS8_VCN4-VCN5_chr6:15601040 |
| VCN4 | ILMN (BWA) | 25.3x - NA | IS9 | chr6 | 24,825,065 | 24,825,069 | GTACT | + | 7379 | IS9_VCN4-VCN5_chr6:24825065 |
| VCN4 | ILMN (BWA) | 25.3x - NA | IS1 | chr17 | 78,730,345 | 78,730,349 | GAAAC | - | 6655 | IS1_VCN1-VCN2-VCN3-VCN4-VCN5_chr17:78730345 |

| | | | | | | | | | | |
|---|---|---|---|---|---|---|---|---|---|---|
| VCN5 | HiFi (pbmm2), ILMN (BWA), pbsvINS (pbmm2) | 24.7x - 31.3x | IS6 | chr1 | 243,273,502 | 243,273,506 | ACCCC | + | 7420 | IS6_VCN4-VCN5_chr1:243273502 |
| VCN5 | HiFi (pbmm2), ILMN (BWA), pbsvINS (pbmm2) | 24.7x - 31.3x | IS7 | chr3 | 18,115,277 | 18,115,281 | TACTC | - | 7379 | IS7_VCN4-VCN5_chr3:18115277 |
| VCN5 | HiFi (pbmm2), ILMN (BWA), pbsvINS (pbmm2) | 24.7x - 31.3x | IS10 | chr5 | 39,044,696 | 39,044,700 | TTCAC | - | 7379 | IS10_VCN5_chr5:39044696 |
| VCN5 | HiFi (pbmm2), ILMN (BWA), pbsvINS (pbmm2) | 24.7x - 31.3x | IS8 | chr6 | 15,601,040 | 15,601,062 | possibly AG | - | 2070 | IS8_VCN4-VCN5_chr6:15601040 |
| VCN5 | HiFi (pbmm2), ILMN (BWA), pbsvINS (pbmm2) | 24.7x - 31.3x | IS9 | chr6 | 24,825,065 | 24,825,069 | GTACT | + | 7379 | IS9_VCN4-VCN5_chr6:24825065 |
| VCN5 | HiFi (pbmm2), ILMN (BWA), pbsvINS (pbmm2) | 24.7x - 31.3x | IS1 | chr17 | 78,730,345 | 78,730,349 | GAAAC | - | 6655 | IS1_VCN1-VCN2-VCN3-VCN4-VCN5_chr17:78730345 |

**Table S3: Summary of integration site observations**

This table builds upon the genomic loci identified in Table S2 to describe the precise composition and sequence integrity of each specific integration event. It includes the VCN clone identifier and the unique integration site identifier (IS_ID), allowing for direct cross-referencing with the genomic coordinates and sequencing metrics in Table S2. The table characterizes integration site , specifically noting the expected length. Furthermore, it provides a high-level categorization of the proviral integration state—such as full-length integration, incomplete integration, or integration with structural abnormality— alongside detailed descriptions of any sequence variations.

| VCN | IS_ID | integration site observations | integration integrity | integrity notes |
|---|---|---|---|---|
| VCN0 | NA | NA | NA | control, jurkat genome with no integrations |
| VCN1 | IS1 | 5 bp as expected | integration with structural abnormality | Structural abnormality with 724 bp deletion of expected sequence, inferred from HiFi sequencing of VCN2, VCN3 and VCN5. VCN2-5 derives from VCN1. |
| VCN2 | IS1 | 5 bp as expected | integration with structural abnormality | Large 724 bp deletion in middle of the integrated sequence (bases 1917-2640 of expected sequence) in the EF1α promoter region. |
| VCN2 | IS2 | 5 bp as expected | full length integration | Four SNVs identified in integrated sequence at expected sequence positions 1692: A -> T, 1693: C -> T, 4878: G -> C and 6601: G -> A. Other than SNVs, no other abnormalities. |
| VCN3 | IS3 | 5 bp as expected | integration with structural abnormality | Structural abnormality at the 3' end of a full-length integration. The abnormality contains additional sequence composed of a large duplication, corresponding to expected integration sequence positions 4416 - 5193 (778 bp), followed by 2bp (GT) then 24 bps of the 3' LTR sequence (AGTCAGTGTGGAAAATCTCTAGCA). Within the full-length portion of this integration, there is a SNV T->A at position 1450. |
| VCN3 | IS4 | 4 bp integration site sequence | incomplete integration | A short 263 bp integrated sequence primarily composed of LTR sequence was identified. The sequence begins with a four base-pair duplication of the chr8 integration site. Following this duplication, 159 bp correspond to LTR positions 7216–7374 of the expected integration. The remaining 100 bp are less clearly defined but appear to include a mixture of unknown and LTR-derived sequence (33 bp unknown, 21 bp possible LTR, and 46 bp unknown using BLAST). BLAST of the 79 bp of "unknown" sequence was performed and no significant similarities were found. |
| VCN3 | IS1 | 5 bp as expected | integration with structural abnormality | Large 724 bp deletion in middle of the integrated sequence (bases 1917-2640 of expected sequence) in the EF1α promoter region. |
| VCN3 | IS5 | 5 bp as expected | full length integration | none |
| VCN4 | IS6 | **ACCCC**T: homology ("T") between chromosome and 1st base of integrated sequence. This resulted in "T" appearing duplicated in coverage plot at integration site, but it is not part of the duplicated integration site sequence. | integration with structural abnormality | Structural abnormality inferred from VCN5 HiFi sequencing. VCN5 derives from VCN4. |
| VCN4 | IS7 | **TACTC**T: homology ("T") between chromosome and 1st base of integrated sequence. This resulted in "T" appearing duplicated in coverage plot at integration site, but it is not part of the duplicated integration site sequence. | full length integration | Full length integration with SNV G->A at integrated sequence position 3649 inferred from VCN5 HiFi sequencing. VCN5 was derived from VCN4. There is some evidence for SNV in VCN4 ILMN reads. |
| VCN4 | IS8 | integration site sequence is unclear | incomplete integration | Incomplete integration inferred from VCN5 HiFi sequencing. VCN5 derives from VCN4. |
| VCN4 | IS9 | **GTACT**T: homology ("T") between chromosome and 1st base of integrated sequence. This resulted in "T" appearing duplicated in coverage plot at integration site, but it is not part of the duplicated integration site sequence. | full length integration | full length integration inferred from VCN5, which was sequenced with HiFi. VCN5 derives from VCN4. |
| VCN4 | IS1 | 5 bp as expected | integration with structural abnormality | Structural abnormality with 724 bp deletion of expected sequence inferred from HiFi sequencing of VCN2, VCN3 and VCN5. VCN4 derives from VCN1, where IS1 originated. VCN4 was then used to generate VCN5. |
| VCN5 | IS6 | **ACCCC**T: homology ("T") between chromosome and 1st base of integrated sequence. This resulted in "T" appearing | integration with structural abnormality | SNV G->A at position 1066 in full integrated sequence. End of full integrated sequence has an additional 41 bp. This sequence is somewhat unclear and but appears to be |

| | | | | |
|---|---|---|---|---|
| | | duplicated in coverage plot at integration site, but it is not part of the duplicated integration site sequence. | | comprised of 15 bases of unknown origin followed by the first 26bp of the LTR sequence in reverse orientation. |
| VCN5 | IS7 | **TACTC**T: homology ("T") between chromosome and 1st base of integrated sequence. This resulted in "T" appearing duplicated in coverage plot at integration site, but it is not part of the duplicated integration site sequence. | full length integration | SNV G->A at full integrated sequence position 3649. Other than SNV, no other abnormalities. |
| VCN5 | IS10 | CA**TTCAC:** homology ("CA") between chromosome and last two bases of integrated sequence. This resulted in "CA" appearing duplicated in coverage plot at integration site, but it is not part of the duplicated integration site sequence. | full length integration | none |
| VCN5 | IS8 | integration site sequence is unclear | incomplete integration | Integrated sequence is incomplete (2070 bp) and only contains bases corresponding to positions 5306 - 7374 of the expected integration sequence. It appears that 22 bases of chr6 were deleted at the same time as the insertion, from chr6:15,601,040 - 15,601,062. This 22bp region on chr6 has reduced coverage, likely as part of deletion, and numerous SNVs relative to chr6 due to misalignment of the integrated sequence. The pbsv call appears to be 24bp shorter compared with aligned read (clipped sequence) due to imperfect alignments to the deleted region. 22/24 bases, "CGAAGACGCGGAAGAGGCCGCA", align to the expected integration sequence, but the final two bases, "AG", do not. These two bases are part of chr6 sequence adjacent to deleted portion of chr6. It is possible these two bases are duplicated upon integration. There is also a deletion of a "C" at position 5579 relative to the expected sequence; this "C" is part of a string of Cs. |
| VCN5 | IS9 | **GTACT**T: homology ("T") between chromosome and 1st base of integrated sequence. This resulted in "T" appearing duplicated in coverage plot at integration site, but it is not part of the duplicated integration site sequence. | full length integration | none |
| VCN5 | IS1 | 5 bp as expected | integration with structural abnormality | Large 724 bp deletion in middle of the integrated sequence (bases 1917-2640 of expected sequence) in the EF1α promoter region. |

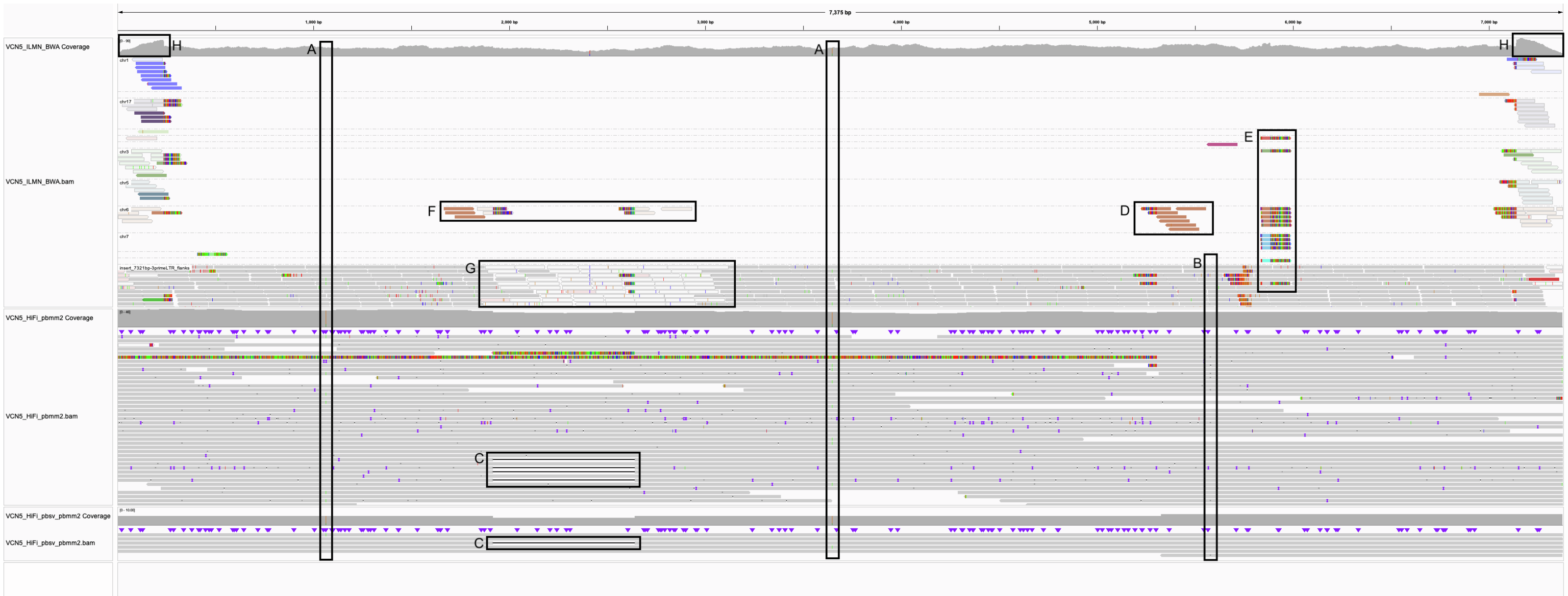


**Figure S1: Alignments of Illumina (ILMN) reads, PacBio HiFi reads, and PacBio pbsv variant calls to the provirus sequence for VCN5**

The Illumina track is grouped by ”chromosome of mate” to highlight integration sites. Visualization with IGV identified specific variants and mapping artifacts annotated as follows: A) SNVs observed on integrated sequence in chr1 and chr3. Note, IGV "coverage allele fraction threshold" = 0.15. B) DEL observed on integrated sequence in chr6. Given the extra insertion (IS8) in VCN5, it was expected that the DEL would be observed in 17% of reads. While not visible in the screen shot, 10 % (6/58) of Illumina reads contained the DEL. The DEL can be seen in HiFi reads and was observed in 19 % (6/32) of reads. With HiFi reads it was possible to see supplementary alignments of the reads supporting the deletion, with four correctly on chr6 but two from other chromosomes, likely due to indel errors in HiFi. C) 724 bp deletion of promoter in integration sequence on chr17. D) Partial insertion (2070 bp) on chr6. E) VCN5, but not other VCNs, have Illumina reads partially aligned to ~60 bp of provirus sequence, but this is not seen in HiFi reads in any VCN. F) Illumina read mates map incorrectly with MQ0 to the *EEF1A1* (chr6:73.49-73.53) gene promoter in GRCh38 because this human gene promoter was used in the provirus. This was observed with all VCN Illumina reads. G) Region has homology with *EEF1A1* gene in GRCh38, causing zero mapping quality in Illumina data but not long reads. This was observed with all VCN Illumina reads. H) Flanking LTR regions have high coverage of Illumina reads with low MQ because these sequences are identical.

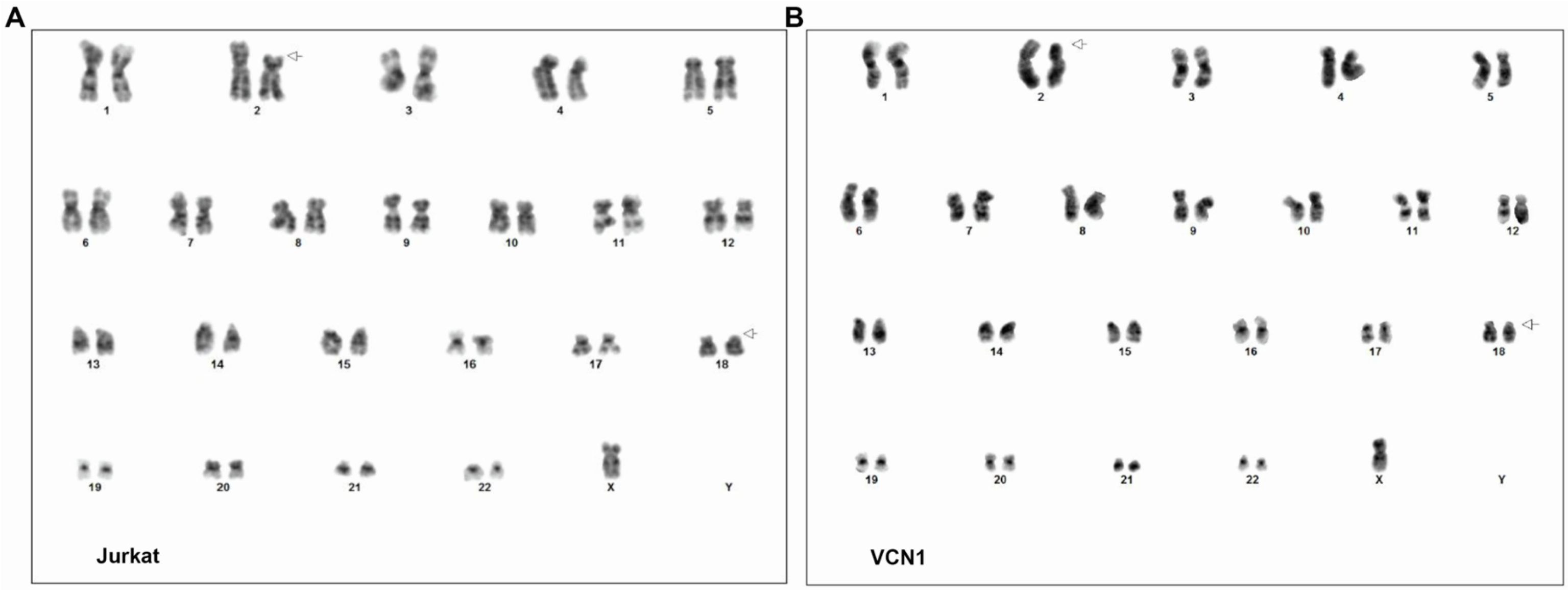


**Figure S2: G-band Karyograms of Jurkat cells and clonal cell line**
A subpopulation of Jurkat cells and the clonal cell line VCN1 lacked the Y chromosome. A) G-band karyotype analysis revealed that a subpopulation (7 out of 20 metaphase spreads) of Jurkat cells lacked the Y chromosome. B) All VCN1 metaphase spreads exhibited a lack of the Y chromosome

# IS1_VCN1-VCN2-VCN3-VCN4-VCN5_chr17:78730345

## PacBio HiFi

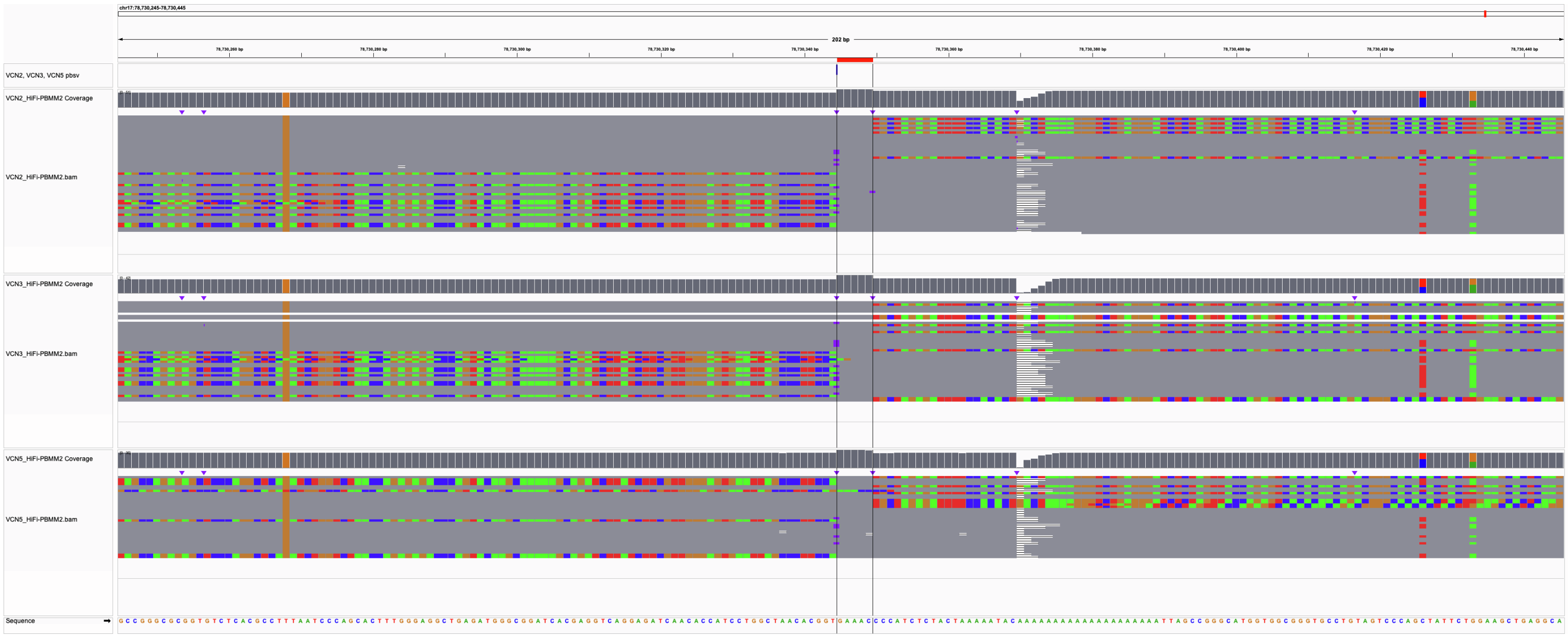


## Illumina

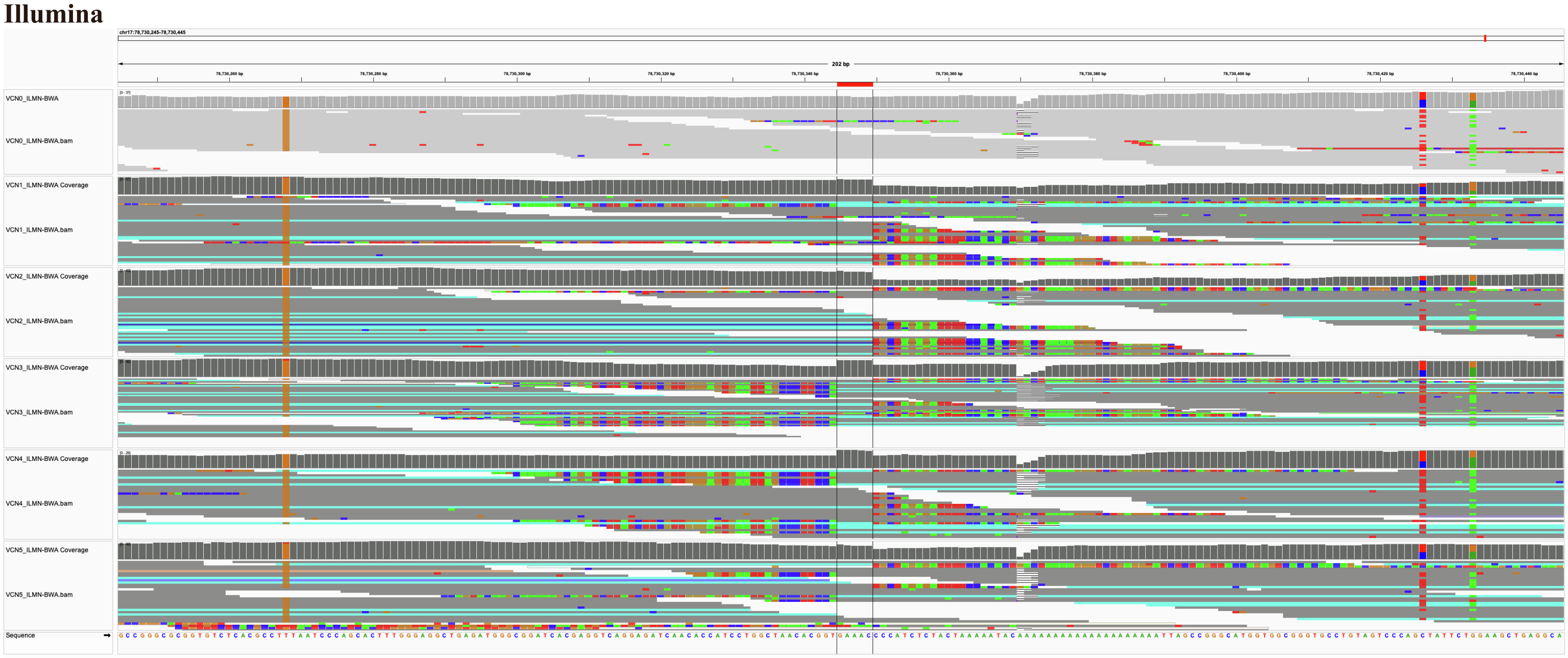

# IS2_VCN2_chr19:21610872

## PacBio HiFi

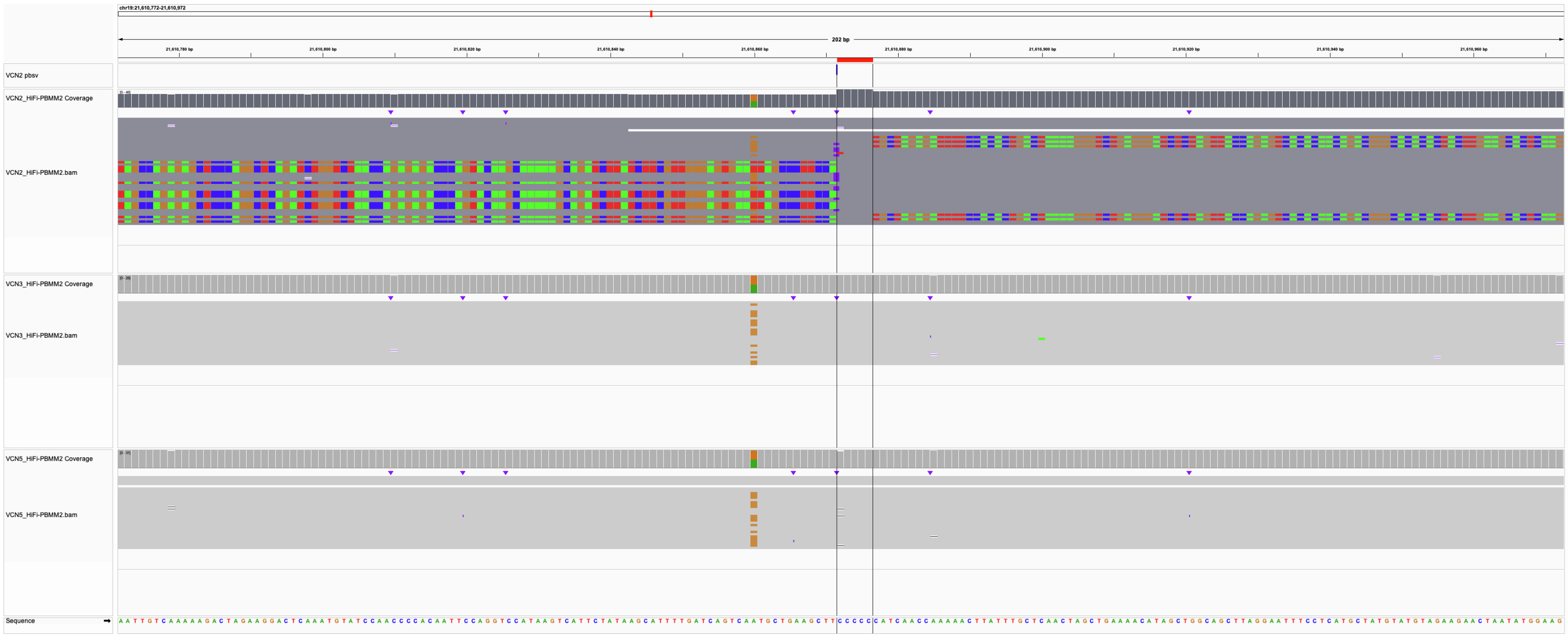

## Illumina

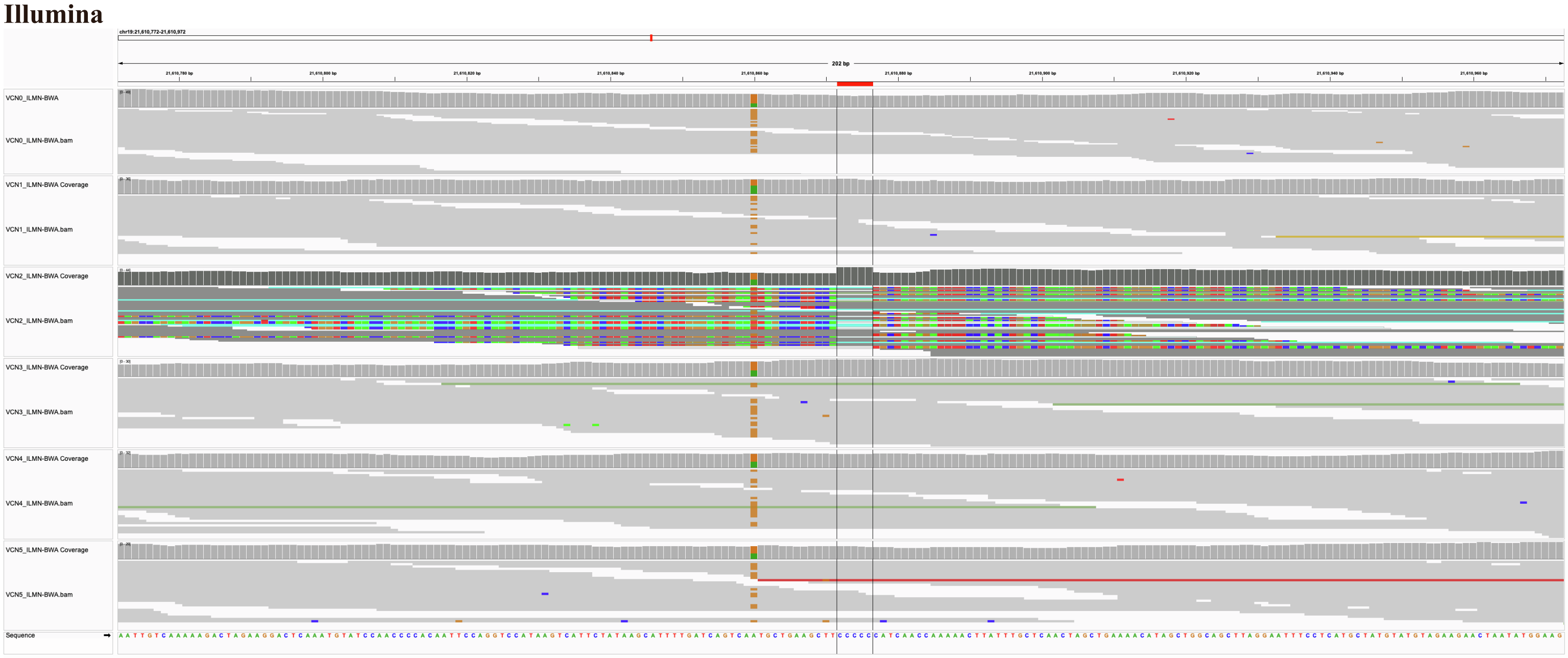

## IS3_VCN3_chr1:23307863

### HiFi

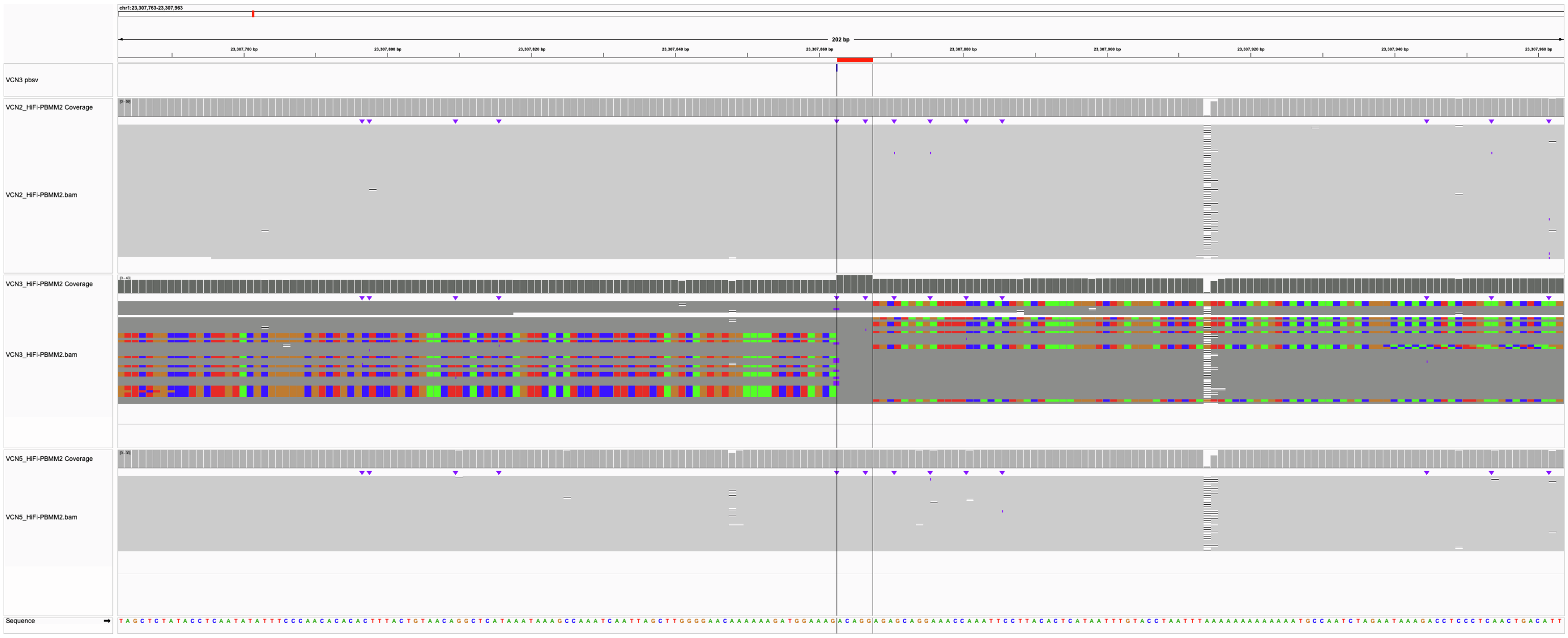

### Illumina

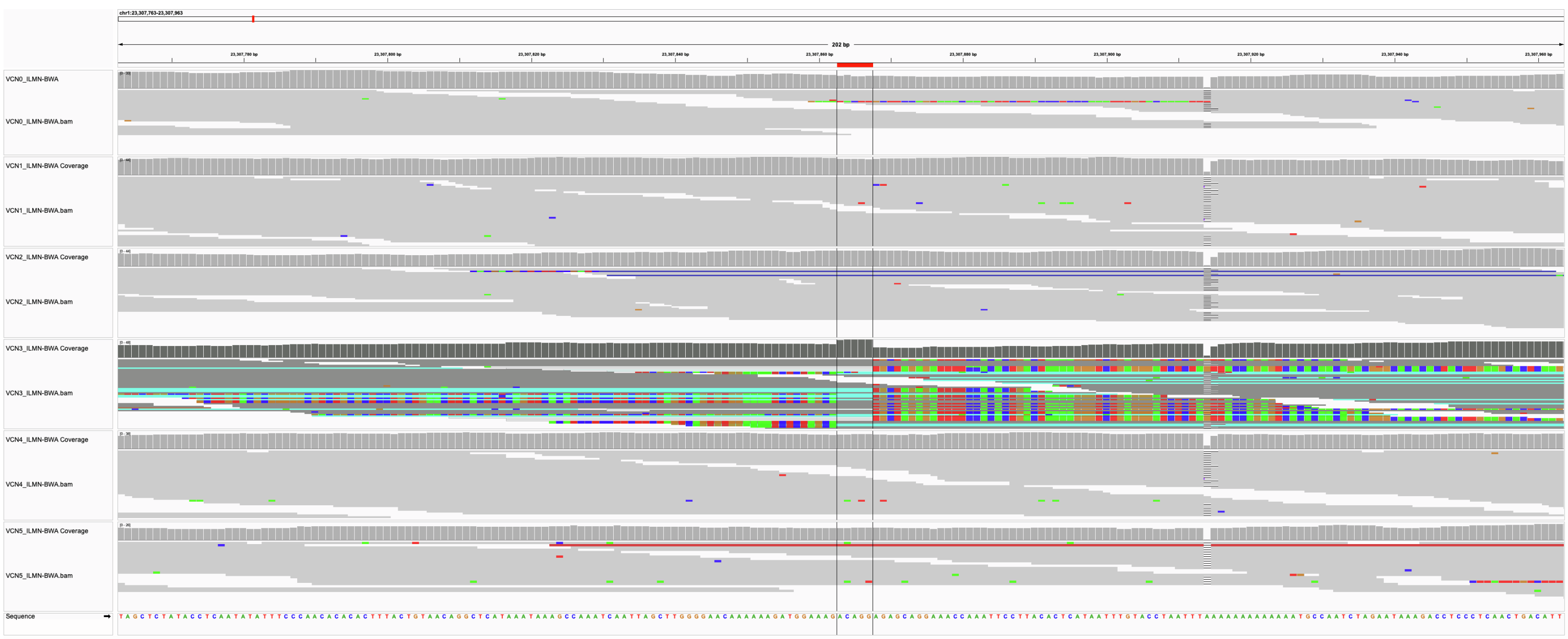

## IS4_VCN3_chr8:99830407

### HiFi

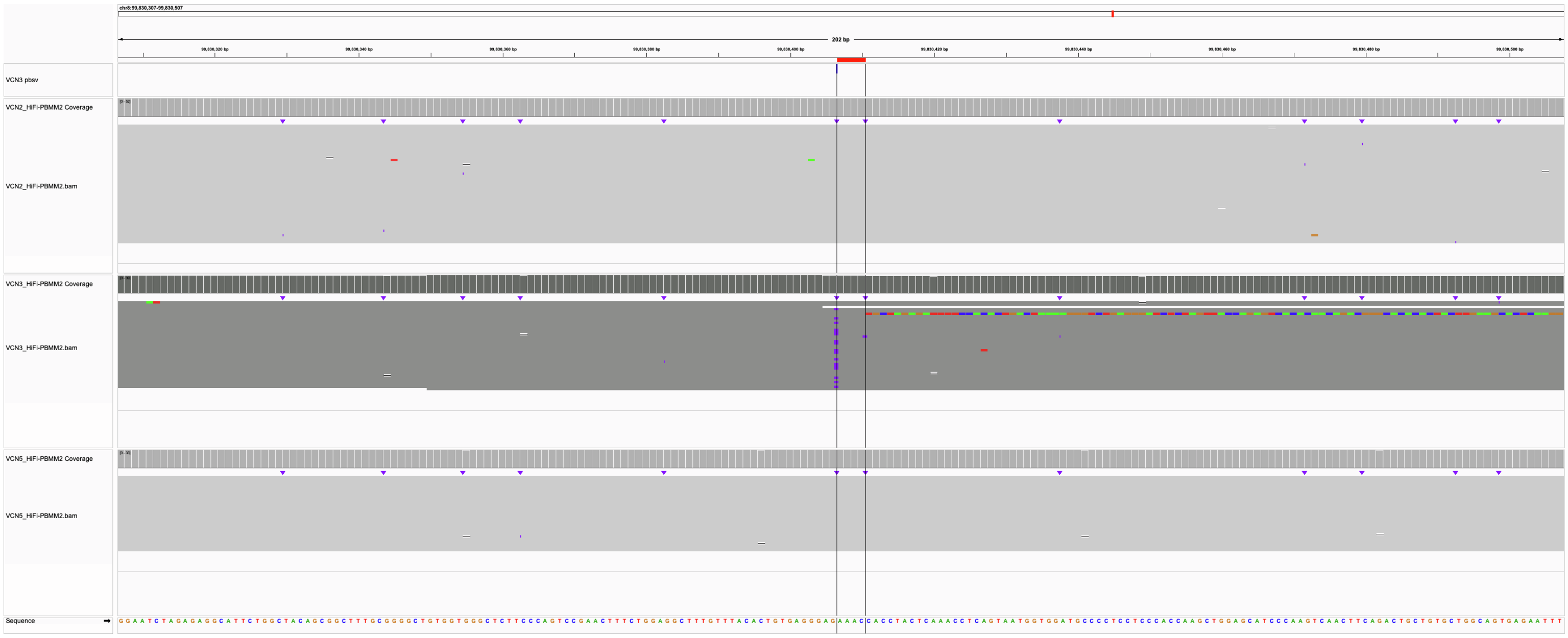


### Illumina

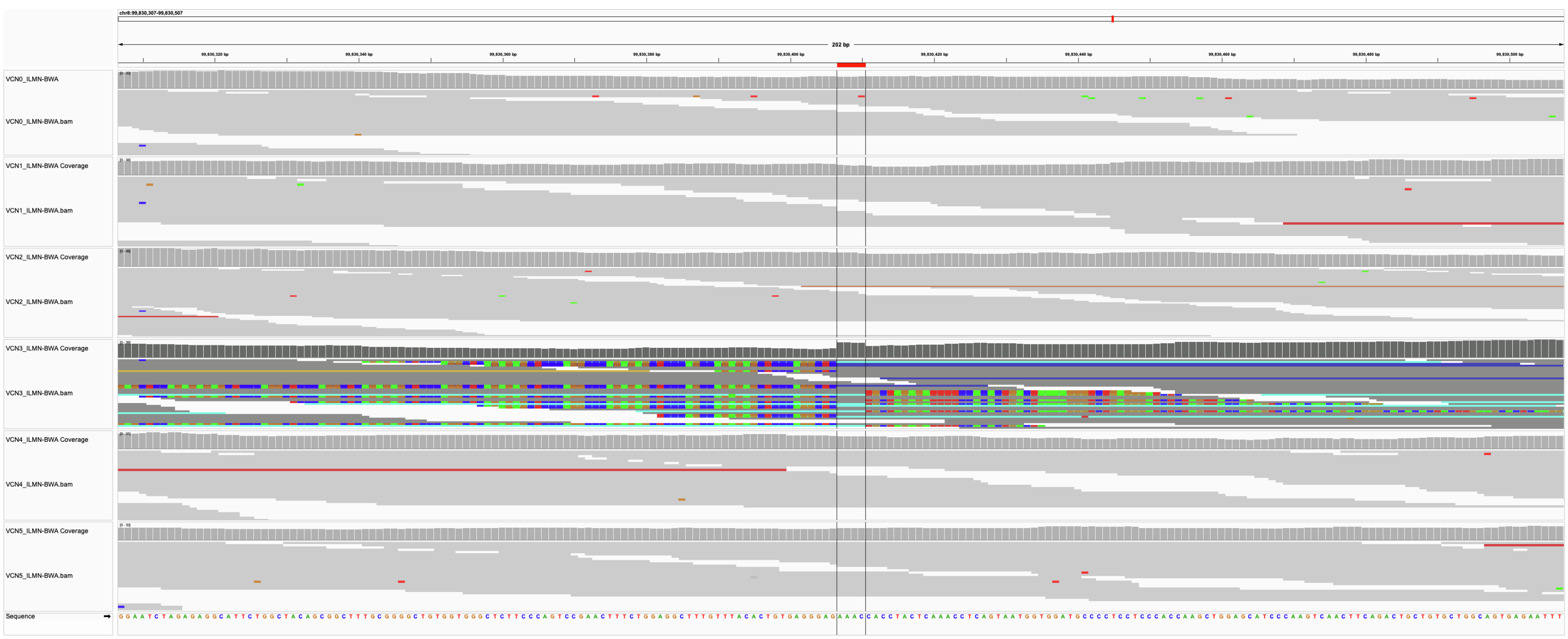

# IS5_VCN3_chrX:103930456

## HiFi

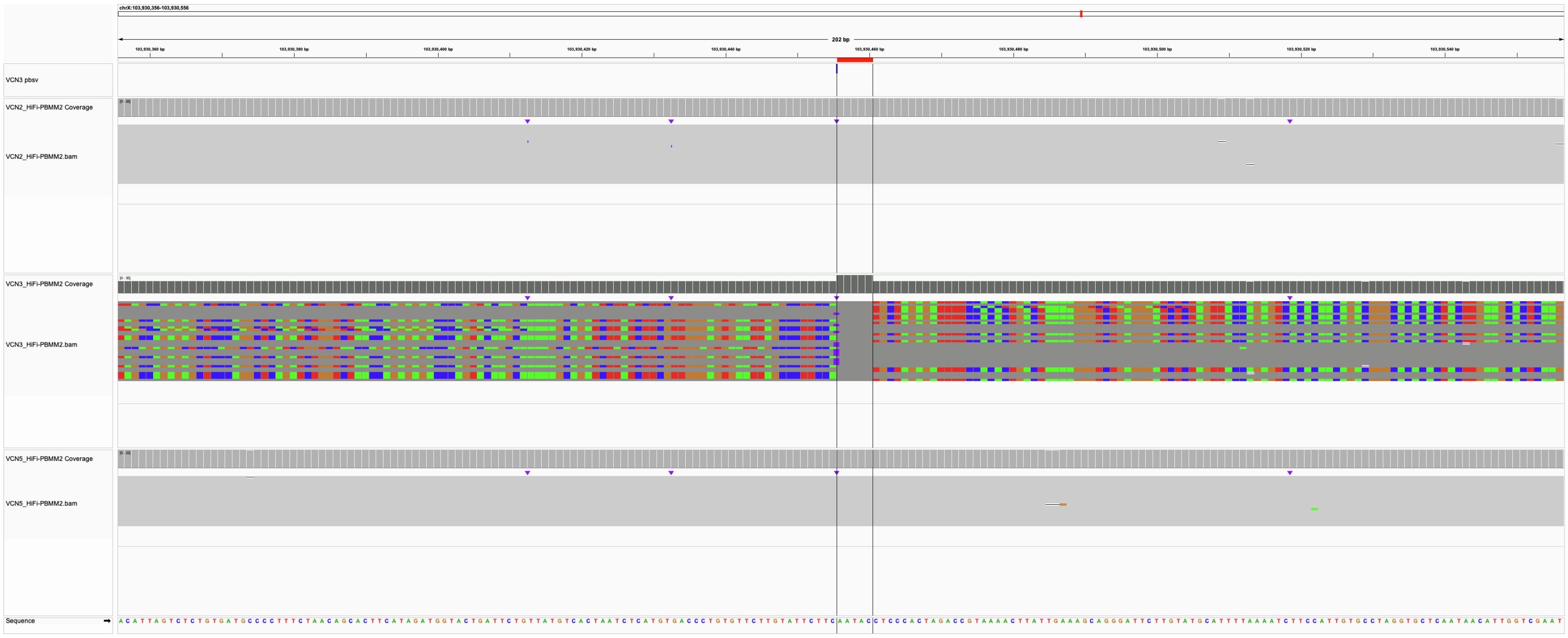

## Illumina

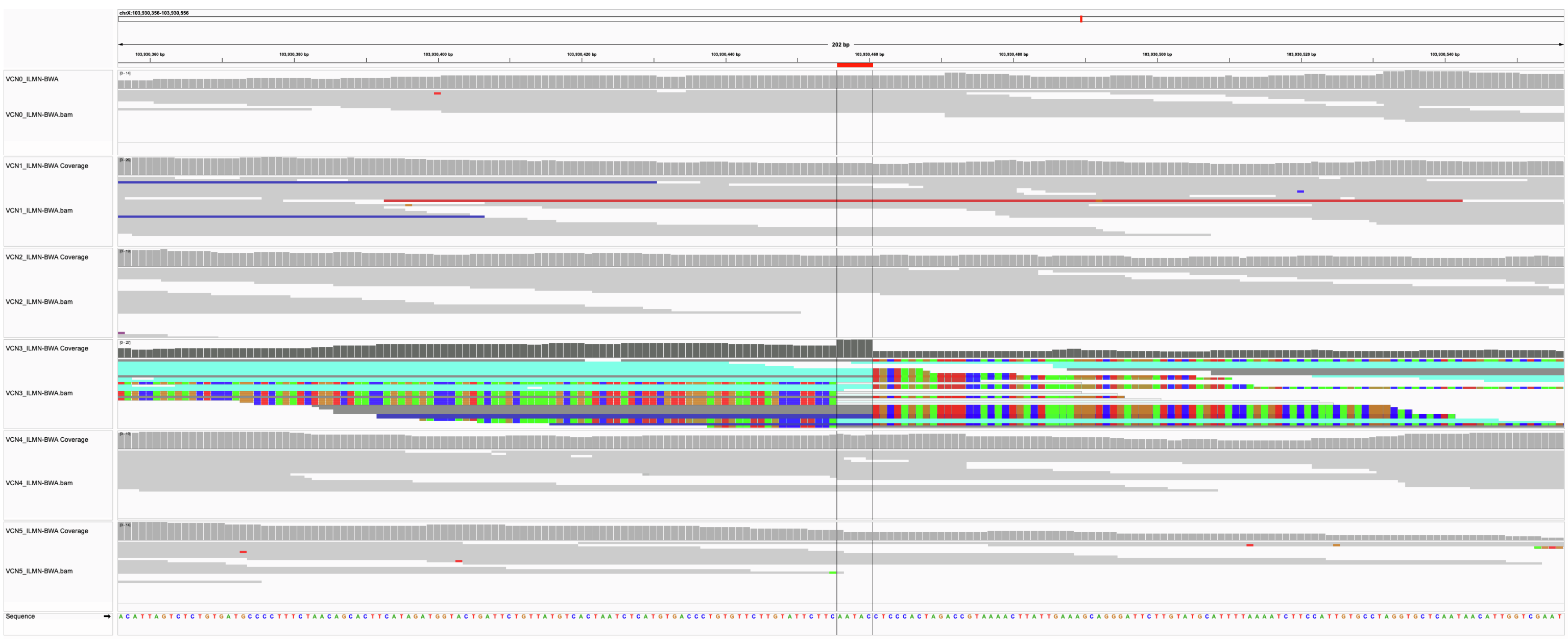

# IS6_VCN4-VCN5_chr1:243273502

## HiFi

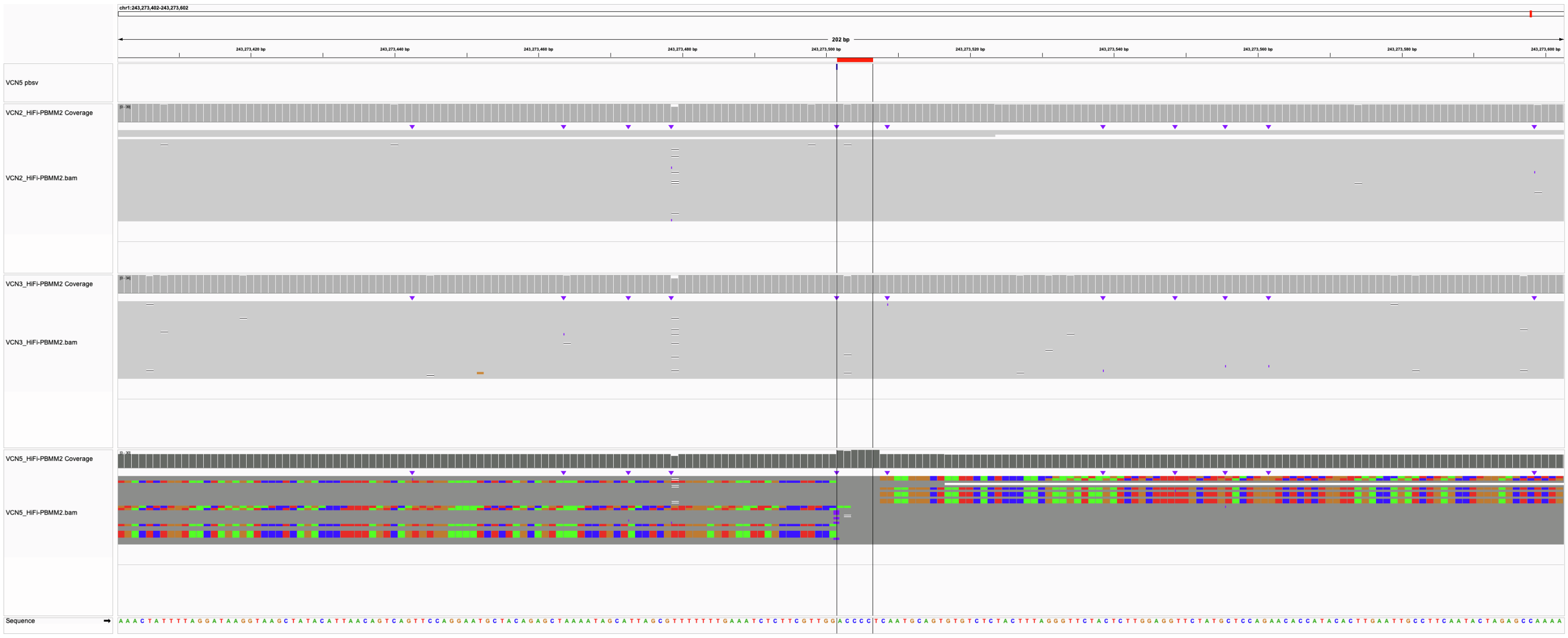

## Illumina

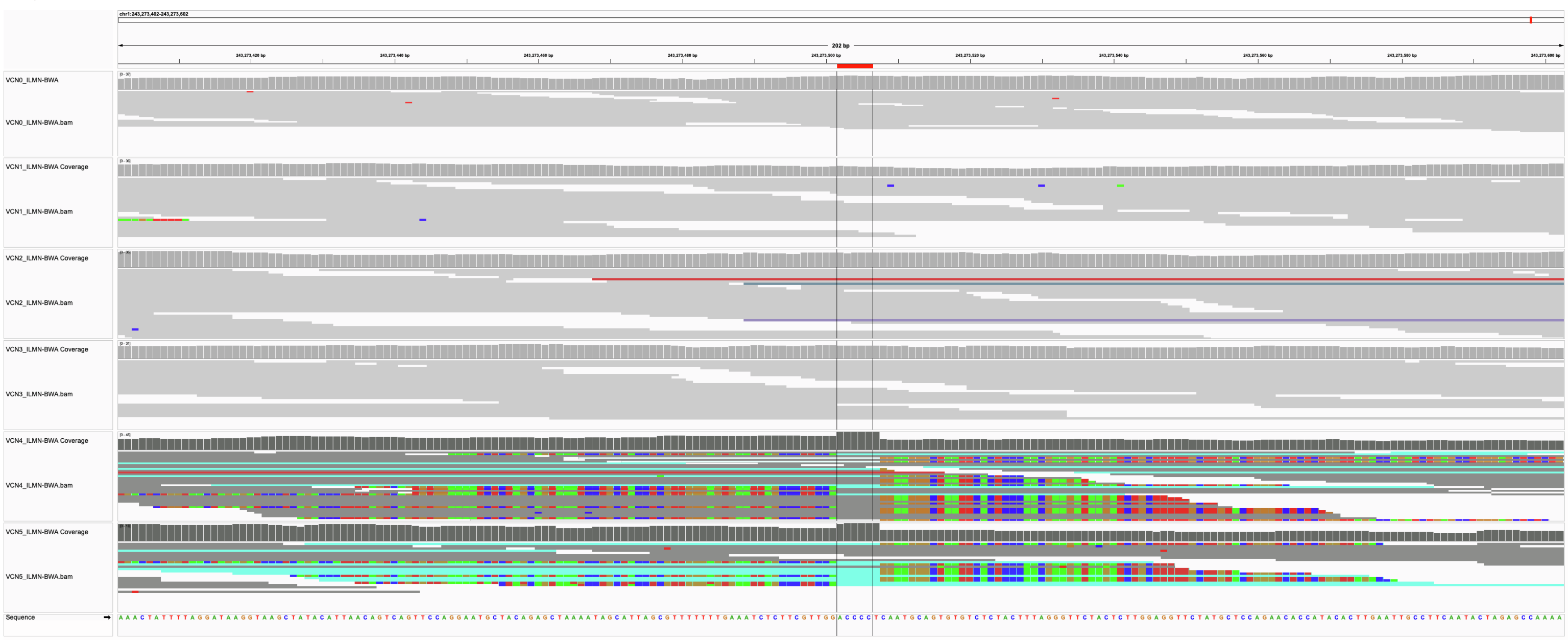

# IS7_VCN4-VCN5_chr3:18115277

## HiFi

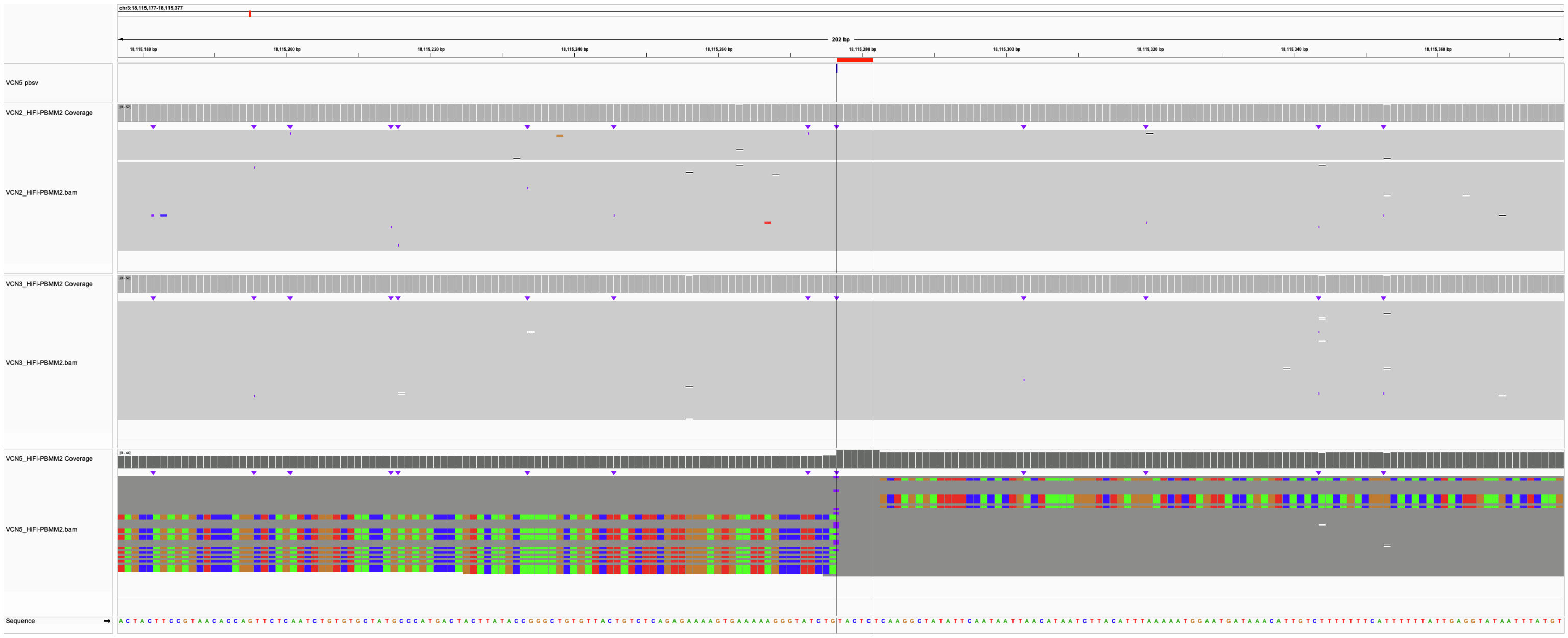

## Illumina

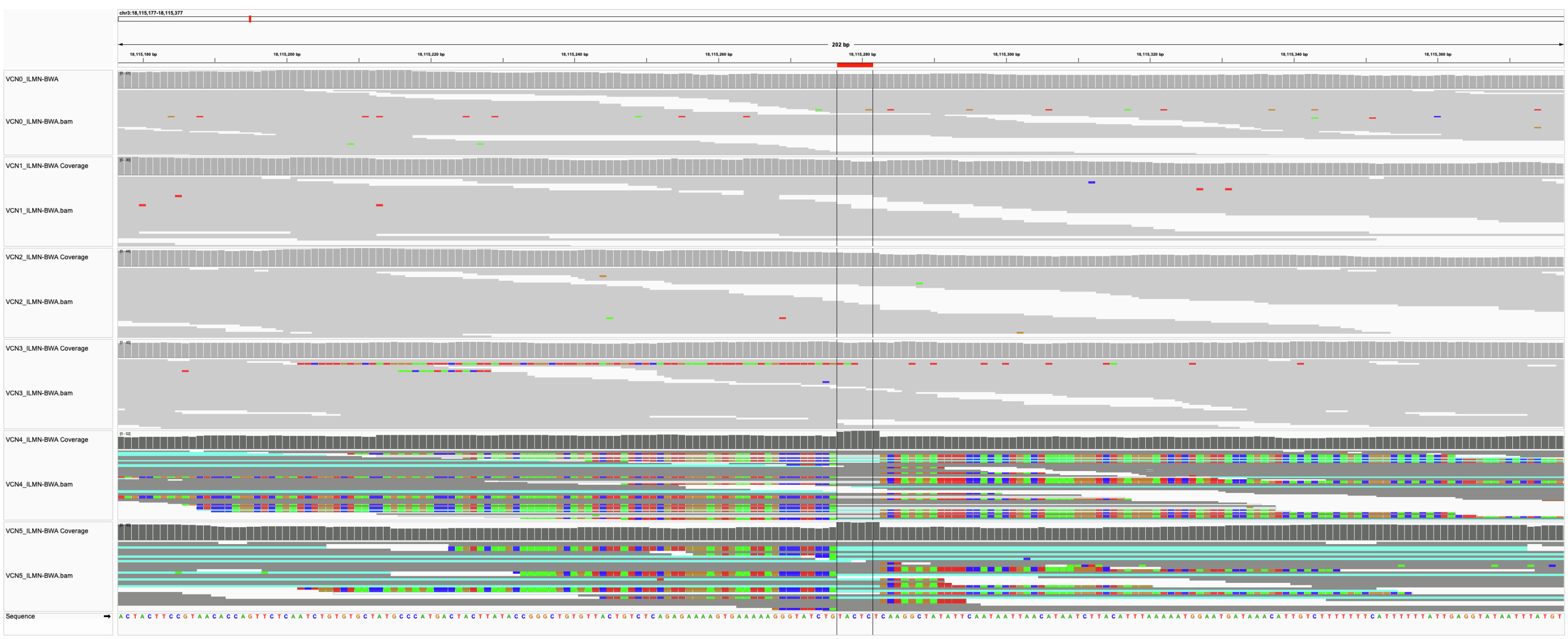

# IS8_VCN4-VCN5_chr6:15601040

## HiFi

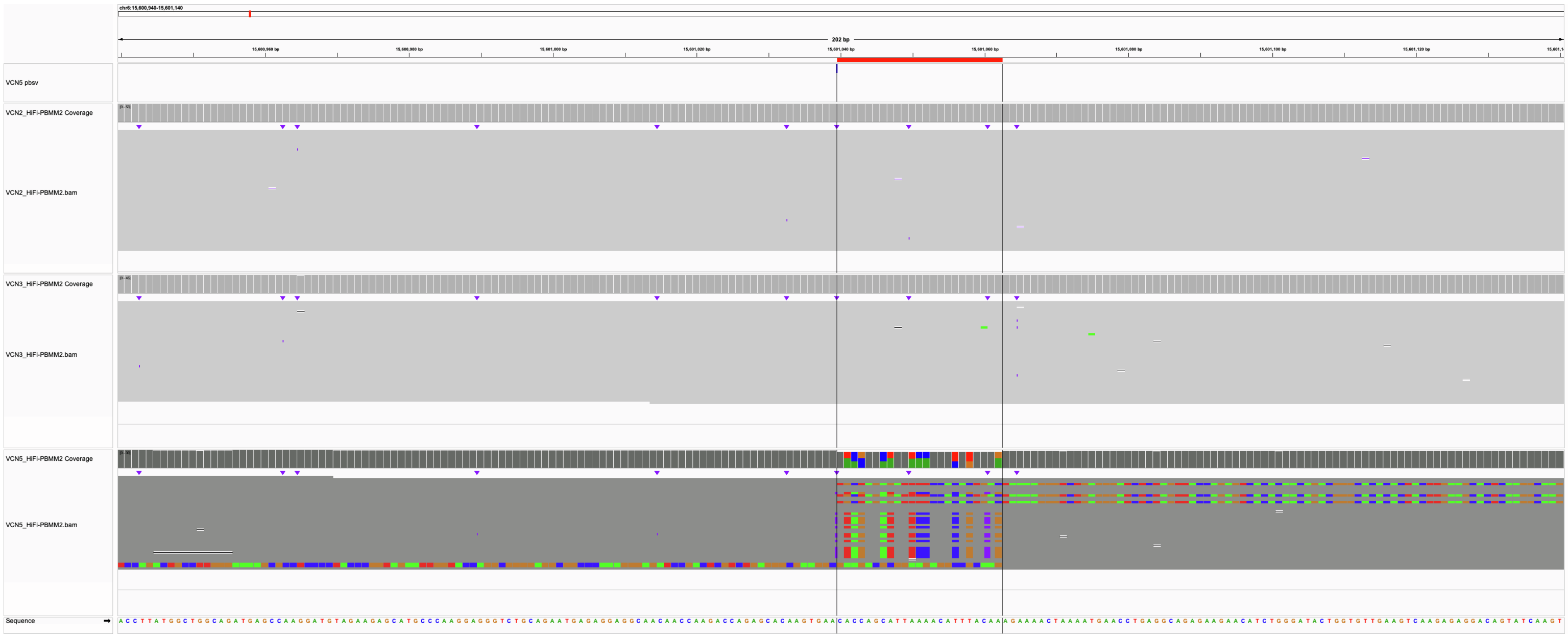

## Illumina

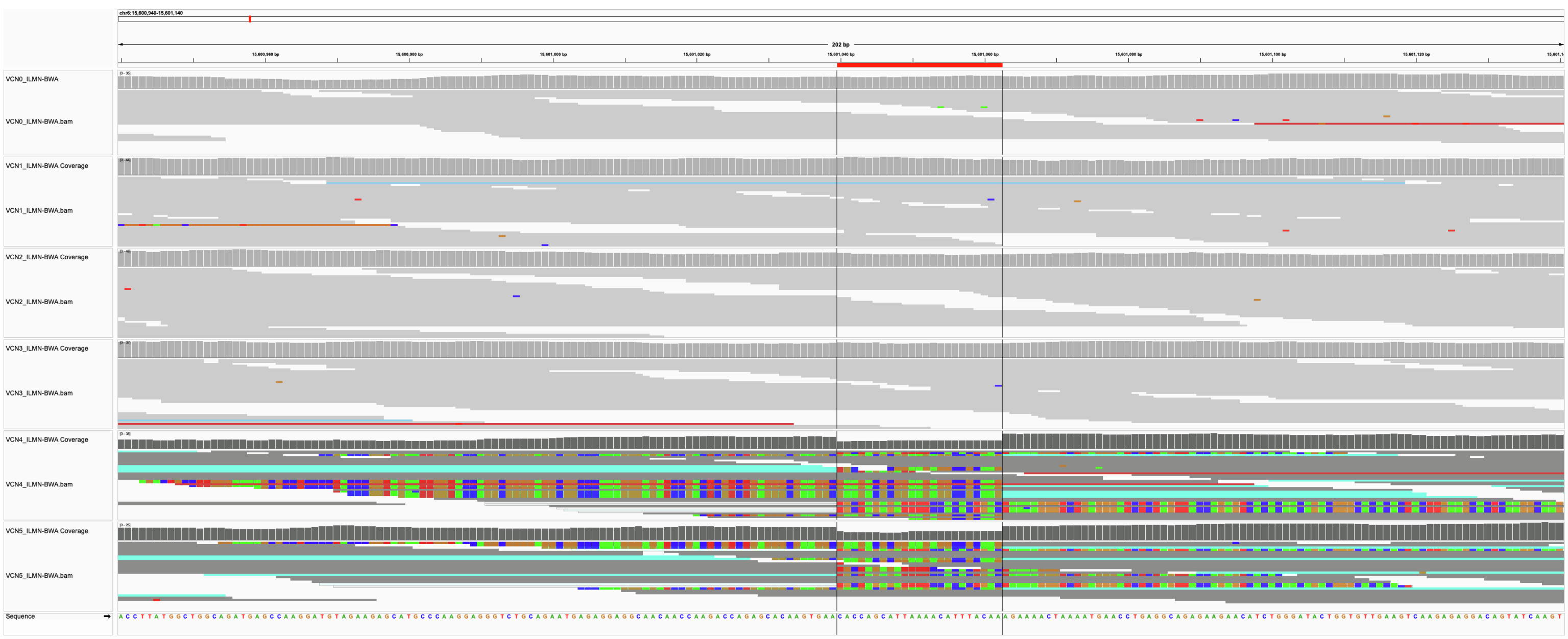

## IS9_VCN4-VCN5_chr6:24825065

### HiFi

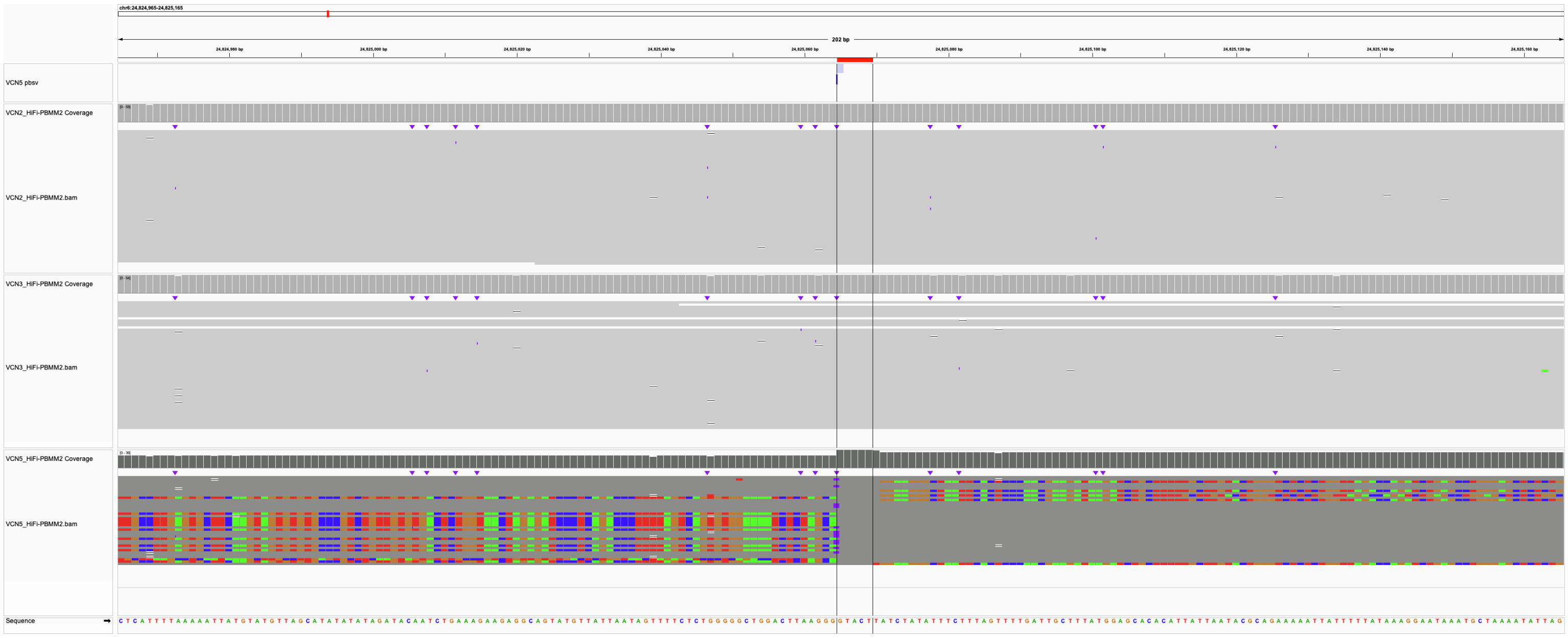

### Illumina

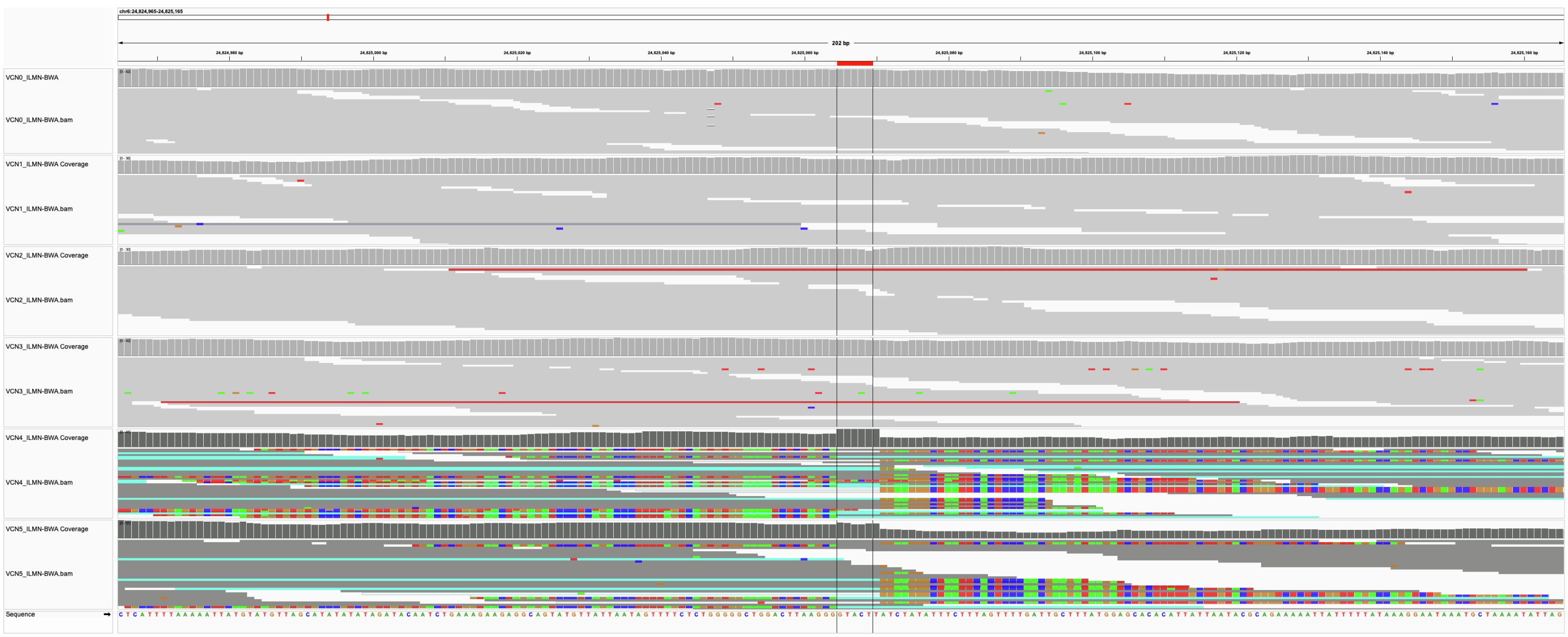

## IS10_VCN5_chr5:39044696

**HiFi**

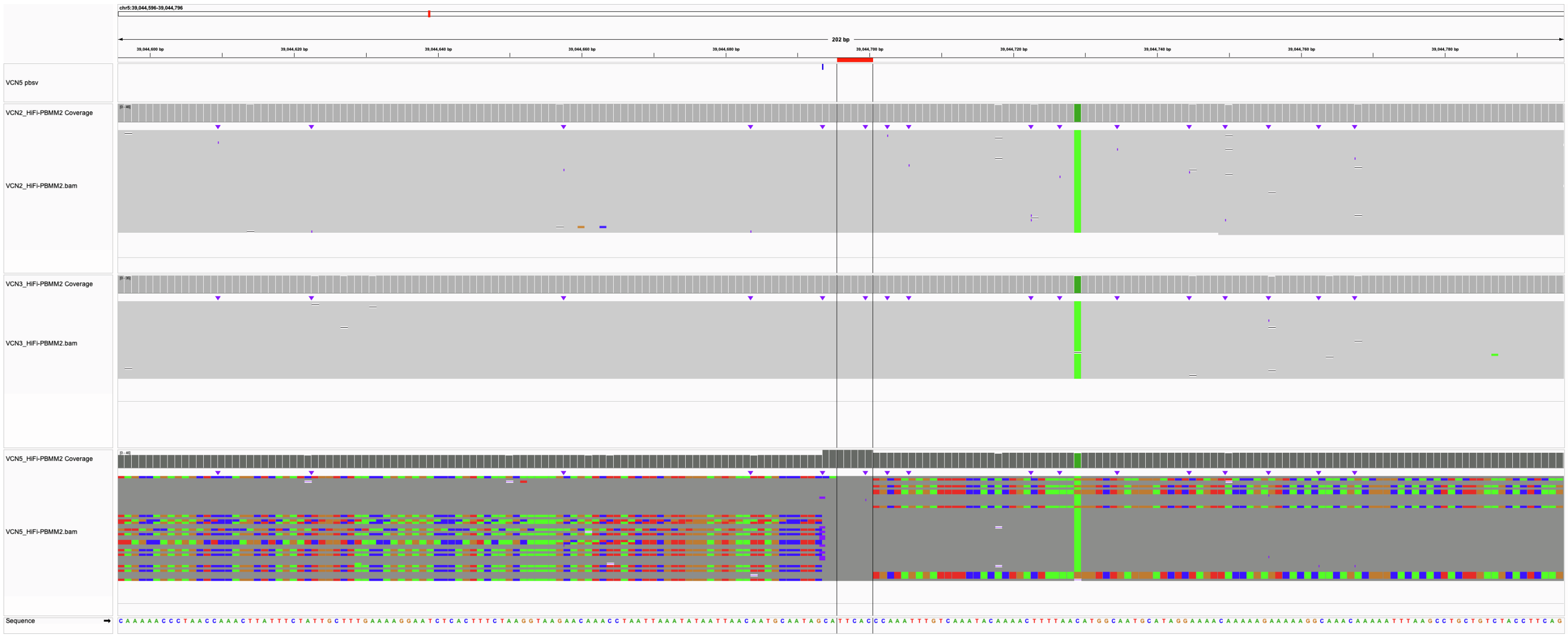

**Illumina**

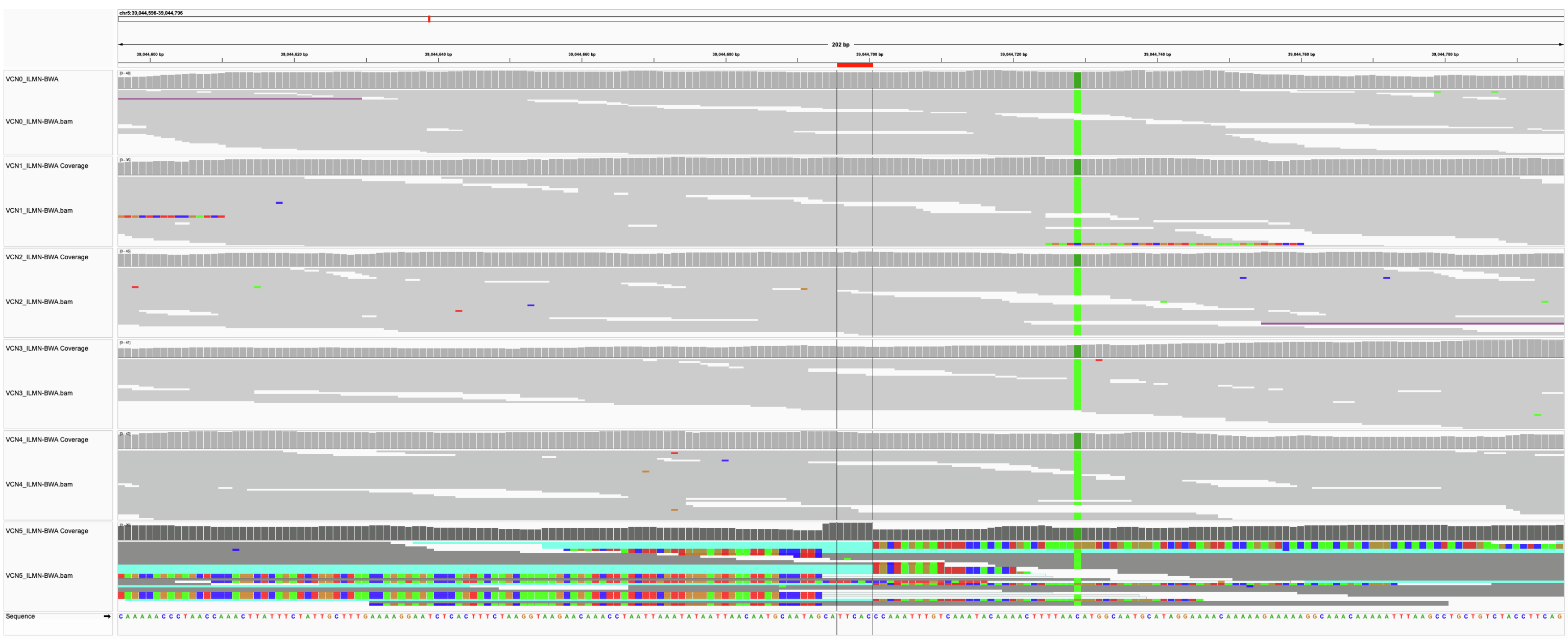

**Figure S3: IGV screenshots of integration sites in Jurkat genome**

Screenshots are grouped by integration site (IS1 through IS10, further described in Table 1). For each integration site, two representative screenshots are provided, corresponding to Figure 1: one for long-read PacBio HiFi data (aligned with pbmm2) and one for short-read Illumina data (aligned with BWA-mem). Tracks for all clones sequenced (VCN0–VCN5 with Illumina and VCN2, VCN3 and VCN5 with HiFi) are displayed for every locus, regardless of whether a specific clone harbors an integration at that site. Clones lacking the integration serve as additional baseline controls alongside VCN0, representing the unaltered Jurkat genome. In each panel, a red bar and black bounding box highlight the precise integration site within the Jurkat genome. To aid visual identification, tracks are shaded darker grey for VCN clones confirmed to contain the specified integration. For clones analyzed with long-read HiFi sequencing, a pbsv insertion call marks the integration site (typically on the left side of the duplicated bases), and read alignments successfully spanning the locus display a large insertion. Conversely, for HiFi read alignments that do not fully span the insertion, as well as for all short-read Illumina data, read alignments terminate abruptly on either side of the

duplicated bases at the integration site. At these boundaries, multi-colored soft-clipped bases are observed, and colored Illumina reads indicate the other read in pair maps to the proviral sequence.
